# Supplementary material for: Observation of unusual outer-sphere mechanism using simple alkenes as nucleophiles in allylation chemistry
Source: Nat Commun. 2024 May 21;15:4317. doi: 10.1038/s41467-024-48541-5 (PMC11109239; doi:10.1038/s41467-024-48541-5)
Supplement: Supplementary file 1 — Supplementary Information [file 41467_2024_48541_MOESM1_ESM.pdf]

Supplementary Information

Observation of unusual outer-sphere mechanism using simple alkenes as nucleophiles in allylation chemistry

Yaxin Zeng[1], Han Gao[2], Zhong-Tao Jiang[1], Yulei Zhu[1], Jinqi Chen[1], Han Zhang[1], Gang Lu[2]* & Ying Xia[1]*

[1]West China School of Public Health and West China Fourth Hospital, West China-PUMC C.C. Chen Institute of Health, and State Key Laboratory of Biotherapy, Sichuan University, Chengdu 610041, China

[2]School of Chemistry and Chemical Engineering, Key Laboratory of Colloid and Interface Chemistry, Ministry of Education, Shandong University, Jinan 250100, China

Email: ganglu@sdu.edu.cn; xiayingscu@scu.edu.cn

CONTENTS

1. General Information 1

2. Reagents and Substrates 2

3. Synthesis of Cationic Dicarbonyl Rhodium Catalysts 8

4. Unsuccessful Substrates 11

5. The Effect of Alkene Configuration on the Allylic Fluorination Reaction 11

6. Product Syntheses and Characterizations 11

7. Synthetic Applications 33

8. The Effect of Feeding Sequence for Rhodium Catalyst Generated In-Situ 38

9. Mechanism Experiments 39

10. X-ray Crystallographic Data 41

11. Computational Details and Additional Computational Results 45

12. NMR Spectra 47

13. References 144

1. General Information

All reactions were carried out under a nitrogen atmosphere. Anhydrous solvents were purchased from Energy Chemical or Adamas in AcroSeal glass bottle (extra dry over molecular sieve) and used directly. NMR spectra were recorded on a JEOL JNM-ECZ400S if noted (400 MHz for ^{1}H, 100 MHz for ^{13}C, 376 MHz for ^{19}F, in which ^{19}F is not H-decoupling in JEOL) with $CDCl_3$ as the solvent and tetramethylsilane (TMS) as the internal standard. Chemical shifts are reported in δ ppm referenced to $CDCl_3$ (δ 7.26 for ^{1}H NMR and δ 77.00 for ^{13}C NMR). The following abbreviations were used to explain the multiplicities: s = singlet, d = doublet, t = triplet, q = quartet, m = multiplet, b = broad. Gas chromatography mass spectrometry (GC-MS) analysis was performed on Agilent Technologies 5975C. High-resolution mass spectrometry (HRMS) was recorded on a Q-TOF (AB SCIEX X500R with ESI source, and Agilent 7250 with EI source), which combines quadrupole precursor ion selection and a high-resolution accurate-mass (HR/AM) Time of Flight mass analyzer to deliver mass accuracy. Flash column chromatography was performed with silica gel (200-300 mesh, Haiyang, Qingdao). Melting points were determined using a digital melting point apparatus (JHX-4).

2. Reagents and Substrates

Unless stated otherwise, commercially available reagents were used as supplied. [Rh(COD)BF_4] was purchased from LAAJOO. [Rh$(CO)_2$Cl]$_2$, [Rh$(COD)_2$Cl]$_2$, BINAP, $AgBF_4$, and all ultra dry solvents were purchased from Energy. *gem*-Difluorinated cyclopropanes were prepared according to the known procedure[1-4] and the structure of these substrates are listed in **Supplementary Figure 1**. Alkenes **2a-2e**, **2i**, **2j**, and **2m** are commercially available and were used as supplied. Other alkenes were prepared according to the known procedure[5-19]. The structures of these substrates are listed in **Supplementary Figure 2**. BINAP and BINAP^{Me} are commercially available and were used as supplied (**Supplementary Figure 3**). Other BINAP derivatives such as BINAP^{OMe} and BINAP^{NMe2} were synthesized according to the known procedure[20-23].

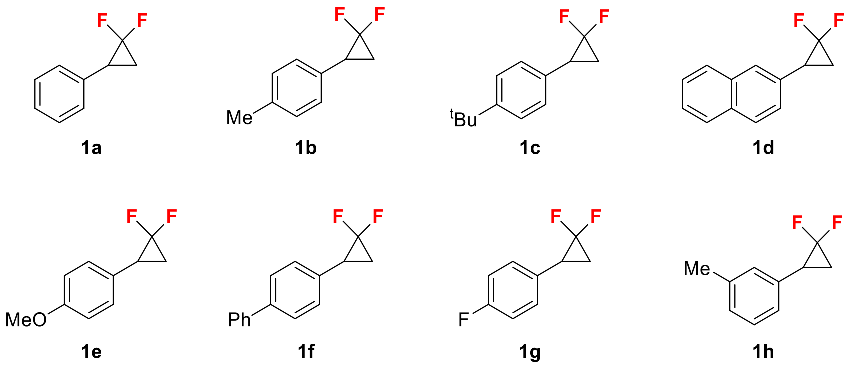

Supplementary Figure 1. *gem*-Difluorinated cyclopropanes used in this work

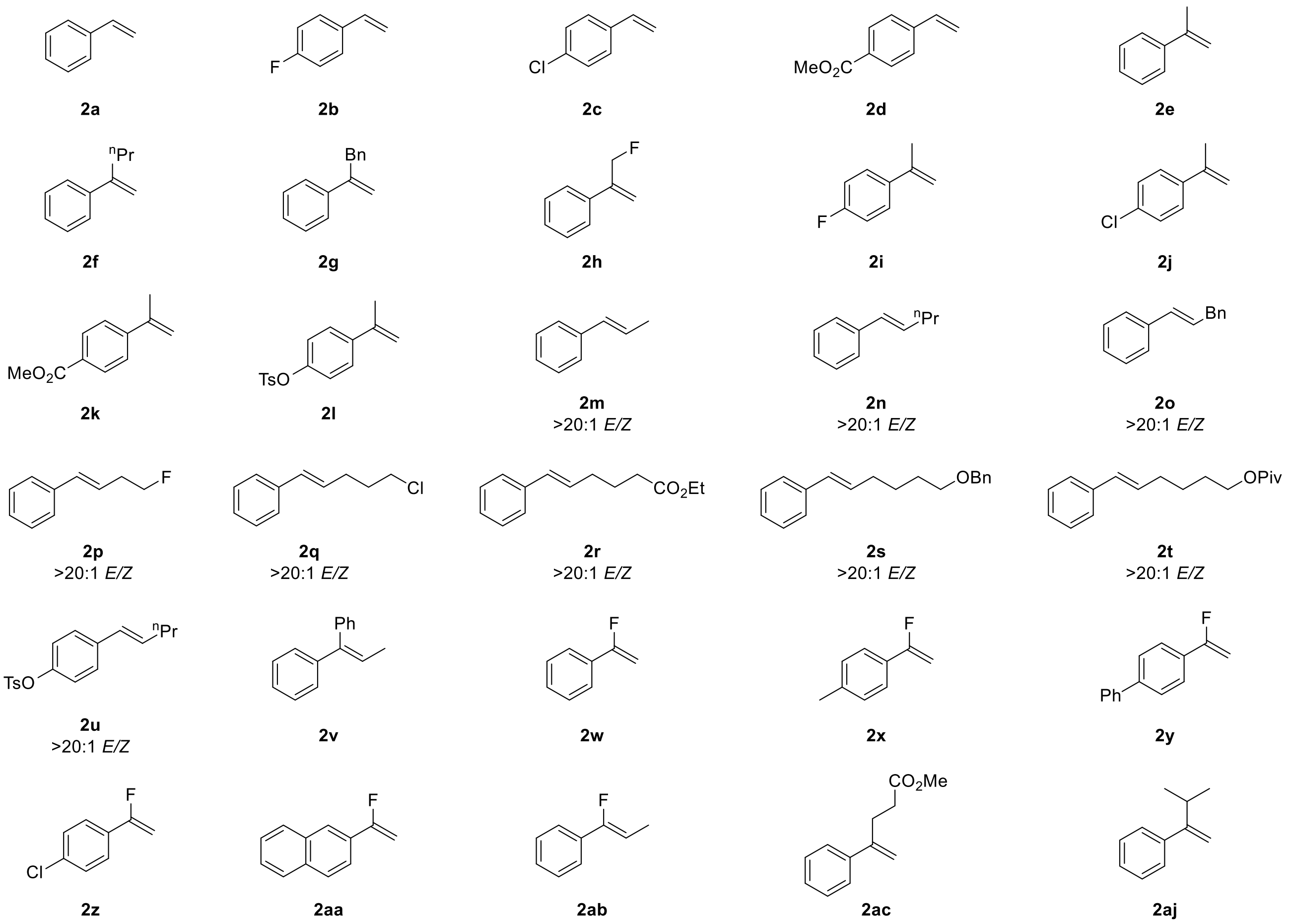

Supplementary Figure 2. Alkenes used in this work

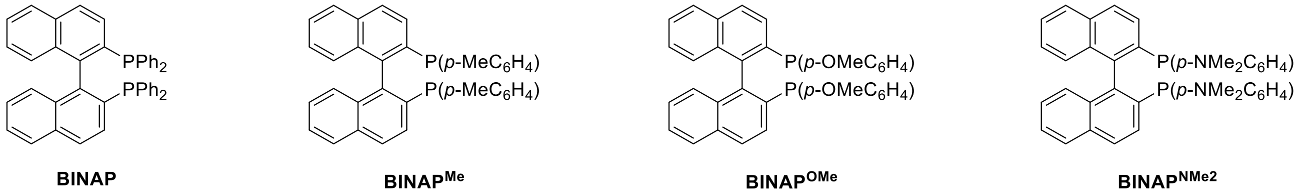

Supplementary Figure 3. BINAP derivatives used in this work

Synthesis of *gem*-Difluorinated Cyclopropanes

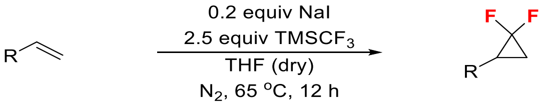

To a flame-dried 100 mL three-necked flask equipped with a magnetic stir bar was added anhydrous NaI (0.3 g, 0.2 equiv), dry THF (20 mL), $TMSCF_3$ (3.7 mL, 2.5 equiv) and corresponding alkenes (10.0 mmol) under nitrogen atmosphere. The flask was sealed and stirred at 65 °C for 12 hours. The reaction mixture was then cooled to room temperature, which was evaporated to dryness under

reduce pressure and directly filtered through a pad of celite. The crude mixture was extracted with ethyl acetate (20 mL) and washed with saturated sodium sulfite solution (20 mL), brine (20 mL). The organic layer was dried over $MgSO_4$, filtered and concentrated, which was purified by silica gel column chromatography to afford corresponding *gem*-difluorinated cyclopropanes. *gem*-Difluorinated cyclopropanes **1a**[1], **1b**[2], **1c**[2], **1d**[3], **1e**[2], **1f**[1], **1g**[1], and **1h**[4] are literature reported compounds.

Synthesis of Alkenes:

Procedure 1:

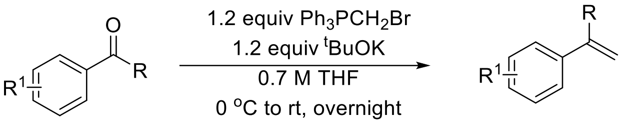

To a stirred suspension of methyltriphenylphosphonium bromide (2.14g, 6 mmol) in THF (10 mL) was added potassium *tert*-butoxide (673.3 mg, 6 mmol). The mixture was stirred for 30 min at 0 °C and became orange. Then, aldehyde (5 mmol) was added and the resulting mixture was further stirred overnight after warming to room temperature. The crude mixture was diluted with petroleum ether, filtered and concentrated, which was purified by silica gel column chromatography to afford corresponding alkenes. Alkenes **2f**[5], **2g**[5], **2k**[6], **2l**[7], **2ac**[8], and **2aj**[9] are literature reported compounds.

Procedure 2:

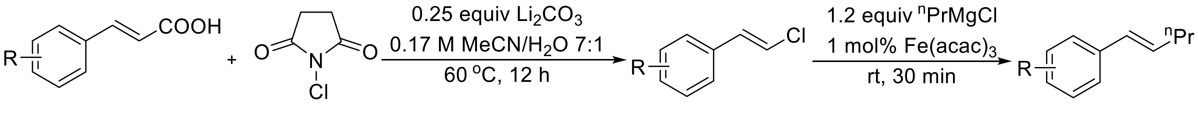

Following the Noël's procedure[10], to a flame-dried 250 mL three-necked flask equipped with a magnetic stir bar was added *trans*-cinnamic acid, *N*-chlorosuccinimide (1.33 g, 10 mmol), Li_2CO_3 (92.4 mg, 1.25 mmol), MeCN (28 mL), and H_2O (4 mL) under nitrogen atmosphere. After stirring at 60 °C for 12 hours, the reaction mixture was then cooled to room temperature, which was diluted with H_2O (20 mL) and extracted with ethyl acetate. The combined organic layer was washed with saturated brine, dried over $MgSO_4$, filtered, and concentrated, which was purified by silica gel column chromatography to afford the corresponding styryl chlorides. The styryl chloride from the last step was added to a solution of $Fe(acac)_3$ in dry THF. Then, AlkylMgCl (1M in THF) was added via syringe and the resulting mixture was stirring vigorously for 30 min at room temperature. After completion of this

reaction, the reaction mixture was quenched with a saturated aqueous solution of NH_4Cl and extracted with ethyl acetate. The combined organic layer was washed with brine, then dried over $MgSO_4$, filtered, and concentrated under vacuum. Purification by column chromatography on silica gel afforded the corresponding *E*-alkene. Alkenes **2n** and **2o** are synthesized by **Procedure 2**, which are literature reported compounds[10].

Procedure 3:

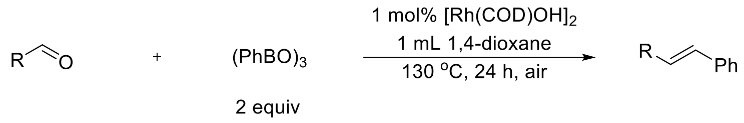

Following our procedure[11], a 8 mL vial equipped with a magnetic stir bar was charged with $[Rh(COD)OH]_2$ (9.2 mg, 0.02 mmol), phenylboroxine (415 mg, 1.33 mmol), and 1,4-dioxane in a nitrogen filled glove box. After the vial was evacuated and filled with air, aldehyde was added and the reaction mixture was stirred at 130 °C for 24 hours. After the reaction was completed, the reaction mixture was diluted with EtOAc and purified by chromatography on silica gel column to give the corresponding *E*-alkenes. Alkenes **2q-2t** are synthesized by **Procedure 3**, which are literature reported compounds[11].

Procedure 4:

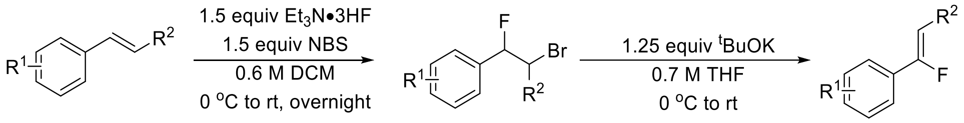

All α-fluorostyrenes were synthesized according to a literature known procedure[12], $NEt_3{\cdot}3HF$ (1.21 g, 7.5 mmol, 1.5 equiv) was added dropwise to a stirred solution of corresponding alkenes (5.0 mmol, 1.0 equiv) and *N*-bromosuccinimide (1.34 g, 7.5 mmol) in anhydrous DCM (8.3 mL) at 0 °C. After warming to room temperature, the reaction mixture was stirred overnight. The resulting mixture was quenched with saturated $NaHCO_3$ and extracted with DCM. The combined organic layer was washed with 1 M HCl, then dried over $MgSO_4$, filtered, and concentrated under vacuum. The residual crude was used for the next step without further purification.

The crude product was added to a solution of ^tBuOK (700 mg, 1.25 equiv) in THF (7 mL) at 0 °C. The reaction mixture was warm to room temperature and stirred overnight. After the reaction was

completed, the resulting mixture was filtered through a pad of celite. The crude mixture was concentrated in vacuo, which was purified by silica gel column chromatography to afford corresponding α-fluorostyrenes. α-Fluorostyrenes **2w**[13], **2x**[13], **2y**[14], **2z**[13], **2aa**[15], and **2ab**[13] are synthesized by **Procedure 4**, which are literature reported compounds.

Synthesis of Alkene 2h

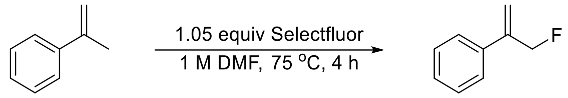

To a stirred suspension of Selectfluor (743 mg, 2.1 mmol) in DMF (2 mL) was added α-methyl styrene (236 mg, 2 mmol). The mixture was stirred for 4 h at 75 °C. After completion of this reaction, the reaction mixture was diluted with EtOAc, washed with H_2O and extracted with ethyl acetate. The combined organic layer was washed with brine, then dried over $MgSO_4$, filtered, and concentrated under vacuum. Purification by column chromatography on silica gel afforded the alkene **2h**[16].

Synthesis of Alkene 2v

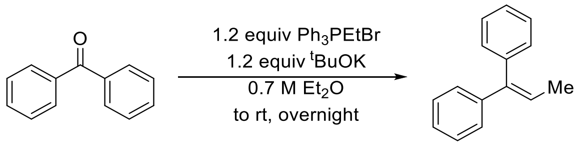

To a stirred suspension of ethyltriphenylphosphonium bromide (2.23 g, 12 mmol) in Et_2O (10 mL) was added potassium *tert*-butoxide (1.35 g, 12 mmol). The mixture was stirred for 30 min at 0 °C and became orange red. Then, aldehyde (5 mmol) was added and the resulting mixture was further stirred overnight after warming to ambient temperature. The crude mixture was diluted with petroleum ether, filtered and concentrated, which was purified by silica gel column chromatography to afford alkene **2v**[17].

Synthesis of Alkene 2p

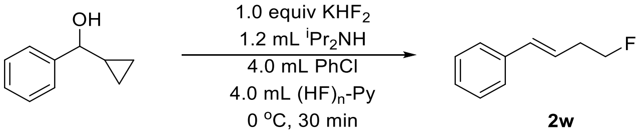

2w

Following the Yoshioka's procedure[18], to a stirred solution of cycloprorylphenylmethanol (296

mg, 2 mmol) and KHF_2 (156 mg, 2 mmol) in PhCl (4.0 mL) was added diisopropylamine (4.0 mL). After stirring at room temperature for 10 min, the $(HF)_n$-pyridine (4.0 mL) was added slowly to this reaction mixture at 0 °C for 10 min and the reaction mixture was stirred at 0 °C for 30 min. After the competition of the reaction, the mixture was quenched with aq. KF and extracted with ethyl acetate. The combined organic layer was washed with saturated aq. $NaHCO_3$ and brine, then dried over $MgSO_4$, filtered, and concentrated under vacuum. Purification by column chromatography on silica gel afforded the alkene **2w** as a colorless oil[19].

Synthesis of BINAP Derivatives

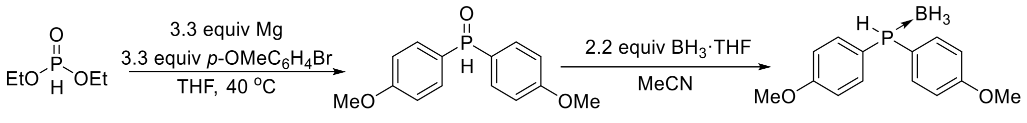

P-1 **P-2** **P-3**

To a suspension of magnesium (705.6 mg, 29.4 mmol) in THF (4 mL) was added slowly a solution of 4-bromoanisole (3.74 g, 20 mmol) and THF (15 mL) at 40 °C. After stirring for 1 h, the resulting mixture was allowed to warm to room temperature. A solution of diethyl phosphite (1.38 g, 10 mmol) in THF (10 mL) was added, and the mixture was stirred at room temperature for 12 h. Ather the completion of this reaction, the reaction mixture was quenched with a saturated aqueous solution of NH_4Cl and extracted with ethyl acetate. The combined organic layer was washed with brine, then dried over $MgSO_4$, filtered, and concentrated under vacuum. Purification by column chromatography on silica gel afforded the diarylphosphine oxide **P-2**[20].

Diarylphosphine oxide **P-2** (2.62 g, 10 mmol) and MeCN (10 mL) was added to a flame-dried 100 mL three-necked flask equipped with a magnetic stir bar under nitrogen atmosphere. BH_3·THF (20 mL, 1 M, 2 equiv) was added to the stirred solution of diarylphosphine oxide at a constant rate (0.10 mmol/min) at room temperature. The resulting reaction was stirred for 1 h at room temperature and was purified by column chromatography on silica gel to afford bisarylphosphine-borane **P-3** (PE/DCM 1:1, Rf=0.5)[21].

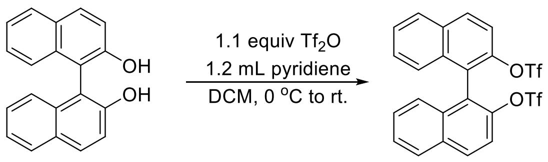

N-1 **N-2**

To a solution of *rac*-BINOL (1.14 g, 4.0 mmol, 1 equiv) in 25 mL DCM was added pryridine (1.2 mL) and followed by dropwise addition of triflic anhydride at 0 °C. The resulting mixture was allowed to warm to room temperature and stirred overnight. After removal of the solvent, the residue was diluted with EtOAc and then washed with 5% aqueous HCl, saturated $NaHCO_3$ and brine. The organic layer was dried over $MgSO_4$, filtered, and concentrated under vacuum. Purification by column chromatography on silica gel afforded the di(trifluoromethylsulfonyl)-1,1'-binaphthyl **N-2** as a white solid[22].

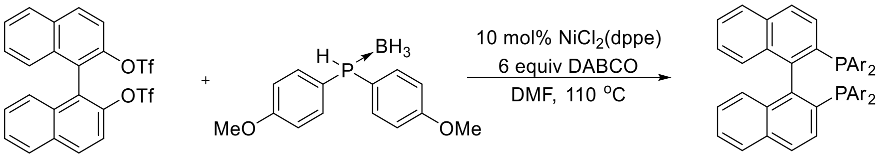

N-2 **P-3** **BINAP^{OMe}**
Ar = *p*-OMeC$_6$H$_4$

To a flame-dried 100 mL three-necked flask equipped with a magnetic stir bar was added di(trifluoromethylsulfonyl)-1,1'-binaphthyl **N-2** (1,1 g, 2 mmol), bisarylphosphine-borane **P-3** (4.6 mmol, 2.3 equiv), and $NiCl_2$(dppe) (108.4 mg, 0.2 mmol), DABCO (1.37 g, 12 mmol, 6 equiv), and DMF (20 mL, 0.1 M). The resulting mixture was stirred at 110 °C for 18 h, and then concentrated under reduce pressure. The crude residue was washed with methanol to give the BINAP^{OMe} as a white solid. Similarly, BINAP^{NMe2} is also synthesized as a pale yellow solid according to the above procedure[23].

3. Synthesis of Cationic Dicarbonyl Rhodium Catalysts

Method 1:

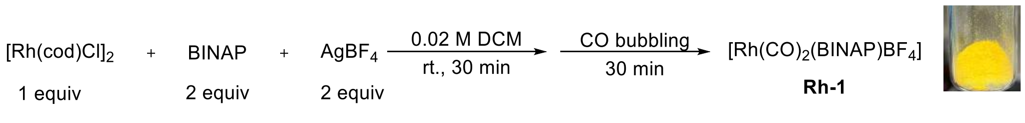

Method 2:

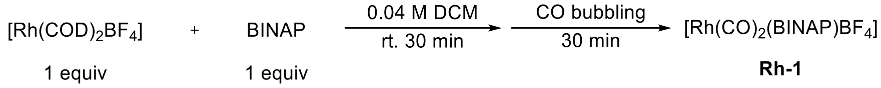

In a nitrogen filled glove box, $[Rh(cod)Cl]_2$ (162.4 mg, 0.2 mmol, 1 equiv) and BINAP (249.0 mg, 0.4 mmol, 2 equiv), and $AgBF_4$ (77.9 mg, 0.4 mmol, 2 equiv) were dissolved in 10 mL dry DCM. The solution was stirred for 1 h, giving an orange suspension. This reaction mixture was removed from the glove box, and CO was bubbling into the resulting solution for 30 min and during this time the solution become bright yellow. Conveniently, the subsequent processing steps can all be carried out under air atmosphere. The catalyst mixture was directly filtered through a pad of celite and then the organic layer was concentrated under vacuum to a volume of approximately 1 mL. This concentrate solution was added dropwise to a flame-dried 250 mL conical flask equipped with a magnetic stir bar and 100 mL diethyl ether. The resulting yellow solid that precipitated was collected by filtration using filter paper and washed with diethyl ether, giving cationic dicarbonyl rhodium catalyst **Rh-1** as a bright yellow solid (341 mg, 98% yield). [24] An alternative method involves using $[Rh(COD)_2BF_4]$ as a precursor for the rhodium catalyst, which also results in the synthesis of **Rh-1**. Both methods yield **Rh-1** with the same catalytic reactivity in the reaction. The structure of $[Rh(CO)_2(BINAP)BF_4]$ was unambiguously verified by X-ray analysis (see section 10).

Note: While investigating this work, this series of Rh-catalysts was consistently kept outside. So far, there have been no irregularities in its catalytic activity and structure, indicating its stability in an air atmosphere.

[Rh(CO)₂(BINAP)BF₄]

Following the **method 1** or **method 2**. Isolated yield = 98%; Yellow solid. **^{1}H NMR** (400 MHz, $CDCl_3$) δ 7.86 – 7.79 (m, 4H), 7.65 – 7.60 (m, 10H), 7.45 – 7.32 (m, 8H), 7.13 (ddd, *J* = 8.3, 6.8, 1.2 Hz, 2H), 6.94 (t, *J* = 7.5 Hz, 2H), 6.80 (t, *J* = 7.1 Hz, 4H), 6.64 (d, *J* = 8.7 Hz, 2H). **^{13}C NMR** (101 MHz, $CDCl_3$) δ 182.9 – 181.0 (m, CO), 139.0 (t, *J* = 7.2 Hz), 134.9 (t, *J* = 5.9 Hz), 134.3 (t, *J* = 5.5 Hz), 134.1, 133.0 (t, *J* = 3.6 Hz), 132.2, 131.1, 130.6 (probable dt, superimposed), 129.6 (t, *J* = 5.0 Hz), 129.3 (t, *J* = 5.3 Hz), 128.5 – 128.2 (m), 128.1, 127.1, 127.0 – 126.2 (m) (probable dt, superimposed), 126.9, 123.5 – 122.6 (m). ^{31}P NMR (162 MHz, CDCl3) δ 22.73 (d, *J* = 125.2 Hz). **^{19}F NMR** (376 MHz, $CDCl_3$) δ -152.98, -153.03.

$[Rh(CO)_2(BINAP^{Me})BF_4]$

Following the **method 1** or **method 2**. Isolated yield = 95%; Yellow solid. **^{1}H NMR** (400 MHz, CD_2Cl_2) δ 7.72 – 7.57 (m, 8H), 7.46 – 7.37 (m, 6H), 7.31 (dd, *J* = 10.9, 8.7 Hz, 2H), 7.19 – 7.13 (m, 4H), 7.12 (ddd, *J* = 8.3, 6.8, 1.2 Hz, 2H), 6.62 (dd, *J* = 8.7, 1.0 Hz, 2H), 6.55 (d, *J* = 7.3 Hz, 4H), 2.44 (s, 6H), 2.02 (s, 6H). **^{13}C NMR** (101 MHz, CD_2Cl_2) δ 184.4 – 181.8 (m, CO), 143.4, 142.6, 139.5 (t, *J* = 7.2 Hz), 135.3 (t, *J* = 7.2 Hz), 134.7, 134.6 (superimposed), 133.5 (t, *J* = 3.7 Hz), 130.3 (t, *J* = 5.5 Hz), 129.7 (t, *J* = 5.0 Hz, superimposed),129.6 (t, *J* = 6.2 Hz, superimposed), 128.7, 128.4, 128.3 – 127.6 (m), 127.5, 127.5, 127.4 – 127.2 (m), 121.1 – 119.8 (m), 21.6, 21.3. **^{31}P NMR** (162 MHz, CD_2Cl_2) δ 20.95 (d, *J* = 124.3 Hz). **^{19}F NMR** (376 MHz, CD_2Cl_2) δ -152.81, -152.87.

[Rh(CO)$_2$(BINAP^{OMe})BF$_4$]

Following the **method 1** or **method 2**. Isolated yield = 98%; Yellow solid. **^{1}H NMR** (400 MHz, $CDCl_3$) δ 7.75 – 7.68 (m, 4H), 7.65 (d, *J* = 8.3 Hz, 4H), 7.47 – 7.41 (m, 2H), 7.36 – 7.29 (m, 2H), 7.22 (s, 4H), 7.15 – 7.09 (m, 6H), 6.66 (d, *J* = 8.6 Hz, 2H), 6.29 (d, *J* = 7.6 Hz, 4H), 3.90 (s, 6H), 3.62 (s, 6H). **^{13}C NMR** (101 MHz, $CDCl_3$) δ 184.1 – 180.5 (m), 162.2, 161.7, 138.8 (t, *J* = 7.2 Hz), 136.5, 136.0 (t, *J* = 6.2 Hz), 134.2, 133.0 (t, *J* = 3.6 Hz), 128.1, 128.0 – 127.4 (m), 127.9 (d, *J* = 4.5 Hz), 127.2 – 127.1 (m), 127.0, 127.0, 122.1 – 121.1 (m), 114.9 – 114.6 (m), 114.2 (t, *J* = 6.6 Hz), 114.3 – 113.8 (m), 55.6, 55.2. **^{31}P NMR** (162 MHz, $CDCl_3$) δ 19.67 (d, *J* = 124.9 Hz). **^{19}F NMR** (376 MHz, CDCl3) δ -153.22, -153.28.

[Rh(CO)$_2$(BINAP^{NMe2})BF$_4$]

Following the **method 1** or **method 2**. Isolated yield = 90%; Yellow solid. **^{1}H NMR** (400 MHz, $CDCl_3$) δ 7.59 (dt, *J* = 19.9, 9.7 Hz, 8H), 7.43 – 7.31 (m, 4H), 7.14 – 6.99 (m, 6H), 6.79 (d, *J* = 8.3 Hz, 4H), 6.64 (d, *J* = 8.6 Hz, 2H), 5.94 (d, *J* = 8.1 Hz, 4H), 3.06 (s, 12H), 2.78 (s, 12H). **^{13}C NMR** (101 MHz, $CDCl_3$) δ 183.0 (ddd, *J* = 102.8, 60.8, 24.2 Hz, CO), 151.7, 151.2, 138.8 (t, *J* = 7.1 Hz), 136.0, 135.2 (t, *J* = 6.2 Hz), 134.2, 133.1 (t, *J* = 3.6 Hz), 129.0 (t, *J* = 5.0 Hz), 128.9 – 128.0 (m), 127.9, 127.3, 127.2, 126.4, 116.1 – 115.0 (m), 111.3 (dt, *J* = 17.8, 6.0 Hz), 108.4 – 107.6 (m), 39.8.0, 39.6. **^{31}P NMR** NMR (162 MHz, CDCl3) δ 18.25 (d, *J* = 123.2 Hz). **^{19}F NMR** (376 MHz, $CDCl_3$) δ -154.11, -154.16.

4. Unsuccessful Substrates

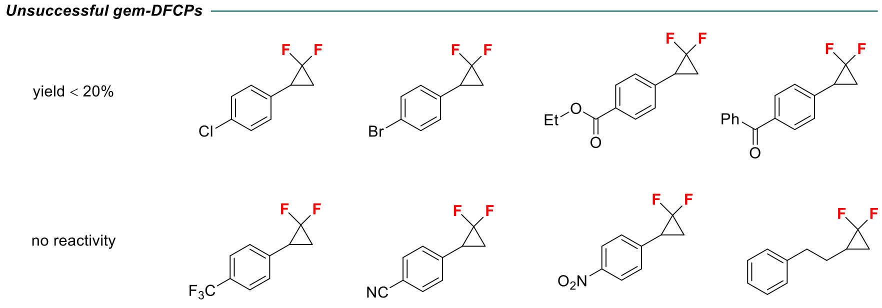

Supplementary Figure 4. Unsuccessful *gem*-DFCPs

gem-DFCPs containing chlorine, bromine, ester, and carbonyl group led to the yields of corresponding product below 20% with 5-20% conversions, while *gem*-DFCPs bearing strong with-drawing groups such as trifluoromethyl, cyan and nitro group, and alkyl-substituted *gem*-DFCPs have no reactivity.

5. The Effect of Alkene Configuration on the Allylic Fluorination Reaction

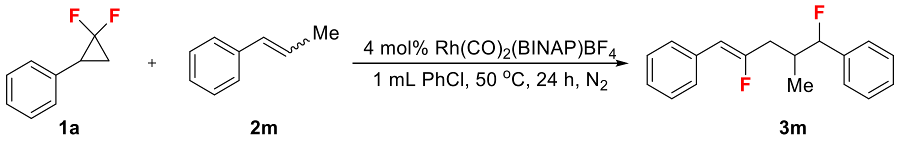

entry	alkene	time	yield of **3a**[a]	dr
1	*E*-**2m**	24 h	92%(88%[b])	1.3:1
2	**2m** (E:Z 1:1)	24 h	75%	1.5:1
3	*Z*-**2m**	24 h	50%	2.0:1
4	*Z*-**2m**	48 h	66%(60%[b])	1.3:1

[a]Yield was determined by ^{1}H NMR using the 1,1,2,2-tetrachloroethane as the internal standard.
[b]Isolated yield

Supplementary Table 1. The effect of alkenes configuration

We investigated the effect of the configuration of 1,2-disubstituted alkenes at different ratio of E/Z under otherwise the same conditions. (see below). When using 1:1 *E/Z* mixture of alkene **2m**, the yield of **3m** was decreased to 75% with 1.5:1 dr. Treating the pure *Z*-**2m** under standard conditions resulted in a 50% yield of product **3m** with a 2.0:1 dr and incomplete conversion. Prolonging the reaction time to 48 hours increased the yield of **3m** to 66% with a comparable dr value.

6. Product Syntheses and Characterizations

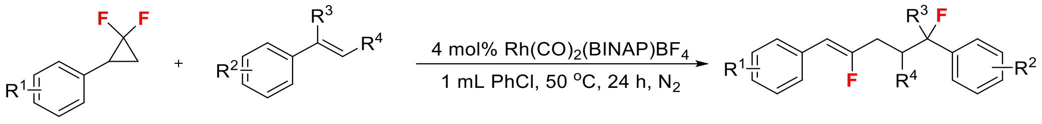

1, 0.15 mmol **2**, 0.1 mmol **3** or **4**

In a nitrogen filled glove box, a 4 mL vial equipped with a stir bar was charged with [Rh(CO)$_2$(BINAP)BF$_4$] (3.5mg, 0.004 mmol, 4 mol%) and PhCl (1 mL). After stirring at room temperature for about 5 min, *gem*-difluorinated cyclopropane **1** (0.15 mmol) and the corresponding alkene **2** (0.1 mmol) was added to the resulting yellow catalyst solution. The 4 mL vial was sealed and removed from the glove box and stirred at 50 °C for 24 hours. The reaction mixture was cooled to room temperature and purified by chromatography on silica gel column to give the fluorides **3** or **4**. Note that this reaction is sensitive for air, requiring a relatively strict inert atmosphere and sealing. All isolated yields and mass of carbofluorination products are the average of three runs.

(Z)-(2,5-difluoropent-1-ene-1,5-diyl)dibenzene (3a)

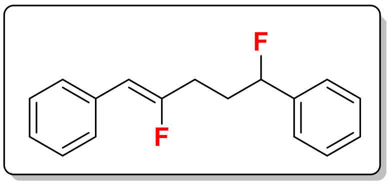

Following the general procedure (eluting with PE/DCM = 20/1). Isolated yield = 88% (22.8 mg); Colorless oil; Rf = 0.2 (PE); **^{1}H NMR** (400 MHz, CDCl$_3$) δ 7.46 (dt, *J* = 6.9, 1.5 Hz, 2H), 7.41 – 7.27 (m, 7H), 7.20 (tt, *J* = 6.6, 1.3 Hz, 1H), 5.52 (ddd, *J* = 47.5, 8.3, 4.4 Hz, 1H), 5.52 (d, *J* = 39.2 Hz, 1H), 2.56 – 2.43 (m, 2H), 2.31 – 2.01 (m, 2H); **^{13}C NMR** (101 MHz, CDCl$_3$) δ 159.5 (d, *J* = 266.2 Hz), 139.7 (d, *J* = 19.7 Hz), 133.5 (d, *J* = 2.5 Hz), 128.5, 128.43 (d, *J* = 1.9 Hz), 128.42, 128.3 (d, *J* = 7.4 Hz), 126.9 (d, *J* = 2.2 Hz), 125.5 (d, *J* = 6.8 Hz), 106.6 (d, *J* = 8.5 Hz), 93.3 (d, *J* = 171.6 Hz), 33.9 (d, *J* = 24.2 Hz), 28.9 (dd, *J* = 27.3, 4.4 Hz); **^{19}F NMR** (376 MHz, CDCl$_3$) δ -102.04 (dt, *J* = 39.3, 17.7 Hz), -176.90 (ddd, *J* = 47.6, 30.2, 17.1 Hz). **HRMS** (ESI, m/z): calcd for $C_{17}H_{16}F_2$ [M+Na]$^+$ 281.1112, found 281.1116.

(Z)-1-(1,4-difluoro-5-phenylpent-4-en-1-yl)-4-fluorobenzene (3b)

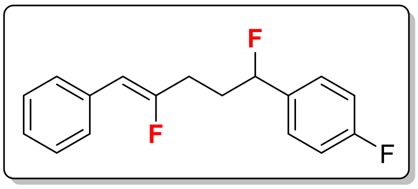

Following the general procedure (eluting with PE/DCM = 20/1). Isolated yield = 56% (15.4 mg); Colorless oil; Rf = 0.2 (PE); **^{1}H NMR** (400 MHz, CDCl$_3$) δ 7.46 (dt, *J* = 6.7, 1.4 Hz, 2H), 7.32 (ddt, *J* = 8.0, 6.4, 2.4 Hz, 4H), 7.22 (tt, *J* = 6.7, 1.3 Hz, 1H), 7.12 – 7.03 (m, 2H), 5.53 (d, *J* = 39.5 Hz, 1H), 5.50 (ddd, *J* = 47.6, 8.9, 4.6 Hz, 1H), 2.57 – 2.44 (m, 2H), 2.32 – 2.03 (m, 2H); **^{13}C NMR** (101 MHz,

CDCl3) δ 162.7 (d, *J* = 248.4 Hz), 159.3 (d, *J* = 266.1 Hz), 135.5 (dd, *J* = 20.2, 3.3 Hz), 133.4 (d, *J* = 2.7 Hz), 128.4, 128.3 (d, *J* = 7.4 Hz), 127.4 (t, *J* = 7.5 Hz), 126.9, 115.5 (d, *J* = 21.5 Hz), 106.8 (d, *J* = 8.4 Hz), 92.7 (d, *J* = 171.8 Hz), 33.9 (d, *J* = 24.4 Hz), 28.9 (dd, *J* = 27.2, 3.6 Hz); **^{19}F NMR** (376 MHz, CDCl3) δ -102.33 (dt, *J* = 39.0, 19.4 Hz), -113.25 (tq, *J* = 8.4, 4.2 Hz), -174.75 (ddd, *J* = 46.4, 29.9, 15.9 Hz). **HRMS** (EI, m/z): calcd for $C_{17}H_{15}F_3$ $[M]^{+\bullet}$ 276.1120, found 276.1119.

(Z)-1-chloro-4-(1,4-difluoro-5-phenylpent-4-en-1-yl)benzene (3c)

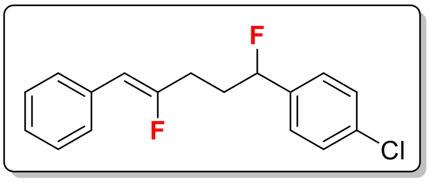

Following the general procedure (eluting with PE/DCM = 20/1). Isolated yield = 64% (18.7 mg); Colorless oil; Rf = 0.2 (PE); **^{1}H NMR** (400 MHz, CDCl3) δ 7.46 (dt, *J* = 7.3, 1.7 Hz, 2H), 7.39 – 7.25 (m, 6H), 7.24 – 7.17 (m, 1H), 5.52 (d, *J* = 39.4 Hz, 1H), 5.50 (ddd, *J* = 47.6, 8.6, 4.5 Hz, 1H), 2.57 – 2.42 (m, 2H), 2.29 – 2.02 (m, 2H); **^{13}C NMR** (101 MHz, CDCl3) δ 159.2 (d, *J* = 266.0 Hz), 138.2 (d, *J* = 20.1 Hz), 134.2 (d, *J* = 2.3 Hz), 133.4 (d, *J* = 2.5 Hz), 128.8, 128.4, 128.3 (d, *J* = 7.4 Hz), 126.9 (d, *J* = 2.4 Hz), 126.8 (d, *J* = 6.9 Hz), 106.8 (d, *J* = 8.4 Hz), 92.5 (d, *J* = 172.5 Hz), 33.8 (d, *J* = 24.1 Hz), 28.8 (dd, *J* = 27.4, 4.3 Hz); **^{19}F NMR** (376 MHz, CDCl3) δ -102.40 (dt, *J* = 36.6, 18.5 Hz), -177.40 (ddd, *J* = 47.6, 30.2, 17.2 Hz). **HRMS** (EI, m/z): calcd for $C_{17}H_{15}ClF_2$ $[M]^{+\bullet}$ 292.0825, found 292.0830.

methyl (Z)-4-(1,4-difluoro-5-phenylpent-4-en-1-yl)benzoate (3d)

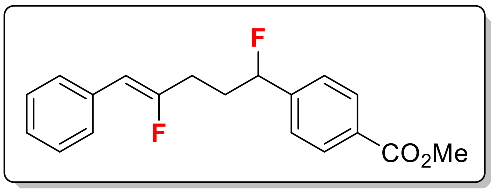

Following the general procedure (eluting with PE/EtOAc = 20/1). Isolated yield = 52% (16.5 mg); Pale yellow oil; Rf = 0.3 (PE:EA = 20:1); **^{1}H NMR** (400 MHz, CDCl3) δ 8.06 (d, *J* = 7.9 Hz, 1H), 7.48 – 7.44 (m, 2H), 7.41 (d, *J* = 8.1 Hz, 2H), 7.32 (t, *J* = 7.7 Hz, 2H), 7.22 (tt, *J* = 6.6, 1.3 Hz, 1H), 5.60 (ddd, *J* = 47.8, 7.8, 4.9 Hz, 1H), 5.53 (d, *J* = 39.4 Hz, 1H), 3.92 (s, 3H), 2.52 (dt, *J* = 18.6, 7.5 Hz, 2H), 2.27 – 2.07 (m, 2H); **^{13}C NMR** (101 MHz, CDCl3) δ 166.6, 159.2 (d, *J* = 266.1 Hz), 144.7 (d, *J* = 19.8 Hz), 133.4, 130.1, 129.9, 128.4, 128.3 (d, *J* = 7.2 Hz), 126.9, 125.2 (d, *J* = 7.4 Hz), 106.9 (d, *J* = 8.5 Hz), 92.6 (d, *J* = 173.5 Hz), 52.2, 33.9 (d, *J* = 23.6 Hz), 28.8 (dd, *J* = 27.0, 4.0 Hz); **^{19}F NMR** (376 MHz, CDCl3) δ -102.53 (dt, *J* = 37.7, 17.8 Hz), -181.00 (ddd, *J* = 48.0, 29.0, 19.1 Hz). **HRMS** (ESI, m/z): calcd for $C_{19}H_{18}F_2O_2$ $[M+H]^+$ 317.1348, found 317.1351.

(Z)-(2,5-difluorohex-1-ene-1,5-diyl)dibenzene (3e)

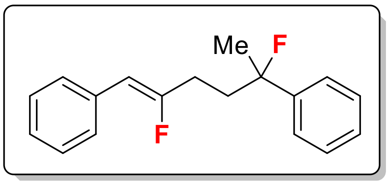

Following the general procedure (except the reaction was carried out at 40 °C; eluting with PE/DCM = 20/1). Isolated yield = 71% (19.3 mg); Colorless oil; Rf = 0.2 (PE); **^{1}H NMR** (400 MHz, $CDCl_3$) δ 7.47 – 7.32 (m, 6H), 7.29 (t, *J* = 7.6 Hz, 3H), 7.18 (tt, *J* = 6.6, 1.2 Hz, 1H), 5.40 (d, *J* = 39.4 Hz, 1H), 2.52 – 2.35 (m, 1H), 2.32 – 2.24 (m, 1H), 2.23 – 2.07 (m, 2H), 1.70 (d, *J* = 22.4 Hz, 3H); **^{13}C NMR** (101 MHz, $CDCl_3$) δ 160.3 (d, *J* = 266.2 Hz), 143.8 (d, *J* = 22.1 Hz), 133.6 (d, *J* = 2.5 Hz), 128.39 (d, *J* = 1.8 Hz), 128.36, 128.2 (d, *J* = 7.3 Hz), 127.3, 126.7 (d, *J* = 2.3 Hz), 124.0 (d, *J* = 10.0 Hz), 105.9 (d, *J* = 8.7 Hz), 97.0 (d, *J* = 174.0 Hz), 38.6 (d, *J* = 23.7 Hz), 28.1 (d, *J* = 25.3 Hz), 27.7 (dd, *J* = 27.1, 4.5 Hz); **^{19}F NMR** (376 MHz, $CDCl_3$) δ -101.27 (dt, *J* = 39.1, 17.3 Hz), -151.09 (dqd, *J* = 28.1, 23.7, 22.8, 17.4 Hz). **HRMS** (EI, m/z): calcd for $C_{18}H_{18}F_2$ $[M]^{+\bullet}$ 272.1371, found 272.1377.

(Z)-(2,5-difluorooct-1-ene-1,5-diyl)dibenzene (3f)

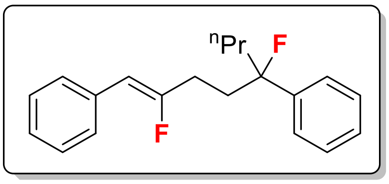

Following the general procedure (eluting with PE/DCM = 20/1). Isolated yield = 50% (15.1 mg); Colorless oil; Rf = 0.2 (PE); **^{1}H NMR** (400 MHz, $CDCl_3$) δ 7.44 – 7.34 (m, 4H), 7.28 (ddd, *J* = 7.8, 4.9, 2.5 Hz, 5H), 7.18 (tt, *J* = 6.7, 1.3 Hz, 1H), 5.38 (d, *J* = 39.4 Hz, 1H), 2.47 – 2.24 (m, 2H), 2.24 – 2.00 (m, 2H), 2.00 – 1.78 (m, 2H), 1.46 – 1.32 (m, 1H), 1.19 – 1.03 (m, 1H), 0.85 (t, *J* = 7.4 Hz, 3H); **^{13}C NMR** (101 MHz, $CDCl_3$) δ 160.5 (d, *J* = 266.3 Hz), 142.3 (d, *J* = 22.2 Hz), 133.7, 128.3, 128.3 (d, *J* = 1.8 Hz), 128.2 (d, *J* = 7.3 Hz), 127.1, 126.7, 124.4 (d, *J* = 10.5 Hz), 105.8 (d, *J* = 8.7 Hz), 99.3 (d, *J* = 177.1 Hz), 43.5 (d, *J* = 23.3 Hz), 37.4 (d, *J* = 23.2 Hz), 27.4 (dd, *J* = 27.2, 4.3 Hz), 16.5 (d, *J* = 3.9 Hz), 14.2; **^{19}F NMR** (376 MHz, $CDCl_3$) δ -101.24 (dt, *J* = 39.2, 17.5 Hz), -162.37 (tt, *J* = 30.6, 14.4 Hz). **HRMS** (EI, m/z): calcd for $C_{20}H_{22}F_2$ $[M]^{+\bullet}$ 300.1684, found 300.1691.

(Z)-(2,5-difluorohex-5-ene-1,2,6-triyl)tribenzene (3g)

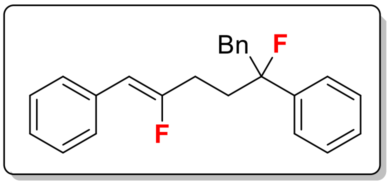

Following the general procedure (eluting with PE/DCM = 20/1). Isolated yield = 69% (24.1 mg); White solid; m.p.: 86.6-88.0 °C; Rf = 0.15 (PE); **^{1}H NMR** (400 MHz, $CDCl_3$) δ 7.44 – 7.14 (m, 13H), 7.02 (dd, *J* = 6.1, 2.6 Hz, 2H), 5.34 (d, *J* = 39.4 Hz, 1H), 3.20 (d, *J* = 23.0 Hz, 2H), 2.47 – 2.17 (m,

3H), 2.07 (dddd, J = 15.3, 13.7, 8.3, 4.6 Hz, 1H); **^{13}C NMR** (101 MHz, $CDCl_3$) δ 160.3 (d, J = 266.1 Hz), 141.6 (d, J = 22.1 Hz), 135.3 (d, J = 2.4 Hz), 133.6 (d, J = 2.5 Hz), 130.6, 128.3, 128.23, 128.19 (d, J = 5.2 Hz), 127.9, 127.3, 126.7 (d, J = 2.3 Hz), 126.6, 124.6 (d, J = 10.4 Hz), 105.9 (d, J = 8.6 Hz), 98.7 (d, J = 180.0 Hz), 48.3 (d, J = 23.4 Hz), 36.0 (d, J = 22.8 Hz), 27.5 (dd, J = 27.0, 4.0 Hz); **^{19}F NMR** (376 MHz, $CDCl_3$) δ -101.48 (dt, J = 37.1, 17.7 Hz), -160.50 (dt, J = 44.9, 23.3 Hz). **HRMS** (EI, m/z): calcd for $C_{22}H_{24}F_2$ $[M]^{+\bullet}$ 348.1684, found 348.1692.

(Z)-(2,5,6-trifluorohex-1-ene-1,5-diyl)dibenzene (3h)

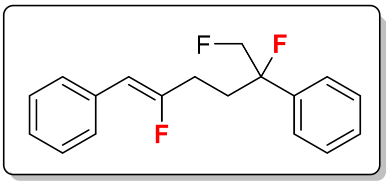

Following the general procedure (eluting with PE/DCM = 20/1). Isolated yield = 63% (18.3 mg); Colorless oil; Rf = 0.1 (PE); **^{1}H NMR** (400 MHz, $CDCl_3$) δ 7.45 – 7.34 (m, 7H), 7.29 (t, J = 7.7 Hz, 2H), 7.19 (tt, J = 6.7, 1.4 Hz, 1H), 5.41 (d, J = 39.3 Hz, 1H), 4.74 – 4.36 (m, 2H), 2.63 – 2.35 (m, 2H), 2.33 – 2.06 (m, 2H); **^{13}C NMR** (101 MHz, $CDCl_3$) δ 159.7 (d, J = 265.9 Hz), 137.4 (dd, J = 21.4, 4.4 Hz), 133.5 (d, J = 2.6 Hz), 128.7 (d, J = 1.7 Hz), 128.39, 128.37, 128.2 (d, J = 7.3 Hz), 126.8 (d, J = 2.2 Hz), 124.8 (d, J = 10.1 Hz), 106.2 (d, J = 8.6 Hz), 97.4 (dd, J = 180.4, 18.1 Hz), 86.6 (dd, J = 183.6, 26.3 Hz), 31.8 (dd, J = 22.7, 3.4 Hz), 26.8 (dd, J = 27.4, 4.0 Hz); **^{19}F NMR** (376 MHz, $CDCl_3$) δ -101.88 (dt, J = 39.1, 17.3 Hz), -169.14 (dddt, J = 45.5, 25.9, 19.3, 8.8 Hz), -224.79 (td, J = 47.6, 14.9 Hz). **HRMS** (EI, m/z): calcd for $C_{18}H_{17}F_3$ $[M]^{+\bullet}$ 290.1277, found 290.1284.

(Z)-1-(2,5-difluoro-6-phenylhex-5-en-2-yl)-4-fluorobenzene (3i)

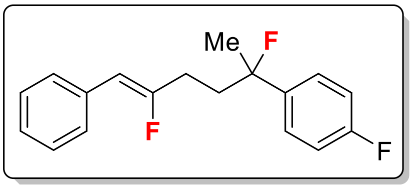

Following the general procedure (except the reaction was carried out at 40 °C; eluting with PE/DCM = 20/1). Isolated yield = 69% (20.1 mg); Colorless oil; Rf = 0.2 (PE); **^{1}H NMR** (400 MHz, $CDCl_3$) δ 7.41 (d, J = 7.4 Hz, 2H), 7.30 (q, J = 7.8, 7.4 Hz, 4H), 7.19 (t, J = 7.4 Hz, 1H), 7.06 (t, J = 8.6 Hz, 2H), 5.40 (d, J = 39.4 Hz, 1H), 2.41 (dddd, J = 18.8, 15.6, 9.9, 3.8 Hz, 1H), 2.29 – 2.08 (m, 3H), 1.69 (d, J = 22.4 Hz, 3H); **^{13}C NMR** (101 MHz, $CDCl_3$) δ 162.0 (d, J = 245.7 Hz), 160.0 (d, J = 265.9 Hz), 139.6 (d, J = 22.7 Hz), 133.5, 128.4, 128.2 (d, J = 7.3 Hz), 126.8 (d, J = 1.7 Hz), 125.8 (dd, J = 9.8, 8.3 Hz), 115.2 (d, J = 21.4 Hz), 106.0 (d, J = 8.6 Hz), 96.8 (d, J = 174.1 Hz), 38.6 (d, J = 23.8 Hz), 28.1 (d, J = 25.1 Hz), 27.7 (dd, J = 27.3, 4.4 Hz); **^{19}F NMR** (376 MHz, $CDCl_3$) δ -101.55 (dt, J = 38.5, 17.2 Hz), -115.29 (p, J = 8.2 Hz), -149.76 (dq, J = 45.0, 22.1 Hz). **HRMS** (EI, m/z): calcd for $C_{18}H_{17}F_3$

[M]$^{+\bullet}$ 290.1277, found 290.1279.

NOTE: In some examples (**3e**, **3i**, and **3l**), the major reaction pathway under the standard conditions (50 °C) is the Heck-type allylation. Specifically, 4-fluoro-α-methylstyrene (**2i**) was subjected at 50 °C to produce the allylation products consisting of five isomers (shown below) in 66% combined yield, which were identified by ^{1}H NMR and ^{19}F NMR analysis. Note that lowering the reaction temperature from 50 °C to 40 °C can make the carbofluorination return predominate (**3i**, 69% yield at 40 °C).

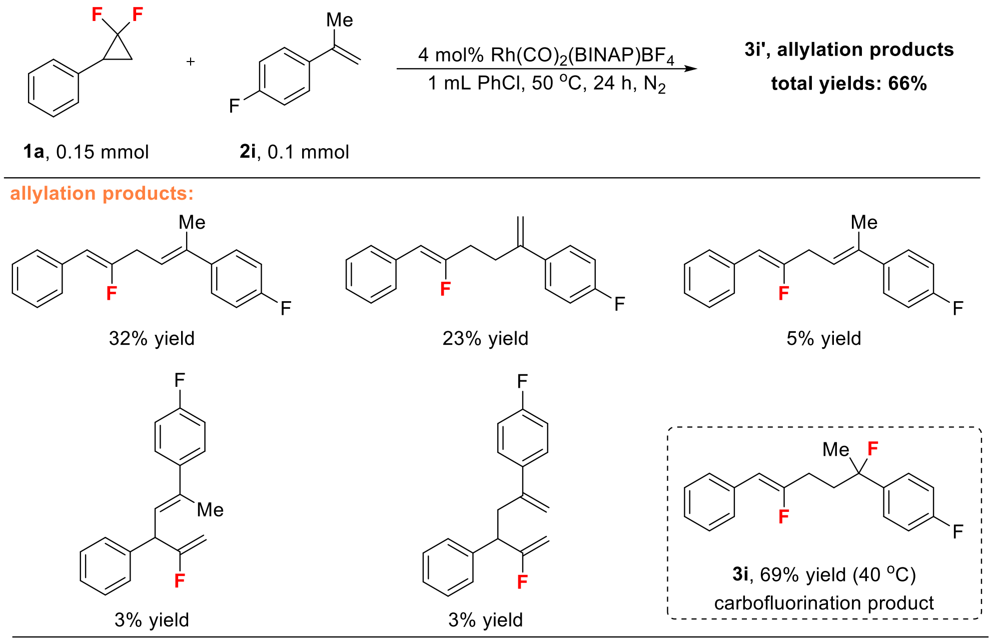

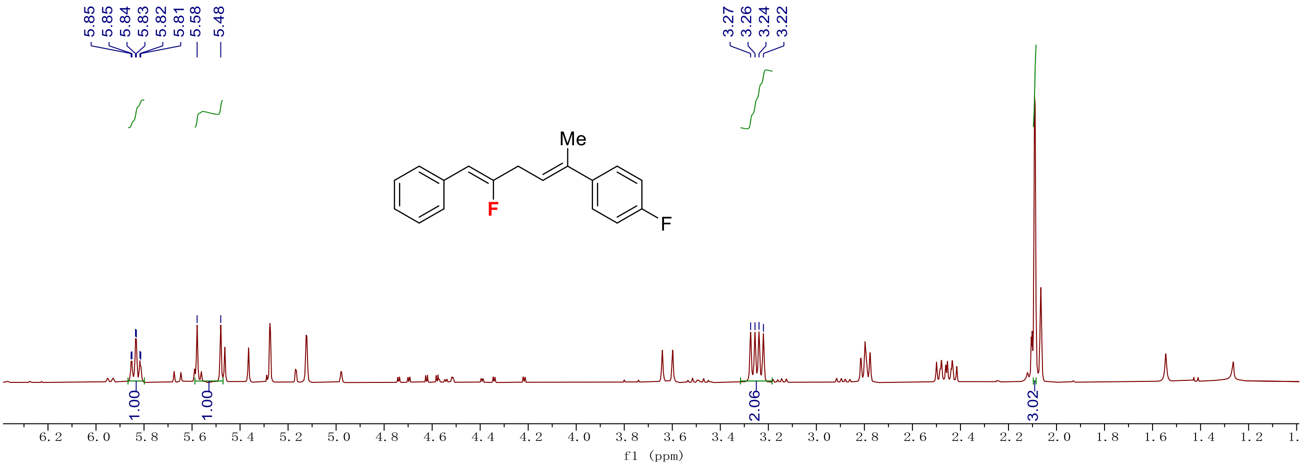

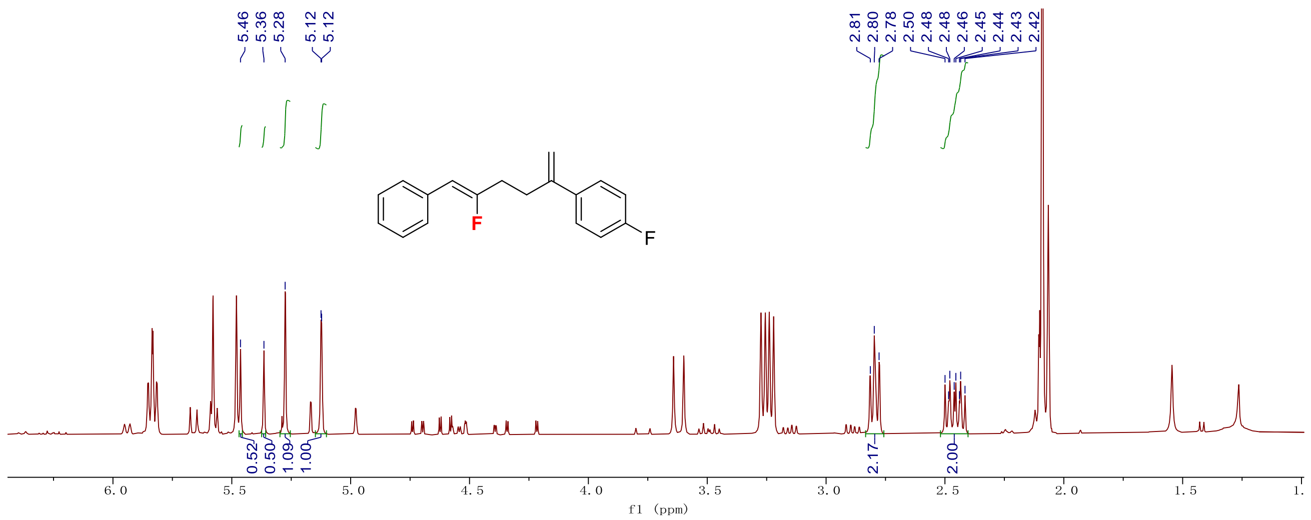

Supplementary Figure 5. The ^{1}H NMR spectrum of the by-products in the reaction of 3i at 50 °C.

(Z)-1-chloro-4-(2,5-difluoro-6-phenylhex-5-en-2-yl)benzene (3j)

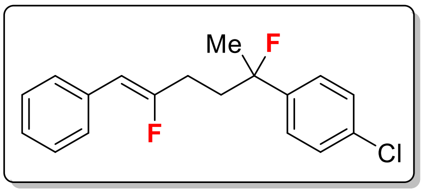

Following the general procedure (eluting with PE/DCM = 20/1). Isolated yield = 83% (25.4 mg); Colorless oil; Rf = 0.2 (PE); **^{1}H NMR** (400 MHz, $CDCl_3$) δ 7.41 (dd, *J* = 8.4, 1.3 Hz, 2H), 7.36 – 7.24 (m, 6H), 7.19 (tt, *J* = 6.7, 1.4 Hz, 1H), 5.39 (d, *J* = 39.4 Hz, 1H), 2.41 (dddd, *J* = 18.8, 15.7, 10.2, 3.9 Hz, 1H), 2.32 – 2.05 (m, 3H), 1.67 (d, *J* = 22.3 Hz, 3H); **^{13}C NMR** (101 MHz, $CDCl_3$) δ 159.9 (d, *J* = 265.9 Hz), 142.3 (d, *J* = 22.5 Hz), 133.5 (d, *J* = 2.5 Hz), 133.2, 128.6, 128.4, 128.2 (d, *J* = 7.2 Hz), 126.8 (d, *J* = 2.4 Hz), 125.5 (d, *J* = 10.0 Hz), 106.1 (d, *J* = 8.7 Hz), 96.7 (d, *J* = 174.5 Hz), 38.5 (d, *J* = 23.7 Hz), 28.0 (d, *J* = 25.3 Hz), 27.6 (dd, *J* = 27.2, 4.4 Hz); **^{19}F NMR** (376 MHz, $CDCl_3$) δ -101.62 (dt, *J* = 39.4, 17.4 Hz), -150.91 (dq, *J* = 43.3, 23.2, 21.8 Hz). **HRMS** (EI, m/z): calcd for $C_{18}H_{17}ClF_2$ $[M]^{+\bullet}$ 306.0981, found 306.0989.

methyl (Z)-4-(2,5-difluoro-6-phenylhex-5-en-2-yl)benzoate (3k)

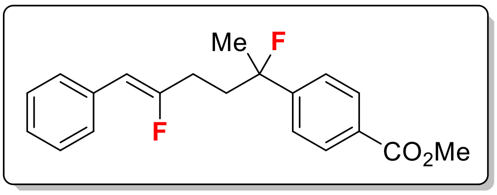

Following the general procedure (eluting with PE/EtOAc = 20/1). Isolated yield = 83% (27.4 mg); Pale yellow oil; Rf = 0.2 (PE:EA = 20:1); **^{1}H NMR** (400 MHz, $CDCl_3$) δ 8.05 (d, *J* = 8.1 Hz, 2H), 7.41 (dd, *J* = 8.8, 7.0 Hz, 4H), 7.28 (t, *J* = 7.7 Hz, 2H), 7.18 (tt, *J* = 6.8, 1.4 Hz, 1H), 5.39 (d, *J* = 39.3 Hz, 1H), 3.92 (s, 3H), 2.42 (dddd, *J* = 18.7, 14.4, 11.1, 5.2 Hz, 1H), 2.32 – 2.03 (m, 3H), 1.71 (d, *J* = 22.4 Hz, 3H); **^{13}C NMR** (101 MHz, $CDCl_3$) δ 166.7, 159.8 (d, *J* = 266.0 Hz), 148.8

(d, J = 22.0 Hz), 133.5 (d, J = 2.5 Hz), 129.8 (d, J = 1.8 Hz), 129.3, 128.3, 128.2 (d, J = 7.3 Hz), 126.8 (d, J = 2.3 Hz), 124.1 (d, J = 10.1 Hz), 106.1 (d, J = 8.6 Hz), 96.9 (d, J = 175.6 Hz), 52.1, 38.4 (d, J = 23.6 Hz), 27.9 (d, J = 25.2 Hz), 27.6 (dd, J = 27.2, 4.4 Hz); **^{19}F NMR** (376 MHz, $CDCl_3$) δ -101.76 (dt, J = 38.9, 17.4 Hz), -151.89 (dqd, J = 28.0, 22.0, 16.5 Hz). **HRMS** (ESI, m/z): calcd for $C_{20}H_{20}F_2O_{20}$ $[M+H]^+$ 331.1504, found 331.1506.

(Z)-4-(2,5-difluoro-6-phenylhex-5-en-2-yl)phenyl 4-methylbenzenesulfonate (3l)

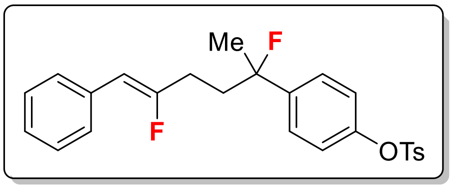

Following the general procedure (eluting with PE/EtOAc = 10/1). Isolated yield = 90% (39.7 mg); White solid; m.p.: 102.8-105.0 °C; Rf = 0.2 (PE:EA = 20:1); **^{1}H NMR** (400 MHz, $CDCl_3$) δ 7.71 (dt, J = 8.6, 1.8 Hz, 2H), 7.41 (dd, J = 8.3, 1.3 Hz, 2H), 7.34 – 7.24 (m, 6H), 7.19 (tt, J = 6.7, 1.3 Hz, 1H), 6.99 (d, J = 8.6 Hz, 2H), 5.40 (d, J = 39.4 Hz, 1H), 2.44 (s, 3H), 2.42 – 2.31 (m, 1H), 2.26 – 2.02 (m, 3H), 1.66 (d, J = 22.4 Hz, 3H); **^{13}C NMR** (101 MHz, $CDCl_3$) δ 159.9 (d, J = 265.9 Hz), 148.7, 145.4, 142.8 (d, J = 22.5 Hz), 133.4 (d, J = 2.4 Hz), 132.3, 129.7, 128.44, 128.37, 128.2 (d, J = 7.3 Hz), 126.8 (d, J = 2.3 Hz), 125.4 (d, J = 10.0 Hz), 122.3, 106.0 (d, J = 8.6 Hz), 96.6 (d, J = 175.0 Hz), 38.5 (d, J = 23.7 Hz), 27.8 (d, J = 24.8 Hz), 27.6 (dd, J = 26.3, 5.3 Hz), 21.7; **^{19}F NMR** (376 MHz, $CDCl_3$) δ -101.56 (dt, J = 41.0, 17.8 Hz), -150.48 (dqd, J = 26.8, 22.8, 22.0, 16.5 Hz). **HRMS** (ESI, m/z): calcd for $C_{25}H_{24}F_2O_3S$ $[M+Na]^+$ 465.1306, found 465.1310.

(Z)-(2,5-difluoro-4-methylpent-1-ene-1,5-diyl)dibenzene (3m)

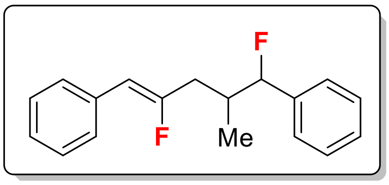

Following the general procedure (eluting with PE/DCM = 20/1). Isolated yield = 92% (24.9 mg); Colorless oil; Rf = 0.2 (PE); The reaction was analyzed by ^{19}F NMR of the crude product giving 1.3:1 dr. **^{1}H NMR** (400 MHz, $CDCl_3$) δ 7.49 – 7.45 (m, 2H), 7.41 – 7.34 (m, 2H), 7.34 – 7.28 (m, 5H), 7.24 – 7.18 (m, 1H), 5.52 (d, J = 39.3 Hz, 0.4 H), 5.51 (d, J = 39.2 Hz, 0.6 H), 5.48 (dd, J = 47.0, 4.3 Hz, 0.4 H), 5.25 (dd, J = 46.9, 7.1 Hz, 0.6 H), 2.71 (td, J = 13.9, 3.8 Hz, 0.6 H), 2.53 (ddd, J = 19.7, 14.2, 5.9 Hz, 0.4 H), 2.46 – 2.30 (m, 1H), 2.29 – 2.12 (m, 1H), 0.98 (d, J = 6.7 Hz, 1.2 H), 0.93 (d, J = 6.9 Hz, 1.8 H); **^{13}C NMR** (101 MHz, $CDCl_3$) δ 159.1 (d, J = 266.8 Hz), 158.7 (d, J = 266.1 Hz), 138.9 (d, J = 20.4 Hz), 138.5 (d, J = 20.2 Hz), 133.6 (d, J = 2.7 Hz), 133.5 (d, J = 2.7 Hz), 128.39, 128.38, 128.31, 128.28, 128.2, 128.0, 126.9 (d, J = 2.1 Hz), 126.8 (d, J = 2.2 Hz), 126.1 (d, J = 7.1 Hz), 125.5

(d, J = 8.2 Hz), 108.0 (d, J = 8.6 Hz), 107.9 (d, J = 8.7 Hz), 97.7 (d, J = 174.8 Hz), 37.0 (d, J = 20.9 Hz), 36.8 (d, J = 22.0 Hz), 36.8 (dd, J = 26.5, 4.5 Hz), 35.3 (dd, J = 26.2, 5.1 Hz), 15.1 (d, J = 5.2 Hz), 13.1 (d, J = 5.3 Hz); **^{19}F NMR** (376 MHz, $CDCl_3$) δ -100.45 (ddd, J = 39.8, 28.1, 13.3 Hz, 0.6 F), -102.02 (dt, J = 39.2, 20.6 Hz, 0.4 F), -179.52 (dd, J = 46.7, 16.5 Hz, 0.6 F), -191.81 (dd, J = 46.9, 24.8 Hz, 0.4 F). **HRMS** (EI, m/z): calcd for $C_{18}H_{18}F_2$ $[M]^{+\bullet}$ 272.1371, found 272.1375.

Note that: In examples **3m-3u**, the range of dr is 1.2:1-1.5:1, as determined by ^{19}F NMR analysis of the crude reaction mixture. The two diastereoisomers were difficult to separate by column chromatography, so they were collected together. The characterization data will be described as follows. First, the total amounts of the two diastereoisomers will be normalized to **1** in the ^{19}F NMR spectrum and their respective contents are calculated. Secondly, in the ^{1}H NMR spectrum, the integration values and coupling pattern of characteristic hydrogen signals will be indicated as much as possible based on the quantized data obtained from the ^{19}F NMR analysis. The overlapping signals will be integrated together and written as multiple peaks (m). Finally, for the characterization of ^{13}C NMR spectrum, the coupling pattern and chemical shifts of most signals can be characterized based on the coupling principle between carbon and fluorine. However, many signals of aromatic carbon overlap and cannot be determined where these peaks would only be considered as a single peak and written as "s".

(Z)-(2,5-difluoro-4-propylpent-1-ene-1,5-diyl)dibenzene (3n)

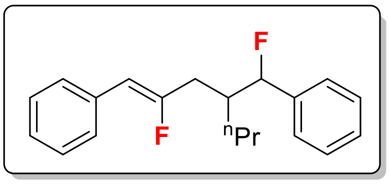

Following the general procedure (eluting with PE/DCM = 20/1). Isolated yield = 88% (26.3 mg); Colorless oil; Rf = 0.2 (PE); The reaction was analyzed by ^{19}F NMR of the crude product giving 1.5:1 dr. **^{1}H NMR** (400 MHz, $CDCl_3$) δ 7.52 – 7.42 (m, 2H), 7.41 – 7.34 (m, 2H), 7.34 – 7.26 (m, 5H), 7.26 – 7.15 (m, 1H), 5.59 (dd, J = 46.9, 4.5 Hz, 0.4 H), 5.51 (d, J = 39.5 Hz, 0.4 H), 5.50 (dd, J = 46.9, 5.2 Hz, 0.6 H), 5.47 (d, J = 39.3 Hz, 0.6 H), 2.59 – 2.16 (m, 3H), 1.49 – 1.19 (m, 4H), 0.93 – 0.81 (m, 3H); **^{13}C NMR** (101 MHz, $CDCl_3$) δ 159.3 (d, J = 266.7 Hz), 158.9 (d, J = 266.2 Hz), 138.9 (d, J = 20.4 Hz), 133.7 (d, J = 2.5 Hz), 133.5 (d, J = 2.5 Hz), 128.41, 128.36, 128.3, 128.27, 128.2, 128.1, 127.9, 126.9 (d, J = 2.3 Hz), 126.7 (d, J = 2.1 Hz), 125.7 (d, J = 7.9 Hz), 125.5 (d, J = 8.2 Hz), 108.1 (d, J = 8.6 Hz), 107.8 (d, J = 8.7 Hz), 95.1 (d, J = 175.2 Hz), 95.2 (d, J = 175.2 Hz), 41.5 (d, J = 21.1 Hz), 41.3 (d, J = 21.9 Hz), 34.1 (dd, J = 26.3, 4.1 Hz), 32.4 (dd, J = 26.2, 5.5 Hz), 31.4 (d, J = 3.8 Hz), 29.9

(d, J = 4.1 Hz), 20.1, 19.9, 14.1, 14.1; **^{19}F NMR** (376 MHz, $CDCl_3$) δ -100.66 (ddd, J = 39.2, 27.0, 13.0 Hz, 0.6F), -101.67 (ddd, J = 40.0, 23.9, 18.1 Hz, 0.4F), -187.39 (dd, J = 47.0, 22.4 Hz, 0.6F), -190.18 (dd, J = 46.8, 24.7 Hz, 0.4F). **HRMS** (EI, m/z): calcd for $C_{20}H_{22}F_2$ $[M]^{+\bullet}$ 300.1684, found 300.1692.

(Z)-(4-benzyl-2,5-difluoropent-1-ene-1,5-diyl)dibenzene (3o)

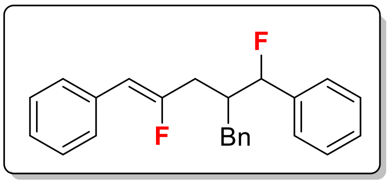

Following the general procedure (eluting with PE/DCM = 20/1). Isolated yield = 76% (26.3 mg); Colorless oil; Rf = 0.15 (PE); The reaction was analyzed by ^{19}F NMR of the crude product giving 1.5:1 dr. **^{1}H NMR** (400 MHz, $CDCl_3$) δ 7.47 – 7.41 (m, 2H), 7.39 – 7.17 (m, 12H), 7.13 – 7.10 (m, 1H), 5.67 (dd, J = 46.6, 3.8 Hz, 0.37H), 5.49 (dd, J = 46.6, 3.6 Hz, 0.63H), 5.46 (d, J = 39.3 Hz, 1H), 2.91 – 2.81 (m, 1H), 2.78 – 2.23 (m, 4H); **^{13}C NMR** (101 MHz, $CDCl_3$) δ 158.8 (d, J = 266.4 Hz), 158.4 (d, J = 266.1 Hz), 139.5, 139.4, 138.7 (d, J = 20.4 Hz), 138.7 (d, J = 20.4 Hz), 133.5 (d, J = 2.4 Hz), 133.4 (d, J = 2.6 Hz), 129.3, 129.2, 128.5, 128.4, 128.37, 128.34, 128.28, 128.26, 128.1, 127.9, 126.9 (d, J = 2.3 Hz), 126.8 (d, J = 2.2 Hz), 126.3, 108.6 (d, J = 8.4 Hz), 108.3 (d, J = 8.6 Hz), 94.1 (d, J = 175.7 Hz), 93.3 (d, J = 176.2 Hz), 44.1 (d, J = 21.4 Hz), 43.5 (d, J = 21.7 Hz), 35.8 (d, J = 2.9 Hz), 33.6 (d, J = 4.6 Hz), 33.2 (dd, J = 26.1, 3.7 Hz), 31.8 (dd, J = 26.3, 4.7 Hz); **^{19}F NMR** (376 MHz, $CDCl_3$) δ -101.61 (ddd, J = 40.9, 27.8, 13.5 Hz, 0.63F), -101.87 (ddd, J = 39.6, 25.3, 15.4 Hz, 0.37F), -191.32 (dd, J = 46.6, 24.4 Hz, 0.37F), -195.71 (dd, J = 46.5, 26.7 Hz, 0.63F). **HRMS** (EI, m/z): calcd for $C_{24}H_{22}F_2$ $[M]^{+\bullet}$ 348.1684, found 348.1688.

(Z)-(2,5-difluoro-4-(2-fluoroethyl)pent-1-ene-1,5-diyl)dibenzene (3p)

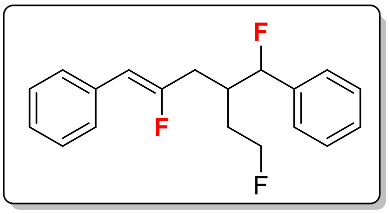

Following the general procedure (eluting with PE/DCM = 20/1). Isolated yield = 67% (20.3 mg); Colorless oil; Rf = 0.5 (PE:EA = 100:1); The reaction was analyzed by ^{19}F NMR of the crude product giving 1.2:1 dr. **^{1}H NMR** (400 MHz, $CDCl_3$) δ 7.50 – 7.29 (m, 9H), 7.25 – 7.16 (m, 1H), 5.63 (dd, J = 46.8, 4.1 Hz, 0.47H), 5.62 (dd, J = 46.8, 5.2 Hz, 0.47H), 5.55 (d, J = 39.4 Hz, 0.43H), 5.50 (d, J = 39.0 Hz, 0.53H), 4.66 – 4.27 (m, 2H), 2.67 – 2.27 (m, 3H), 2.08 – 1.69 (m, 2H); **^{13}C NMR** (101 MHz, $CDCl_3$) δ 158.5 (d, J = 266.4 Hz), 158.0 (d, J = 266.1 Hz), 138.4 (d, J = 20.4 Hz), 138.2 (d, J = 20.3 Hz), 133.3 (d, J = 2.6 Hz), 133.2 (d, J = 2.7 Hz), 128.51, 128.47, 128.45,

128.41, 128.35, 128.28, 128.23, 128.22, 108.6 (d, J = 8.4 Hz), 108.4 (d, J = 8.5 Hz), 95.9, 94.1, 82.4 (dd, J = 165.1, 1.8 Hz), 81.9 (d, J = 165.2 Hz), 38.5 (d, J = 22.2 Hz), 38.4 (d, J = 23.8 Hz), 34.4 (dd, J = 26.5, 4.3 Hz), 32.2 (dd, J = 26.2, 5.4 Hz), 30.0 (dd, J = 19.8, 3.8 Hz), 28.8 (dd, J = 20.1, 3.8 Hz); **^{19}F NMR** (376 MHz, $CDCl_3$) δ -100.99 (ddd, J = 40.2, 27.6, 13.3 Hz, 0.53F), -102.08 (ddd, J = 39.8, 23.1, 17.0 Hz,0.46F), -187.25 (dd, J = 46.7, 22.5 Hz, 0.53F), -190.04 (dd, J = 46.7, 24.8 Hz, 0.47F), -217.24 (tt, J = 48.5, 25.7 Hz0.47F), -218.52 (tt, J = 47.6, 26.1 Hz, 0.53F). **HRMS** (EI, m/z): calcd for $C_{19}H_{19}F_3$ $[M]^{+\bullet}$ 304.1433, found 304.1440.

(Z)-(4-(3-chloropropyl)-2,5-difluoropent-1-ene-1,5-diyl)dibenzene (3q)

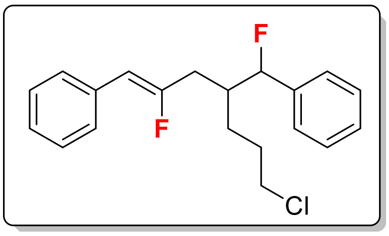

Following the general procedure (eluting with PE/DCM = 20/1). Isolated yield = 68% (22.7 mg); Colorless oil; Rf = 0.5 (PE:EA = 100:1); The reaction was analyzed by ^{19}F NMR of the crude product giving 1.2:1 dr. **^{1}H NMR** (400 MHz, $CDCl_3$) δ 7.52 – 7.42 (m, 2H), 7.41 – 7.36 (m, 2H), 7.35 – 7.27 (m, 5H), 7.25 – 7.16 (m, 1H), 5.57 (dd, J = 46.9, 4.6 Hz, 0.45H), 5.53 (d, J = 39.4 Hz, 0.45H)., 5.49 (dd, J = 46.7, 5.2 Hz, 0.55H), 5.49 (d, J = 39.3 Hz, 0.55H), 3.46 (t, J = 6.6 Hz, 1.1H), 3.44 (td, J = 6.5, 1.3 Hz, 1H), 2.64 – 2.14 (m, 3H), 1.93 – 1.49 (m, 4H); **^{13}C NMR** (101 MHz, $CDCl_3$) δ 158.7 (d, J = 266.2 Hz), 158.2 (d, J = 265.8 Hz), 138.6 (d, J = 20.3 Hz), 138.4 (d, J = 20.4 Hz), 133.4 (d, J = 2.2 Hz), 133.3 (d, J = 2.5 Hz), 128.5, 128.43, 128.40, 128.34, 128.31, 128.26, 128.16, 127.0 (d, J = 2.0 Hz), 126.9 (d, J = 2.3 Hz), 125.7 (d, J = 7.8 Hz), 125.5 (d, J = 7.9 Hz), 108.4 (d, J = 8.6 Hz), 108.2 (d, J = 8.6 Hz), 95.2 (d, J = 175.8 Hz), 95.1 (d, J = 175.8 Hz), 44.9, 44.8, 41.3 (d, J = 22.2 Hz), 41.1 (d, J = 23.1 Hz), 34.3 (dd, J = 26.5, 4.4 Hz), 32.4 (dd, J = 26.3, 5.5 Hz), 30.1, 29.9, 26.9 (d, J = 3.7 Hz), 25.5 (d, J = 3.7 Hz); **^{19}F NMR** (376 MHz, $CDCl_3$) δ -101.12 (ddd, J = 39.5, 27.1, 13.2 Hz, 0.55F), -102.22 (dt, J = 40.8, 20.5 Hz, 0.45F), -186.20 (dd, J = 47.1, 21.6 Hz, 0.55F), -189.31 (dd, J = 47.3, 23.9 Hz, 0.45F). **HRMS** (EI, m/z): calcd for $C_{20}H_{21}ClF_2$ $[M]^{+\bullet}$ 334.1294, found 334.1298.

ethyl (Z)-7-fluoro-5-(fluoro(phenyl)methyl)-8-phenyloct-7-enoate (3r)

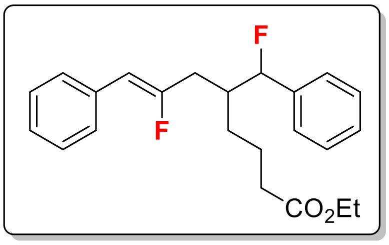

Following the general procedure (eluting with PE/EtOAc = 20/1). Isolated yield = 75% (27.8 mg); Colorless oil; Rf = 0.3 (PE:EA = 20:1); The reaction was analyzed by ^{19}F NMR of the crude product giving 1.2:1 dr. **^{1}H NMR** (400 MHz, $CDCl_3$) δ 7.51 – 7.43 (m, 2H), 7.42 – 7.36 (m, 2H), 7.35 – 7.28 (m, 5H), 7.24 – 7.18 (m, 1H), 5.58 (dd, *J* = 46.8, 4.7 Hz, 0.4H), 5.55 (dd, *J* = 46.9, 5.4 Hz, 0.6H), 5.54 (d, *J* = 39.4 Hz, 0.4H), 5.50 (d, *J* = 39.2 Hz, 0.6H), 4.08 (q, *J* = 7.1 Hz, 1.2H), 4.07 (q, *J* = 7.2 Hz, 0.8H), 2.61 – 2.12 (m, 5H), 1.84 – 1.39 (m, 4H), 1.20 (t, *J* = 7.2 Hz, 1.2H), 1.20 (t, *J* = 7.2 Hz, 1.8H); **^{13}C NMR** (101 MHz, $CDCl_3$) δ 173.29, 173.25, 158.9 (d, *J* = 266.6 Hz), 158.4 (d, *J* = 266.0 Hz), 138.7 (d, *J* = 20.4 Hz), 138.6 (d, *J* = 20.3 Hz), 133.5 (d, *J* = 2.5 Hz), 133.4 (d, *J* = 2.5 Hz), 128.4, 128.38, 128.34, 128.32, 128.3, 128.27, 128.22, 128.19 (d, *J* = 1.7 Hz), 128.0 (d, *J* = 1.7 Hz), 126.9 – 126.9 (m), 126.8 (d, *J* = 2.3 Hz), 125.6 (d, *J* = 7.9 Hz), 125.5 (d, *J* = 8.0 Hz), 108.3 (d, *J* = 8.4 Hz), 108.0 (d, *J* = 8.6 Hz), 94.9 (d, *J* = 175.6 Hz), 94.9 (d, *J* = 175.5 Hz), 60.2, 60.2, 41.6 (d, *J* = 21.6 Hz), 41.3 (d, *J* = 22.2 Hz), 34.3, 34.3, 33.9 (dd, *J* = 26.2, 4.0 Hz), 32.2 (dd, *J* = 26.2, 5.5 Hz), 28.7, 27.3 (d, *J* = 4.0 Hz), 22.3, 22.2, 14.1, 14.1; **^{19}F NMR** (376 MHz, $CDCl_3$) δ -100.92 (ddd, *J* = 39.8, 27.0, 13.4 Hz, 0.6F), -101.95 (ddd, *J* = 41.0, 23.9, 18.5 Hz, 0.4F), -187.15 (dd, *J* = 47.1, 22.0 Hz, 0.6F), -189.75 (dd, *J* = 46.2, 24.1 Hz, 0.4F). **HRMS** (ESI, m/z): calcd for $C_{23}H_{26}F_2O_2$ $[M+Na]^+$ 395.1793, found 395.1797.

(Z)-(4-(4-(benzyloxy)butyl)-2,5-difluoropent-1-ene-1,5-diyl)dibenzene (3s)

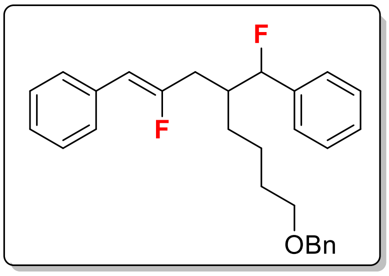

Following the general procedure (eluting with PE/DCM = 10/1). Isolated yield = 70% (29.4 mg); Pale yellow oil; Rf = 0.4 (PE:EA = 100:1); The reaction was analyzed by ^{19}F NMR of the crude product giving 1.3:1 dr. **^{1}H NMR** (400 MHz, $CDCl_3$) δ 7.48 – 7.40 (m, 2H), 7.37 – 7.24 (m, 12H), 7.22 – 7.13 (m, 1H), 5.58 (dd, *J* = 46.9, 4.5 Hz, 0.4H), 5.49 (dd, *J* = 46.8, 5.2 Hz, 0.6H), 5.49 (d, *J* = 39.0 Hz, 0.4H), 5.45 (d, *J* = 39.3 Hz, 0.6H), 4.43 (s, 1.2H), 4.42 (s, 0.8H), 3.40 (t, *J* = 6.3 Hz, 1.2H), 3.37 (t, *J* = 6.2 Hz, 0.8H), 2.58 – 2.15 (m, 3H), 1.62 – 1.38 (m, 6H); **^{13}C NMR** (101 MHz, $CDCl_3$) δ 159.1 (d, *J* = 266.5 Hz), 158.7 (d, *J* = 266.0 Hz), 138.84 (d, *J* = 20.6 Hz), 138.82 (d, *J* = 20.6 Hz), 138.51, 138.5, 133.6 (d, *J* = 2.4 Hz), 133.4 (d, *J* = 2.4 Hz), 128.4, 128.33, 128.28,

128.25, 128.1 (d, J = 1.6 Hz), 127.9 (d, J = 1.5 Hz), 127.5, 127.4, 126.8 (d, J = 1.9 Hz), 126.7 (d, J = 2.2 Hz), 125.6 (d, J = 7.8 Hz), 125.5 (d, J = 8.1 Hz), 108.1 (d, J = 8.5 Hz), 107.9 (d, J = 8.7 Hz), 95.0 (d, J = 175.3 Hz), 94.9 (d, J = 175.3 Hz), 72.8, 70.0, 41.7 (d, J = 21.3 Hz), 41.4 (d, J = 21.6 Hz), 34.0 (dd, J = 26.1, 4.2 Hz), 32.3 (dd, J = 26.1, 5.4 Hz), 29.7, 29.0 (d, J = 3.8 Hz), 27.4 (d, J = 4.3 Hz), 23.4, 23.3; **^{19}F NMR** (376 MHz, $CDCl_3$) δ -100.55 (ddd, J = 39.9, 27.3, 13.6 Hz, 0.6F), -101.59 (ddd, J = 41.3, 23.8, 17.8 Hz, 0.4F), -187.20 (dd, J = 46.8, 22.4 Hz, 0.6F), -190.06 (dd, J = 46.7, 24.6 Hz, 0.4F). **HRMS** (ESI, m/z): calcd for $C_{28}H_{30}F_2O$ $[M+Na]^+$ 443.2157, found 443.2161.

(Z)-7-fluoro-5-(fluoro(phenyl)methyl)-8-phenyloct-7-en-1-yl pivalate (3t)

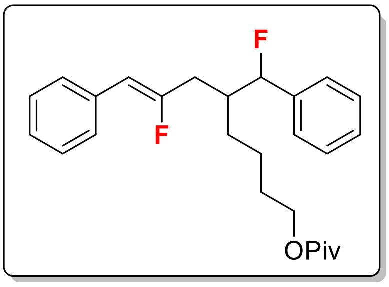

Following the general procedure (eluting with PE/EtOAc = 20/1). Isolated yield = 69% (28.4 mg); Pale yellow oil; Rf = 0.2 (PE:EA = 20:1); The reaction was analyzed by ^{19}F NMR of the crude product giving 1.4:1 dr. **^{1}H NMR** (400 MHz, $CDCl_3$) δ 7.49 – 7.42 (m, 2H), 7.40 – 7.35 (m, 2H), 7.34 – 7.26 (m, 5H), 7.24 – 7.15 (m, 1H), 5.60 (dd, J = 47.5, 4.6 Hz, 0.42H), 5.60 (dd, J = 46.1, 4.3 Hz, 0.58H), 5.51 (d, J = 40.0 Hz, 0.42H), 5.48 (d, J = 39.4 Hz, 0.58H), 4.00 (t, J = 6.4 Hz, 1.16H), 3.98 (t, J = 6.4 Hz, 0.84H), 2.73 – 2.15 (m, 2H), 1.62 – 1.39 (m, 6H), 1.30 (ddd, J = 19.9, 9.4, 5.0 Hz, 1H), 1.17 (s, 9H); **^{13}C NMR** (101 MHz, $CDCl_3$) δ 178.5, 159.0 (d, J = 266.6 Hz), 158.6 (d, J = 266.3 Hz), 138.8 (d, J = 20.4 Hz), 138.7 (d, J = 20.4 Hz), 133.5 (d, J = 2.4 Hz), 133.4 (d, J = 2.8 Hz), 128.44, 128.40, 128.37, 128.31, 128.25, 128.18, 128.0, 128.0, 126.9 (d, J = 2.1 Hz), 126.8 (d, J = 2.1 Hz), 125.7 (d, J = 7.9 Hz), 125.4, 108.2 (d, J = 8.6 Hz), 108.0 (d, J = 8.8 Hz), 95.1 (d, J = 175.4 Hz), 95.0 (d, J = 175.5 Hz), 64.0, 41.7 (d, J = 21.4 Hz), 41.5 (d, J = 21.8 Hz), 38.7, 34.1 (dd, J = 26.2, 4.2 Hz), 32.4 (dd, J = 26.0, 5.3 Hz), 28.9 (d, J = 3.8 Hz), 28.7, 27.4 (d, J = 4.2 Hz), 27.1, 23.4, 23.2; **^{19}F NMR** (376 MHz, $CDCl_3$) δ -100.81 (ddd, J = 40.9, 28.0, 14.7 Hz, 0.58F), -101.88 (ddd, J = 41.0, 23.5, 18.2 Hz, 0.42F), -186.72 (dd, J = 47.3, 21.8 Hz, 0.58F), -189.99 (dd, J = 47.2, 24.2 Hz, 0.42F). **HRMS** (ESI, m/z): calcd for $C_{26}H_{32}F_2O_2$ $[M+Na]^+$ 437.2263, found 437.2265.

(Z)-4-(1,4-difluoro-5-phenyl-2-propylpent-4-en-1-yl)phenyl 4-methylbenzenesulfonate (3u)

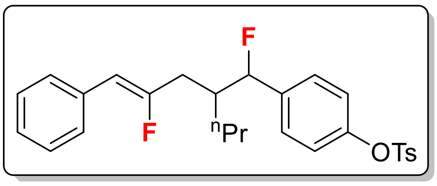

Following the general procedure (eluting with PE/EtOAc = 10/1). Isolated yield = 79% (37.1 mg); Pale yellow oil; Rf = 0.2 (PE:EA = 20:1); The reaction was analyzed by ^{19}F NMR of the crude

product giving 1.2:1 dr. **^{1}H NMR** (400 MHz, $CDCl_3$) δ 7.73 – 7.62 (m, 2H), 7.53 – 7.39 (m, 2H), 7.37 – 7.16 (m, 7H), 7.03 – 6.91 (m, 2H), 2.42 (s, 3H), 5.59 (dd, *J* = 46.7, 4.1 Hz, 0.43H), 5.52 (d, *J* = 39.4 Hz, 0.43H), 5.48 (dd, *J* = 46.8, 5.4 Hz, 0.57H), 5.47 (d, *J* = 39.2 Hz, 0.57H), 2.50 – 2.07 (m, 3H), 1.46 – 1.12 (m, 4H), 0.95 – 0.77 (m, 3H); **^{13}C NMR** (101 MHz, $CDCl_3$) δ 158.8 (d, *J* = 266.5 Hz), 158.5 (d, *J* = 266.0 Hz), 149.2 (d, *J* = 1.9 Hz), 149.0 (d, *J* = 1.5 Hz), 145.43, 145.42, 138.0 (d, *J* = 20.8 Hz), 137.9 (d, *J* = 20.9 Hz), 133.5 (d, *J* = 2.5 Hz), 133.3 (d, *J* = 2.7 Hz), 132.1, 129.69, 129.68, 128.5, 128.4, 128.37, 128.31, 128.25, 128.23, 128.18, 126.9 (d, *J* = 7.9 Hz), 126.8 (d, *J* = 1.8 Hz), 126.6 (d, *J* = 8.4 Hz), 108.3 (d, *J* = 8.4 Hz), 108.0 (d, *J* = 8.6 Hz), 94.4 (d, *J* = 176.1 Hz), 94.2 (d, *J* = 176.5 Hz), 41.5 (d, *J* = 21.1 Hz), 41.2 (d, *J* = 21.5 Hz), 34.5 – 33.6 (m), 32.3 (dd, *J* = 26.3, 5.4 Hz), 31.3 (d, *J* = 3.5 Hz), 29.6 (d, *J* = 4.0 Hz), 21.6, 20.0, 19.8, 14.0, 14.0; **^{19}F NMR** (376 MHz, $CDCl_3$) δ -101.01 (ddd, *J* = 41.0, 27.9, 14.9 Hz, 0.57F), -102.01 (ddd, *J* = 40.8, 24.3, 18.1 Hz, 0.43F), -187.24 (dd, *J* = 46.7, 22.7 Hz, 0.57F), -191.79 (dd, *J* = 46.1, 26.3 Hz, 0.43F). **HRMS** (ESI, m/z): calcd for $C_{27}H_{28}F_2O_3S$ $[M+Na]^+$ 493.1619, found 493.1622.

(Z)-(1,4-difluoro-2-methylpent-4-ene-1,1,5-triyl)tribenzene (3v)

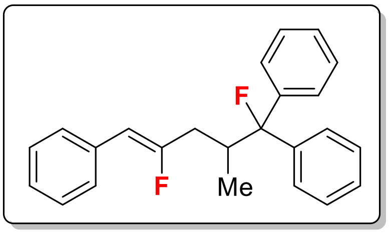

Following the general procedure (eluting with PE/DCM = 20/1). Isolated yield = 35% (12.0 mg); Colorless oil; Rf = 0.6 (PE:EA = 100:1); **^{1}H NMR** (400 MHz, $CDCl_3$) δ 7.53 – 7.47 (m, 2H), 7.47 – 7.42 (m, 4H), 7.38 – 7.27 (m, 6H), 7.26 – 7.16 (m, 3H), 5.44 (dd, *J* = 39.4, 1.0 Hz, 1H), 3.05 (dddd, *J* = 32.3, 11.0, 6.8, 2.8 Hz, 1H), 2.57 (ddd, *J* = 14.9, 9.0, 2.8 Hz, 1H), 2.13 (ddd, *J* = 33.6, 14.7, 11.0 Hz, 1H), 1.00 (d, *J* = 6.8 Hz, 3H); **^{13}C NMR** (101 MHz, $CDCl_3$) δ 159.5 (d, *J* = 265.8 Hz), 143.1 (t, *J* = 23.6 Hz), 128.5, 128.4, 128.2 (d, *J* = 7.6 Hz), 127.2 (d, *J* = 23.2 Hz), 126.7 (d, *J* = 2.0 Hz), 124.7 (d, *J* = 10.1 Hz), 124.5 (d, *J* = 10.4 Hz), 107.6 (d, *J* = 8.8 Hz), 101.4 (d, *J* = 183.0 Hz), 37.8 (d, *J* = 22.0 Hz), 35.4 (dd, *J* = 26.0, 3.6 Hz), 13.6 (d, *J* = 3.4 Hz); **^{19}F NMR** (376 MHz, $CDCl_3$) δ -102.89 (ddd, *J* = 40.9, 33.7, 8.8 Hz), -172.00 (d, *J* = 32.2 Hz). **HRMS** (EI, m/z): calcd for $C_{24}H_{22}F_2$ $[M]^{+\bullet}$ 348.1684, found 348.1688.

Note that: In addition to the allylic fluorination product **3v**, we can observe another two by-products such as Heck-type product and allylic hydroxylation product. We guessed that the formation of the Heck-type product may be due to the advantages conferred by the thermodynamic stability of

tetra-substituted alkenes. After treating Rh-catalyst under vacuum overnight, the hydroxylation products decreased somewhat to 17%, but the yield of target product **3v** still remained at 35%. This outcome suggests that the hydroxylation product may have originated from H_2O, and a little of H_2O did not seem to impact the yield of **3v**.

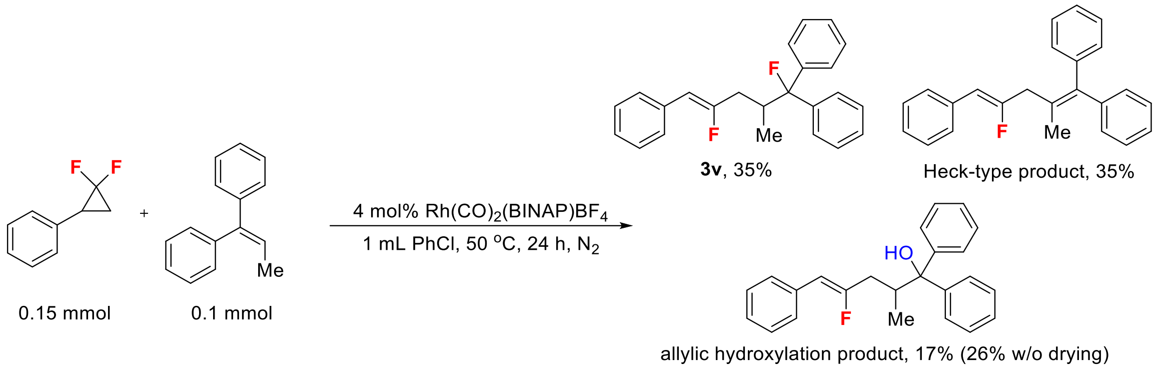

Supplementary Figure 6. The reaction of tri-substituted alkenes under standard conditions.

Moreover, we have attempted primary asymmetric explorations on this substrate. Screening some chiral BINAP derivatives showed that *R*-BINAP gave the desired product with 6% ee, whereas *R*-BINAP$^{3,5\text{-Me2}}$ led to a better result (29% ee). Replacing chiral BINAP derivatives with *R*-SegPhos or *R*-OMe-BIPHEP did not yield the product, with low conversion of the starting materials.

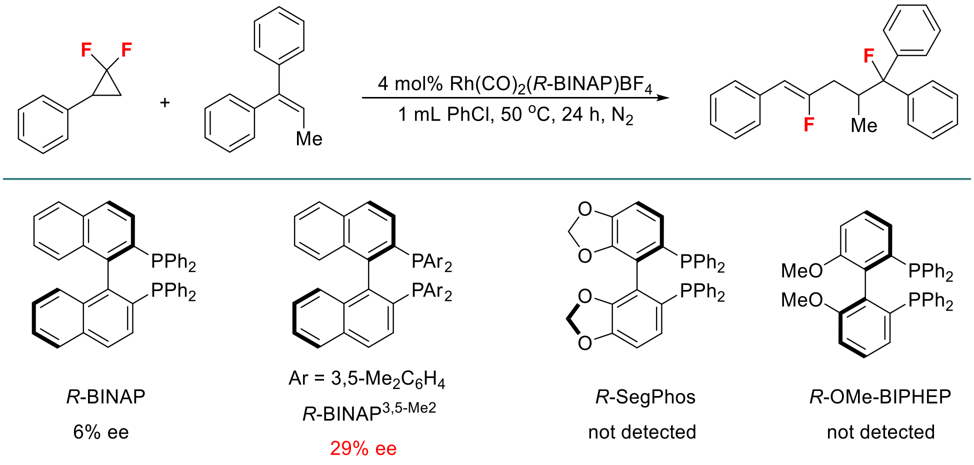

Supplementary Figure 7. Initially asymmetric experiment.

1-fluoro-2-((Z)-2-fluoro-3-phenylallyl)cyclohexyl)benzene (3w)

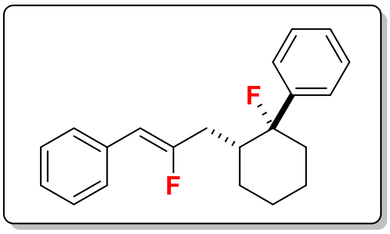

Following the general procedure (except the reaction was carried out at 40 °C, eluting with PE/DCM = 20/1). The carbofluorination product **3w** and Heck-type product were separated difficultly and isolated together (23.2 mg, **3w**: Heck-type product = 2:1). Corrected yield = 51%; The purification of **3w** relied on preparative Thin-Layer Chromatography; Colorless oil; Rf = 0.4 (PE:EA = 100:1); **^{1}H NMR** (400 MHz, $CDCl_3$) δ 7.42 – 7.34 (m, 6H), 7.33 – 7.23 (m, 3H), 7.21 – 7.13 (m, 1H), 5.32 (d, *J* = 39.3 Hz, 1H), 2.28 – 2.10 (m, 2H), 2.10 – 1.91 (m, 3H), 1.89 – 1.64 (m, 4H), 1.59 – 1.36 (m, 2H).**^{13}C NMR** (101 MHz, $CDCl_3$) δ 159.4 (d, *J* = 266.2 Hz), 144.0 (d, *J* = 21.9 Hz), 133.7, 128.4 (d, *J* = 2.3 Hz), 128.3, 128.2 (d, *J* = 7.4 Hz), 127.1, 126.6 (d, *J* = 2.2 Hz), 123.9 (d, *J* = 10.9 Hz), 107.4 (d, *J* = 8.9 Hz), 98.2 (d, *J* = 180.0 Hz), 41.7 (d, *J* = 21.9 Hz), 39.5 (d, *J* = 23.8 Hz), 34.2 (dd, *J* = 26.2, 2.9 Hz), 27.0, 25.3, 21.8. **^{19}F NMR** (376 MHz, $CDCl_3$) δ -102.32 (ddd, *J* = 41.2, 33.4, 10.3 Hz), -177.74 (dt, *J* = 45.3, 21.8 Hz).

(Z)-(2,5,5-trifluoropent-1-ene-1,5-diyl)dibenzene (3x)

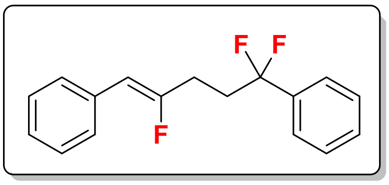

Following the general procedure (eluting with PE/DCM = 20/1). The carbofluorination product **3w** and the supposed allyl-branched product **3w'** were inseparable and isolated together (**3w**:**3w'** = 6.4:1). Isolated total yield = 61% (16.8 mg); Colorless oil; Rf = 0.2 (PE); **^{1}H NMR** (400 MHz, $CDCl_3$) δ 7.53 – 7.47 (m, 2H), 7.45 – 7.40 (m, 5H), 7.33 – 7.27 (m, 2H), 7.20 (tt, *J* = 6.7, 1.3 Hz, 1H), 5.48 (d, *J* = 39.1 Hz, 1H), 2.58 – 2.36 (m, 4H); **^{13}C NMR** (101 MHz, $CDCl_3$) δ 158.8 (d, *J* = 265.9 Hz), 136.7 (t, *J* = 26.3 Hz), 133.3 (d, *J* = 2.4 Hz), 129.9, 128.54, 128.43, 128.41, 128.3 (d, *J* = 7.3 Hz), 126.9 (d, *J* = 2.3 Hz), 124.9 (t, *J* = 6.2 Hz), 106.6 (d, *J* = 8.4 Hz), 36.1 (t, *J* = 28.2 Hz), 26.7 (dt, *J* = 27.9, 4.6 Hz); **^{19}F NMR** (376 MHz, $CDCl_3$) δ -96.57 (t, *J* = 15.0 Hz), -102.60 (dt, *J* = 38.8, 16.3 Hz). **HRMS** (EI, m/z): calcd for $C_{17}H_{15}F_3$ $[M]^{+\bullet}$ 276.1120, found 276.1122.

(Z)-1-methyl-4-(1,1,4-trifluoro-5-phenylpent-4-en-1-yl)benzene (3y)

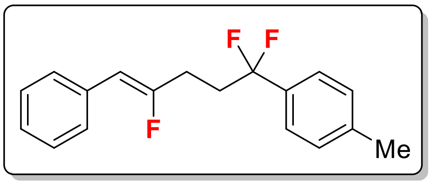

Following the general procedure (eluting with PE/DCM = 20/1). The carbofluorination product **3x** and the supposed allyl-branched product **3x'** were inseparable and isolated together (**3x**:**3x'** = 5.6:1). Isolated total yield = 84% (24.4 mg); Colorless oil; Rf = 0.2 (PE); **^{1}H NMR** (400 MHz, $CDCl_3$) δ 7.45

– 7.34 (m, 4H), 7.33 – 7.26 (m, 3H), 7.23 – 7.16 (m, 2H), 5.47 (d, J = 39.1 Hz, 1H), 2.57 – 2.38 (m, 4H), 2.37 (s, 3H); **^{13}C NMR** (101 MHz, $CDCl_3$) δ 158.9 (d, J = 265.8 Hz), 139.9, 133.8 (t, J = 26.5 Hz), 133.4 (d, J = 2.6 Hz), 129.2, 128.8 (d, J = 48.2 Hz), 128.4, 128.3 (d, J = 7.3 Hz), 126.9 (d, J = 2.4 Hz), 124.8 (t, J = 6.2 Hz), 106.5 (d, J = 8.4 Hz), 36.0 (t, J = 28.6 Hz), 26.8 (dt, J = 27.8, 4.5 Hz), 21.2; **^{19}F NMR** (376 MHz, $CDCl_3$) δ -95.78 (t, J = 15.2 Hz), -102.51 (dt, J = 39.0, 16.9 Hz). **HRMS** (EI, m/z): calcd for $C_{18}H_{17}F_3$ $[M]^{+\bullet}$ 290.1277, found 290.1280.

(Z)-4-(1,1,4-trifluoro-5-phenylpent-4-en-1-yl)-1,1'-biphenyl (3z)

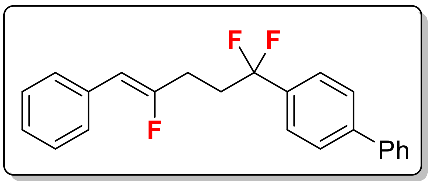

Following the general procedure (eluting with PE/DCM = 20/1). The carbofluorination product **3y** and the supposed allyl-branched product **3y'** were inseparable and isolated together (**3y**:**3y'** = 7.9:1). Isolated total yield = 88% (30.9 mg); White solid; m.p.: 87.4-89.0 °C; Rf = 0.1 (PE); **^{1}H NMR** (400 MHz, $CDCl_3$) δ 7.64 (d, J = 8.5 Hz, 2H), 7.60 – 7.53 (m, 4H), 7.49 – 7.41 (m, 4H), 7.40 – 7.35 (m, 1H), 7.30 (dd, J = 8.5, 6.9 Hz, 2H), 7.20 (tt, J = 6.7, 1.3 Hz, 1H), 5.49 (d, J = 39.1 Hz, 1H), 2.61 – 2.39 (m, 4H); **^{13}C NMR** (101 MHz, $CDCl_3$) δ 158.8 (d, J = 265.8 Hz), 142.9, 140.1, 135.5 (t, J = 26.5 Hz), 133.3, 128.9, 128.4, 128.3 (d, J = 7.3 Hz), 127.8, 127.3, 127.17, 127.19 (d, J = 1.9 Hz), 126.9 (d, J = 2.3 Hz), 125.4 (t, J = 6.2 Hz), 106.6 (d, J = 8.4 Hz), 36.0 (t, J = 28.2 Hz), 26.7 (dt, J = 28.3, 4.9 Hz); **^{19}F NMR** (376 MHz, $CDCl_3$) δ -96.08 (t, J = 15.3 Hz), -102.62 (dt, J = 39.0, 16.2 Hz). **HRMS** (EI, m/z): calcd for $C_{23}H_{19}F_3$ $[M]^{+\bullet}$ 352.1433, found 352.1440.

(Z)-1-chloro-4-(1,1,4-trifluoro-5-phenylpent-4-en-1-yl)benzene (3aa)

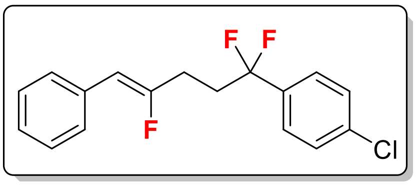

Following the general procedure (eluting with PE/DCM = 20/1). The carbofluorination product **3z** and the supposed allyl-branched product **3z'** were inseparable and isolated together (**3z**:**3z'** = 5:1). Isolated total yield = 67% (20.8 mg); Colorless oil; Rf = 0.2 (PE); **^{1}H NMR** (400 MHz, $CDCl_3$) δ 7.45 – 7.38 (m, 6H), 7.33 – 7.28 (m, 2H), 7.23 – 7.17 (m, 1H), 5.48 (d, J = 39.0 Hz, 1H), 2.56 – 2.33 (m, 4H); **^{13}C NMR** (101 MHz, $CDCl_3$) δ 158.5 (d, J = 265.9 Hz), 136.1, 135.2 (t, J = 26.8 Hz), 133.2 (d, J = 2.2 Hz), 128.8, 128.7 (d, J = 2.9 Hz), 128.4, 128.3 (d, J = 7.3 Hz), 127.0 (d, J = 2.3 Hz), 126.4 (t, J = 6.0 Hz), 106.8 (d, J = 8.4 Hz), 36.0 (t, J = 28.0 Hz), 26.6 (dt, J = 27.9, 4.7 Hz); **^{19}F NMR** (376 MHz, $CDCl_3$) δ -96.41 (t, J = 16.1 Hz), -102.93 (dt, J = 39.0, 17.4 Hz). **HRMS** (EI, m/z): calcd for

$C_{17}H_{14}ClF_3$ $[M]^{+\bullet}$ 310.0731, found 310.0732.

Note that: In this reaction, we can observe a certain amount of another isomeric product **3z'** when using the α-fluorostyrene as substrates. We assumed **3z'** as the allyl-branched product based on the typical coupling pattern of the terminal fluoro-olefin in the ^{1}H NMR spectrum[25] and typical coupling pattern of the "CF_2" motif in the ^{19}F NMR spectrum of this mixture: **^{1}H NMR** (400 MHz, $CDCl_3$) δ 4.57 (dd, J = 17.3, 3.2 Hz, 1H), 4.31 (dd, J = 49.4, 3.2 Hz, 1H), 3.70 (dt, J = 20.3, 6.9 Hz, 1H), 2.85 (dddd, J = 19.4, 14.9, 12.3, 7.2 Hz, 1H). **^{19}F NMR** (376 MHz, CDCl3) δ -93.45 (ddd, J = 247.2, 19.4, 11.1 Hz), -94.97 (ddd, J = 247.0, 19.4, 13.0 Hz), -101.98 (dt, J = 50.0, 18.7 Hz). The allyl-branched product was also observed in **3w-3ab** and the regioselectivity was determined by ^{19}F NMR analysis of the crude product.

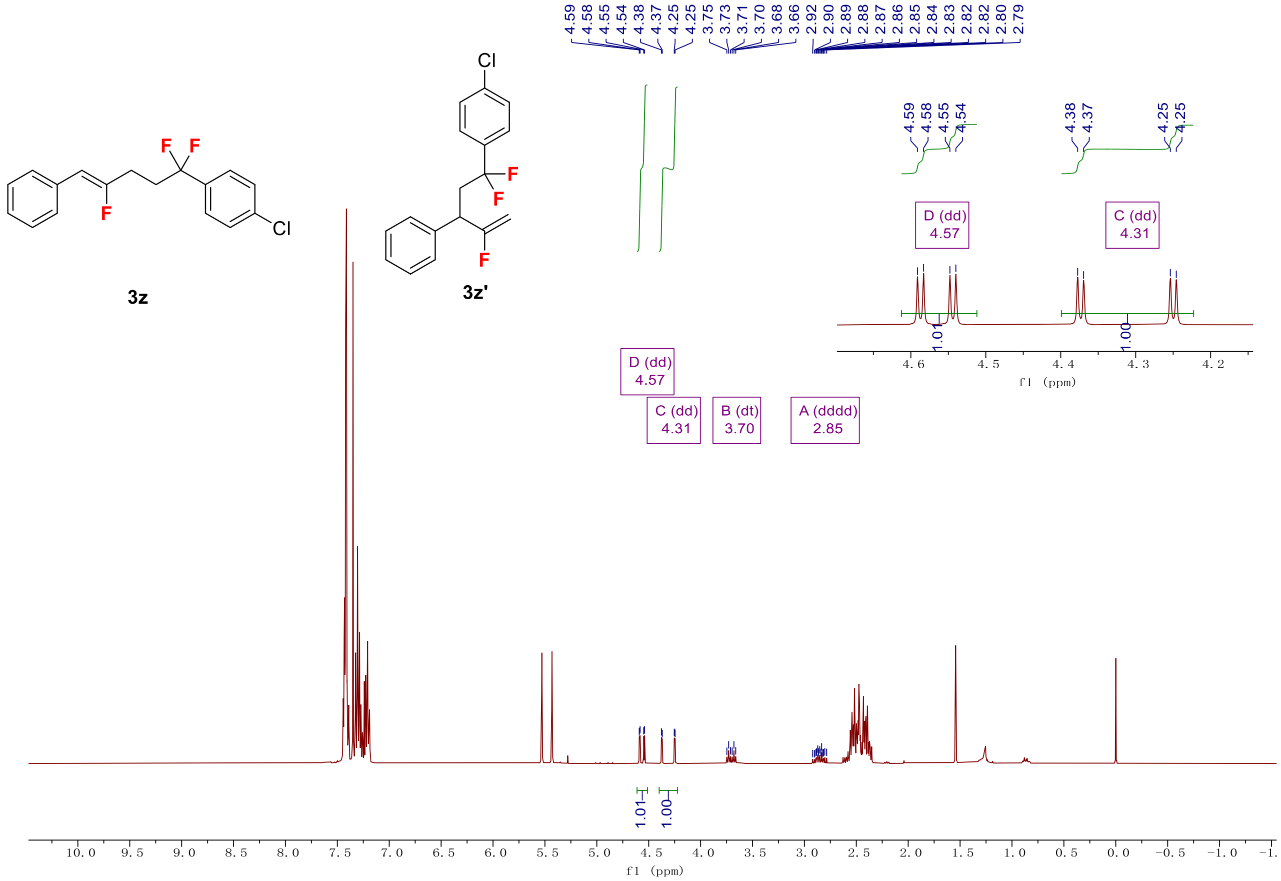

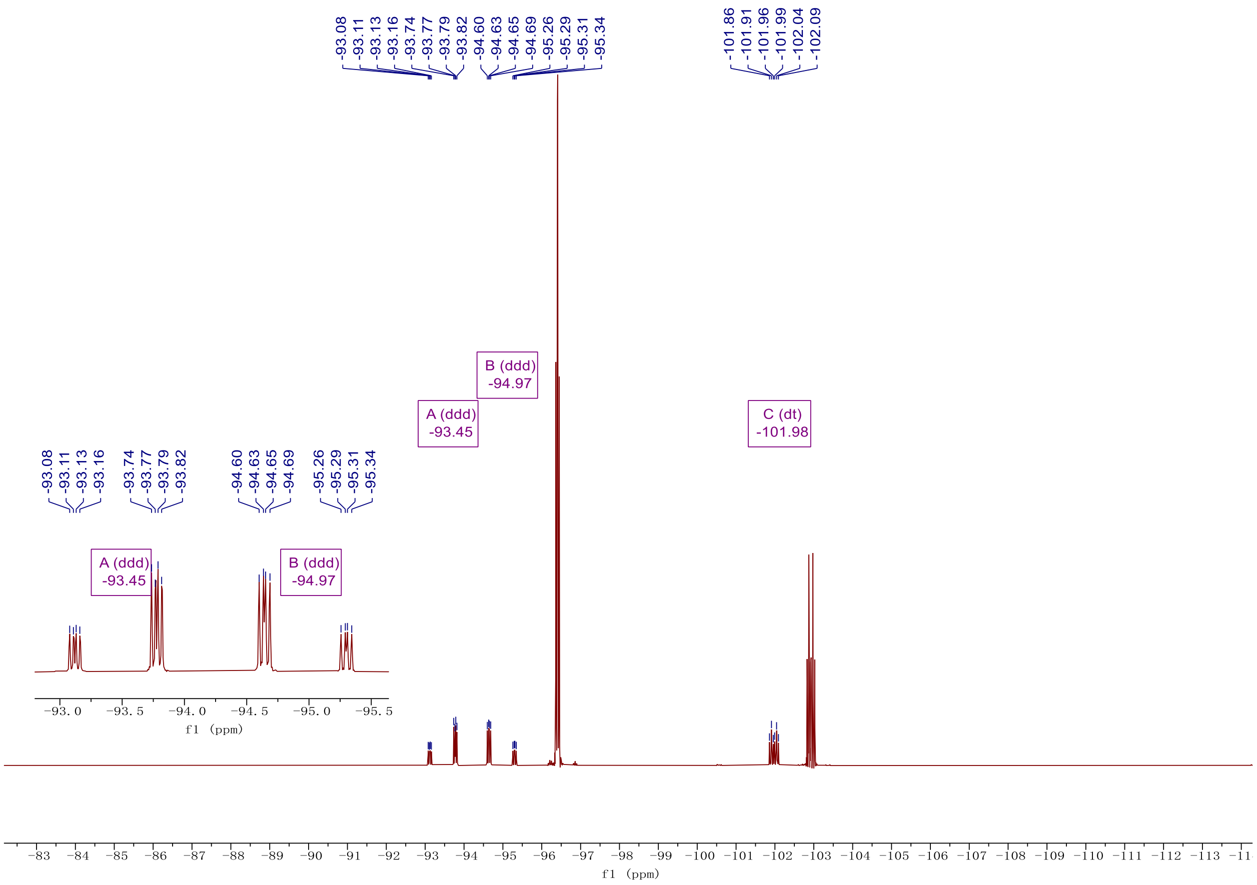

Supplementary Figure 8. The ^{1}H NMR spectrum of the products 3z and 3z'.

(Z)-2-(1,1,4-trifluoro-5-phenylpent-4-en-1-yl)naphthalene (3ab)

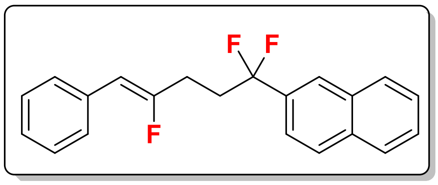

Following the general procedure (eluting with PE/DCM = 20/1). The carbofluorination product **3aa** and the supposed allyl-branched product **3aa'** were inseparable and isolated together (**3aa**:**3aa'** = 16:1). Isolated total yield = 80% (26.0 mg); Colorless oil; Rf = 0.1 (PE); **^{1}H NMR** (400 MHz, $CDCl_3$) δ 8.00 (s, 1H), 7.92 – 7.80 (m, 3H), 7.59 – 7.50 (m, 3H), 7.44 – 7.35 (m, 2H), 7.31 – 7.26 (m, 2H), 7.22 – 7.16 (m, 1H), 5.47 (d, J = 39.1 Hz, 1H), 2.62 – 2.44 (m, 4H); **^{13}C NMR** (101 MHz, $CDCl_3$) δ 158.8 (d, J = 265.9 Hz), 133.7 (t, J = 13.2 Hz), 133.3 (d, J = 2.5 Hz), 132.5, 128.7, 128.5, 128.4, 128.32, 128.25, 127.7, 127.2, 126.9 (d, J = 2.3 Hz), 126.8, 124.7 (t, J = 7.1 Hz), 122.4, 122.0 (t, J = 5.5 Hz), 106.6 (d, J = 8.5 Hz), 36.1 (t, J = 28.3 Hz), 26.8 (dt, J = 27.7, 4.5 Hz); **^{19}F NMR** (376 MHz, $CDCl_3$) δ -96.01 (t, J = 14.3 Hz), -102.59 (ddd, J = 38.9, 19.3, 14.0 Hz). **HRMS** (EI, m/z): calcd for $C_{21}H_{17}F_3$ $[M]^{+\bullet}$ 326.1277, found 326.1282.

(Z)-(2,5,5-trifluoro-4-methylpent-1-ene-1,5-diyl)dibenzene (3ac)

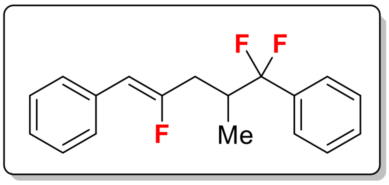

Following the general procedure (eluting with PE/DCM = 20/1). The carbofluorination product **3ab** and the supposed allyl-branched product **3ab'** were inseparable and isolated together (**3ab**:**3ab'** > 20:1). Isolated total yield = 51% (14.7 mg); Colorless oil; Rf = 0.2 (PE); **^{1}H NMR** (400 MHz, $CDCl_3$) δ 7.50 – 7.40 (m, 7H), 7.37 – 7.28 (m, 2H), 7.23 – 7.17 (m, 1H), 5.49 (d, *J* = 39.1 Hz, 1H), 2.80 (ddd, *J* = 14.3, 10.6, 3.5 Hz, 1H), 2.60 (ddddd, *J* = 15.9, 12.3, 10.6, 7.0, 3.6 Hz, 1H), 2.12 (ddd, *J* = 30.5, 14.5, 10.9 Hz, 1H), 1.04 (d, *J* = 6.9 Hz, 3H); **^{13}C NMR** (101 MHz, $CDCl_3$) δ 158.1 (d, *J* = 266.4 Hz), 135.9 (t, *J* = 26.7 Hz), 133.4 (d, *J* = 2.7 Hz), 129.8, 128.4, 128.35, 128.33, 128.26, 126.9 (d, *J* = 2.3 Hz), 125.4 (t, *J* = 6.5 Hz), 108.1 (d, *J* = 8.7 Hz), 39.1 (t, *J* = 26.5 Hz), 33.9 (dt, *J* = 26.4, 4.3 Hz), 12.8 (t, *J* = 4.2 Hz); **^{19}F NMR** (376 MHz, $CDCl_3$) δ -101.16 (dd, *J* = 243.5, 12.3 Hz), -102.91 (ddd, *J* = 40.3, 30.4, 11.1 Hz), -105.50 (dd, *J* = 243.3, 16.1 Hz). **HRMS** (EI, m/z): calcd for $C_{18}H_{17}F_3$ $[M]^{+\bullet}$ 290.1277, found 290.1281.

(Z)-1-(2,5-difluoro-5-phenylpent-1-en-1-yl)-4-methylbenzene (4b)

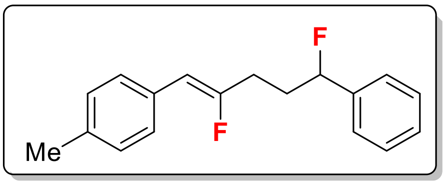

Following the general procedure (except the reaction was carried out using $[Rh(CO)_2(BINAP^{Me})BF_4]$ as catalyst; eluting with PE/DCM = 20/1). Isolated yield = 71% (19.3 mg); Colorless oil; Rf = 0.2 (PE); **^{1}H NMR** (400 MHz, $CDCl_3$) δ 7.43 – 7.31 (m, 7H), 7.13 (d, *J* = 8.0 Hz, 2H), 5.53 (ddd, *J* = 47.7, 8.5, 4.3 Hz, 1H), 5.49 (d, *J* = 39.6 Hz, 1H), 2.56 – 2.44 (m, 2H), 2.33 (s, 3H), 2.29 – 2.03 (m, 2H); **^{13}C NMR** (101 MHz, $CDCl_3$) δ 158.9 (d, *J* = 264.9 Hz), 139.7 (d, *J* = 19.7 Hz), 136.6, 130.6, 129.1, 128.5, 128.4 (d, *J* = 2.0 Hz), 128.2 (d, *J* = 7.2 Hz), 125.5 (d, *J* = 6.8 Hz), 106.5 (d, *J* = 8.6 Hz), 93.3 (d, *J* = 171.4 Hz), 33.9 (d, *J* = 24.2 Hz), 28.9 (dd, *J* = 27.2, 4.3 Hz), 21.2; **^{19}F NMR** (376 MHz, $CDCl_3$) δ -103.15 (dt, *J* = 38.0, 18.3 Hz), -176.92 (ddd, *J* = 47.1, 30.1, 16.5 Hz). **HRMS** (EI, m/z): calcd for $C_{18}H_{18}F_2$ $[M]^{+\bullet}$ 272.1371, found 272.1373.

(Z)-1-(tert-butyl)-4-(2,5-difluoro-5-phenylpent-1-en-1-yl)benzene (4c)

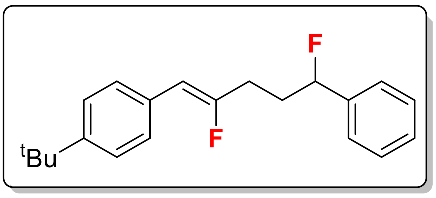

Following the general procedure (except the reaction was carried out using [Rh(CO)$_2$(BINAP^{Me})BF$_4$] as catalyst; eluting with PE/DCM = 20/1). Isolated yield = 86% (27.0 mg); Colorless oil; Rf = 0.2 (PE); ^{1}**H NMR** (400 MHz, CDCl$_3$) δ 7.45 – 7.32 (m, 9H), 5.52 (ddd, *J* = 48.8, 8.7, 4.2 Hz, 1H), 5.51 (d, *J* = 39.8 Hz, 1H), 2.62 – 2.41 (m, 2H), 2.33 – 2.04 (m, 2H), 1.31 (s, 9H); ^{13}**C NMR** (101 MHz, CDCl$_3$) δ 159.1 (d, *J* = 265.3 Hz), 149.9, 139.7 (d, *J* = 19.7 Hz), 130.6, 128.5, 128.4 (d, *J* = 2.0 Hz), 128.0 (d, *J* = 7.2 Hz), 125.5 (d, *J* = 6.8 Hz), 125.3, 106.4 (d, *J* = 8.7 Hz), 93.3 (d, *J* = 171.5 Hz), 34.5, 33.9 (d, *J* = 24.2 Hz), 31.2, 28.9 (dd, *J* = 27.4, 4.3 Hz); ^{19}**F NMR** (376 MHz, CDCl$_3$) δ -102.95 – -103.26 (m), -176.94 (ddd, *J* = 47.3, 30.4, 16.5 Hz). **HRMS** (EI, m/z): calcd for $C_{21}H_{24}F_2$ $[M]^{+\bullet}$ 314.1841, found 314.1844.

(Z)-2-(2,5-difluoro-5-phenylpent-1-en-1-yl)naphthalene (4d)

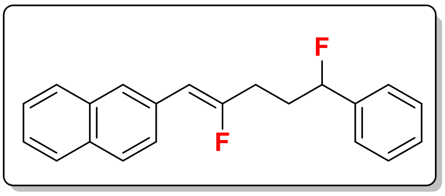

Following the general procedure (except the reaction was carried out using [Rh(CO)$_2$(BINAP^{OMe})BF$_4$] as catalyst; eluting with PE/DCM = 20/1). Isolated yield = 56% (17.2 mg); White solid; m.p.: 102.3-104.1 °C; Rf = 0.1 (PE); ^{1}**H NMR** (400 MHz, CDCl$_3$) δ 7.88 (s, 1H), 7.81 – 7.74 (m, 3H), 7.63 (dd, *J* = 8.6, 1.8 Hz, 1H), 7.49 – 7.30 (m, 7H), 5.67 (d, *J* = 39.2 Hz, 1H), 5.55 (dd, *J* = 47.8, 8.5, 4.3 Hz, 1H), 2.56 (tdd, *J* = 15.1, 6.9, 3.5 Hz, 2H), 2.36 – 2.09 (m, 2H); ^{13}**C NMR** (101 MHz, CDCl$_3$) δ 159.9 (d, *J* = 266.8 Hz), 139.7 (d, *J* = 19.7 Hz), 133.4, 132.3, 131.0 (d, *J* = 2.9 Hz), 128.6, 128.5 (d, *J* = 1.9 Hz), 127.9 (d, *J* = 4.3 Hz), 127.5, 127.2 (d, *J* = 7.5 Hz), 126.5 (d, *J* = 7.6 Hz), 126.1, 125.8, 125.5, 125.4, 106.8 (d, *J* = 8.4 Hz), 93.3 (d, *J* = 171.6 Hz), 33.9 (d, *J* = 24.2 Hz), 29.1 (dd, *J* = 27.1, 4.4 Hz); ^{19}**F NMR** (376 MHz, CDCl$_3$) δ -101.51 (dt, *J* = 37.0, 18.3 Hz), -176.87 (ddd, *J* = 47.9, 29.5, 16.5 Hz). **HRMS** (EI, m/z): calcd for $C_{21}H_{18}F_2$ $[M]^{+\bullet}$ 308.1371, found 308.1376.

(Z)-1-(2,5-difluoro-5-phenylpent-1-en-1-yl)-4-methoxybenzene (4e)

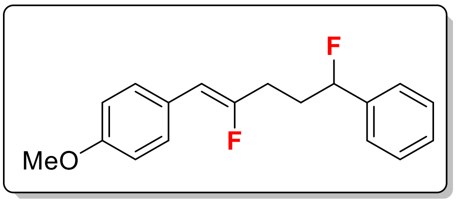

Following the general procedure (except the reaction was carried out for 48 h using [Rh(CO)$_2$(BINAP^{NMe2})BF$_4$] as catalyst; eluting with PE/DCM = 10/1). Isolated yield = 51% (14.7 mg);

Pale yellow oil; Rf = 0.4 (PE:EA = 100:1); **^{1}H NMR** (400 MHz, $CDCl_3$) δ 7.47 – 7.31 (m, 7H), 6.89 – 6.82 (m, 2H), 5.52 (ddd, J = 47.8, 8.6, 4.4 Hz, 1H), 5.46 (d, J = 39.7 Hz, 1H), 3.80 (s, 3H), 2.56 – 2.42 (m, 2H), 2.32 – 2.03 (m, 2H); **^{13}C NMR** (101 MHz, $CDCl_3$) δ 158.4 (d, J = 2.8 Hz), 158.2 (d, J = 263.6 Hz), 139.7 (d, J = 19.7 Hz), 129.5 (d, J = 7.4 Hz), 128.5, 128.4 (d, J = 2.0 Hz), 126.2 (d, J = 1.9 Hz), 125.5 (d, J = 7.0 Hz), 113.8, 106.0 (d, J = 8.9 Hz), 93.3 (d, J = 171.5 Hz), 55.2, 34.0 (d, J = 24.3 Hz), 28.9 (dd, J = 27.5, 4.5 Hz); **^{19}F NMR** (376 MHz, $CDCl_3$) δ -104.95 – -105.22 (m), -176.88 (ddd, J = 46.6, 29.7, 16.5 Hz). **HRMS** (ESI, m/z): calcd for $C_{18}H_{18}F_2O$ $[M+Na]^+$ 311.1218, found 311.1220.

(Z)-4-(2,5-difluoro-5-phenylpent-1-en-1-yl)-1,1'-biphenyl (4f)

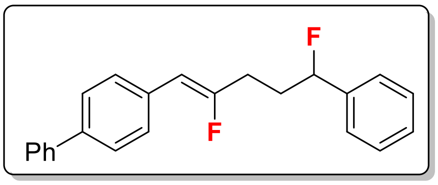

Following the general procedure (except the reaction was carried out using $[Rh(CO)_2(BINAP^{OMe})BF_4]$ as catalyst; eluting with PE/DCM = 20/1). Isolated yield = 72% (24.0 mg); White solid; m.p.: 91.4-93.2°C; Rf = 0.1 (PE); **^{1}H NMR** (400 MHz, $CDCl_3$) δ 7.65 – 7.50 (m, 6H), 7.47 – 7.30 (m, 8H), 5.57 (d, J = 39.2 Hz, 1H), 5.54 (ddd, J = 47.8, 8.1, 3.9 Hz, 1H), 2.63 – 2.46 (m, 2H), 2.35 – 2.06 (m, 2H); **^{13}C NMR** (101 MHz, $CDCl_3$) δ 159.8 (d, J = 266.4 Hz), 140.7, 139.6 (d, J = 22.1 Hz), 139.5 (d, J = 4.9 Hz), 132.5 (d, J = 2.6 Hz), 128.8, 128.7, 128.6, 128.5 (d, J = 2.0 Hz), 127.3, 127.1, 126.9, 125.5 (d, J = 6.8 Hz), 106.3 (d, J = 8.6 Hz), 93.3 (d, J = 171.6 Hz), 33.9 (d, J = 24.2 Hz), 29.0 (dd, J = 26.8, 3.9 Hz); **^{19}F NMR** (376 MHz, $CDCl_3$) δ -101.51 (dt, J = 37.4, 18.2 Hz), -176.96 (ddd, J = 46.8, 29.8, 16.6 Hz). **HRMS** (EI, m/z): calcd for $C_{23}H_{20}F_2$ $[M]^{+\bullet}$ 334.1528, found 334.1533.

(Z)-1-(2,5-difluoro-5-phenylpent-1-en-1-yl)-4-fluorobenzene (4g)

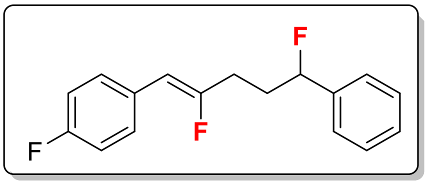

Following the general procedure (except the reaction was carried out using $[Rh(CO)_2(BINAP^{OMe})BF_4]$ as catalyst; eluting with PE/DCM = 20/1). Isolated yield = 51% (14.1 mg); Colorless oil; Rf = 0.2 (PE); **^{1}H NMR** (400 MHz, $CDCl_3$) δ 7.49 – 7.31 (m, 7H), 7.04 – 6.96 (m, 2H), 5.53 (ddd, J = 47.7, 8.5, 4.3 Hz, 1H), 5.49 (d, J = 39.0 Hz, 1H), 2.61 – 2.38 (m, 2H), 2.32 – 2.03 (m, 2H); **^{13}C NMR** (101 MHz, $CDCl_3$) δ 161.5 (dd, J = 246.5, 3.6 Hz), 159.2 (dd, J = 265.2, 2.4 Hz), 139.6 (d, J = 19.6 Hz), 129.9 (t, J = 7.7 Hz), 129.6 (t, J = 3.3 Hz), 128.6, 128.5 (d, J = 2.2 Hz), 125.4 (d, J = 6.8 Hz), 115.3 (d, J = 21.6 Hz), 105.6 (d, J = 8.7 Hz), 93.3 (d, J = 171.7 Hz), 33.9 (d, J = 24.1 Hz), 28.9 (dd, J = 27.2, 4.5 Hz); **^{19}F NMR** (376 MHz, $CDCl_3$) δ -103.38 (dt, J = 38.6, 18.5 Hz), -114.63 (p, J = 8.1,

7.3 Hz), -176.88 – -177.25 (m). **HRMS** (EI, m/z): calcd for $C_{17}H_{15}F_3$ $[M]^{+\bullet}$ 276.1120, found 276.1123.

(Z)-1-(2,5-difluoro-5-phenylpent-1-en-1-yl)-3-methylbenzene (4h)

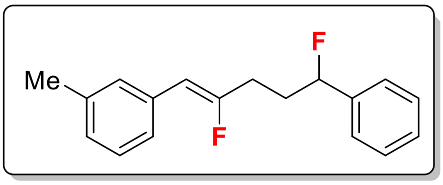

Following the general procedure (except the reaction was carried out using $PhCF_3$ as solvent; eluting with PE/DCM = 20/1). Isolated yield = 60% (16.3 mg); Colorless oil; Rf = 0.2 (PE); **^{1}H NMR** (400 MHz, $CDCl_3$) δ 7.44 – 7.31 (m, 5H), 7.27 (d, *J* = 7.8 Hz, 2H), 7.21 (t, *J* = 7.6 Hz, 1H), 7.03 (d, *J* = 7.5 Hz, 1H), 5.52 (ddd, *J* = 46.6, 7.9, 3.7 Hz, 1H), 5.49 (d, *J* = 39.3 Hz, 1H), 2.56 – 2.44 (m, 2H), 2.34 (s, 3H), 2.30 – 2.04 (m, 2H); **^{13}C NMR** (101 MHz, $CDCl_3$) δ 159.4 (d, *J* = 266.0 Hz), 139.7 (d, *J* = 19.8 Hz), 137.9, 133.4 (d, *J* = 2.5 Hz), 129.0 (d, *J* = 7.0 Hz), 128.5, 128.4 (d, *J* = 2.0 Hz), 128.3, 127.7 (d, *J* = 1.9 Hz), 125.5 (d, *J* = 6.9 Hz), 125.4 (d, *J* = 7.4 Hz), 106.7 (d, *J* = 8.4 Hz), 93.3 (d, *J* = 171.5 Hz), 33.9 (d, *J* = 24.1 Hz), 28.9 (dd, *J* = 27.2, 4.4 Hz), 21.4; **^{19}F NMR** (376 MHz, $CDCl_3$) δ -102.18 (dt, *J* = 38.9, 19.2 Hz), -176.92 (ddd, *J* = 46.8, 29.9, 16.6 Hz). **HRMS** (EI, m/z): calcd for $C_{18}H_{18}F_2$ $[M]^{+\bullet}$ 272.1371, found 272.1371.

7. Synthetic Applications

Gram-Scale Synthesis

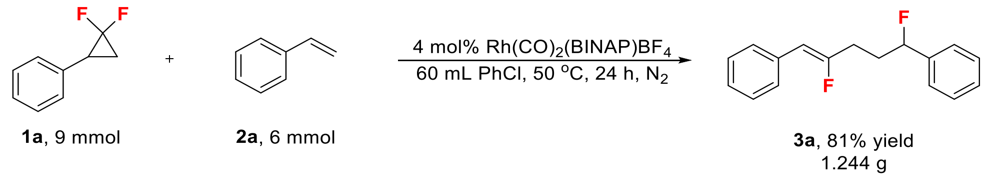

In a nitrogen filled glove box, a 250 mL three-necked flask equipped with a magnetic stir bar was charged with $[Rh(CO)_2(BINAP)BF_4]$ (210 mg, 0.24 mmol, 4 mol%) and PhCl (60 mL). The mixture was stirred for about 10 min, which afforded a yellow catalyst solution, and then *gem*-difluorinated cyclopropane **1a** (1.39 g, 9.0 mmol) and alkene **2a** (624 mg, 6.0 mmol). The flask was sealed and removed from the glove box and stirred at 50 °C for 24 hours. The reaction mixture was cooled to room temperature and purified by chromatography on silica gel column to give the benzyl fluoride **3a** (1.244 g, 81% yield).

Defluorinative Coupling

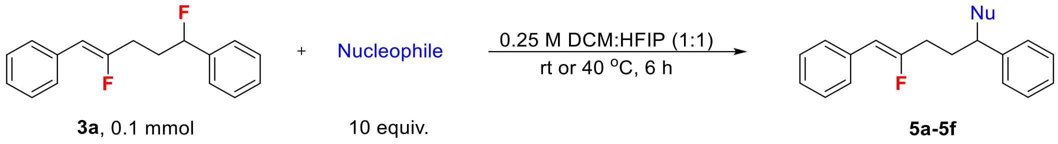

In the glove box, a 4 mL vial equipped with stir bar was charge with **3a** (31.4 mg, 0.1 mmol), nucleophile (10 equiv), DCM (0.2 mL). To this solution was added HFIP (0.2 mL) at room temperature. The vial was removed from the glove box and stirred at room temperature or 40 °C for 6 hours. After completion of the reaction, the reaction mixture diluted with EtOAc and purified by chromatography on silica gel column to give the desired product **5**[26].

(Z)-(5-(2,5-dimethylphenyl)-2-fluoropent-1-ene-1,5-diyl)dibenzene (5a)

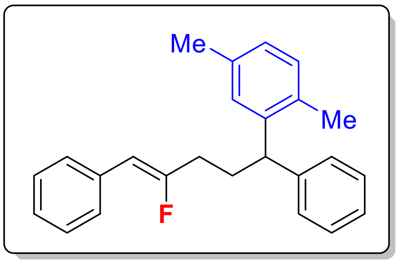

Following the general procedure of defluorinative coupling using *p*-xylene as nucleophile (except the reaction was carried out using pure HFIP as solvent; eluting with PE/DCM = 20/1). Isolated yield = 56% (19.2 mg); Colorless oil; Rf = 0.2 (PE); **^{1}H NMR** (400 MHz, $CDCl_3$) δ 7.48 – 7.40 (m, 2H), 7.34 – 7.25 (m, 4H), 7.23 – 7.14 (m, 5H), 7.02 (d, *J* = 7.6 Hz, 1H), 6.94 (dd, *J* = 7.7, 1.8 Hz, 1H), 5.39 (d, *J* = 39.5 Hz, 1H), 4.16 (t, *J* = 7.0 Hz, 1H), 2.34 (s, 4H), 2.38 – 2.27 (m, 3H), 2.21 (s, 3H); **^{13}C NMR** (101 MHz, $CDCl_3$) δ 160.5 (d, *J* = 266.5 Hz), 143.9, 141.6, 135.4, 133.7 (d, *J* = 2.3 Hz), 133.2, 130.5, 128.41, 128.39, 128.2 (d, *J* = 7.2 Hz), 128.2, 127.2, 126.9, 126.7 (d, *J* = 1.8 Hz), 126.1, 106.2 (d, *J* = 8.7 Hz), 45.8, 32.5, 31.5 (d, *J* = 26.4 Hz), 21.3, 19.4; **^{19}F NMR** (376 MHz, $CDCl_3$) δ -101.10 (dddd, *J* = 39.2, 17.4, 11.0, 6.7 Hz). **HRMS** (EI, m/z): calcd for $C_{25}H_{25}F$ $[M]^{+\bullet}$ 344.1935, found 344.1940.

(Z)-1-(4-fluoro-1,5-diphenylpent-4-en-1-yl)-1H-pyrazole (5b)

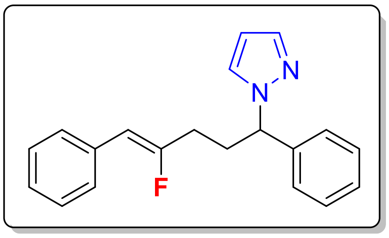

Following the general procedure of defluorinative coupling using pyrazole as nucleophile (eluting with PE/DCM = 20/1). Isolated yield = 80% (24.5 mg); Colorless oil; Rf = 0.4 (PE:DCM = 50:1); **^{1}H NMR** (400 MHz, $CDCl_3$) δ 7.59 (d, *J* = 1.8 Hz, 1H), 7.49 – 7.40 (m, 3H), 7.36 – 7.26 (m, 7H), 7.21 (tt, *J* = 6.7, 1.3 Hz, 1H), 6.28 (t, *J* = 2.1 Hz, 1H), 5.42 (d, *J* = 39.4 Hz, 1H), 5.37 (dd, *J* = 10.2, 5.3 Hz, 1H), 2.78 (dddd, *J* = 13.6, 9.5, 8.0, 5.6 Hz, 1H), 2.47 (dtd, *J* = 13.6, 7.6, 5.9 Hz, 1H), 2.41 – 2.18 (m, 2H); **^{13}C NMR** (101 MHz, $CDCl_3$) δ 159.3 (d, *J* = 266.3 Hz), 140.3, 139.5,

133.4, 128.82, 128.76, 128.4, 128.3 (d, J = 7.5 Hz), 128.0, 126.9 (d, J = 2.2 Hz), 126.7, 106.9 (d, J = 8.5 Hz), 105.6, 64.8, 31.9, 30.1 (d, J = 26.8 Hz); **^{19}F NMR** (376 MHz, $CDCl_3$) δ -102.14 (dt, J = 36.9, 18.4 Hz). **HRMS** (ESI, m/z): calcd for $C_{20}H_{19}FN_2$ $[M+H]^+$ 307.1605, found 307.1607.

(Z)-(2-fluoro-5-methoxypent-1-ene-1,5-diyl)dibenzene (5c)

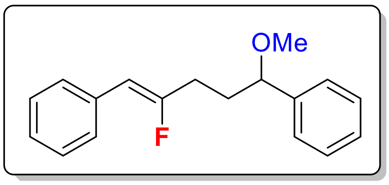

Following the general procedure of defluorinative coupling using methanol as nucleophile (eluting with PE/DCM = 10/1). Isolated yield = 77% (20.8 mg); Pale yellow oil; Rf = 0.4 (PE:EA = 100:1); **^{1}H NMR** (400 MHz, $CDCl_3$) δ 7.49 – 7.43 (m, 2H), 7.40 – 7.26 (m, 7H), 7.20 (tt, J = 6.8, 1.3 Hz, 1H), 5.47 (d, J = 39.4 Hz, 1H), 4.18 (dd, J = 8.1, 5.2 Hz, 1H), 3.23 (s, 3H), 2.48 – 2.35 (m, 2H), 2.06 (dtd, J = 14.3, 8.2, 6.2 Hz, 1H), 1.94 (dddd, J = 13.9, 8.7, 7.0, 5.2 Hz, 1H); **^{13}C NMR** (101 MHz, $CDCl_3$) δ 160.5 (d, J = 266.6 Hz), 141.7, 133.8, 128.5, 128.4, 128.3 (d, J = 7.3 Hz), 127.7, 126.7 (d, J = 2.8 Hz), 126.6, 106.1 (d, J = 8.7 Hz), 82.6, 56.7, 34.7, 29.5 (d, J = 26.6 Hz); **^{19}F NMR** (376 MHz, $CDCl_3$) δ -101.12 (dt, J = 37.7, 18.5 Hz). **HRMS** (ESI, m/z): calcd for $C_{18}H_{19}FO$ $[M+Na]^+$ 293.1312, found 293.1313.

(Z)-(5-(3-bromopropoxy)-2-fluoropent-1-ene-1,5-diyl)dibenzene (5d)

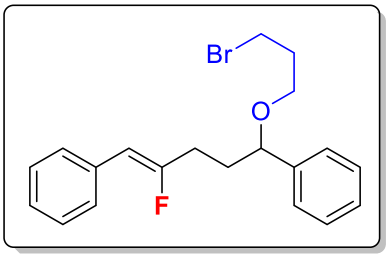

Following the general procedure of defluorinative coupling using 3-bromopropan-1-ol as nucleophile (eluting with PE/DCM = 10/1). Isolated yield = 75% (28.2 mg); Colorless oil; Rf = 0.4 (PE:EA = 100:1); **^{1}H NMR** (400 MHz, $CDCl_3$) δ 7.52 – 7.42 (m, 2H), 7.40 – 7.27 (m, 7H), 7.20 (tt, J = 6.9, 1.3 Hz, 1H), 5.50 (d, J = 39.5 Hz, 1H), 4.29 (dd, J = 8.5, 4.8 Hz, 1H), 3.54 (ddt, J = 27.3, 9.8, 6.6 Hz, 2H), 3.41 (qt, J = 9.5, 5.7 Hz, 2H), 2.53 – 2.33 (m, 2H), 2.13 – 1.99 (m, 3H), 1.93 (dddd, J = 13.7, 8.6, 7.1, 4.9 Hz, 1H); **^{13}C NMR** (101 MHz, $CDCl_3$) δ 160.4 (d, J = 266.9 Hz), 142.0, 133.7 (d, J = 2.1 Hz), 128.5, 128.4, 128.3 (d, J = 7.3 Hz), 127.7, 126.7 (d, J = 2.0 Hz), 126.5, 106.2 (d, J = 8.6 Hz), 81.1, 66.1, 34.8, 33.0, 30.7, 29.6 (d, J = 26.6 Hz); **^{19}F NMR** (376 MHz, $CDCl_3$) δ -101.20 (dt, J = 38.5, 18.5 Hz). **HRMS** (ESI, m/z): calcd for $C_{20}H_{22}{}^{79}BrFO$ $[M+Na]^+$ 399.0730, found 399.0737.

(Z)-(2-fluoroocta-1,7-diene-1,5-diyl)dibenzene (5f)

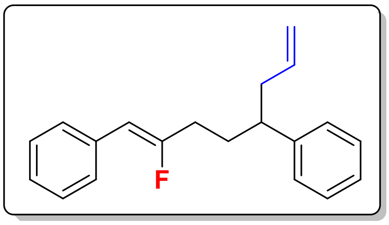

Following the general procedure of defluorinative coupling using allyltrimethylsilane as nucleophile (eluting with PE/DCM = 50/1). Isolated yield = 72% (20.7 mg); Colorless oil; Rf = 0.3 (PE); **^{1}H NMR** (400 MHz, $CDCl_3$) δ 7.49 – 7.38 (m, 2H), 7.36 – 7.26 (m, 4H), 7.24 – 7.14 (m, 4H), 5.67 (ddt, *J* = 17.2, 10.2, 7.0 Hz, 1H), 5.35 (d, *J* = 39.6 Hz, 1H), 5.09 – 4.85 (m, 2H), 2.69 (dtd, *J* = 10.2, 7.3, 4.4 Hz, 1H), 2.39 (tt, *J* = 7.1, 1.3 Hz, 2H), 2.25 – 1.96 (m, 3H), 1.90 – 1.74 (m, 1H); **^{13}C NMR** (101 MHz, $CDCl_3$) δ 160.8 (d, *J* = 266.6 Hz), 144.2, 136.6, 133.8 (d, *J* = 2.3 Hz), 128.44, 128.37, 128.2 (d, *J* = 7.3 Hz), 127.7, 126.6 (d, *J* = 2.2 Hz), 126.3, 116.2, 105.9 (d, *J* = 8.7 Hz), 45.0, 41.4, 32.2, 31.1 (d, *J* = 26.6 Hz); **^{19}F NMR** (376 MHz, $CDCl_3$) δ -101.09 (ddd, *J* = 37.4, 20.3, 16.3 Hz). **HRMS** (EI, m/z): calcd for $C_{20}H_{21}F$ $[M]^{+\bullet}$ 280.1622, found 280.1624.

Kumada-Coupling Reaction

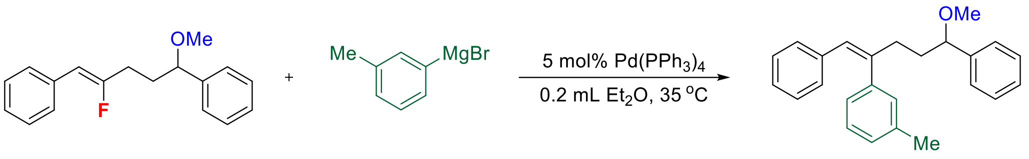

Following the Cao's procedure[27], to a 4 mL vial was added $Pd(PPh_3)_4$ (5.8mg, 0.005 mmol), fluorinated dienes (0.1 mmol), and Et_2O (0.2 mL, dry) in the glove box. To solution was added a Et_2O solution of $3\text{-}MeC_6H_4MgBr$ (0.15 mL, 1.0 M, 0.15 mmol) at room temperature. The vial was removed from the glove box and stirred for 6 h at 35 °C. After completion of the reaction, the reaction mixture was quenched with a saturated aqueous solution of NH_4Cl (2 mL) and extracted with ethyl acetate (3 × 3 mL). The combined organic layer was washed with water and brine, then dried over anhydrous $MgSO_4$, filtered, and concentrated under vacuum. Purification by column chromatography (eluting with PE/DCM = 10/1) on silica gel afforded the desired product **6a** (26.0 mg, 76% yield).

(Z)-(5-methoxy-2-(m-tolyl)pent-1-ene-1,5-diyl)dibenzene (6a)

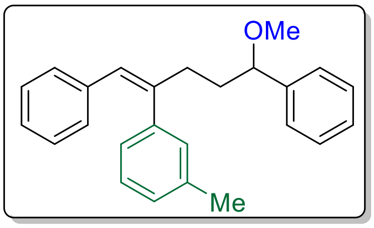

Isolated yield = 76% (26.0 mg); Pale yellow oil; Rf = 0.4 (PE:EA = 100:1); **^{1}H NMR** (400 MHz, $CDCl_3$) δ 7.35 – 7.31 (m, 2H), 7.28 – 7.22 (m, 3H), 7.13 – 6.99 (m, 7H), 6.96 – 6.90 (m, 2H), 6.41 (s, 1H), 4.12 (dd, *J* = 8.1, 5.3 Hz, 1H), 3.20 (s, 3H), 2.65 – 2.45 (m, 2H), 2.33

(s, 3H), 1.89 (dtd, J = 13.8, 8.8, 5.5 Hz, 1H), 1.70 (dddd, J = 14.0, 9.3, 6.6, 5.4 Hz, 1H). **^{13}C NMR** (101 MHz, $CDCl_3$) δ 142.5, 142.2, 137.8, 137.6, 136.5, 129.2, 128.9, 128.4, 128.3, 127.8, 127.5, 126.7, 126.3, 126.0, 83.2, 56.7, 36.8, 36.3, 21.2. **HRMS** (ESI, m/z): calcd for $C_{25}H_{26}O$ $[M+H]^+$ 343.2056, found 343.2063.

Synthesis of Lactone

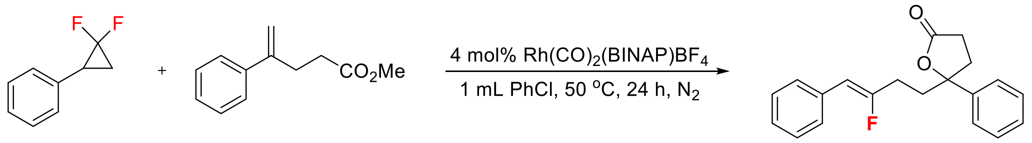

1a, 0.15 mmol **2ac**, 0.1 mmol **7a**, 56%

In a nitrogen filled glove box, a 4 mL vial equipped with a stir bar was charged with $[Rh(CO)_2(BINAP)BF_4]$ (3.5mg, 0.004 mmol, 4 mol%) and PhCl (1 mL). After stirring at room temperature for about 5 min, *gem*-difluorinated cyclopropane **1a** (23.1 mg, 0.15 mmol) and methyl 4-phenylpent-4-enoate **2ac** (19.0 mg, 0.1 mmol) was added to the resulting yellow catalyst solution. The 4 mL vial was sealed and removed from the glove box and stirred at 50 °C for 24 hours. The reaction mixture was cooled to room temperature and purified by chromatography (eluting with PE/EtOAc = 20/1) on silica gel column to give the lactone **7a** in 56% yield.

(Z)-5-(3-fluoro-4-phenylbut-3-en-1-yl)-5-phenyldihydrofuran-2(3H)-one (7a)

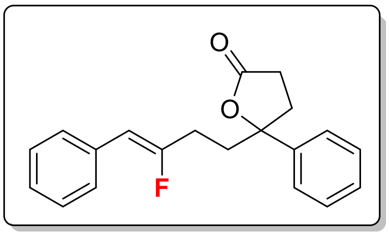

Isolated yield = 56% (17.4 mg); Colorless oil; Rf = 0.2 (PE:EA = 20:1); **^{1}H NMR** (400 MHz, $CDCl_3$) δ 7.46 – 7.26 (m, 9H), 7.19 (tt, J = 6.7, 1.3 Hz, 1H), 5.38 (d, J = 39.3 Hz, 1H), 2.72 – 2.18 (m, 7H), 2.05 (dddd, J = 15.1, 13.4, 10.6, 4.3 Hz, 1H); **^{13}C NMR** (101 MHz, $CDCl_3$) δ 176.3, 159.6 (d, J = 266.3 Hz), 141.8, 133.4, 128.8, 128.4, 128.2 (d, J = 7.4 Hz), 127.9, 126.8 (d, J = 2.3 Hz), 124.5, 106.3 (d, J = 8.6 Hz), 88.5, 39.0, 35.7, 28.4, 28.1 (d, J = 27.0 Hz); **^{19}F NMR** (376 MHz, $CDCl_3$) δ -102.08 (dt, J = 39.6, 17.6 Hz). **HRMS** (ESI, m/z): calcd for $C_{20}H_{19}FO_2$ $[M+H]^+$ 311.1442, found 311.1448.

8. The Effect of Feeding Sequence for Rhodium Catalyst Generated In-Situ

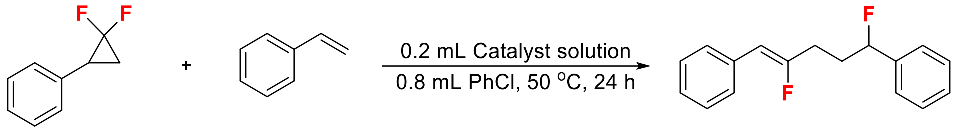

1a, 0.15 mmol **2a**, 0.10 mmol **3a**

$[Rh(CO)_2Cl]_2$ + *rac*-BINAP + $AgBF_4$ —(PhCl, rt., N_2)→ Catalyst solution

Entry	Pre-mixing method	conversion of **2a**	Yield of **3a**
1	A	100%	<5%
2	B	60%	40%
3	C	90%	80%
4[a]	-	99%	92%

[a]$Rh(CO)_2(BINAP)BF_4$ instead of the catalyst solution

Supplementary Table 2. Influence of feeding sequence for rhodium catalyst generated in-situ on the reaction

Method A: In a nitrogen filled glove box, a 4 mL vial equipped with stir bar was charged with $[Rh(CO)_2Cl]_2$ (0.8 mg, 0.002 mmol), *rac*-BINAP (0.004 mmol, 2.5 mg), $AgBF_4$ (0.005 mmol, 1 mg), and PhCl (1 mL). This solution was stirred for about 10 min, and then to this solution was added styrene **2a** (0.1 mmol, 10.4 mg) and *gem*-difluorinated cyclopropane **1a** (0.15 mmol, 23.1 mg). The 4 mL vial was sealed and removed from the glove box and stirred at 50 °C for 24 hours and this reaction mixture was analyzed by TLC analysis and ^{1}H NMR spectroscopy.

Method B: In a nitrogen filled glove box, an 8 mL vial equipped with stir bar was charged with $[Rh(CO)_2Cl]_2$ (7.8 mg, 0.02 mmol), *rac*-BINAP (0.004 mmol, 24.9 mg), $AgBF_4$ (0.05 mmol, 9.7 mg), and PhCl (2 mL). This solution was stirred for about 30 min, which afforded a yellow heterogeneous solution, and then transferred the catalyst solution into another vial with stir bar, styrene **2a** (0.1 mmol, 10.4 mg), and *gem*-difluorinated cyclopropane **1a** (0.15 mmol, 23.1 mg). The 4 mL vial was sealed and removed from the glove box and stirred at 50 °C for 24 hours and this reaction mixture was analyzed by TLC analysis and ^{1}H NMR spectroscopy.

Method C: In a nitrogen filled glove box, an 8 mL vial equipped with stir bar was charged with $[Rh(CO)_2Cl]_2$ (7.8 mg, 0.02 mmol), $AgBF_4$ (0.05 mmol, 9.7 mg), and PhCl (2 mL). After stirring for 10 min, to this mixture was added *rac*-BINAP (0.04 mmol, 24.9 mg). This mixture was stirred for 30 min, which afforded a yellow heterogeneous solution, and then transferred the catalyst solution into

another vial with stir bar, styrene **2a** (0.1 mmol, 10.4 mg), and *gem*-difluorinated cyclopropane **1a** (0.15 mmol, 23.1 mg). The 4 mL vial was sealed and removed from the glove box and stirred at 50 °C for 24 hours and this reaction mixture was analyzed by TLC analysis and ^{1}H NMR spectroscopy.

9. Mechanism Experiments

9.1 The evidence for process of carbon cation

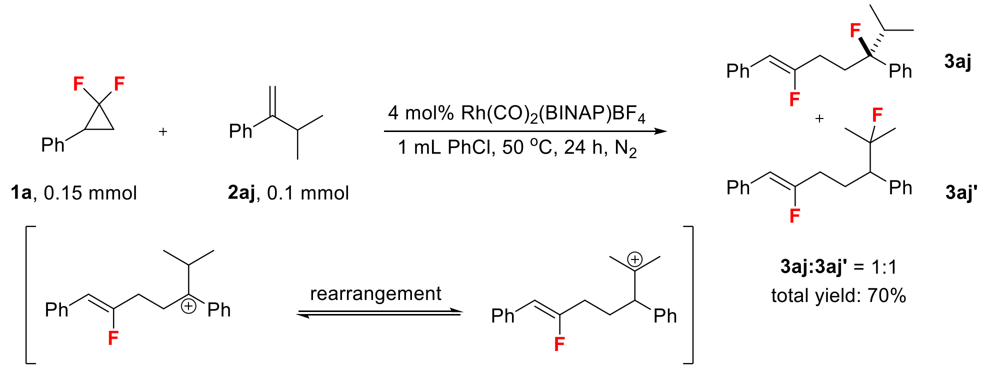

(3-methylbut-1-en-2-yl)Benzene **2aj** was employed in this reaction under the standard reaction conditions, providing the benzyl fluorides **3aj** and tertiary alkyl fluoride **3aj'**. This suggests the involvement of a carbon cation rearrangement in the reaction process.

(Z)-(2,5-difluoro-6-methylhept-1-ene-1,5-diyl)dibenzene (3aj)

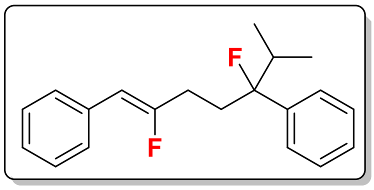

Isolated yield = 35% (10.3 mg); Colorless oil; Rf = 0.2 (PE). **^{1}H NMR** (400 MHz, $CDCl_3$)) δ 7.44 – 7.34 (m, 4H), 7.31 – 7.25 (m, 5H), 7.21 – 7.14 (m, 1H), 5.37 (d, J = 39.4 Hz, 1H), 2.45 – 1.91 (m, 5H), 0.90 (dd, J = 71.0, 6.9 Hz, 6H). **^{13}C NMR** (101 MHz, $CDCl_3$)) δ 160.8 (d, J = 266.1 Hz), 141.3 (d, J = 22.3 Hz), 133.8 (d, J = 2.6 Hz), 128.5, 128.3 (d, J = 7.3 Hz), 128.2 (d, J = 2.0 Hz), 127.2, 126.8 (d, J = 2.3 Hz), 105.9 (d, J = 8.6 Hz), 101.4 (d, J = 179.7 Hz), 37.7 (d, J = 23.6 Hz), 34.3 (d, J = 23.1 Hz), 27.9 (dd, J = 26.9, 4.3 Hz), 17.5 (d, J = 5.8 Hz), 16.7 (d, J = 4.1 Hz). **^{19}F NMR** (376 MHz, $CDCl_3$)) δ -101.29 (dt, J = 37.2, 18.0 Hz), -170.53 (ddd, J = 33.4, 21.5, 10.3 Hz). **HRMS** (EI, m/z): calcd for $C_{20}H_{22}F_2$ $[M]^{+\bullet}$ 300.1684, found 300.1691.

(Z)-(2,6-difluoro-6-methylhept-1-ene-1,5-diyl)dibenzene (3aj')

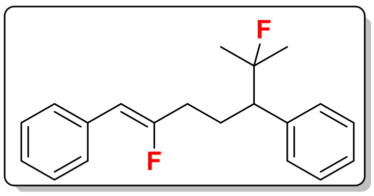

Isolated yield = 35% (10.6 mg); Colorless oil; Rf = 0.25 (PE). **^{1}H NMR** (400 MHz, $CDCl_3$) δ 7.43 (dd, *J* = 8.4, 1.4 Hz, 2H), 7.35 – 7.25 (m, 5H), 7.25 – 7.17 (m, 3H), 5.32 (d, *J* = 39.5 Hz, 1H), 2.79 (ddd, *J* = 18.0, 11.6, 3.3 Hz, 1H), 2.36 – 2.24 (m, 1H), 2.24 – 2.14 (m, 1H), 2.11 – 1.95 (m, 2H), 1.30 (dd, *J* = 24.1, 21.8 Hz, 6H). **^{13}C NMR** (101 MHz, $CDCl_3$) δ 160.4 (d, *J* = 266.6 Hz), 139.9 (d, *J* = 4.9 Hz), 133.7 (d, *J* = 2.6 Hz), 129.4, 128.4, 128.3, 128.2 (d, *J* = 7.6 Hz), 127.0, 126.7 (d, *J* = 2.2 Hz), 106.2 (d, *J* = 8.7 Hz), 97.1 (d, *J* = 171.1 Hz), 54.5 (d, *J* = 21.9 Hz), 31.3 (d, *J* = 26.5 Hz), 26.5 (d, *J* = 24.4 Hz), 25.8 (d, *J* = 4.5 Hz), 24.6 (d, *J* = 24.9 Hz). **^{19}F NMR** (376 MHz, $CDCl_3$) δ -101.65 (ddd, *J* = 37.2, 22.3, 13.9 Hz), -141.50 (dq, *J* = 42.8, 21.5 Hz). **HRMS** (EI, m/z): calcd for $C_{20}H_{22}F_2$ $[M]^{+\bullet}$ 300.1684, found 300.1687.

9.2 The effect of counter-anions

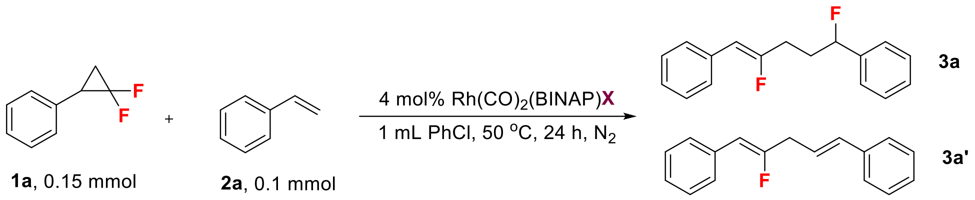

entry	anion **X**	yield of **3a**	yield of **3a'**	conversion of **1a**
1	BF_4	92% (88% isolated)	0	75%
2	PF_6	8%	trace	<20%
3	SbF_6	< 5%	trace	<20%
4	NTf_2	< 5%	13%	<30%
5	OTf	0	trace	<10%

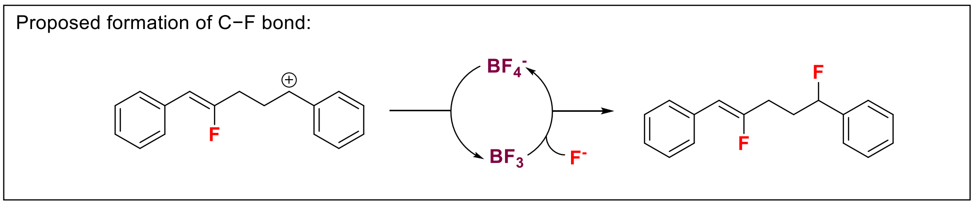

Supplementary Table 3. The effect of counter-anions

We synthesized some dicarbonyl rhodium catalysts with different anions and then investigated the effect of the anions. As shown in **Supplementary Table 3**, this reaction can efficiently generate product **3a** only when BF_4^- was used as an anion. Other anions, such as PF_6^-, SbF^-, NTf_2^-, and OTf^-, were found to be much less effective, leading to low conversion. Inspired by the use of stoichiometric BF_4^- as nucleophilic fluoride donors, we infer that the addition of fluorine to alkenes is mediated by BF_4^-, which acts as a fluorine anion shuttle[28].

10. X-ray Crystallographic Data

Supplementary Table 4. Crystal data and structure refinement for 3g, 3w and rhodium catalyst.

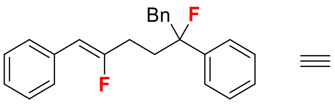

3g

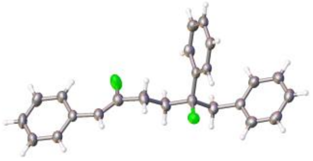

CCDC: 2307401

Identification code	**3g**
Empirical formula	$C_{24}H_{22}F_2$
Formula weight	348.41
Temperature/K	253.0
Crystal system	monoclinic
Space group	$P2_1/c$
a/Å	13.473(2)
b/Å	5.7673(10)
c/Å	23.840(4)
α/°	90
β/°	95.954(6)
γ/°	90
Volume/Å^3	1842.4(5)
Z	4
ρ_{calc}g/cm^3	1.256
μ/mm^{-1}	0.085
F(000)	736.0
Crystal size/mm^3	0.47 × 0.11 × 0.09
Radiation	MoKα (λ = 0.71073)
2Θ range for data collection/°	4.344 to 55.154
Index ranges	$-17 \le h \le 17$, $-6 \le k \le 7$, $-31 \le l \le 28$

Reflections collected	12501
Independent reflections	4240 [R_{int} = 0.0746, R_{sigma} = 0.0870]
Data/restraints/parameters	4240/0/235
Goodness-of-fit on F^2	1.048
Final R indexes [I>=2σ (I)]	R_1 = 0.0584, wR_2 = 0.1400
Final R indexes [all data]	R_1 = 0.0887, wR_2 = 0.1638
Largest diff. peak/hole / e Å^{-3}	0.33/-0.25

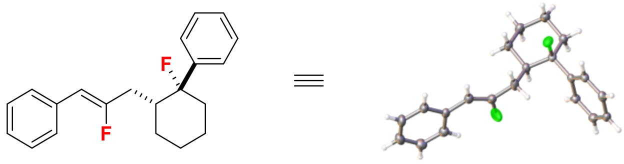

3w (±) CCDC: 2340552

Identification code	**3w**
Empirical formula	$C_{21}H_{22}F_2$
Formula weight	312.38
Temperature/K	150.0
Crystal system	monoclinic
Space group	$P2_1/c$
a/Å	16.8610(15)
b/Å	5.5579(4)
c/Å	17.7291(16)
α/°	90
β/°	96.257(3)
γ/°	90
Volume/Å^3	1651.5(2)
Z	4
ρ_{calc}g/cm^3	1.256
μ/mm^{-1}	0.087

F(000)	664.0
Crystal size/mm^3	0.43 × 0.27 × 0.25
Radiation	MoKα (λ = 0.71073)
2Θ range for data collection/°	4.622 to 55.012
Index ranges	$-21 \leq h \leq 21$, $-6 \leq k \leq 7$, $-23 \leq l \leq 23$
Reflections collected	24746
Independent reflections	3787 [R_{int} = 0.0712, R_{sigma} = 0.0426]
Data/restraints/parameters	3787/0/208
Goodness-of-fit on F^2	1.036
Final R indexes [I>=2σ (I)]	R_1 = 0.0439, wR_2 = 0.1002
Final R indexes [all data]	R_1 = 0.0669, wR_2 = 0.1113
Largest diff. peak/hole / e Å^{-3}	0.20/-0.28

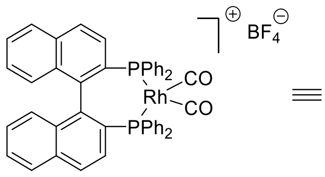

Rhodium catalyst

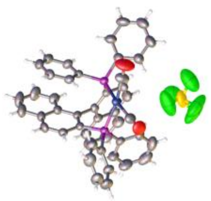

CCDC: 2307402

Identification code	Rhodium catalyst
Empirical formula	$C_{46}H_{32}BF_4O_2P_2Rh$
Formula weight	868.37
Temperature/K	301.0
Crystal system	monoclinic
Space group	$P2_1/n$
a/Å	12.320(2)
b/Å	20.731(4)
c/Å	16.881(3)
α/°	90

β/°	92.800(6)
γ/°	90
Volume/Å^3	4306.3(13)
Z	4
ρ_{calc}g/cm^3	1.339
μ/mm^{-1}	0.524
F(000)	1760.0
Crystal size/mm^3	0.27 × 0.11 × 0.05
Radiation	MoKα (λ = 0.71073)
2Θ range for data collection/°	3.85 to 50
Index ranges	-14 ≤ h ≤ 14, -24 ≤ k ≤ 24, -20 ≤ l ≤ 19
Reflections collected	42621
Independent reflections	7582 [R_{int} = 0.0946, R_{sigma} = 0.0605]
Data/restraints/parameters	7582/6/509
Goodness-of-fit on F^2	1.040
Final R indexes [I>=2σ (I)]	R_1 = 0.0586, wR_2 = 0.1617
Final R indexes [all data]	R_1 = 0.0891, wR_2 = 0.1884
Largest diff. peak/hole / e Å^{-3}	0.80/-0.52

11. Computational Details and Additional Computational Results

11.1 Computational details.

All calculations were performed with Gaussian 16[29]. Geometry optimizations were performed in the gas phase with the B3LYP functional and a mixed basis set of LANL2DZ for Rh and 6-31G(d) for other atoms. Single point energies were calculated with the M06 functional[30] and a mixed basis set of SDD for Rh and 6-311+G(d,p) for other atoms. Solvation energy corrections were calculated using the SMD model[31] with PhCl as the solvent. The same level of theory was used in our previous computational studies of Rh-catalyzed C–C bond activations[32-34].

11.2 Possible active Rh(I) catalyst species.

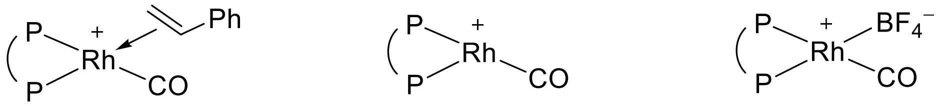

Int1 $\Delta\Delta G$ = 0.0 kcal/mol

Int1-1 $\Delta\Delta G$ = 6.2 kcal/mol

Int1-2 $\Delta\Delta G$ = 1.8 kcal/mol

11.3 Fluorination of the benzylic carbocation with BF_4 anion

The transition state of benzylic carbocation fluorination with BF_4^- cannot be computationally located. As shown below, when scanning the C···F distance from 2.86 Å in structure **C** to 1.46 Å in structure **A**, the energy is continuously declined. This indicates the fluorination of benzylic carbocation with BF_4^- is a barrierless process.

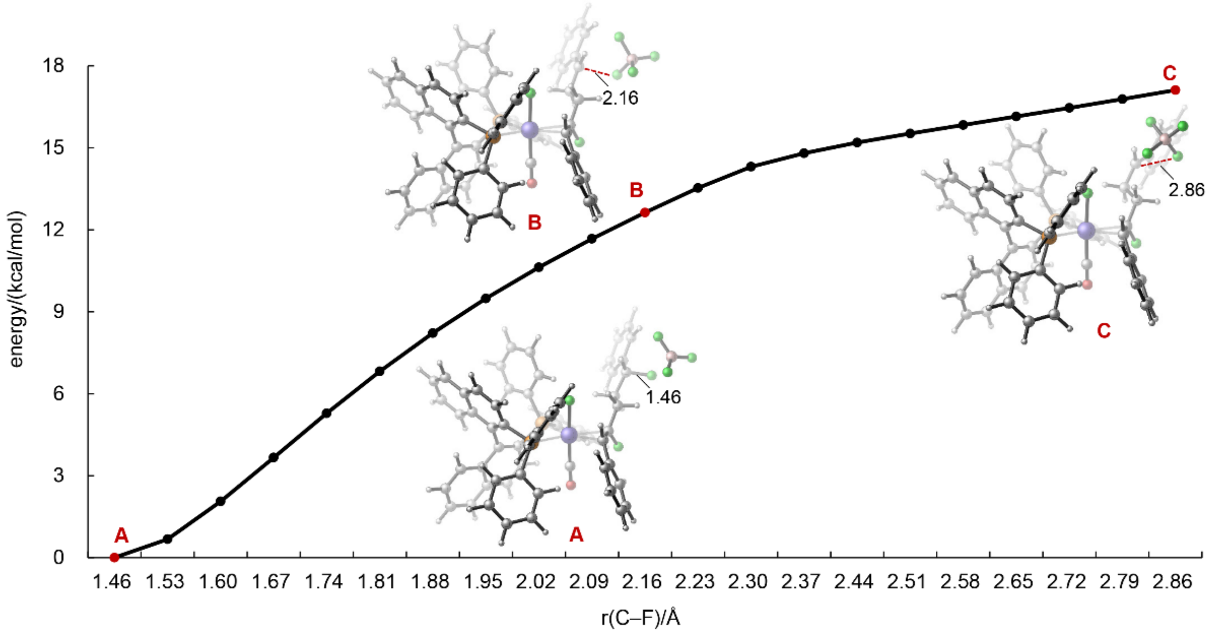

Supplementary Figure 9. The energy scanning for fluorination of the benzylic carbocation with

BF_4 anion

11.4 Possible Rh(III)$^{2+}$-mediated pathways

We also considered the pathway of fluoride elimination with BF_3 and the subsequent C–C formation process. As shown below, the formation of Rh(III)$^{2+}$ complex via fluoride abstraction by BF_3 is thermodynamically accessible, only uphill by 1.9 kcal/mol. However, even mediated by this Rh(III)$^{2+}$ complex (**Int9**), the outer-sphere nucleophilic attack (**TS5**) is still superior to migratory insertion (**TS6**). Although the Rh center in **Int9** has an empty site for styrene insertion, the allyl moieties in **TS6** still adopts η^1 geometry to release steric repulsions with the bidentate phosphine ligand, thus leading to a higher barrier. Compared with the outer-sphere nucleophilic attack (**TS4**, please refer to Fig. 6 in main text) and migratory insertion (**TS3**, please refer to Fig. 6 in main text) mediated by the Rh(III)$^{+}$ complex, these transition states with the Rh(III)$^{2+}$ complex (**TS5** and **TS6**) require lower barriers. However, the feasibility of this alternative Rh(III)$^{2+}$-mediated pathway depends on the concentration of free BF_3 in the experimental condition. Because BF_3 is *in situ* formed in the Rh^{+} pathway and consumed to complete the catalytic cycle, the Rh(III)$^{2+}$ species generated from fluoride abstraction by BF_3 is less possible.

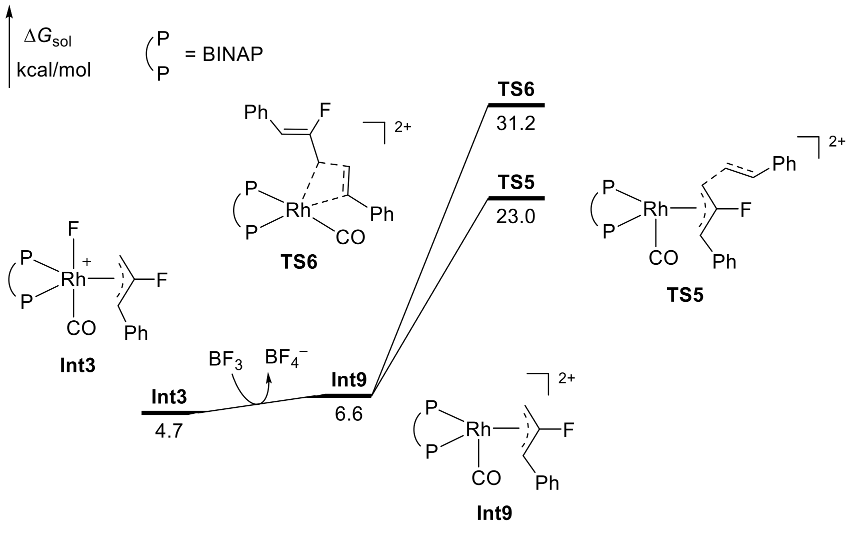

Supplementary Figure 10. Energy profile for Rh(III)$^{2+}$-mediated pathways

12. NMR Spectra

^{1}H NMR (400 MHz, CDCl3) spectrum of 3a

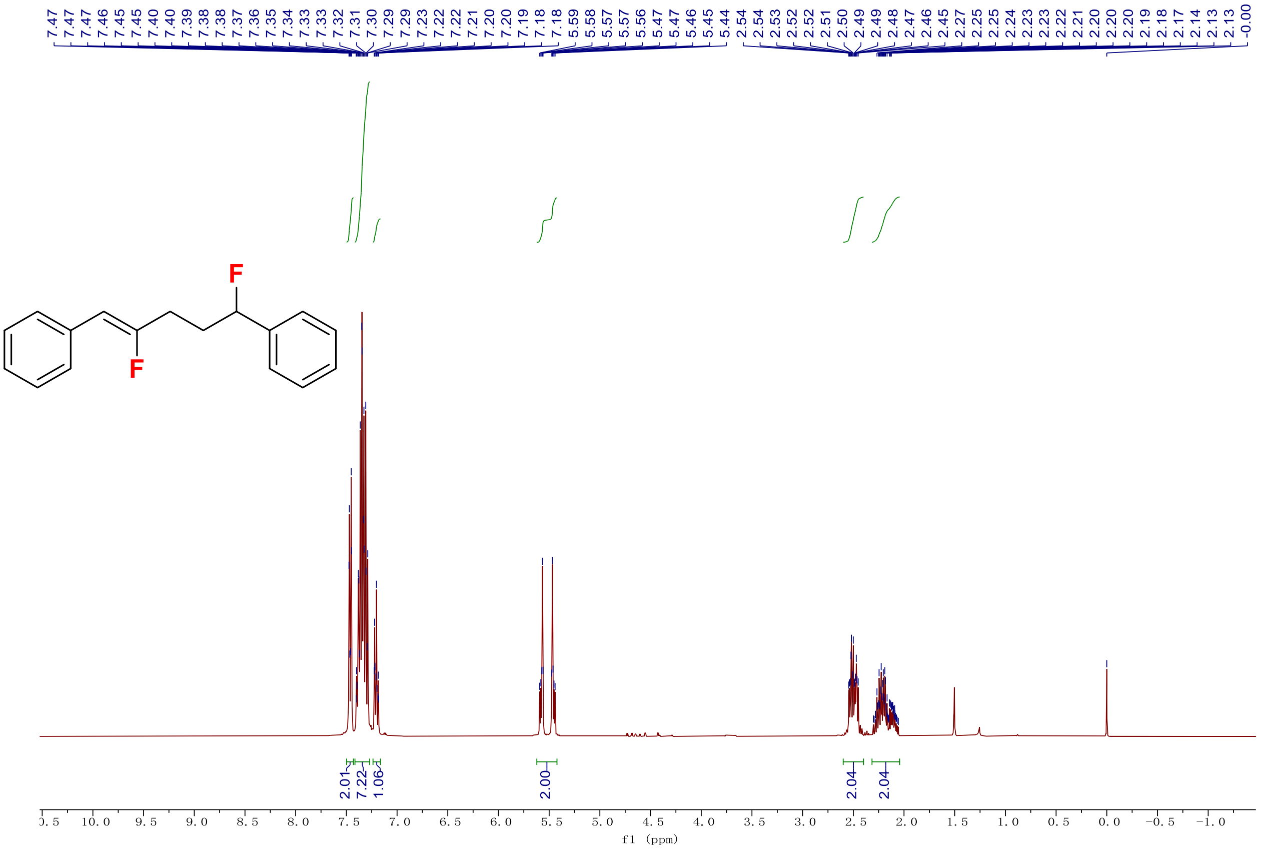

^{13}C NMR (101 MHz, CDCl3) spectrum of 3a

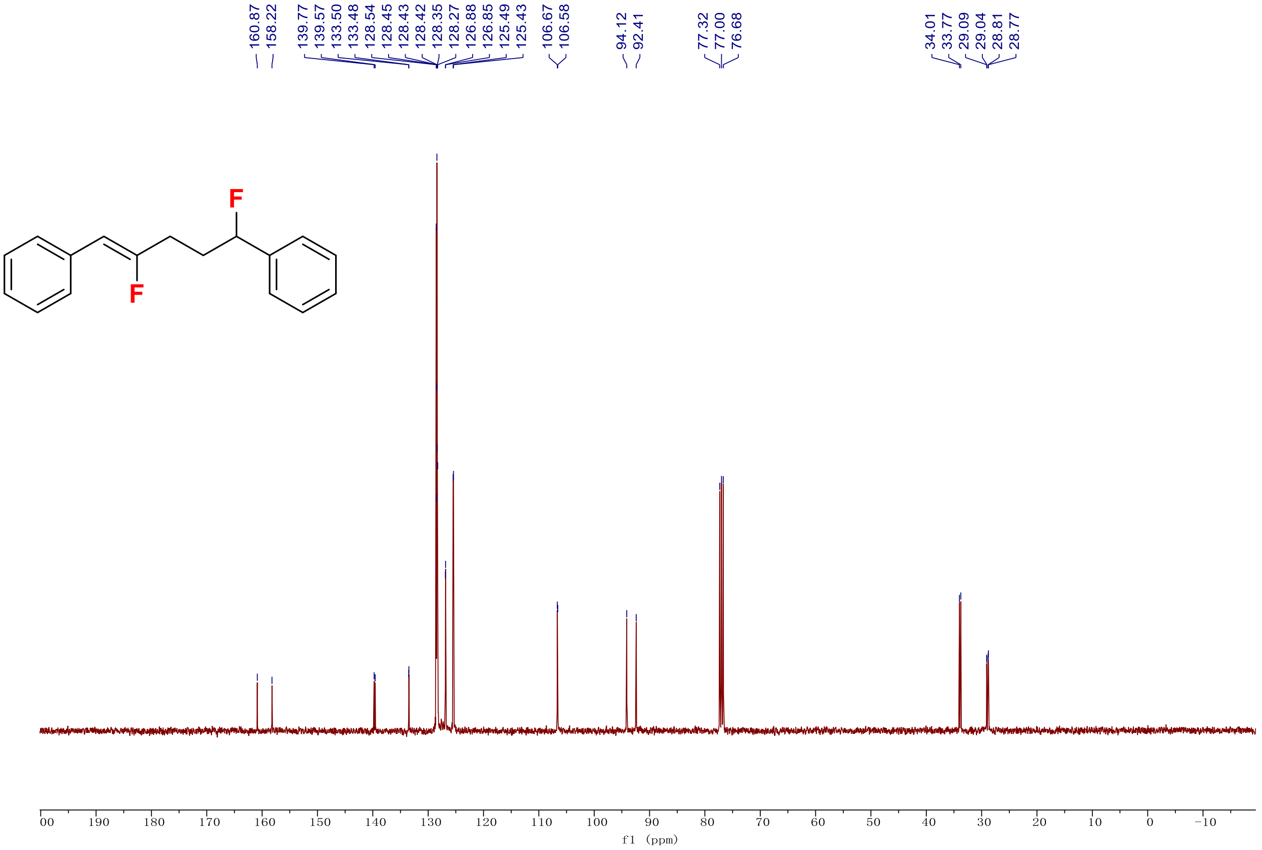

^{19}F NMR (376 MHz, $CDCl_3$) spectrum of 3a

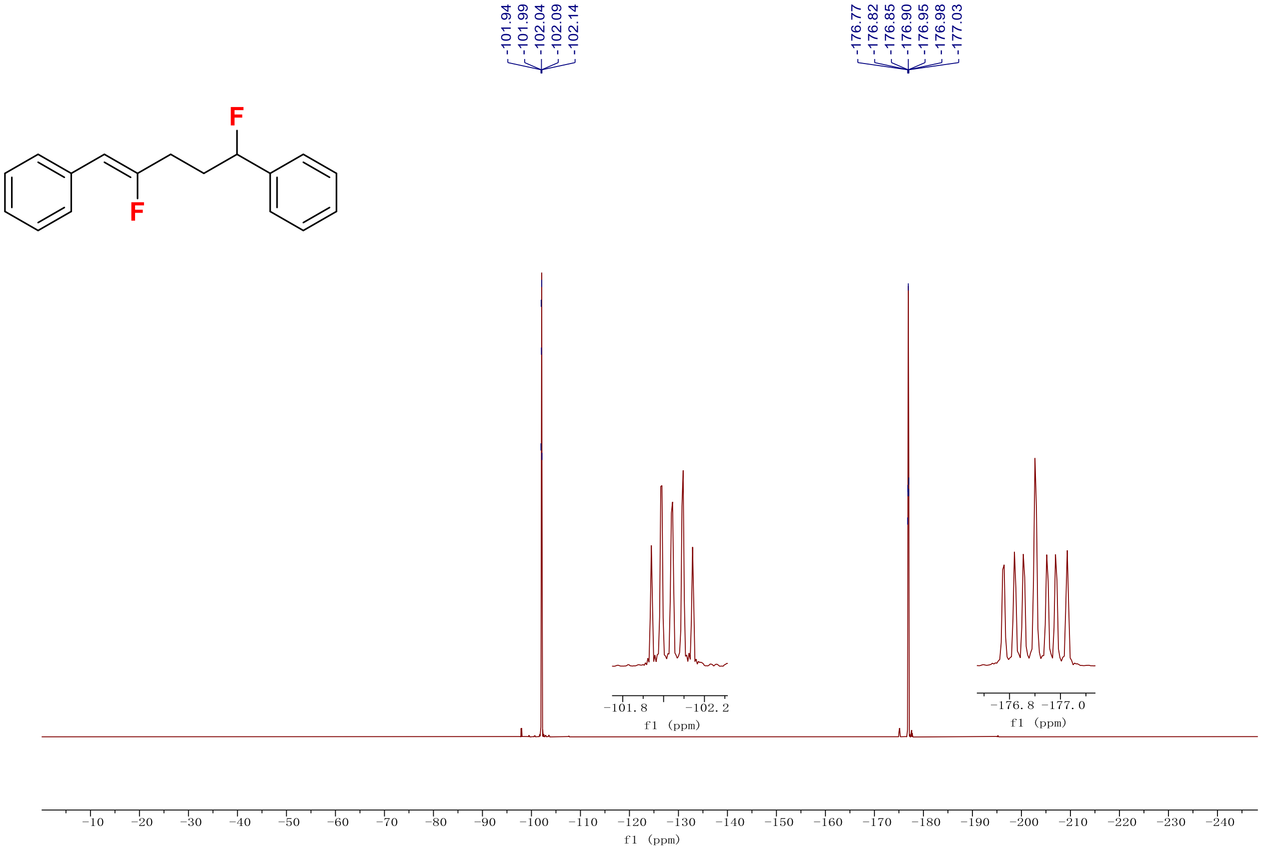

^{1}H NMR (400 MHz, $CDCl_3$) spectrum of 3b

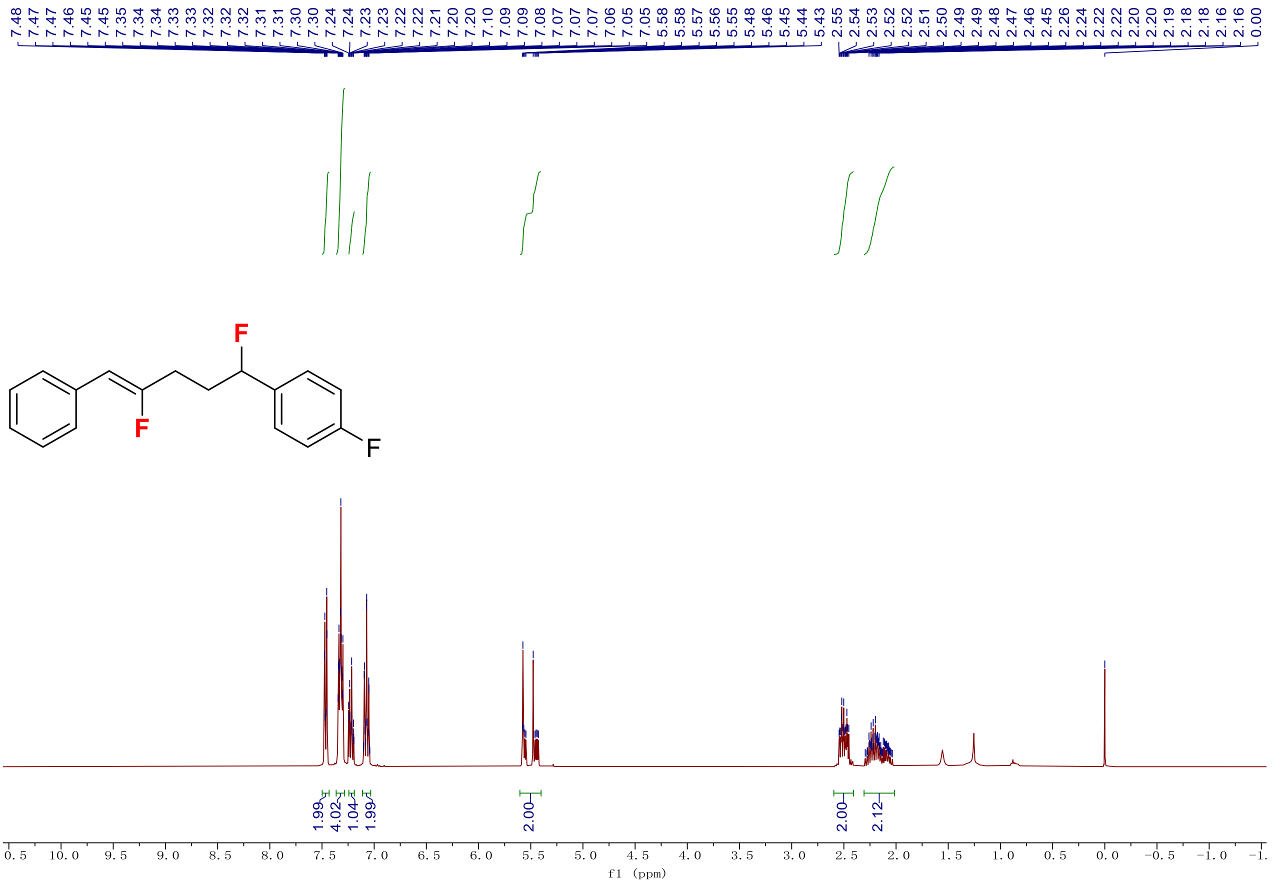

^{13}C NMR (101 MHz, $CDCl_3$) spectrum of 3b

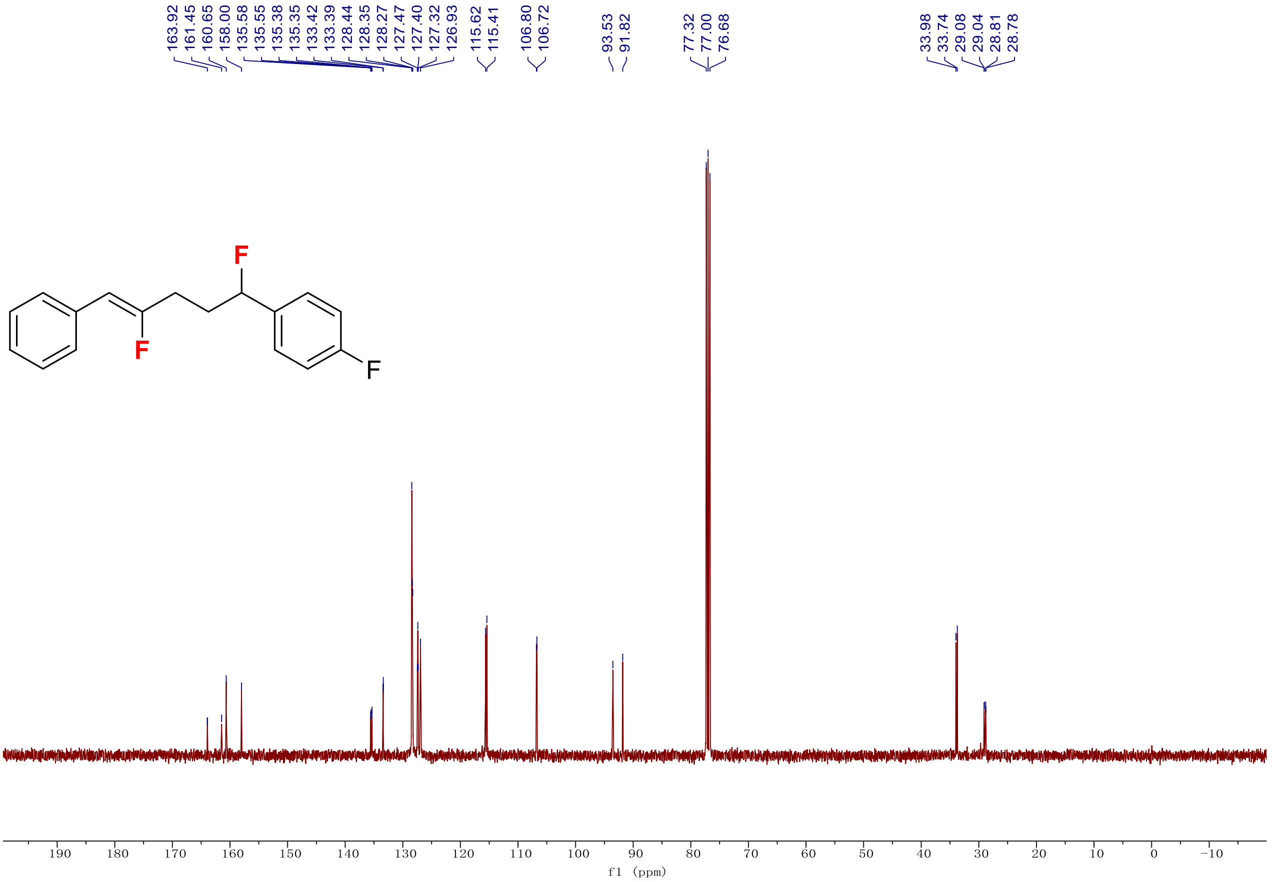

^{19}F NMR (376 MHz, $CDCl_3$) spectrum of 3b

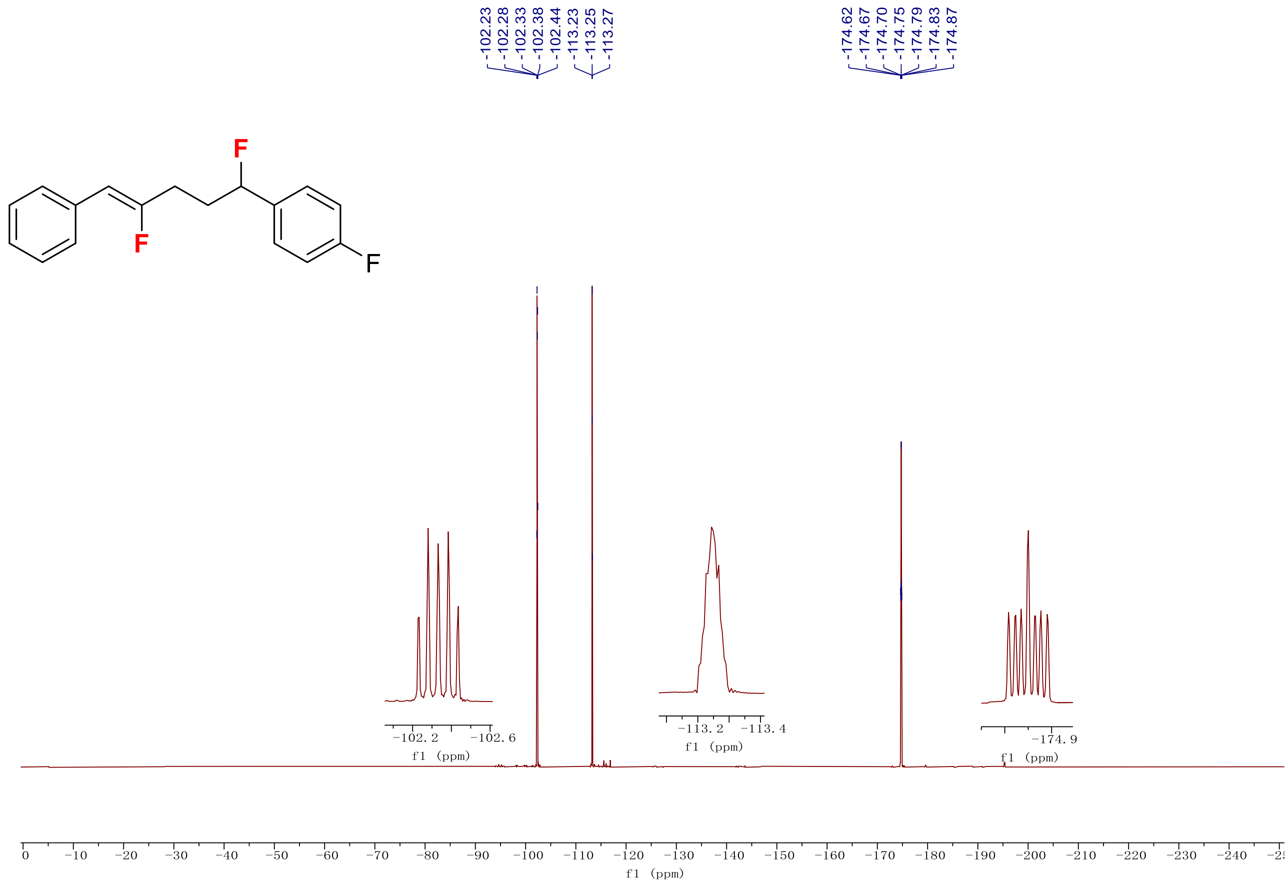

^{1}H NMR (400 MHz, CDCl3) spectrum of 3c

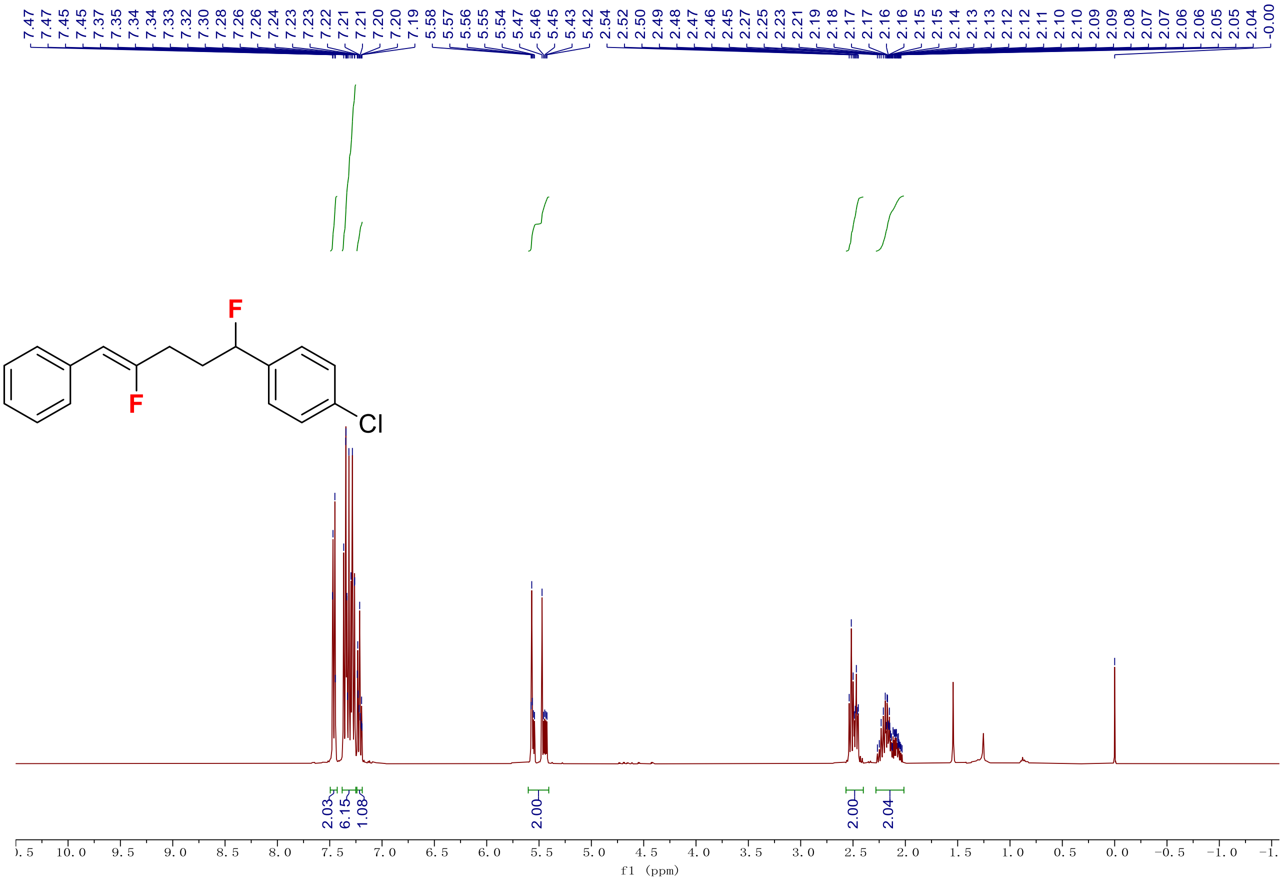

^{13}C NMR (101 MHz, CDCl3) spectrum of 3c

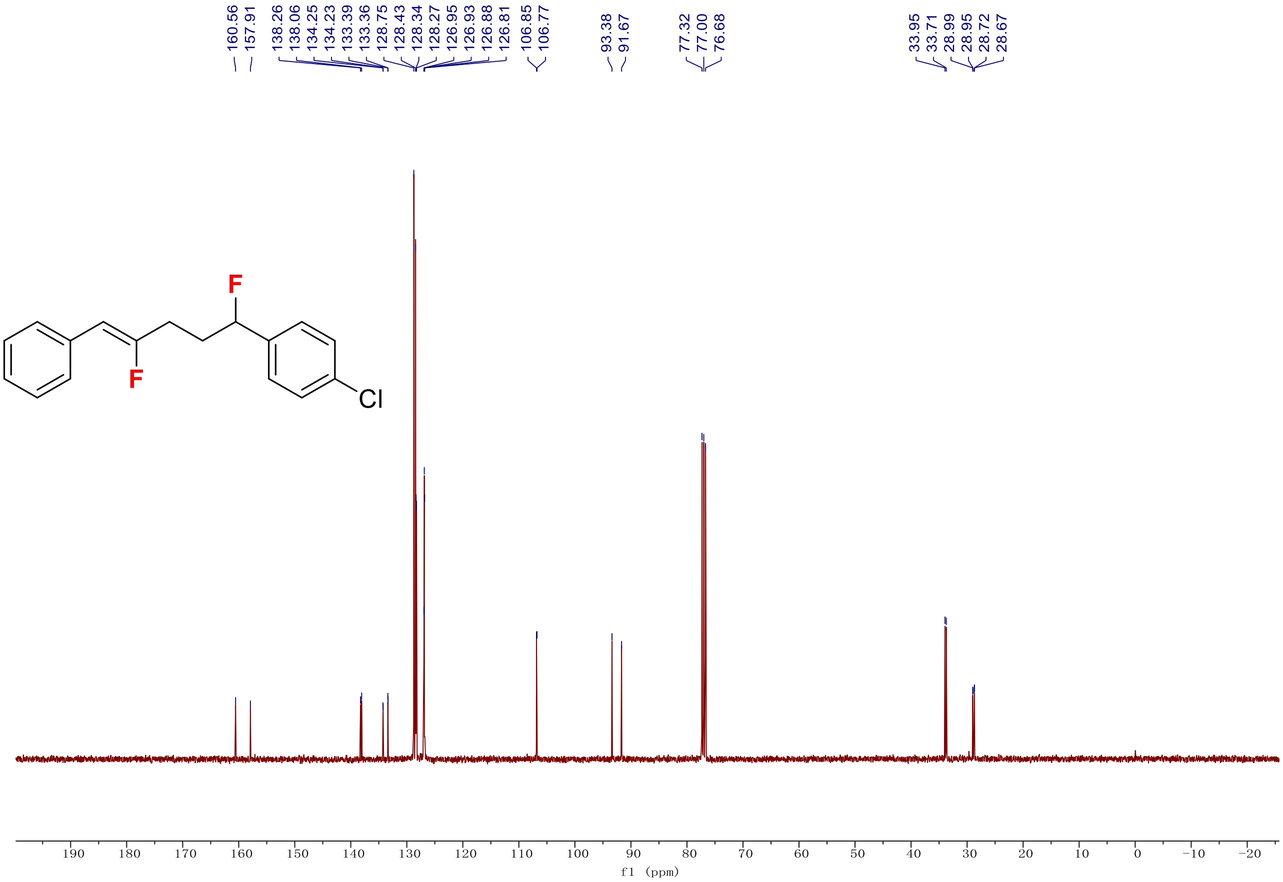

^{19}F NMR (376 MHz, $CDCl_3$) spectrum of 3c

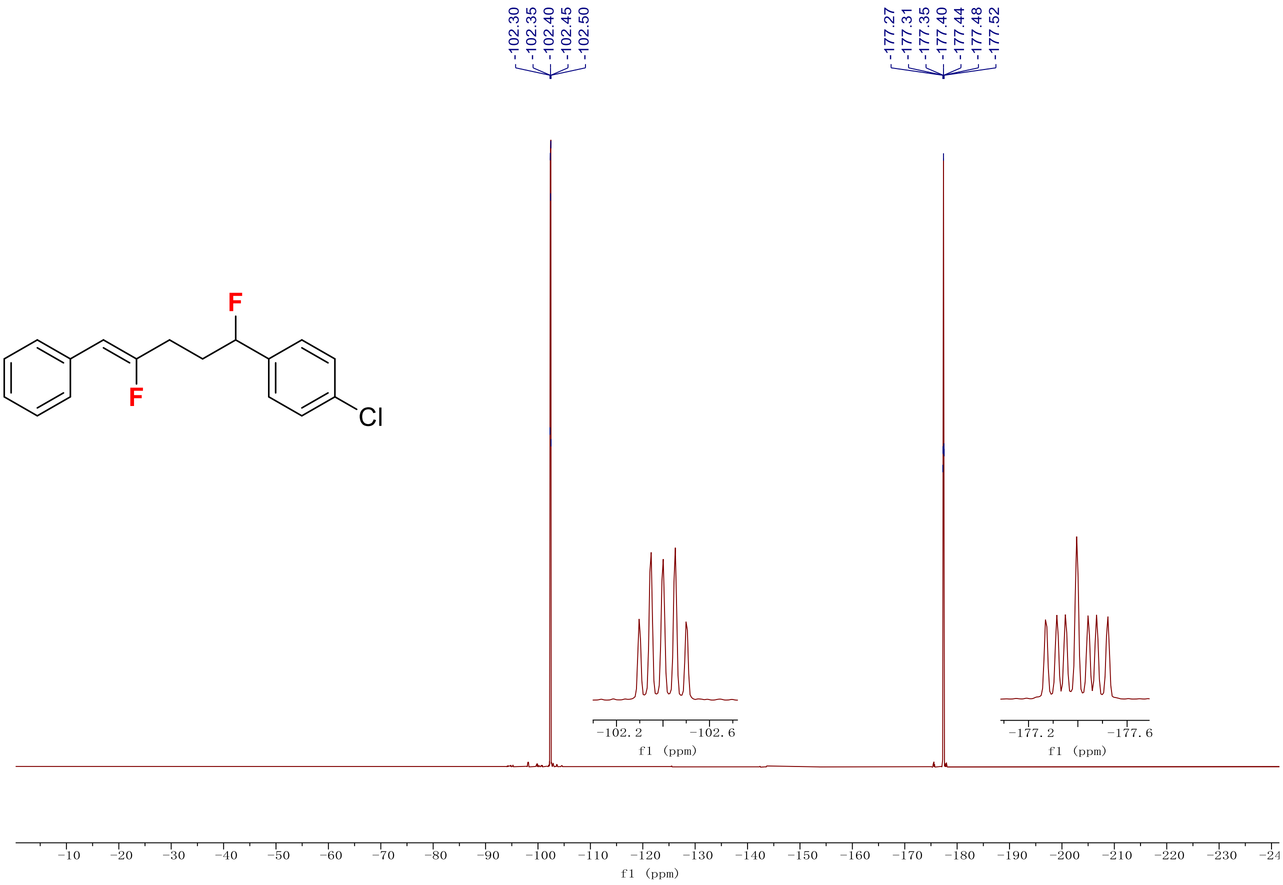

^{1}H NMR (400 MHz, CDCl3) spectrum of 3d

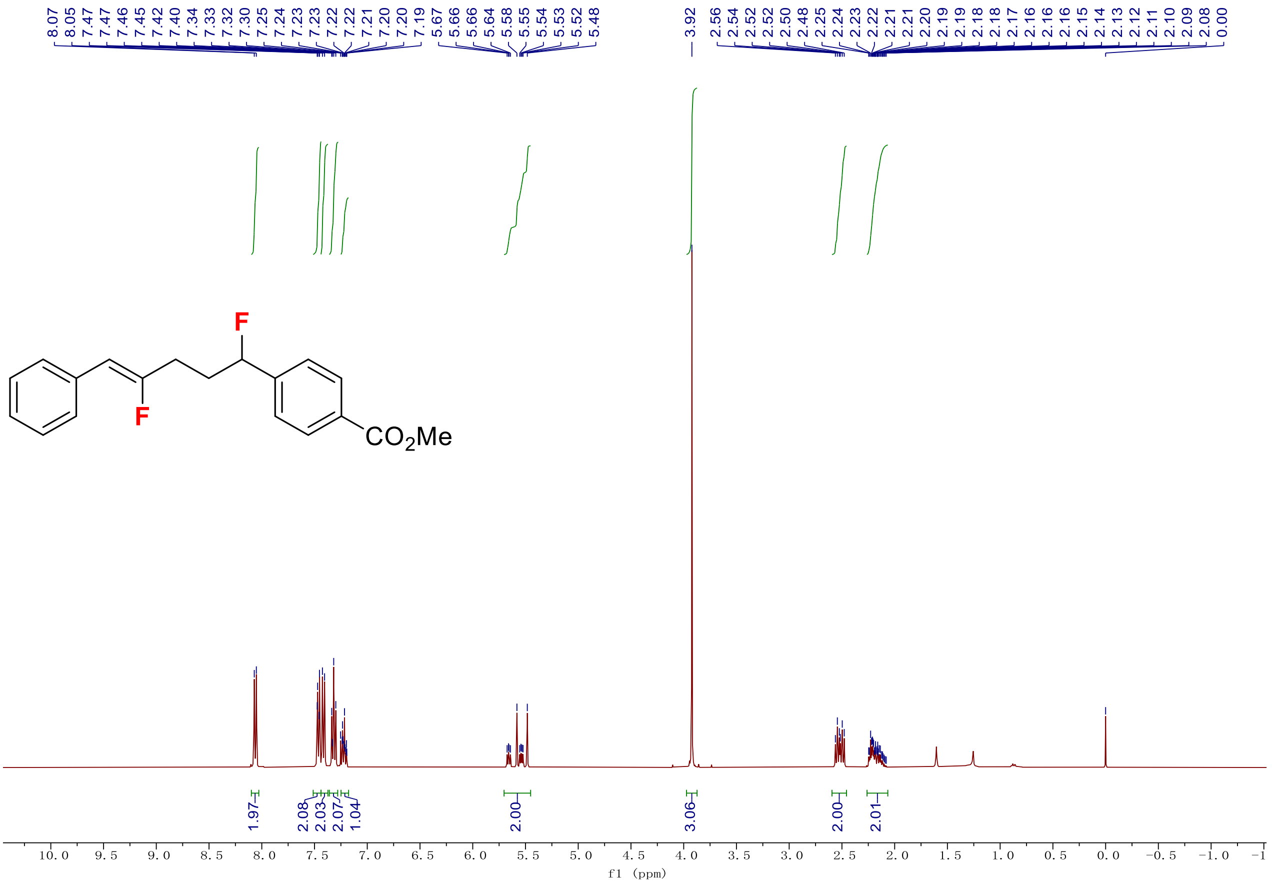

^{13}C NMR (101 MHz, CDCl3) spectrum of 3d

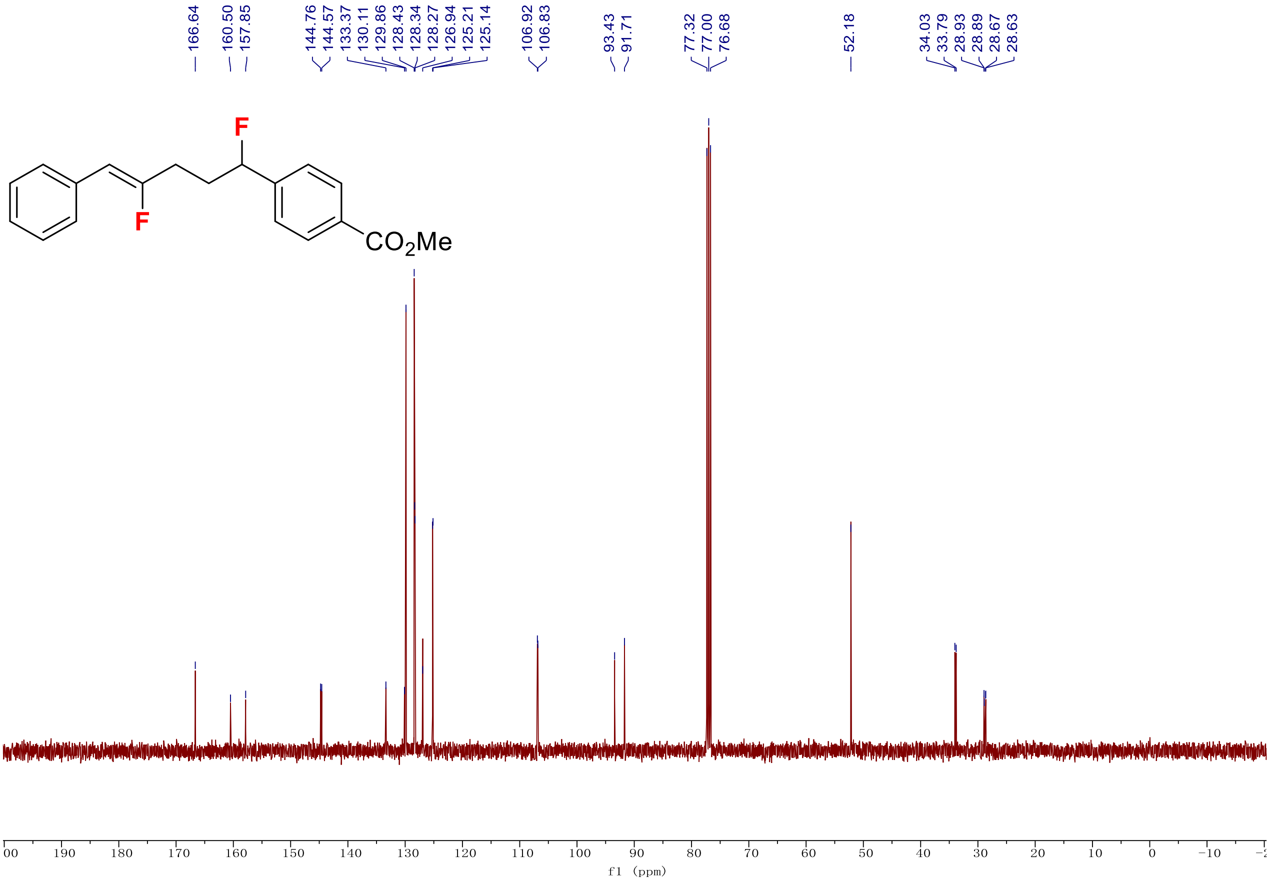

^{19}F NMR (376 MHz, CDCl3) spectrum of 3d

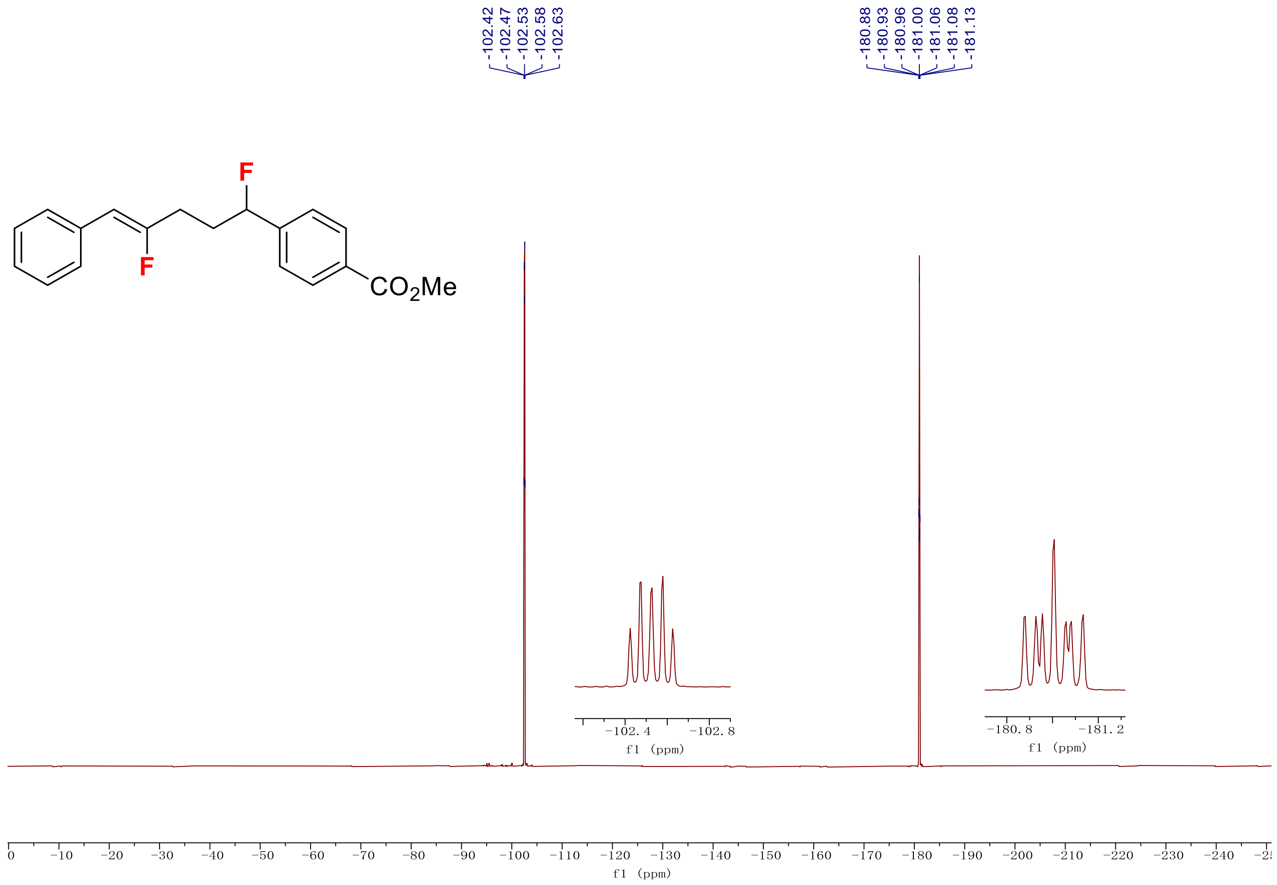

^{1}H NMR (400 MHz, $CDCl_3$) spectrum of 3e

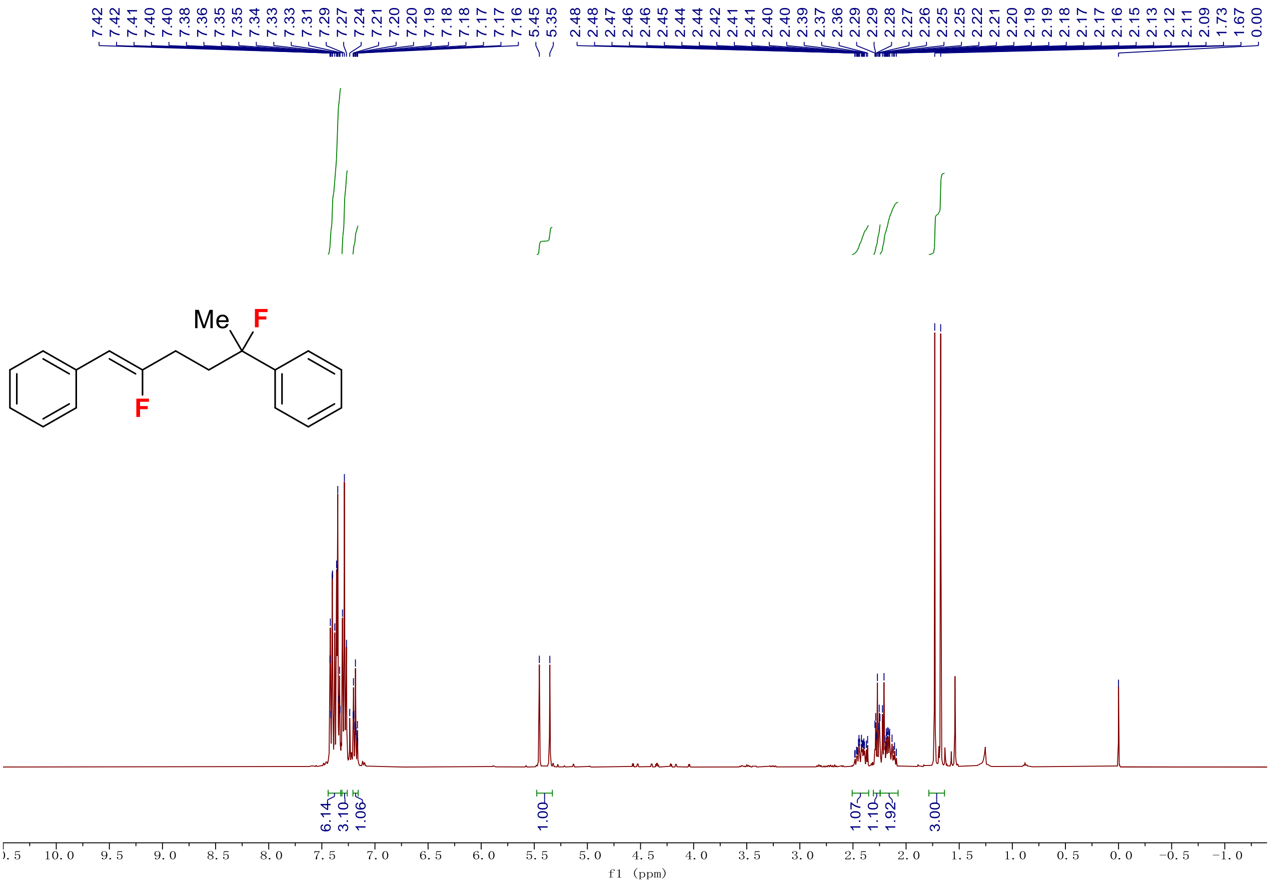

^{13}C NMR (101 MHz, $CDCl_3$) spectrum of 3e

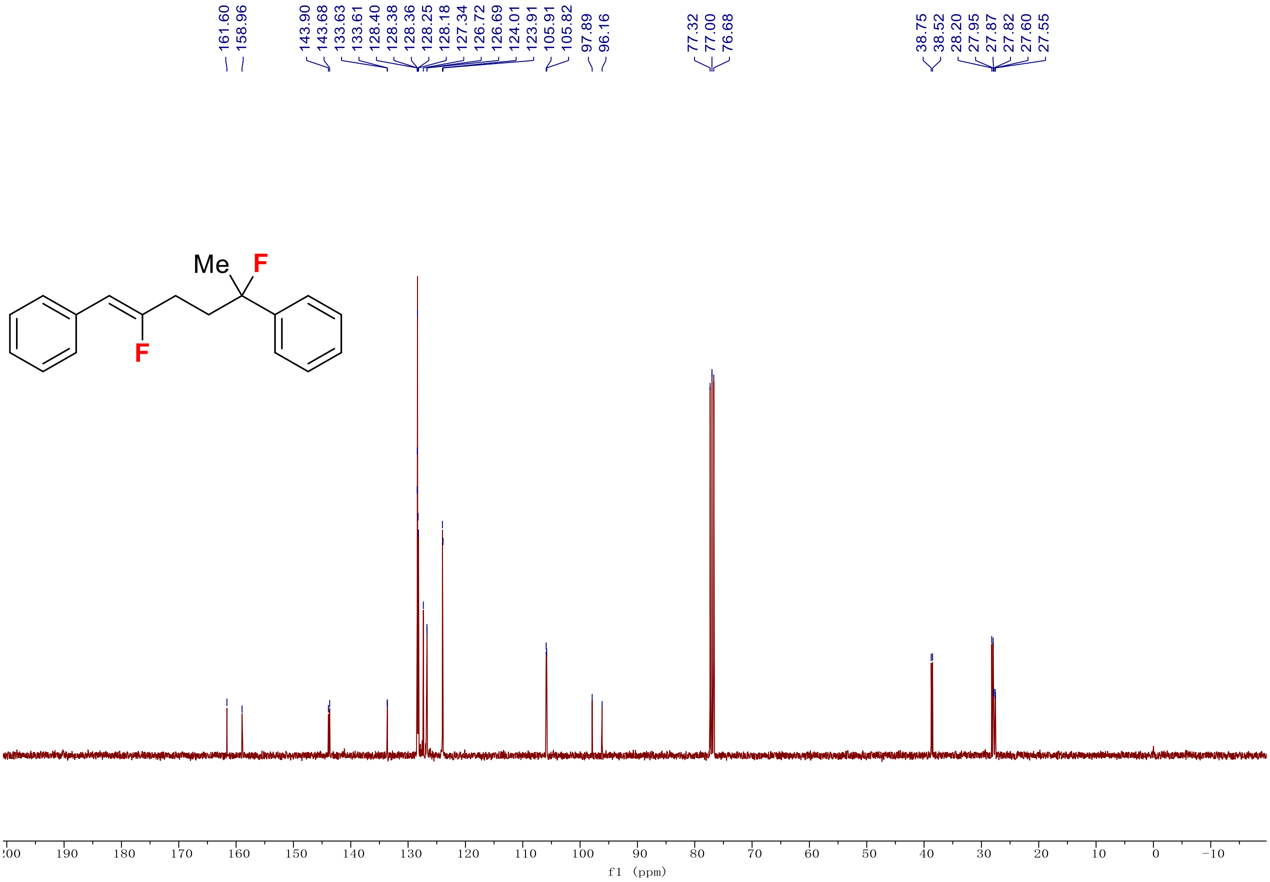

^{19}F NMR (376 MHz, $CDCl_3$) spectrum of 3e

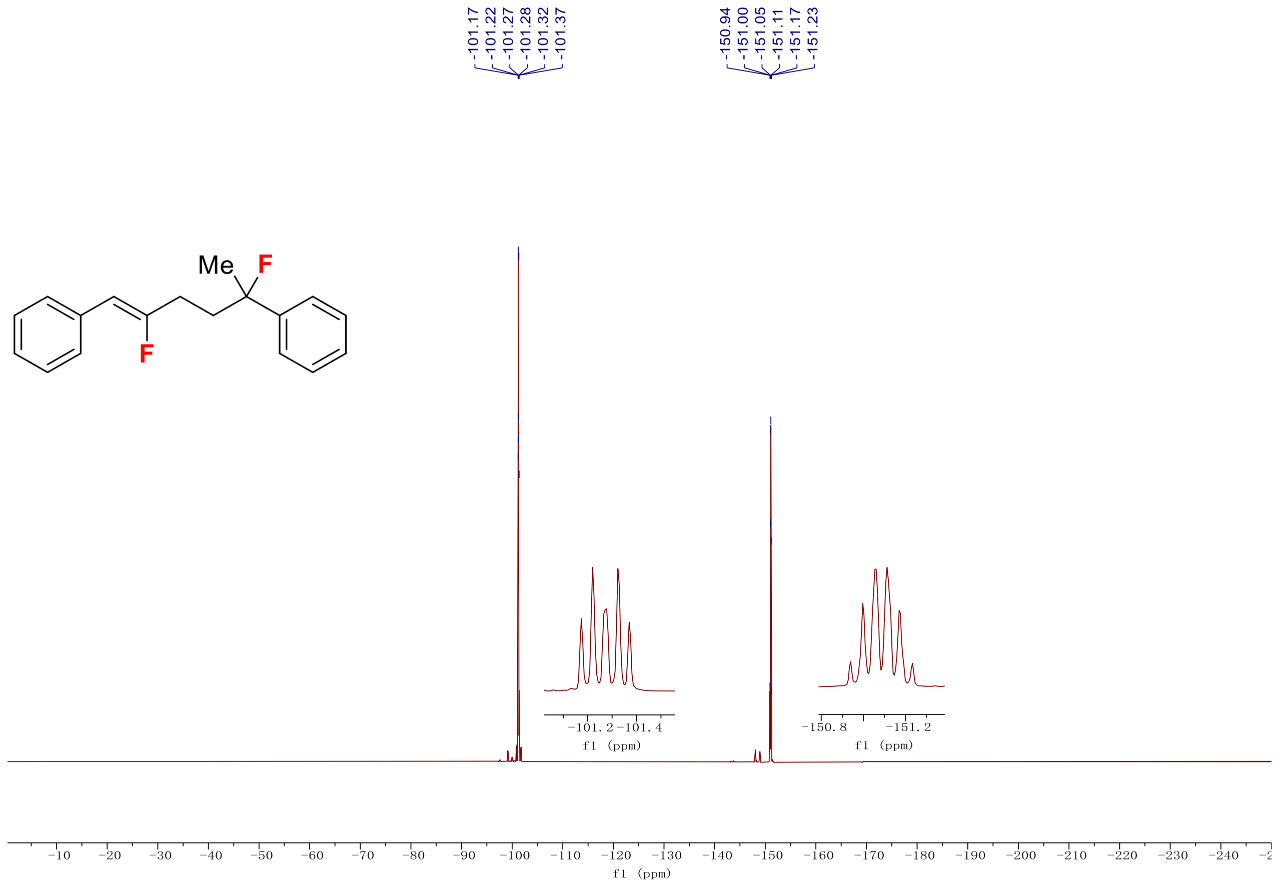

^{1}H NMR (400 MHz, CDCl$_3$) spectrum of 3f

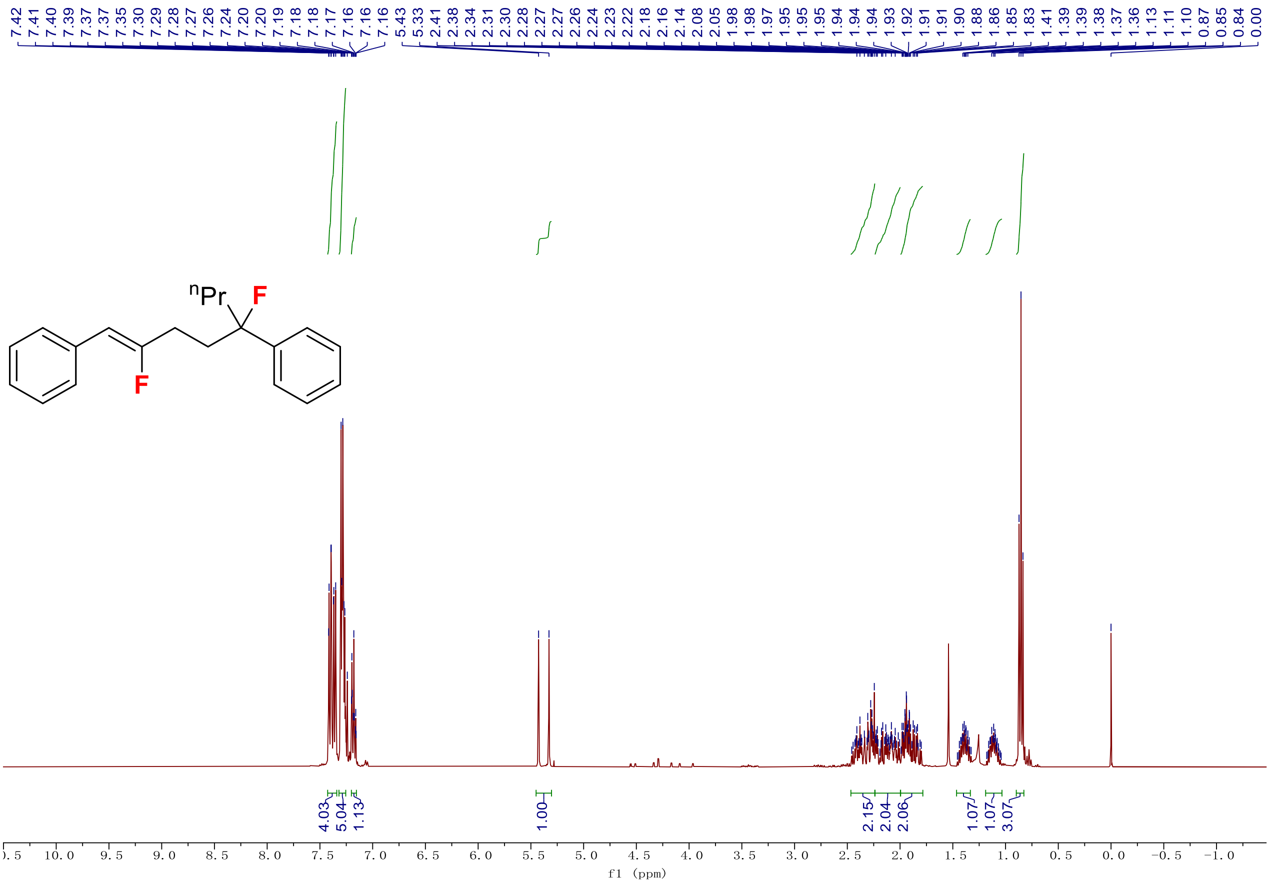

^{13}C NMR (101 MHz, CDCl$_3$) spectrum of 3f

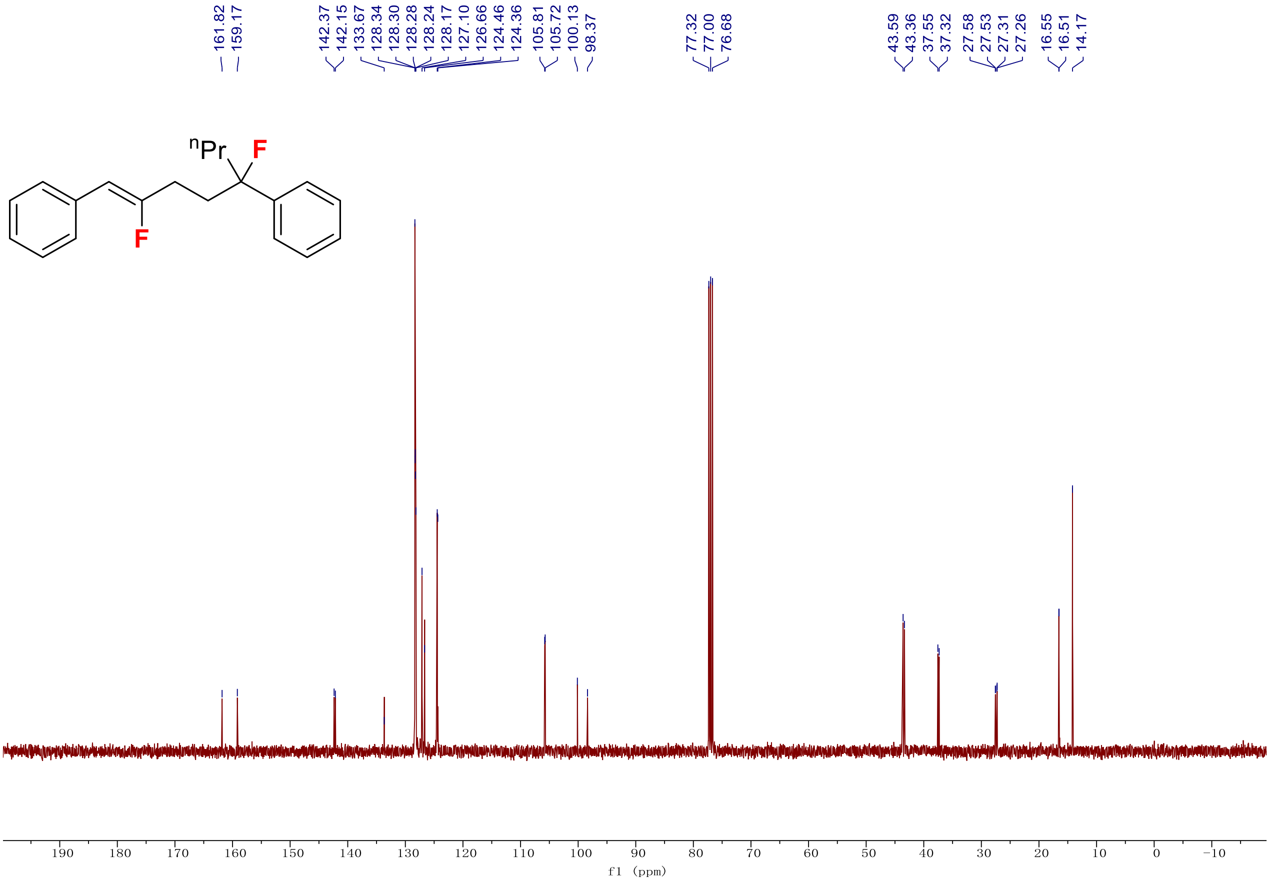

^{19}F NMR (376 MHz, $CDCl_3$) spectrum of 3f

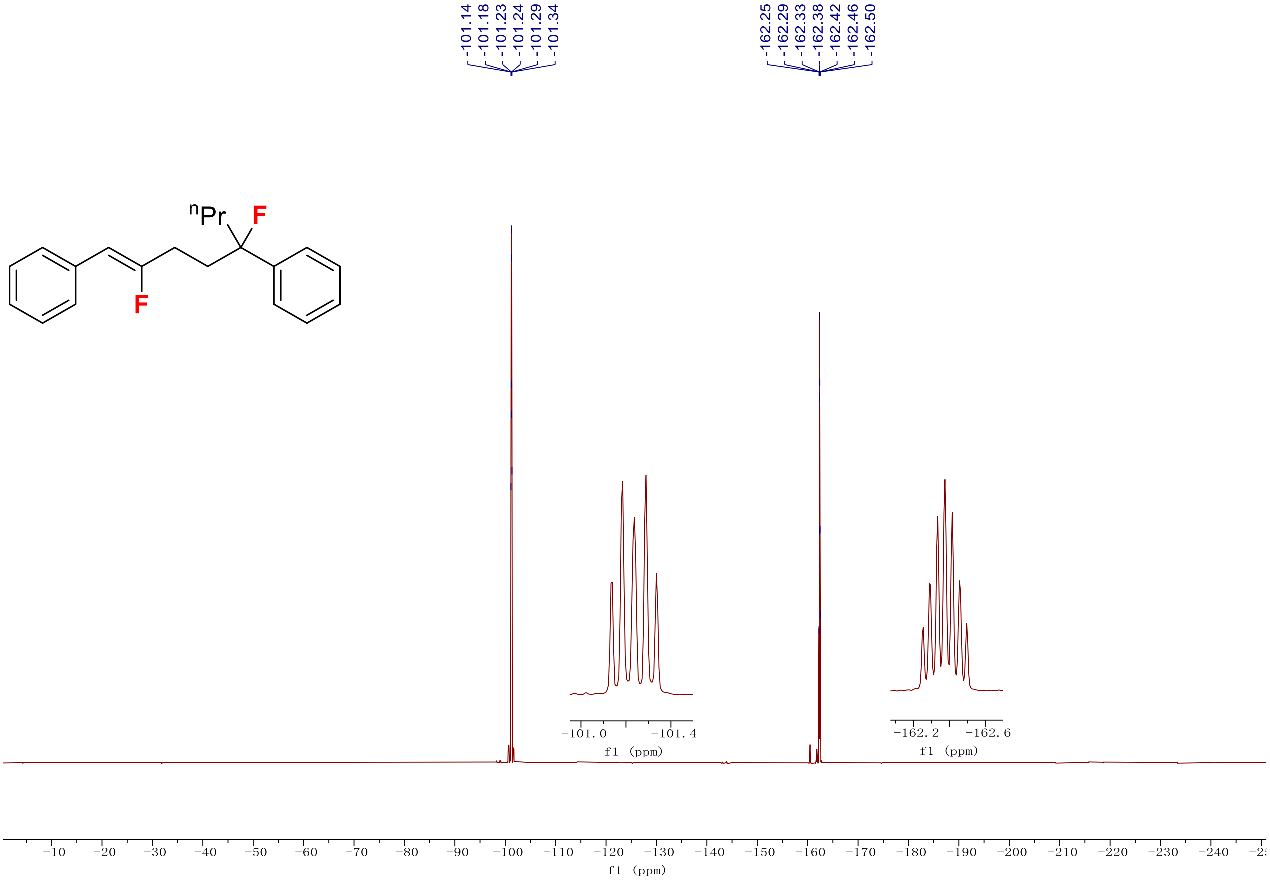

^{1}H NMR (400 MHz, CDCl3) spectrum of 3g

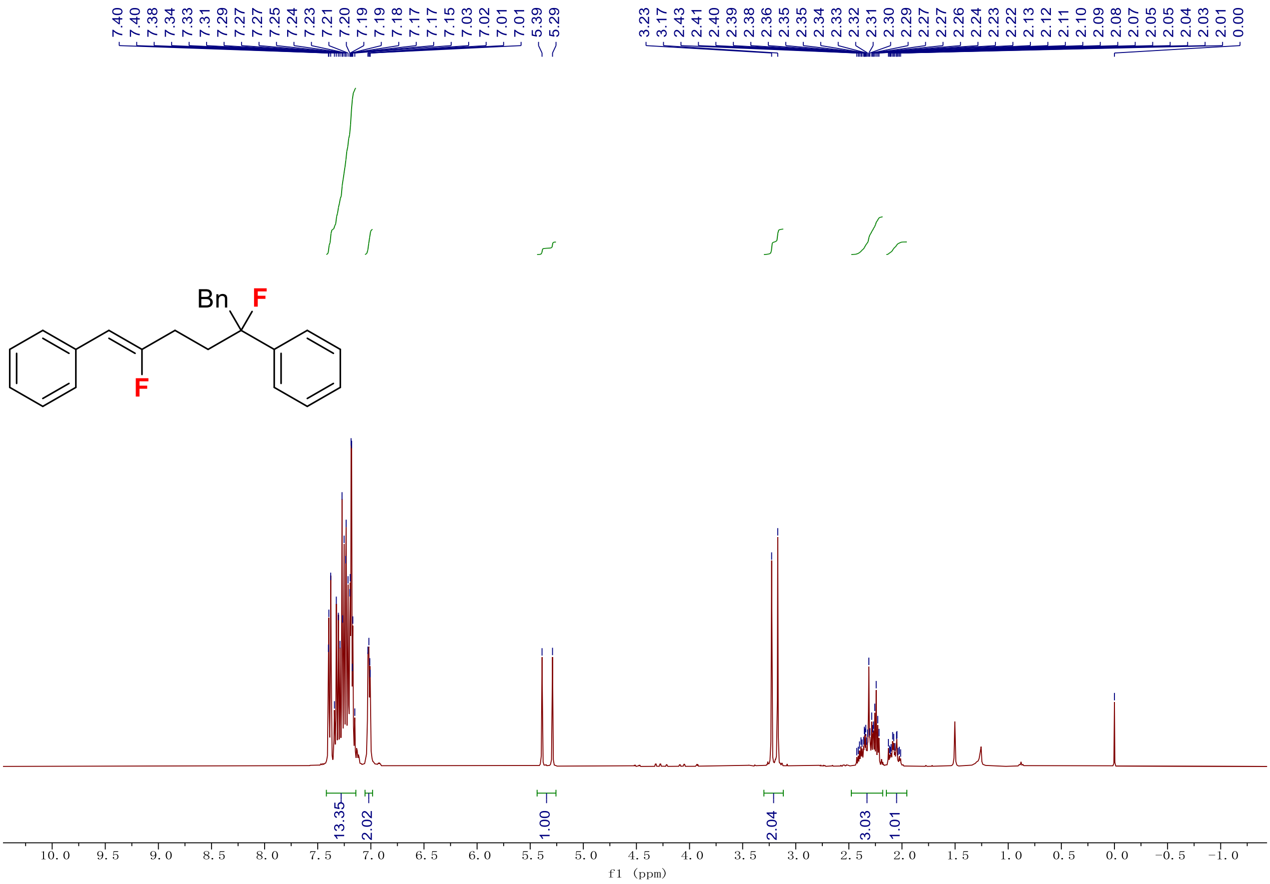

^{13}C NMR (101 MHz, CDCl3) spectrum of 3g

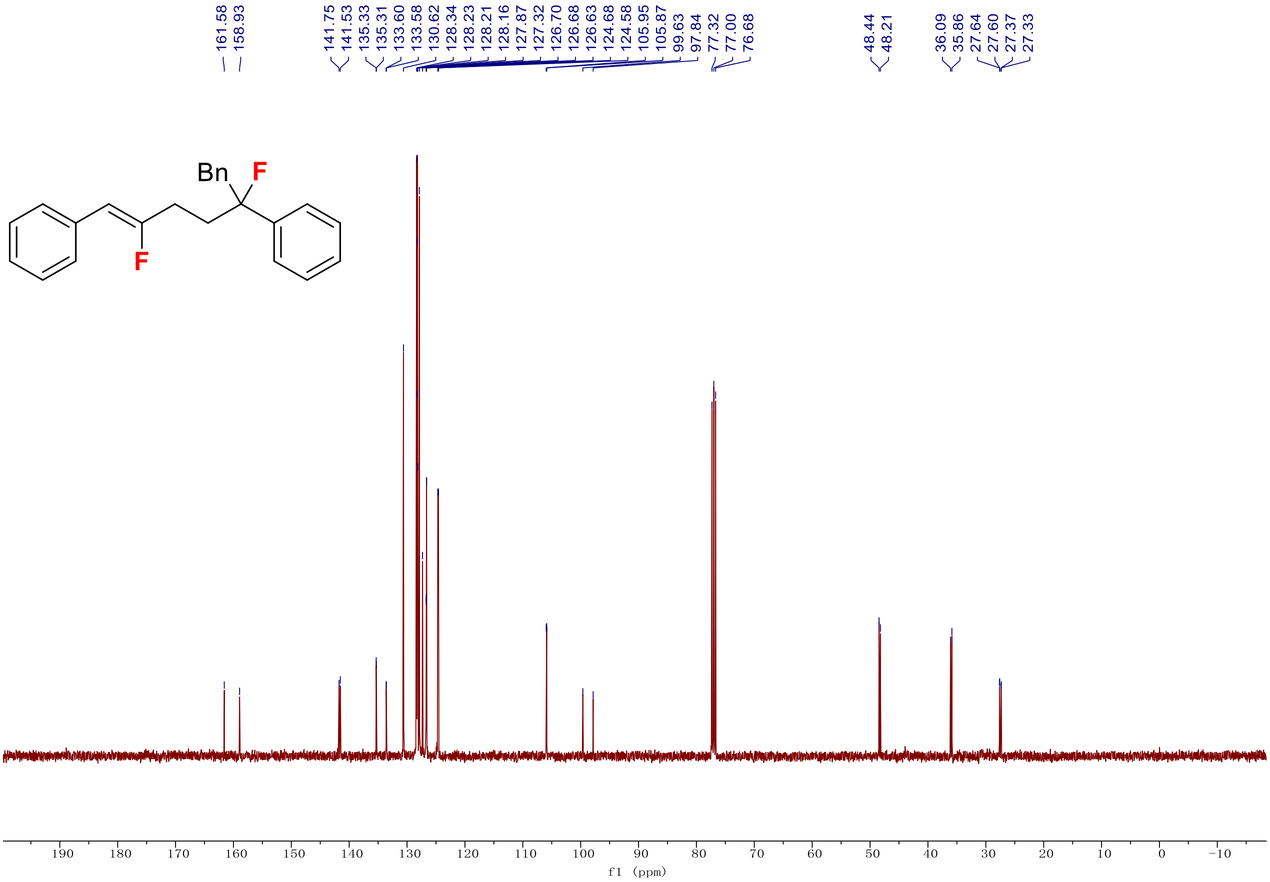

^{19}F NMR (376 MHz, $CDCl_3$) spectrum of 3g

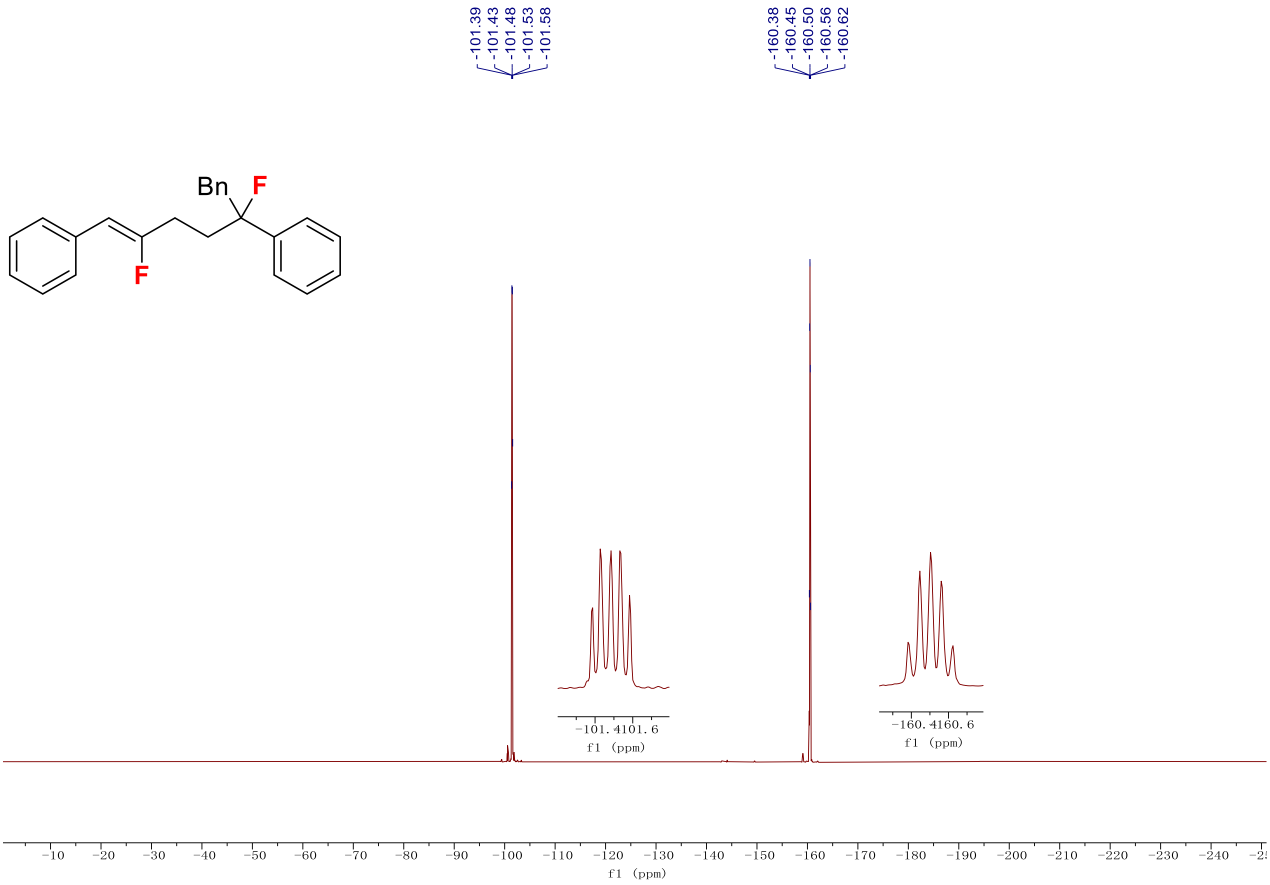

^{1}H NMR (400 MHz, $CDCl_3$) spectrum of 3h

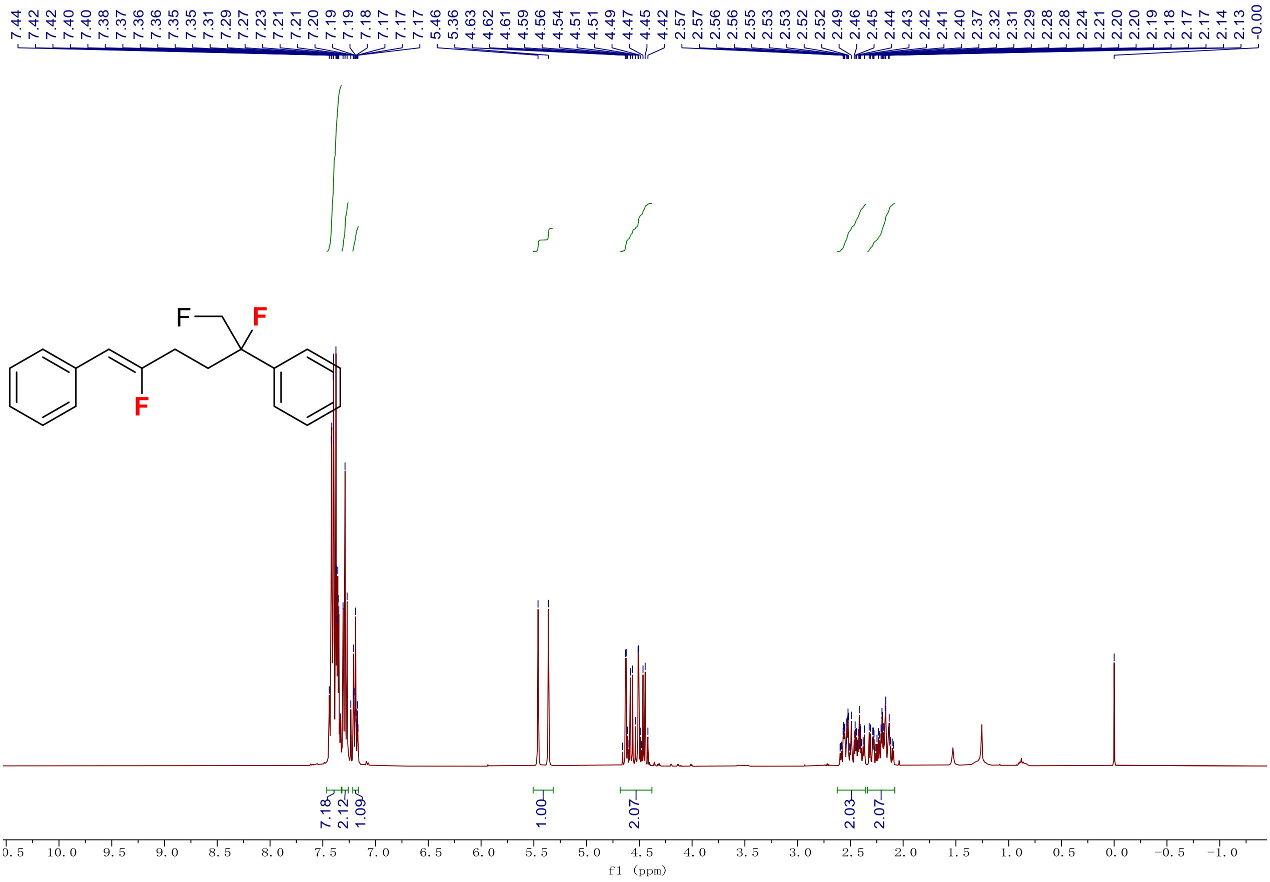

^{13}C NMR (101 MHz, $CDCl_3$) spectrum of 3h

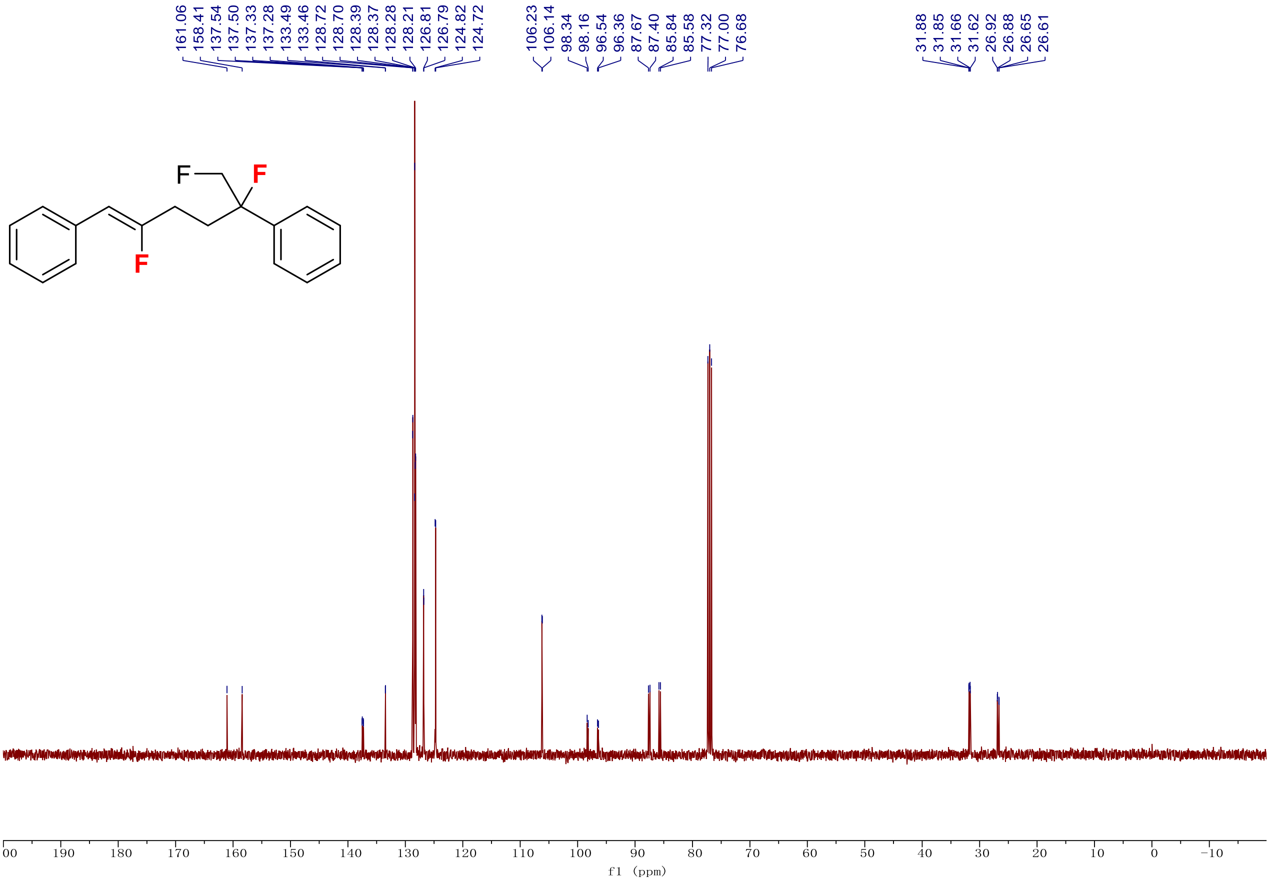

^{19}F NMR (376 MHz, CDCl3) spectrum of 3h

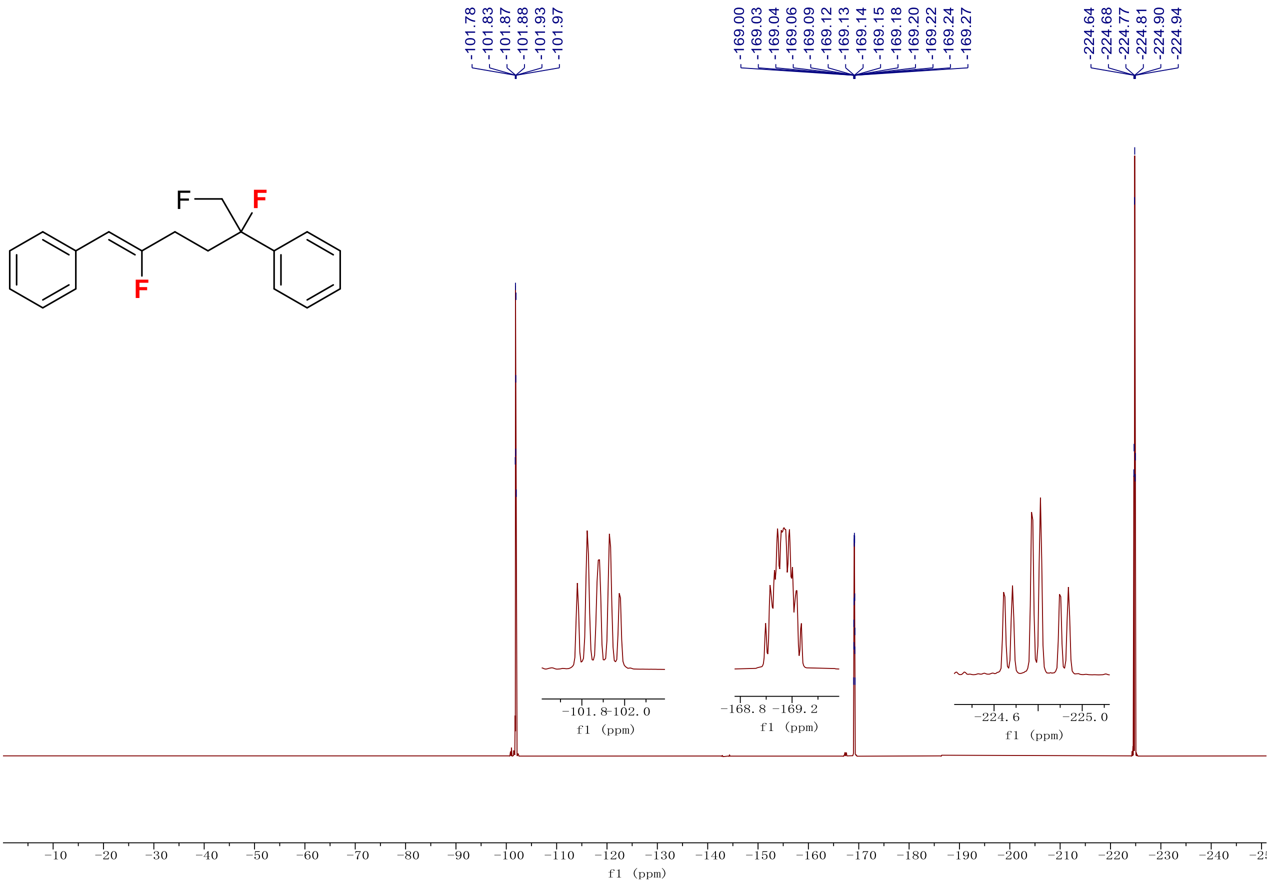

^{1}H NMR (400 MHz, CDCl$_3$) spectrum of 3i

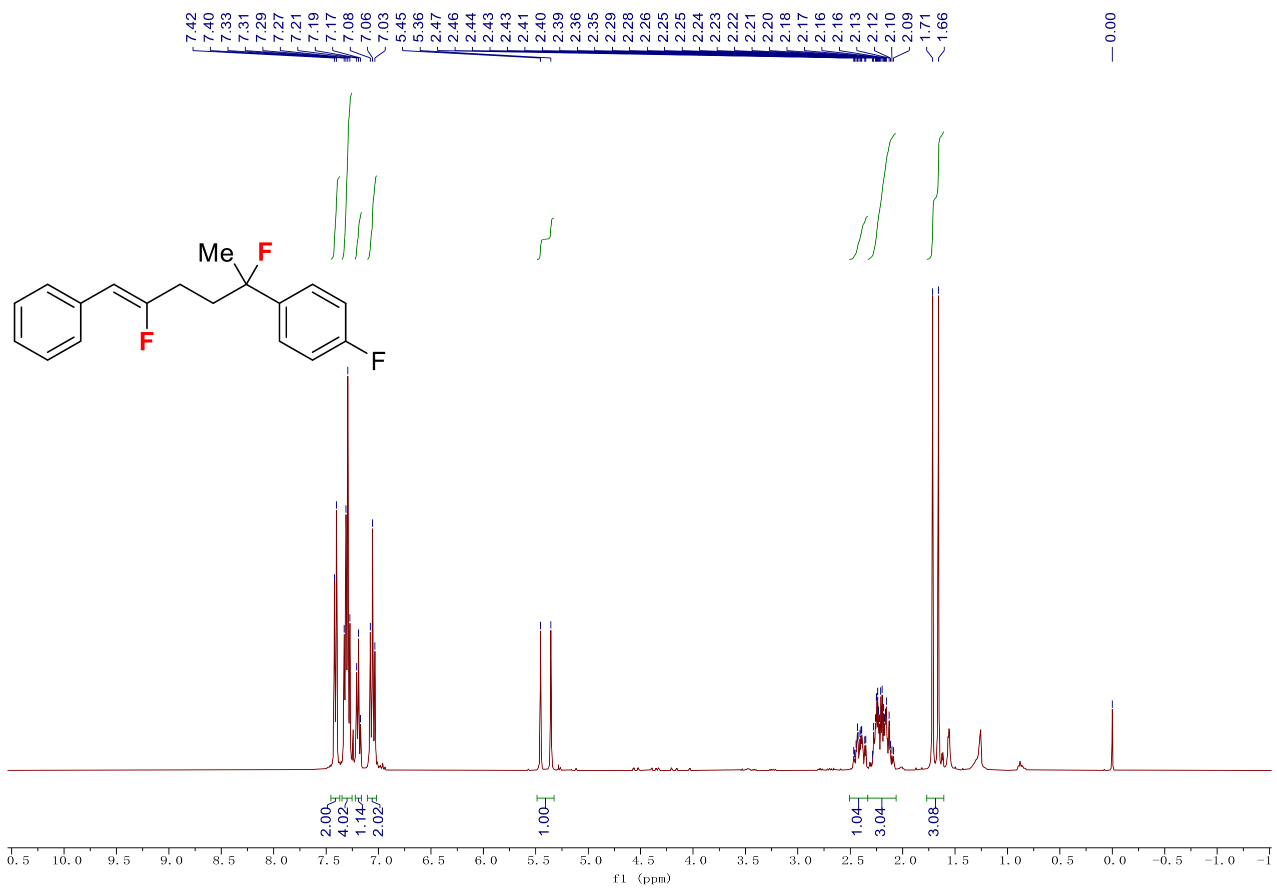

^{13}C NMR (101 MHz, CDCl$_3$) spectrum of 3i

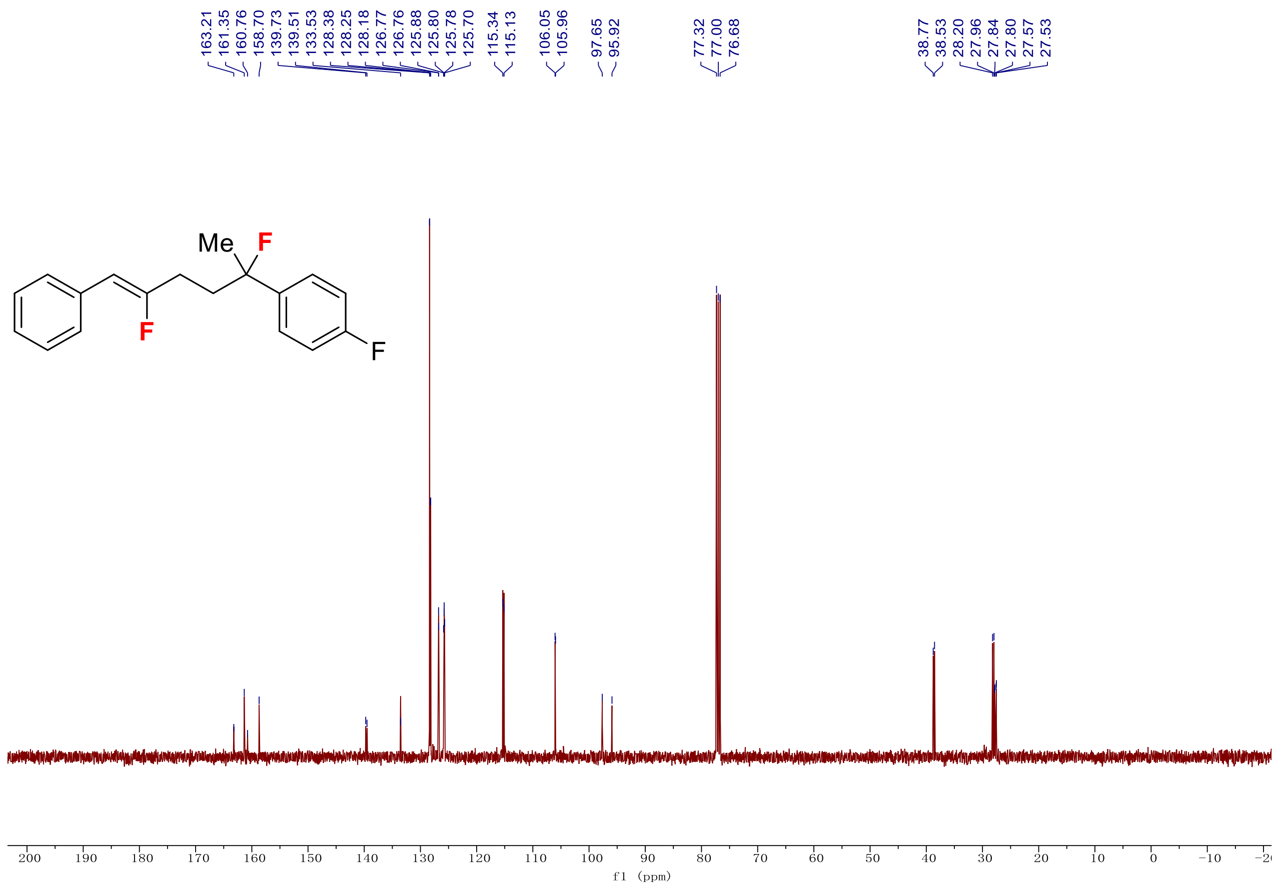

^{19}F NMR (376 MHz, CDCl$_3$) spectrum of 3i

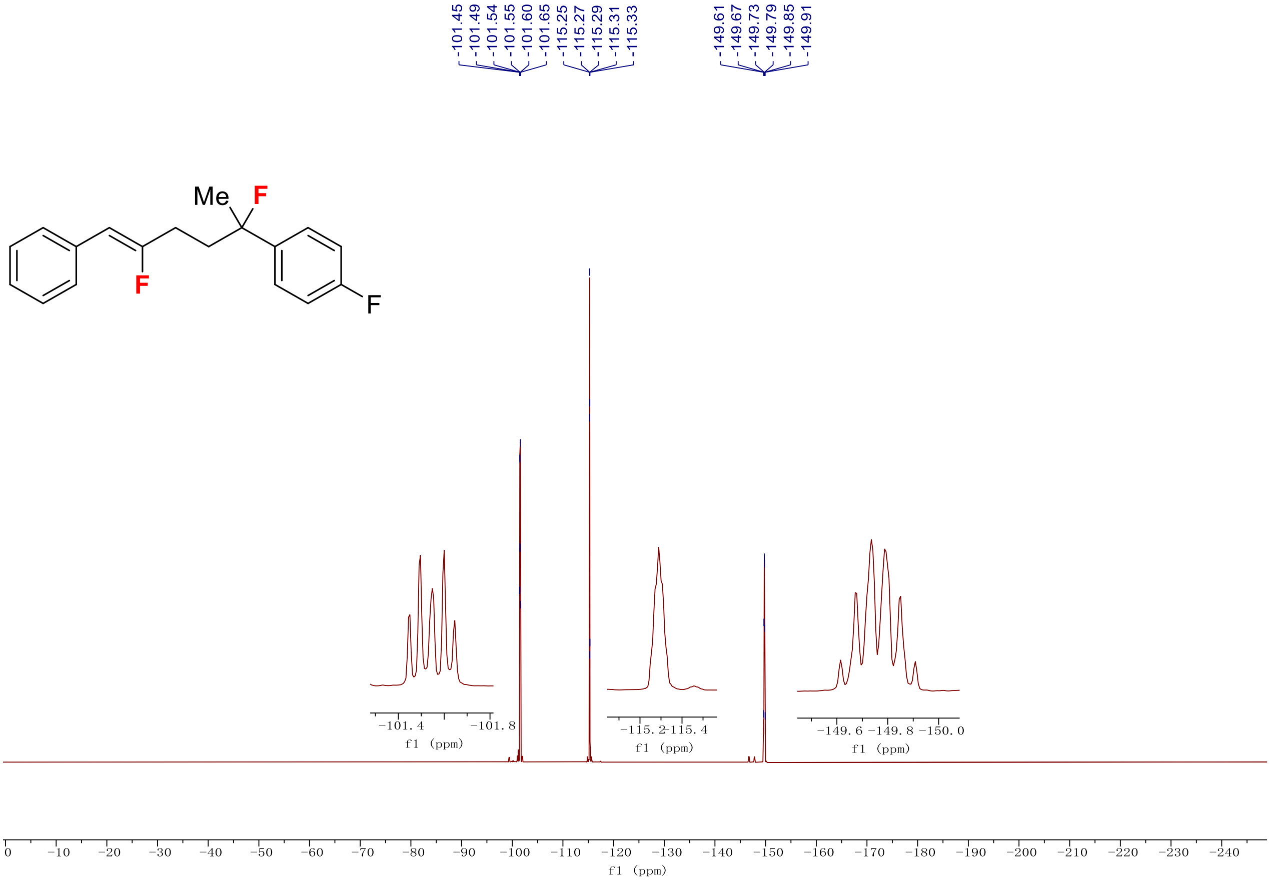

^{1}H NMR (400 MHz, $CDCl_3$) spectrum of 3j

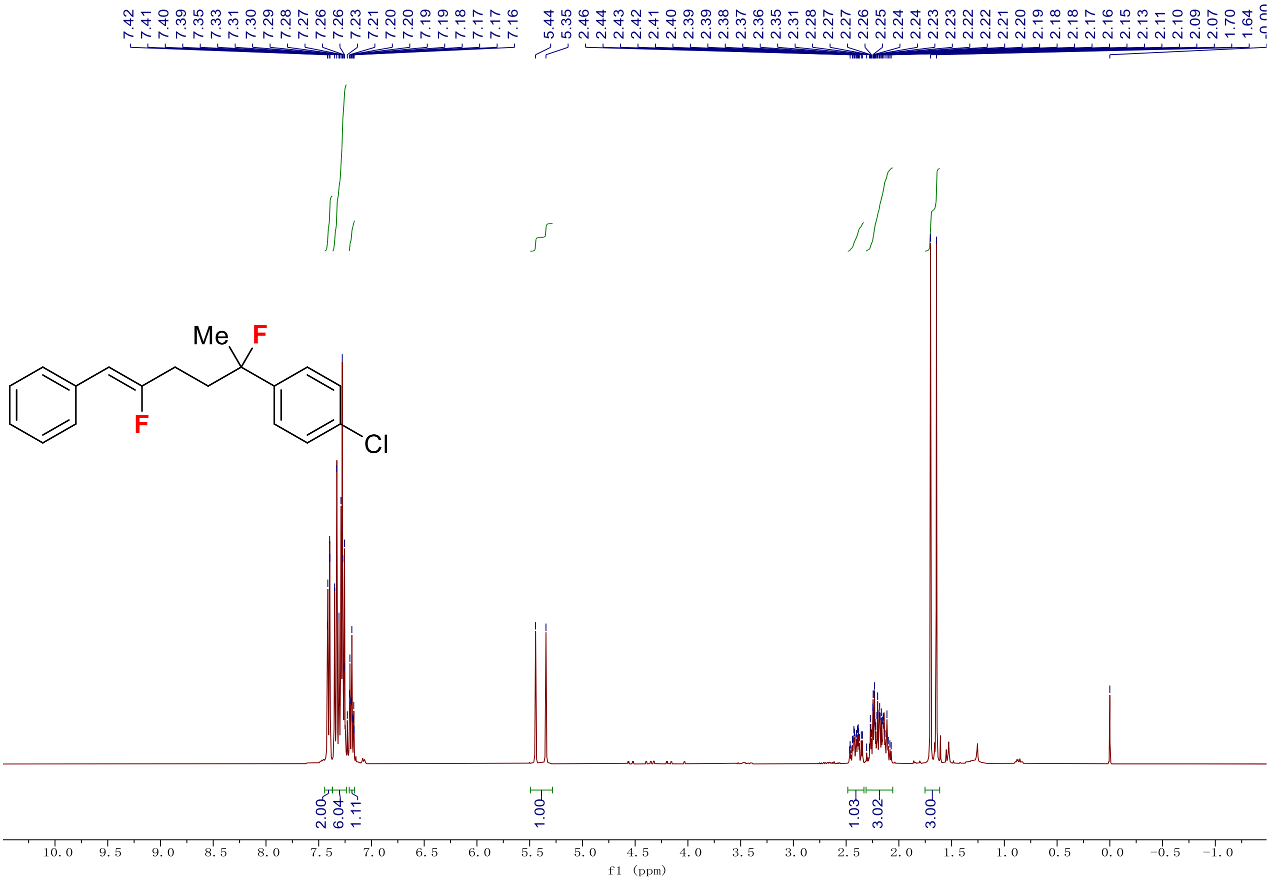

^{13}C NMR (101 MHz, $CDCl_3$) spectrum of 3j

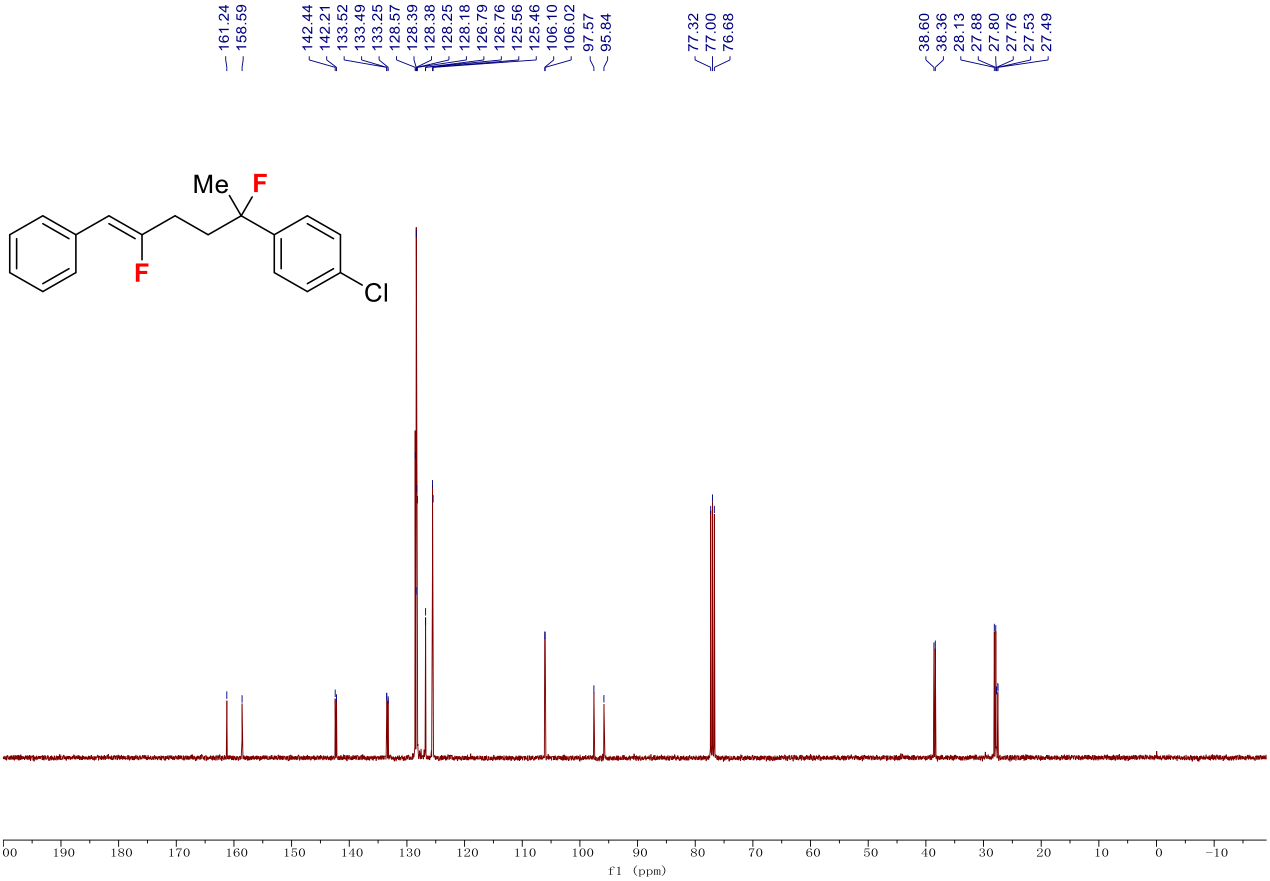

^{19}F NMR (376 MHz, $CDCl_3$) spectrum of 3j

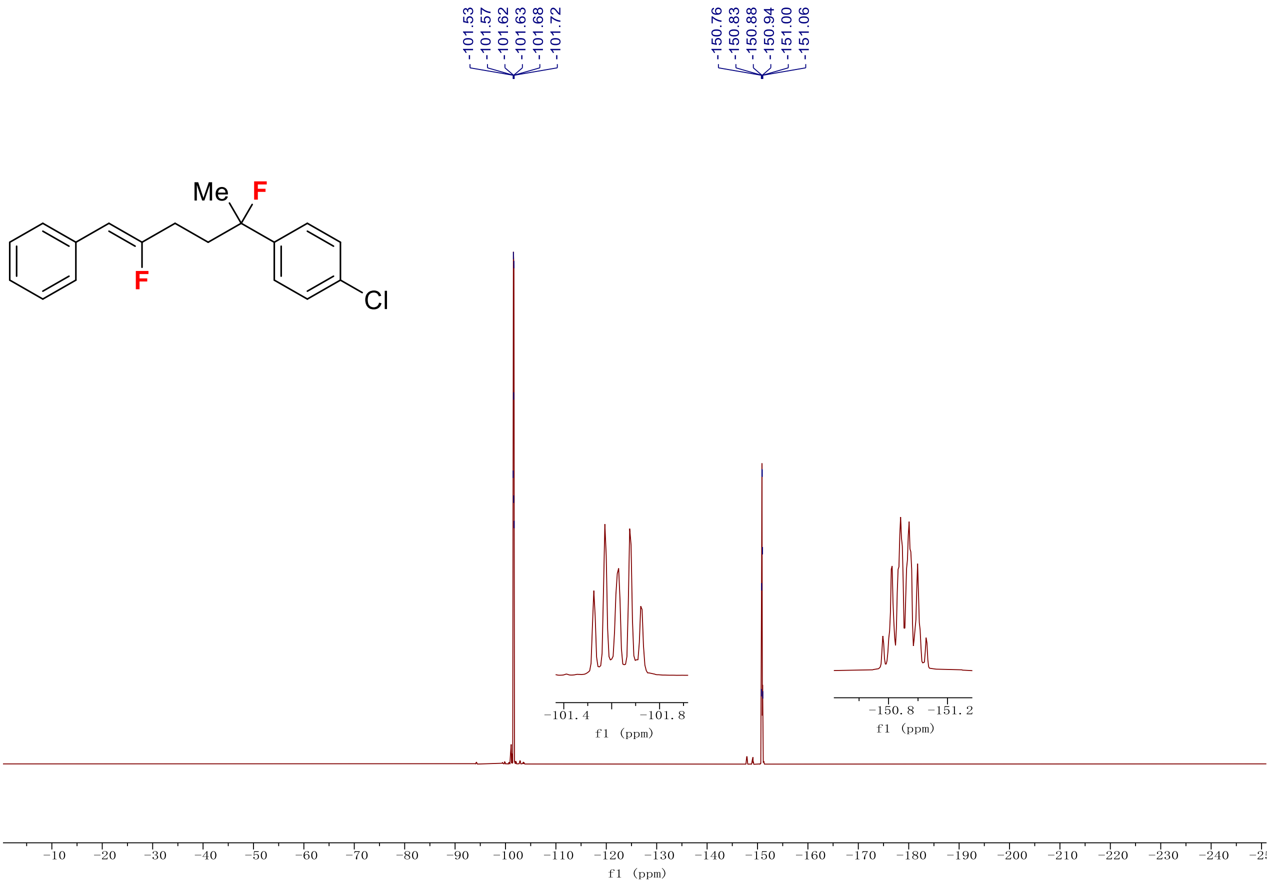

^{1}H NMR (400 MHz, CDCl3) spectrum of 3k

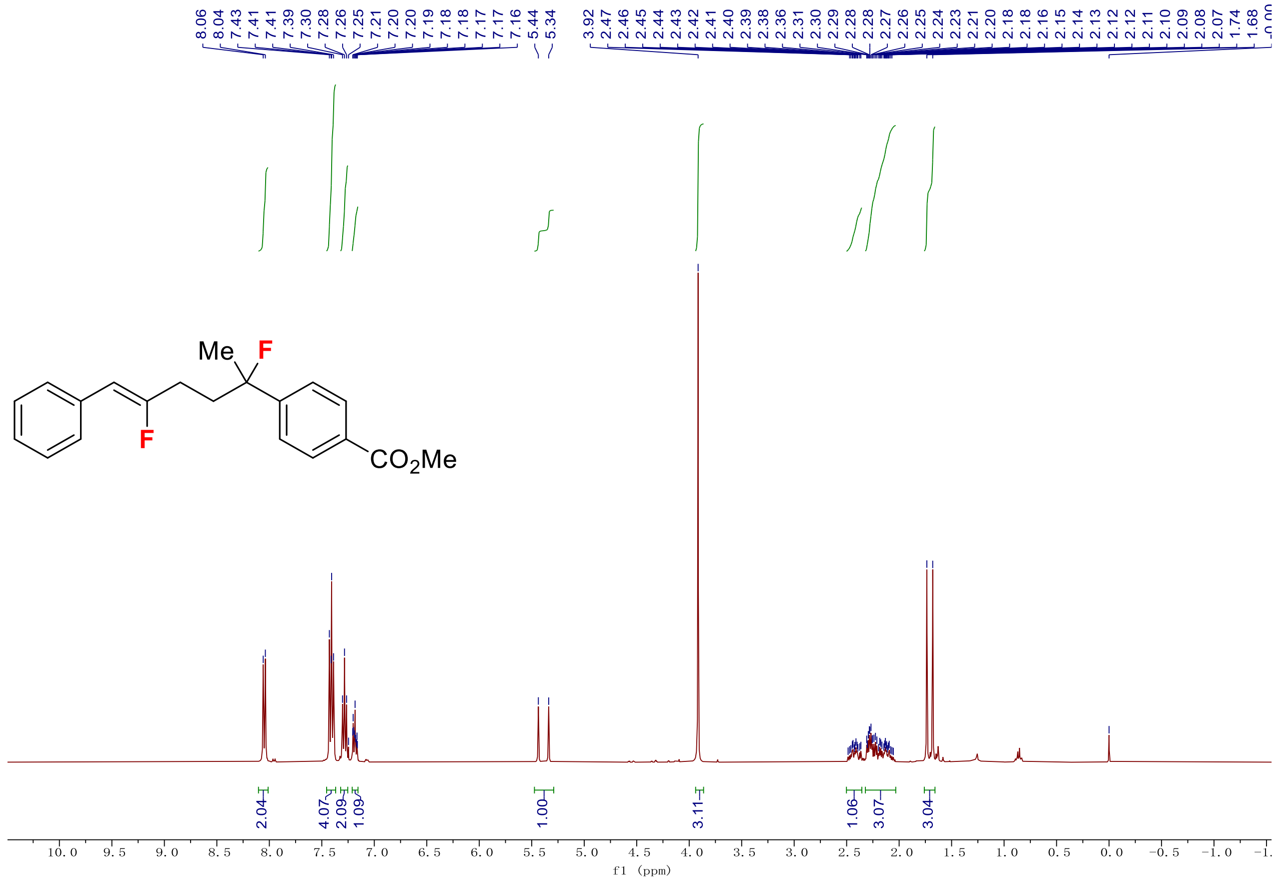

^{13}C NMR (101 MHz, CDCl3) spectrum of 3k

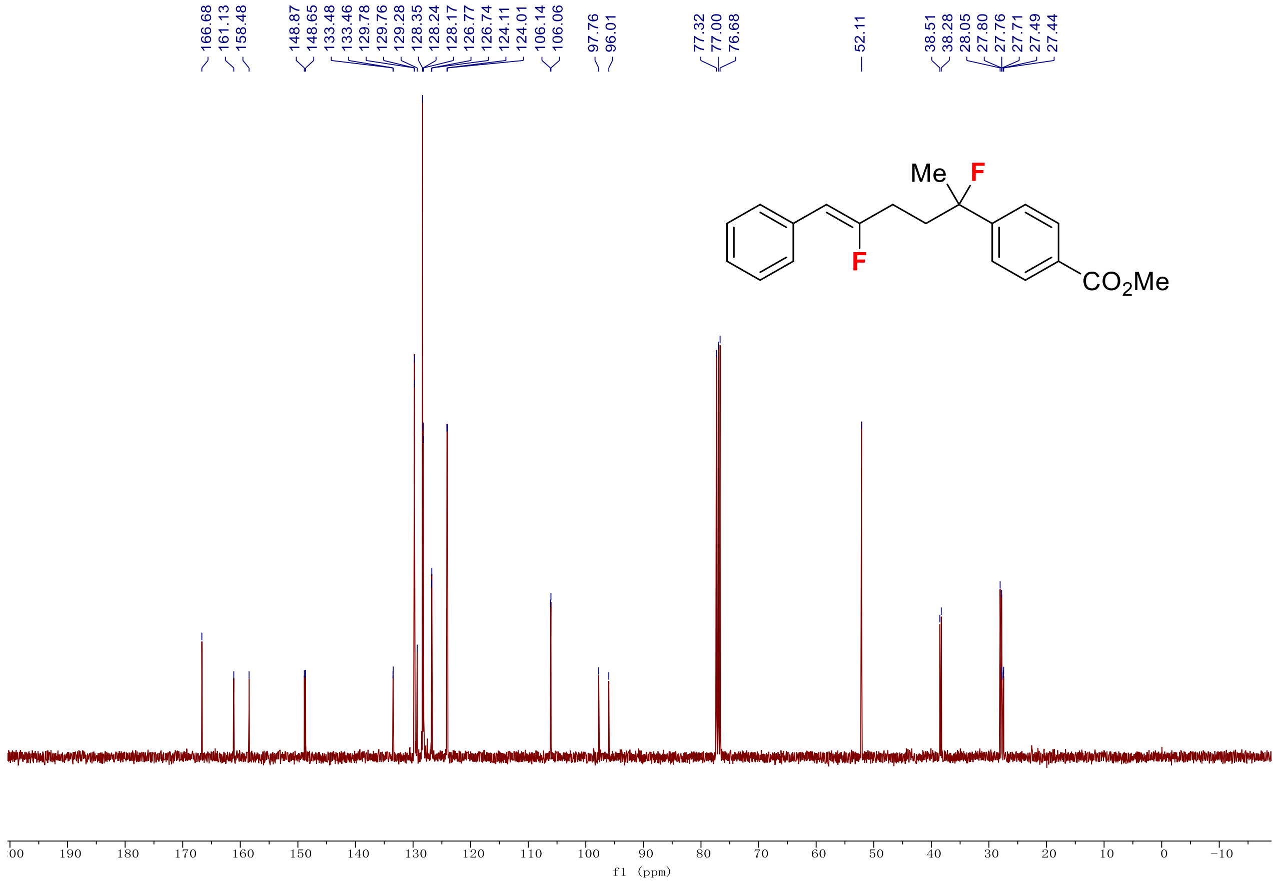

^{19}F NMR (376 MHz, $CDCl_3$) spectrum of 3k

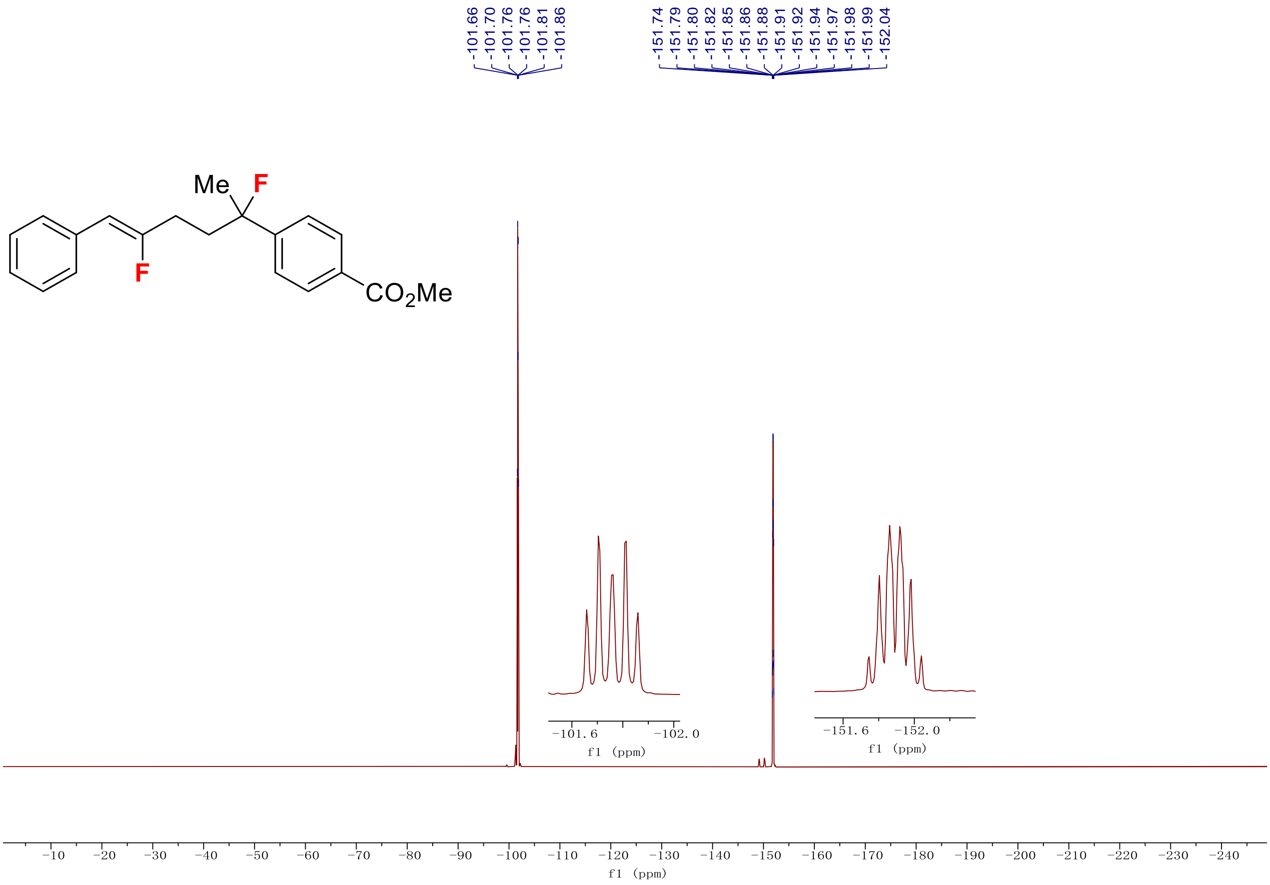

^{1}H NMR (400 MHz, $CDCl_3$) spectrum of 3l

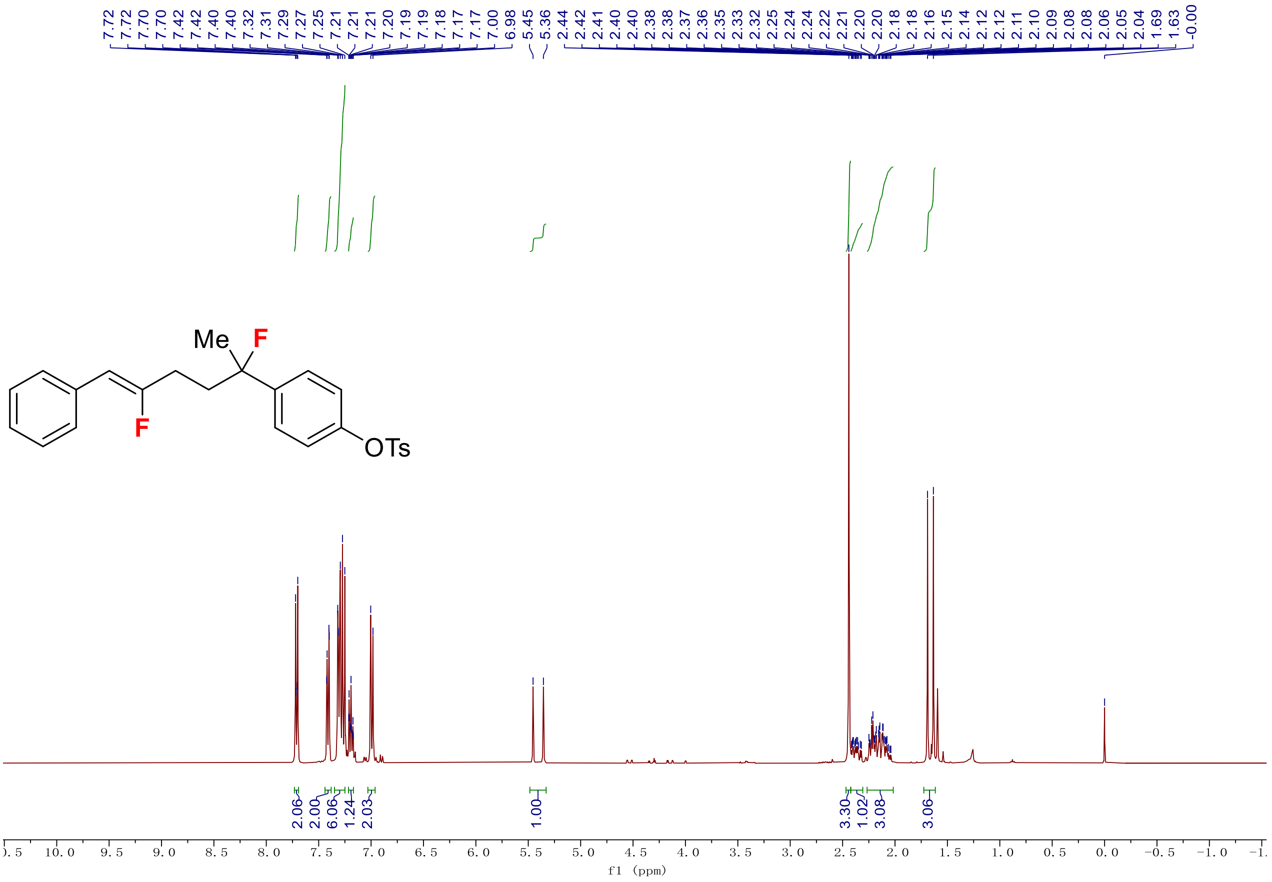

^{13}C NMR (101 MHz, $CDCl_3$) spectrum of 3l

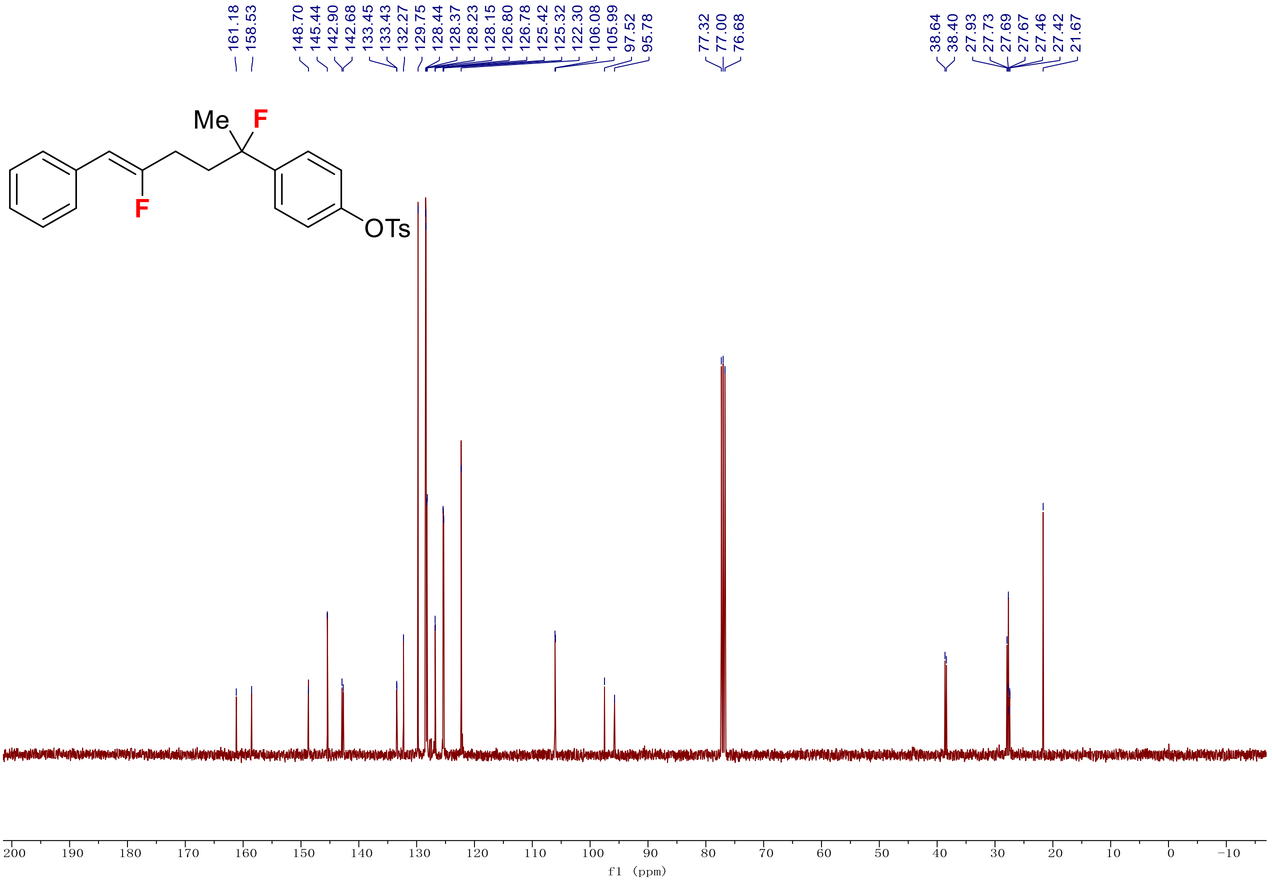

^{19}F NMR (376 MHz, CDCl3) spectrum of 3l

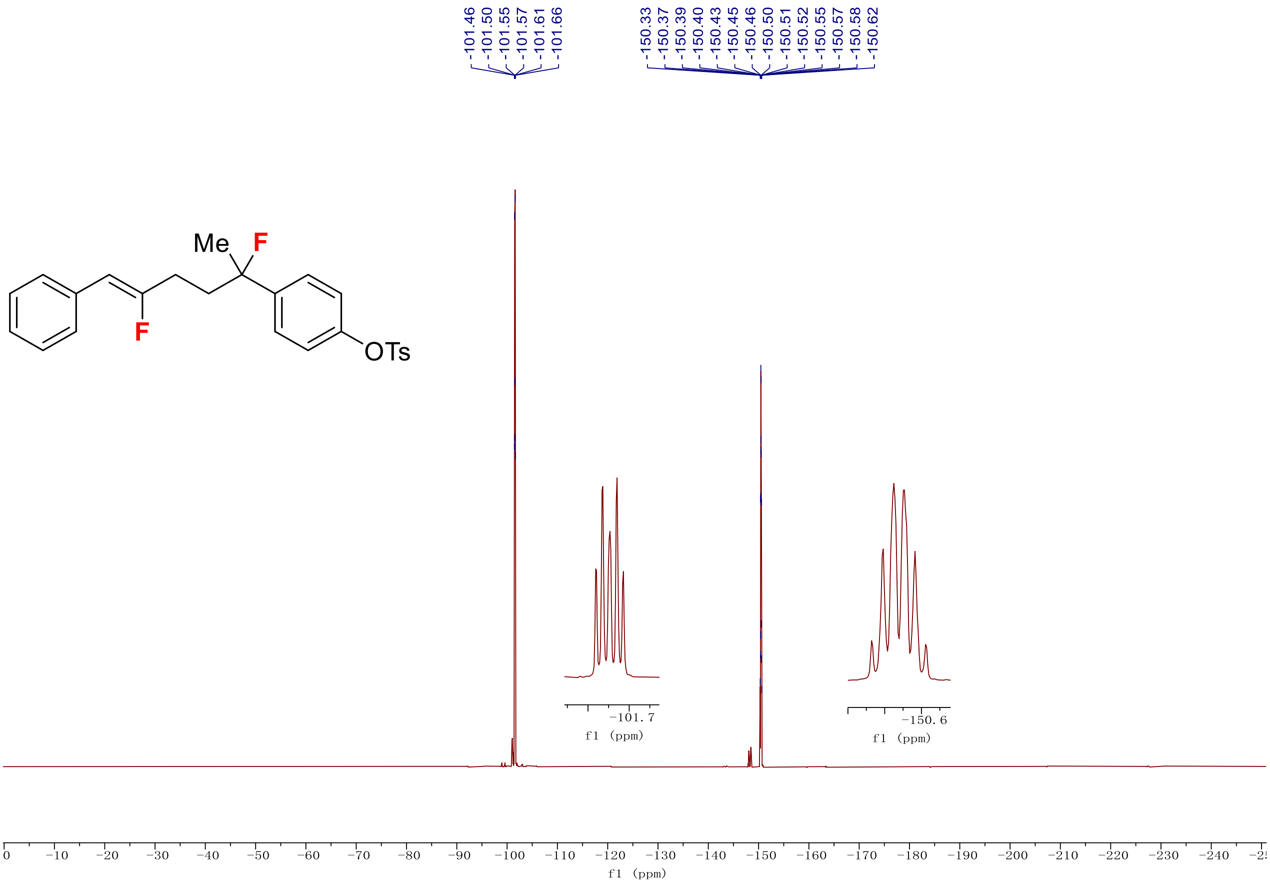

^{1}H NMR (400 MHz, CDCl3) spectrum of 3m

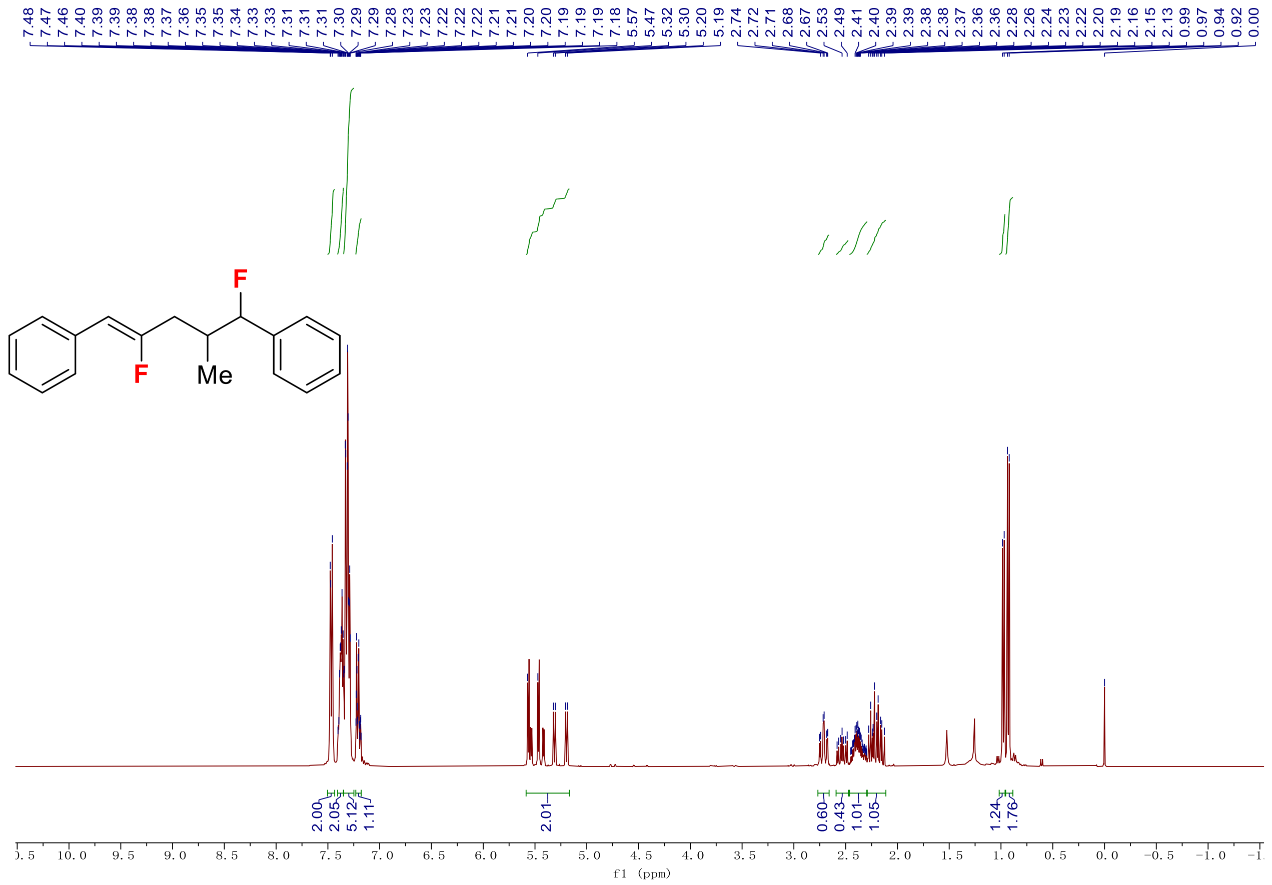

^{13}C NMR (101 MHz, CDCl3) spectrum of 3m

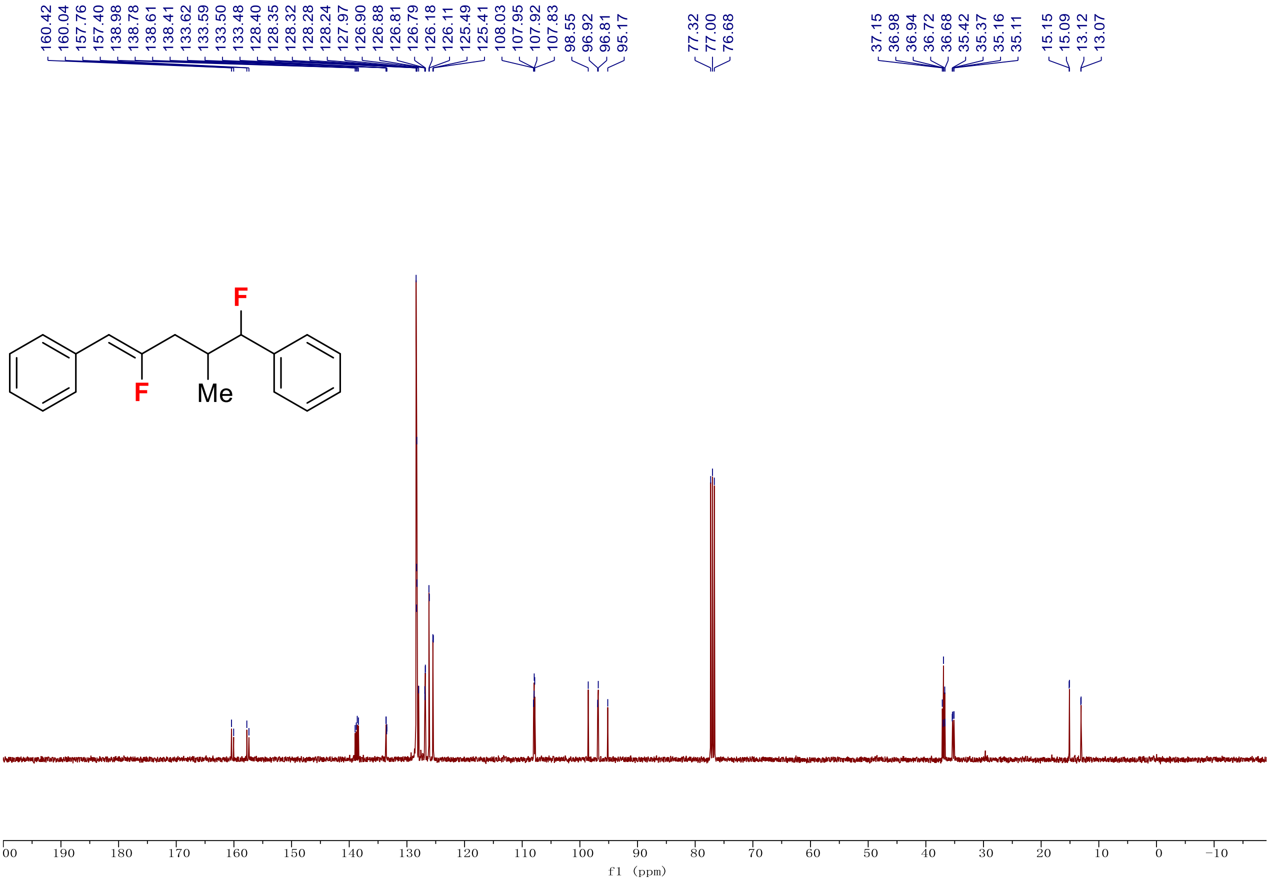

^{19}F NMR (376 MHz, CDCl3) spectrum of 3m

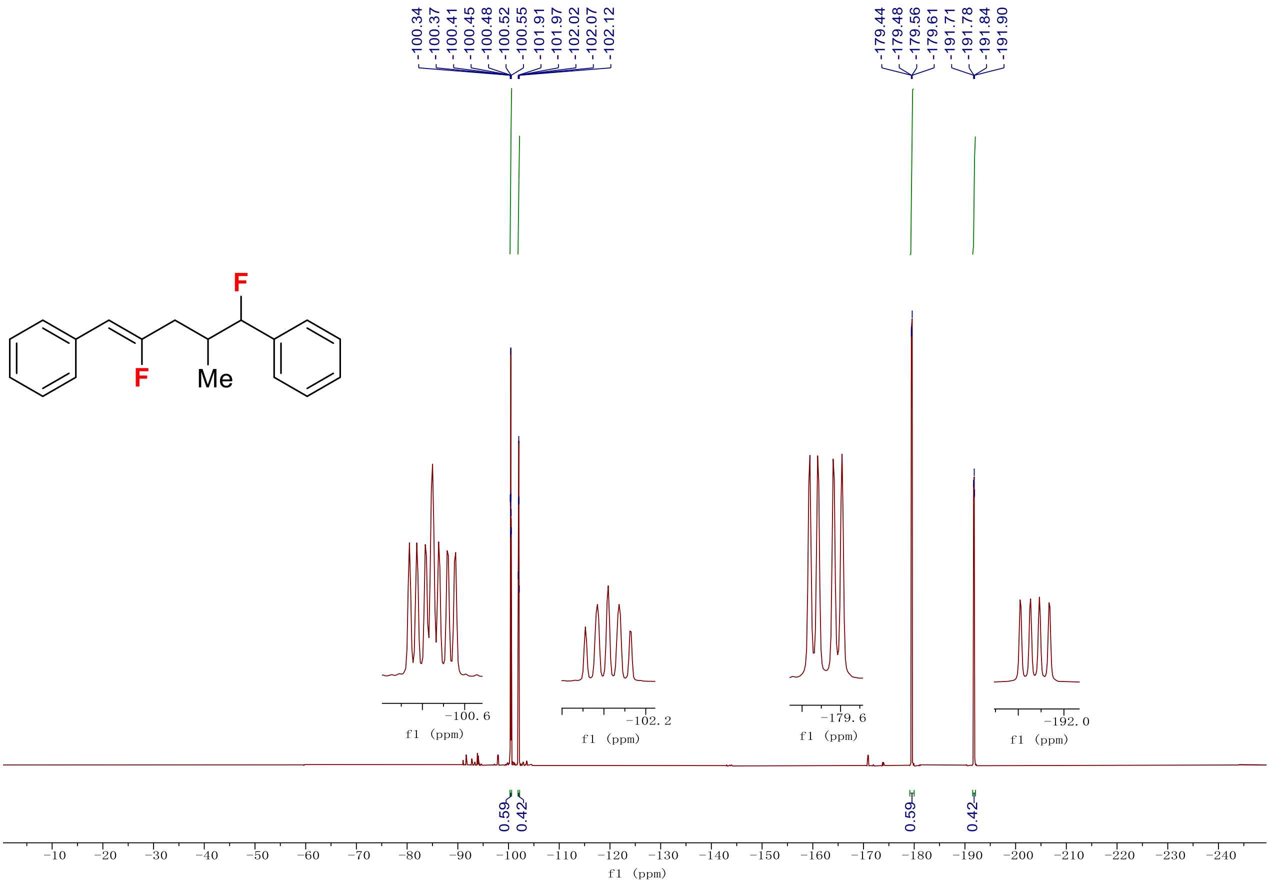

^{1}H NMR (400 MHz, CDCl3) spectrum of 3n

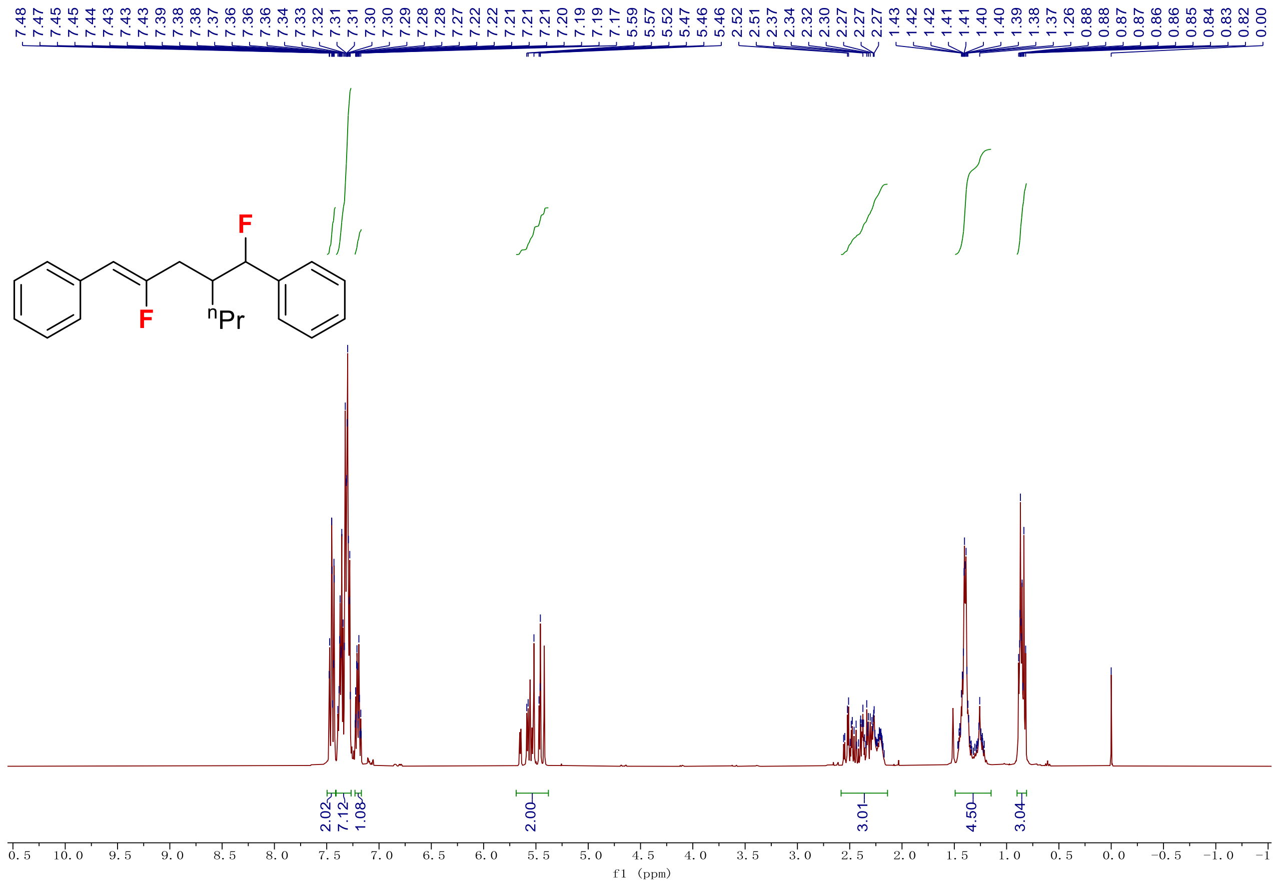

^{13}C NMR (101 MHz, CDCl3) spectrum of 3n

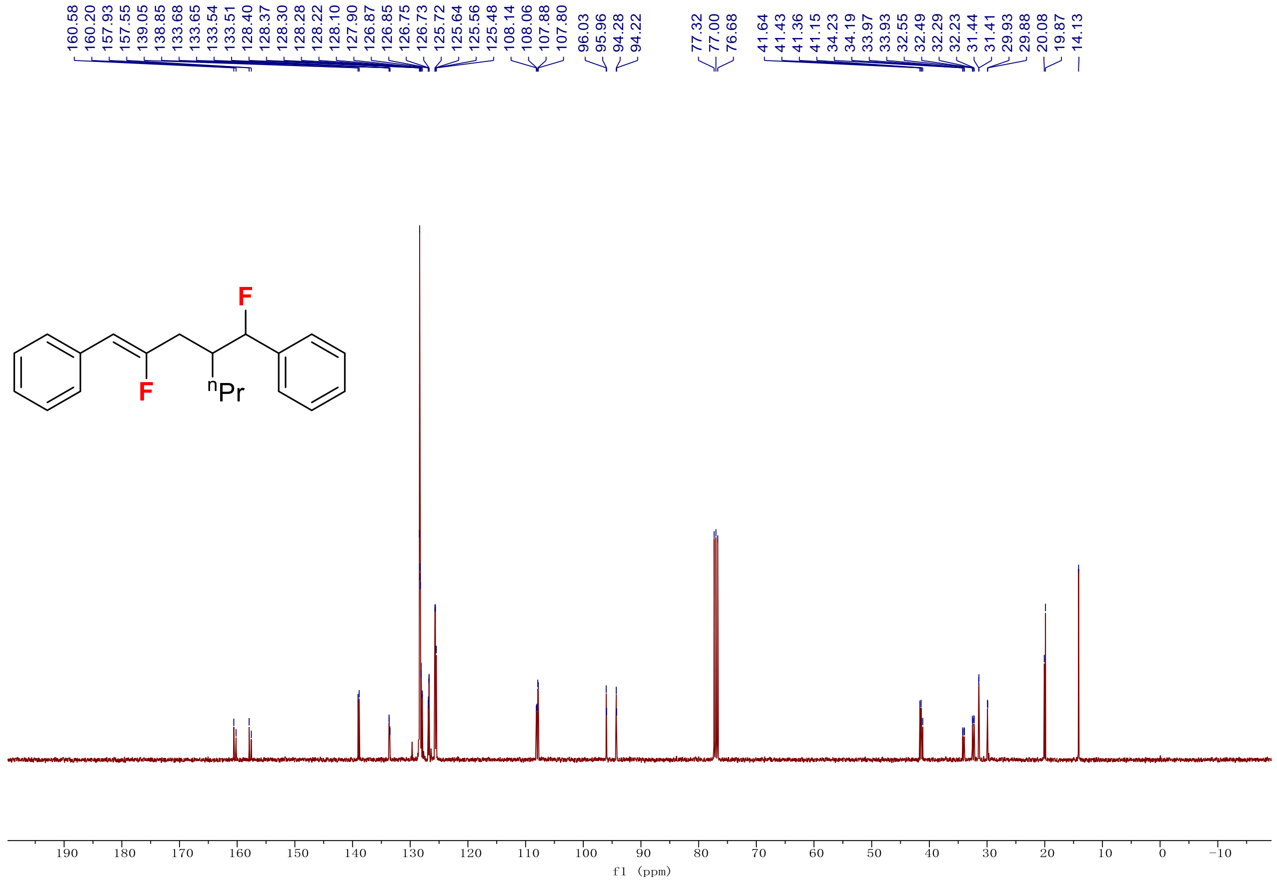

^{19}F NMR (376 MHz, CDCl3) spectrum of 3n

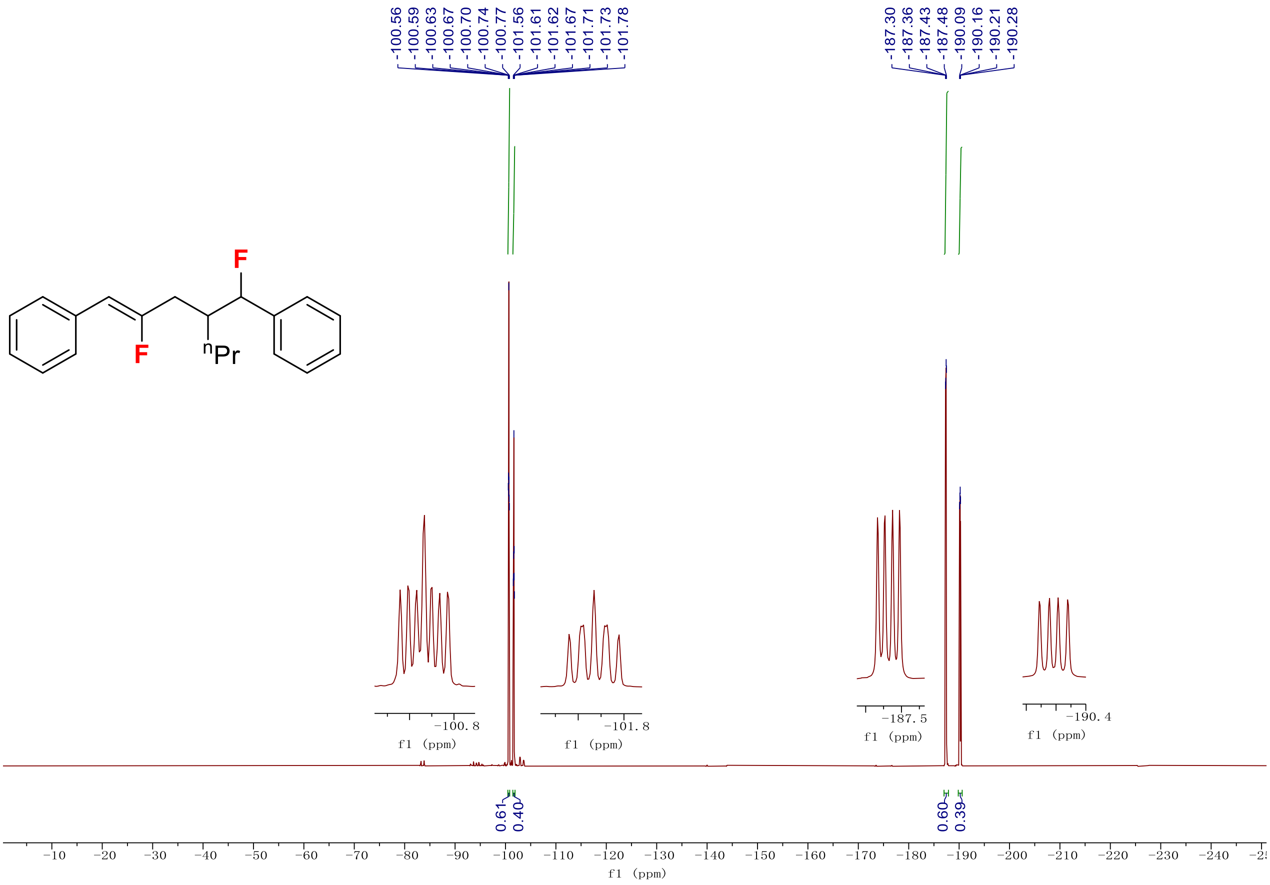

^{1}H NMR (400 MHz, $CDCl_3$) spectrum of 3o

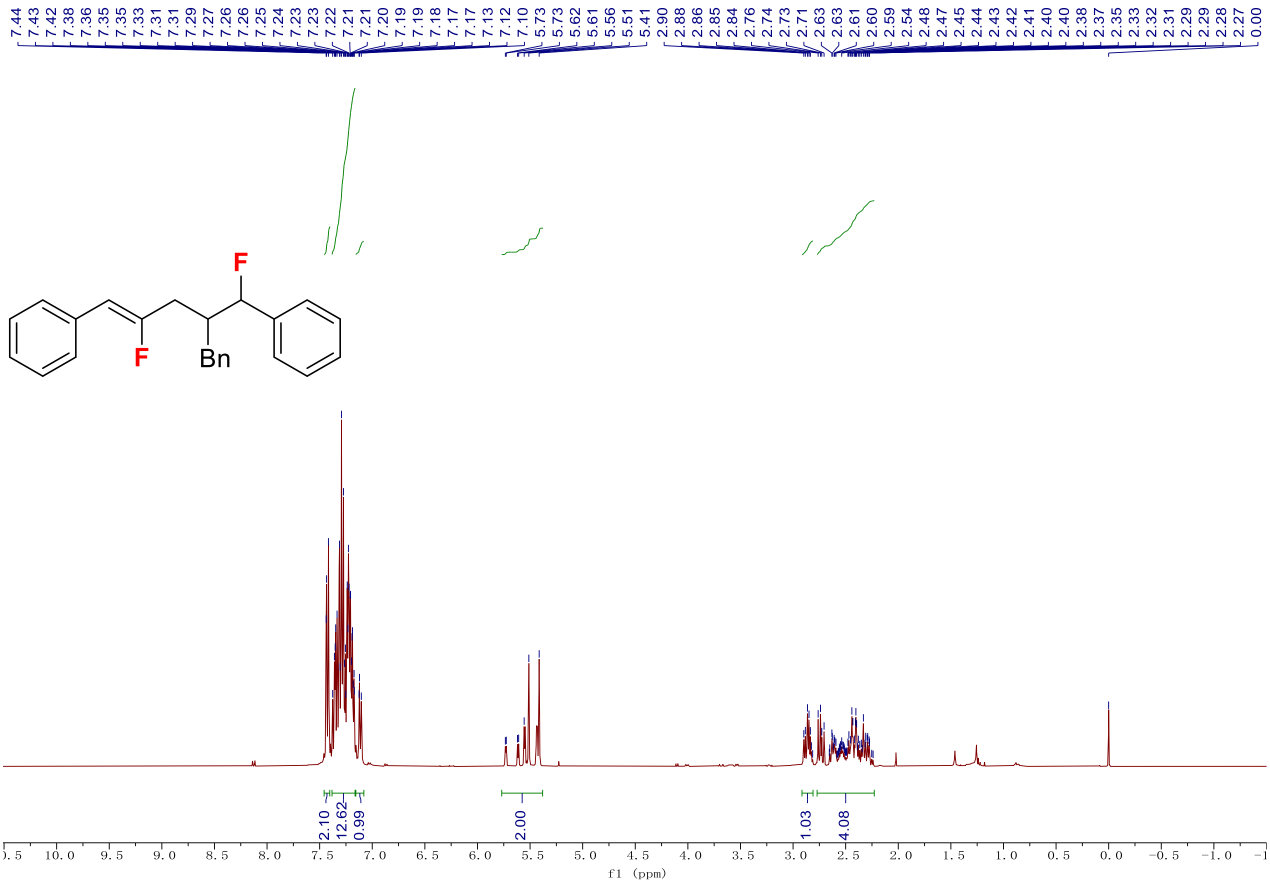

^{13}C NMR (101 MHz, $CDCl_3$) spectrum of 3o

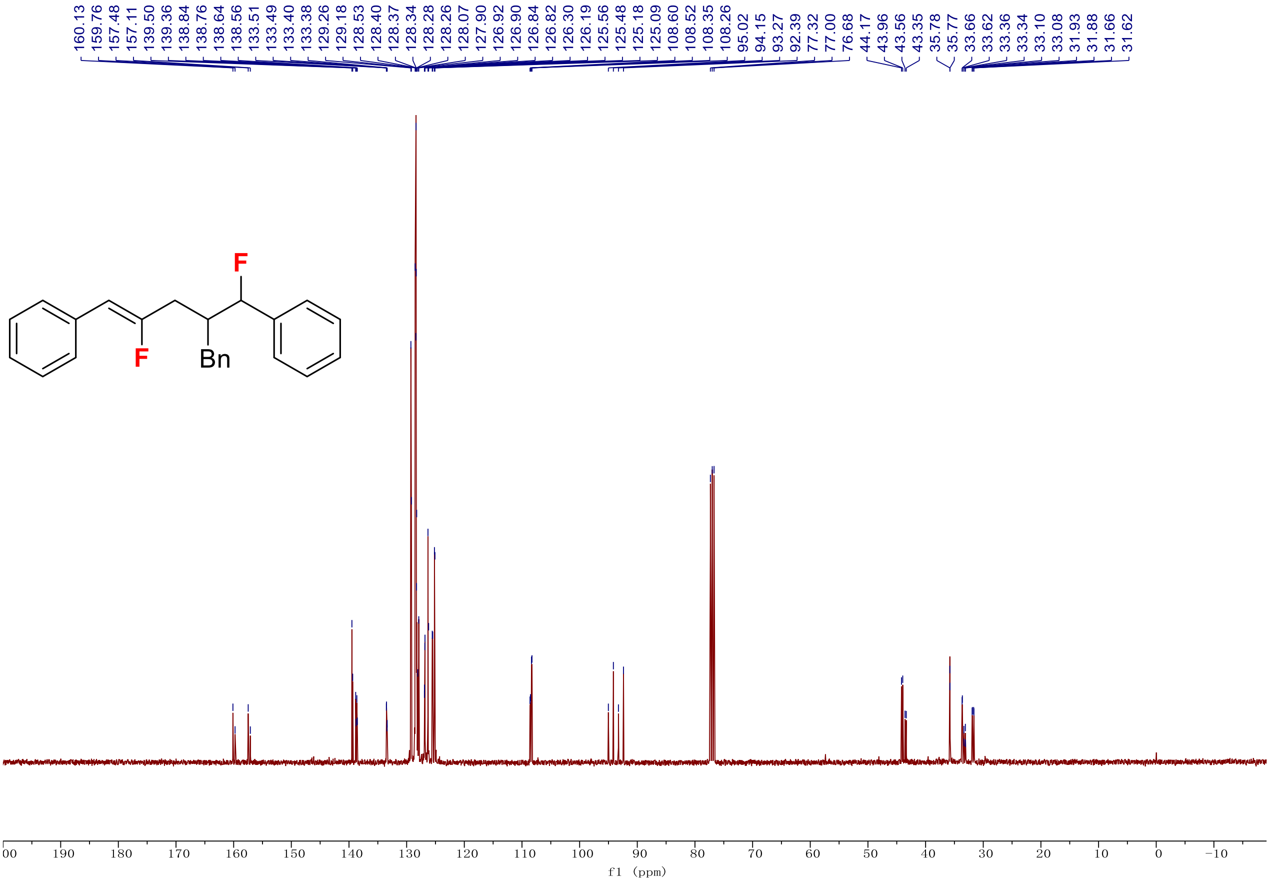

^{19}F NMR (376 MHz, $CDCl_3$) spectrum of 3o

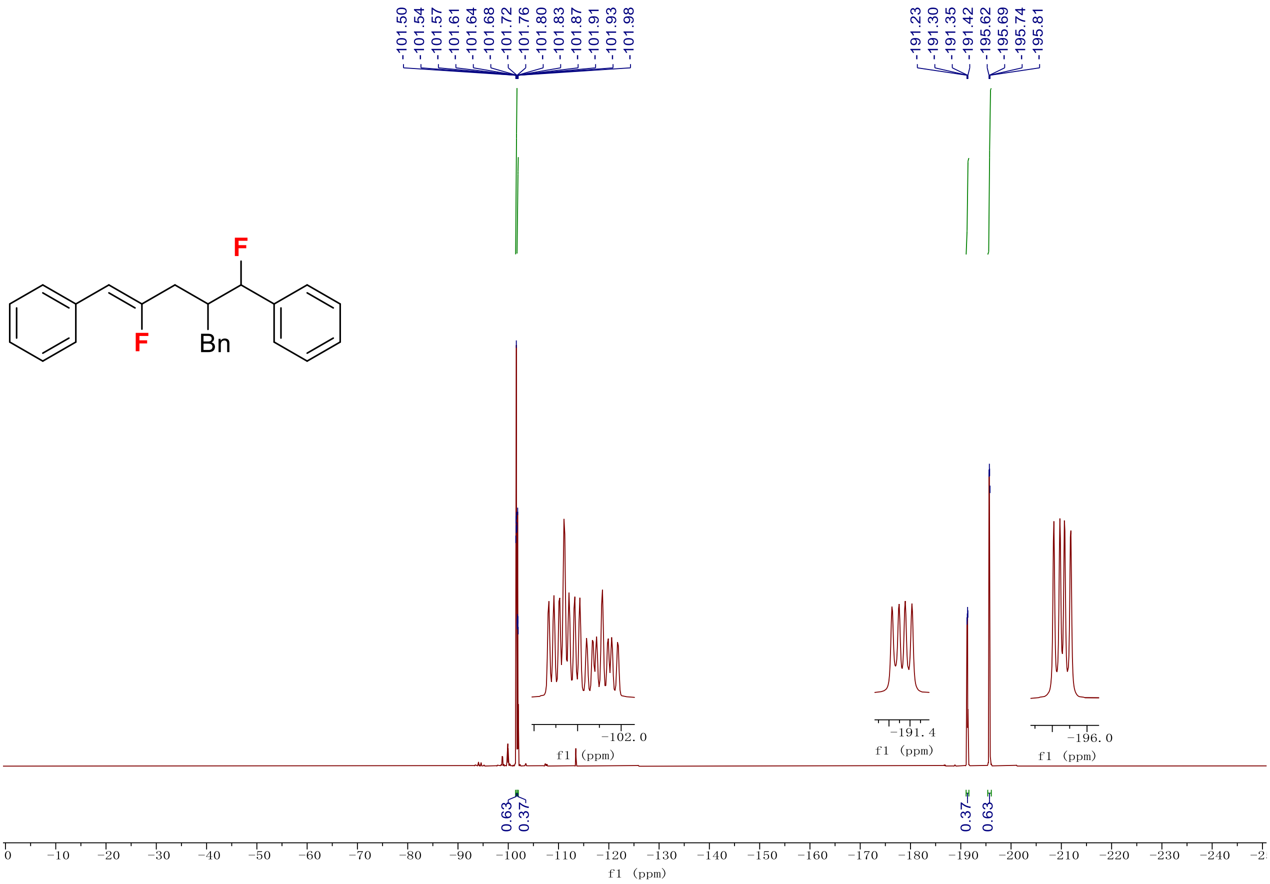

^{1}H NMR (400 MHz, CDCl3) spectrum of 3p

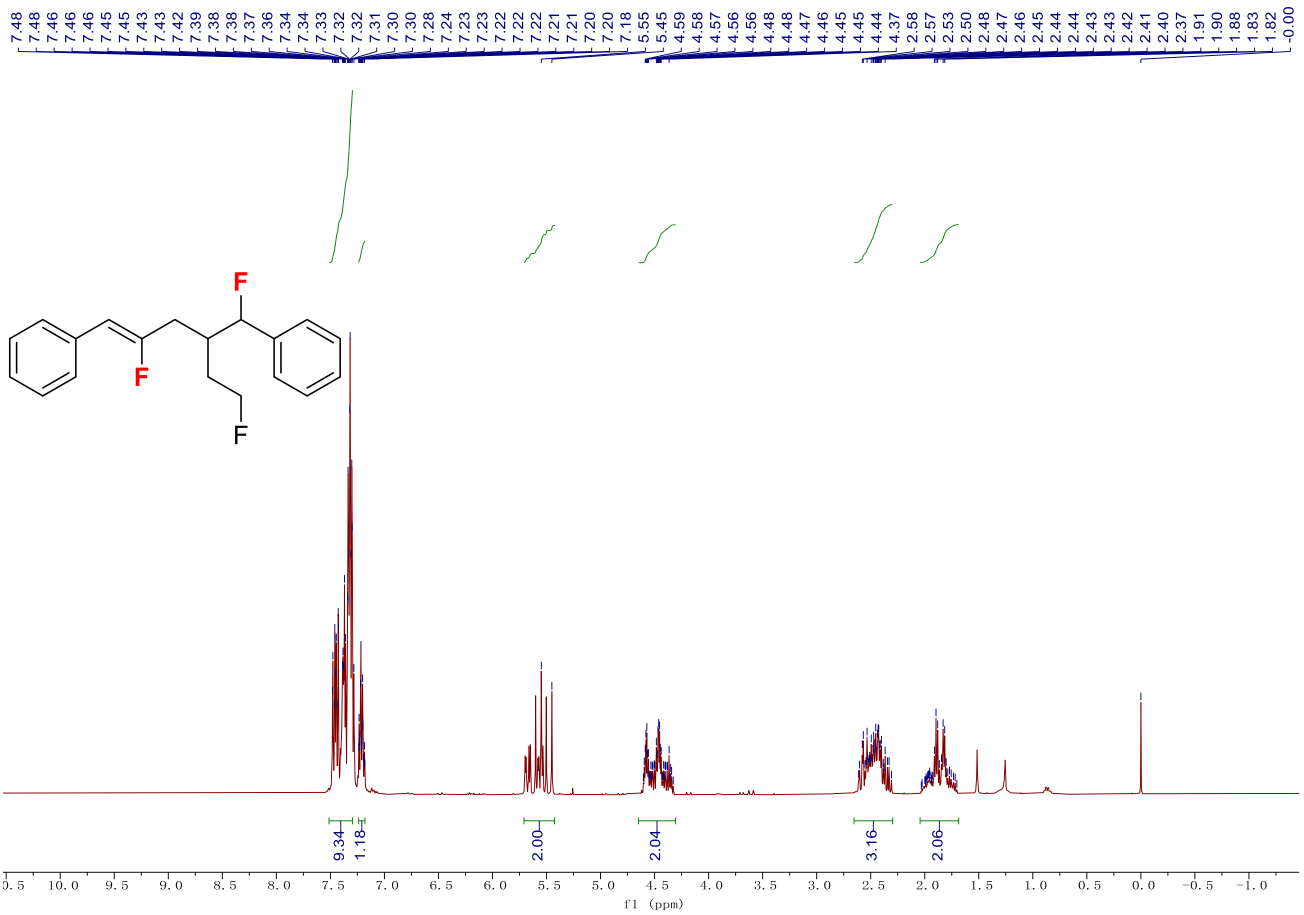

^{13}C NMR (101 MHz, CDCl3) spectrum of 3p

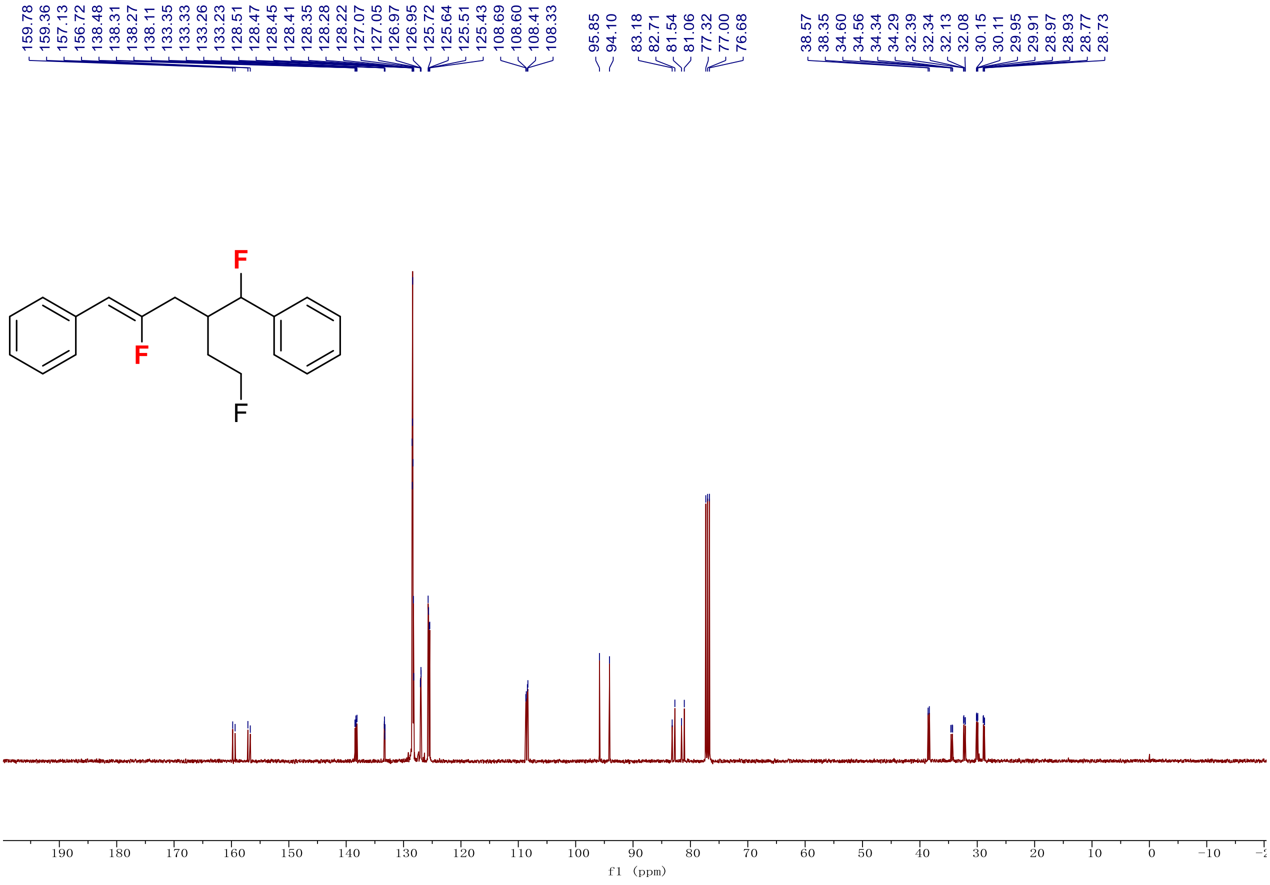

^{19}F NMR (376 MHz, CDCl3) spectrum of 3p

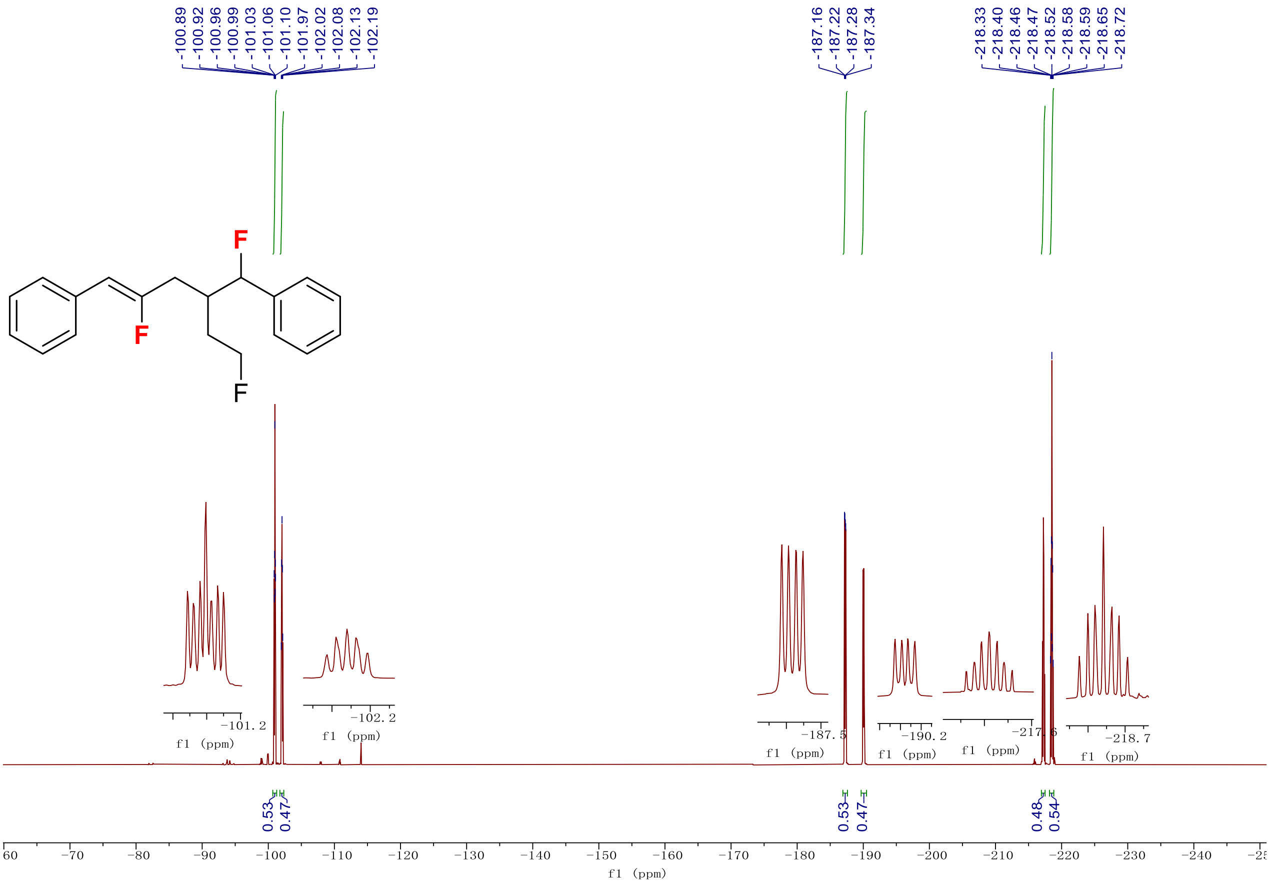

^{1}H NMR (400 MHz, $CDCl_3$) spectrum of 3q

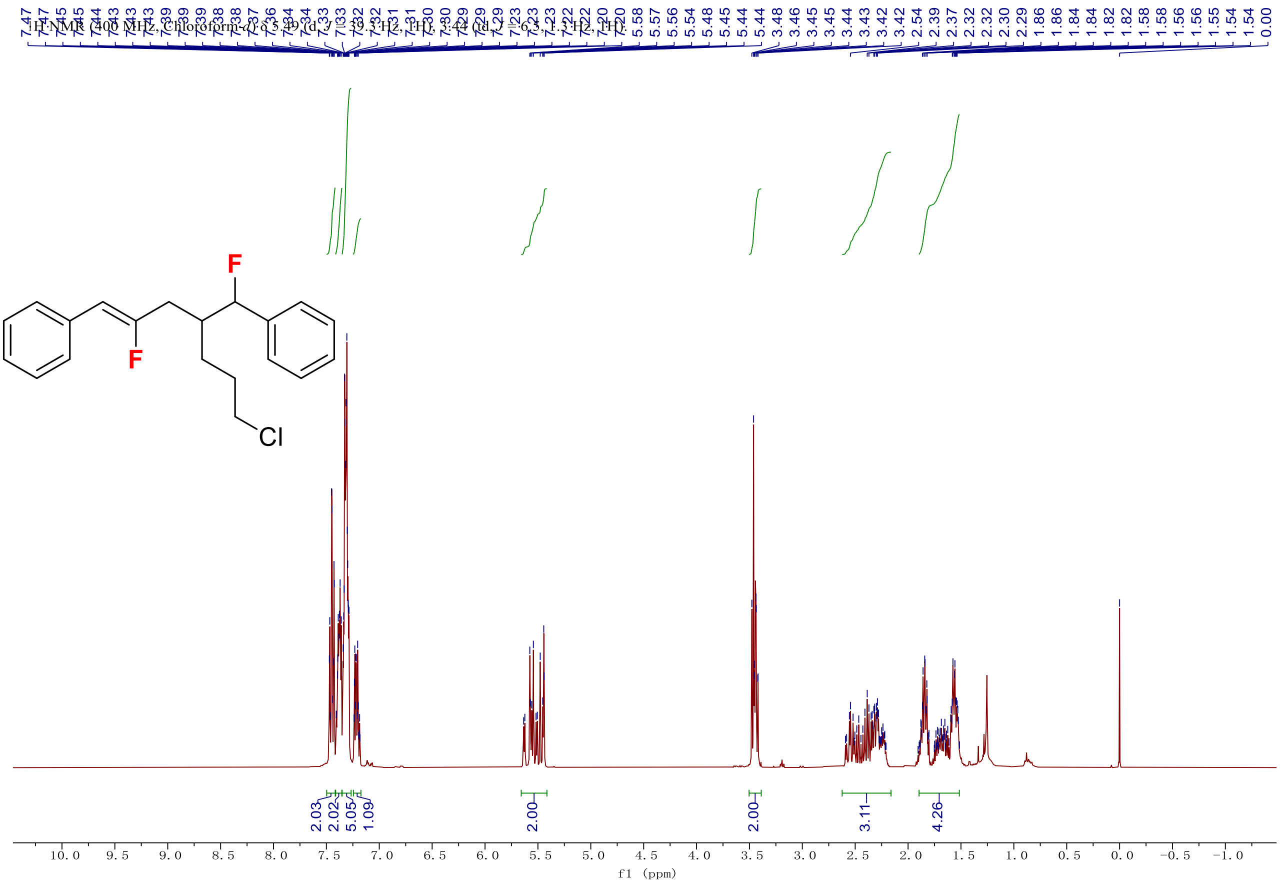

^{13}C NMR (101 MHz, $CDCl_3$) spectrum of 3q

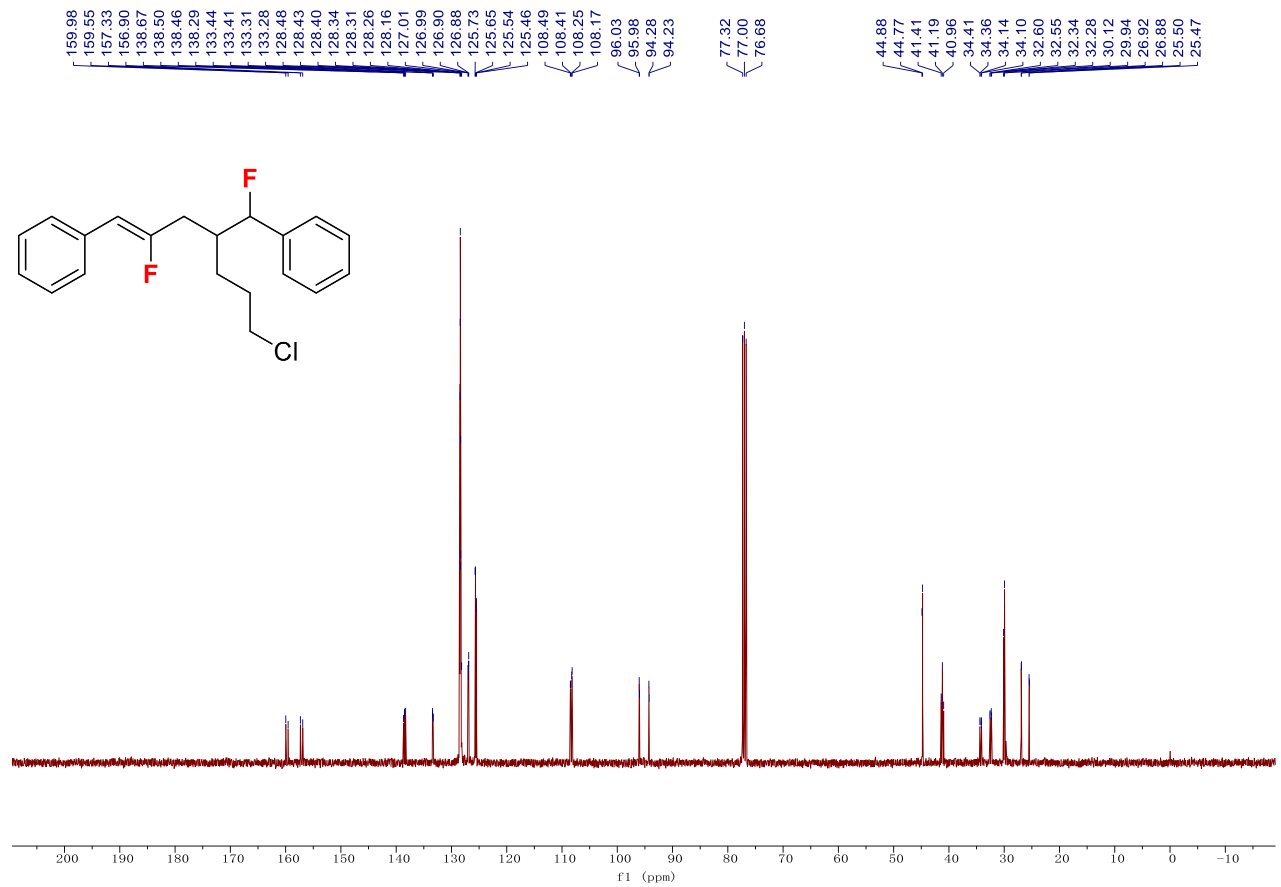

^{19}F NMR (376 MHz, $CDCl_3$) spectrum of 3q

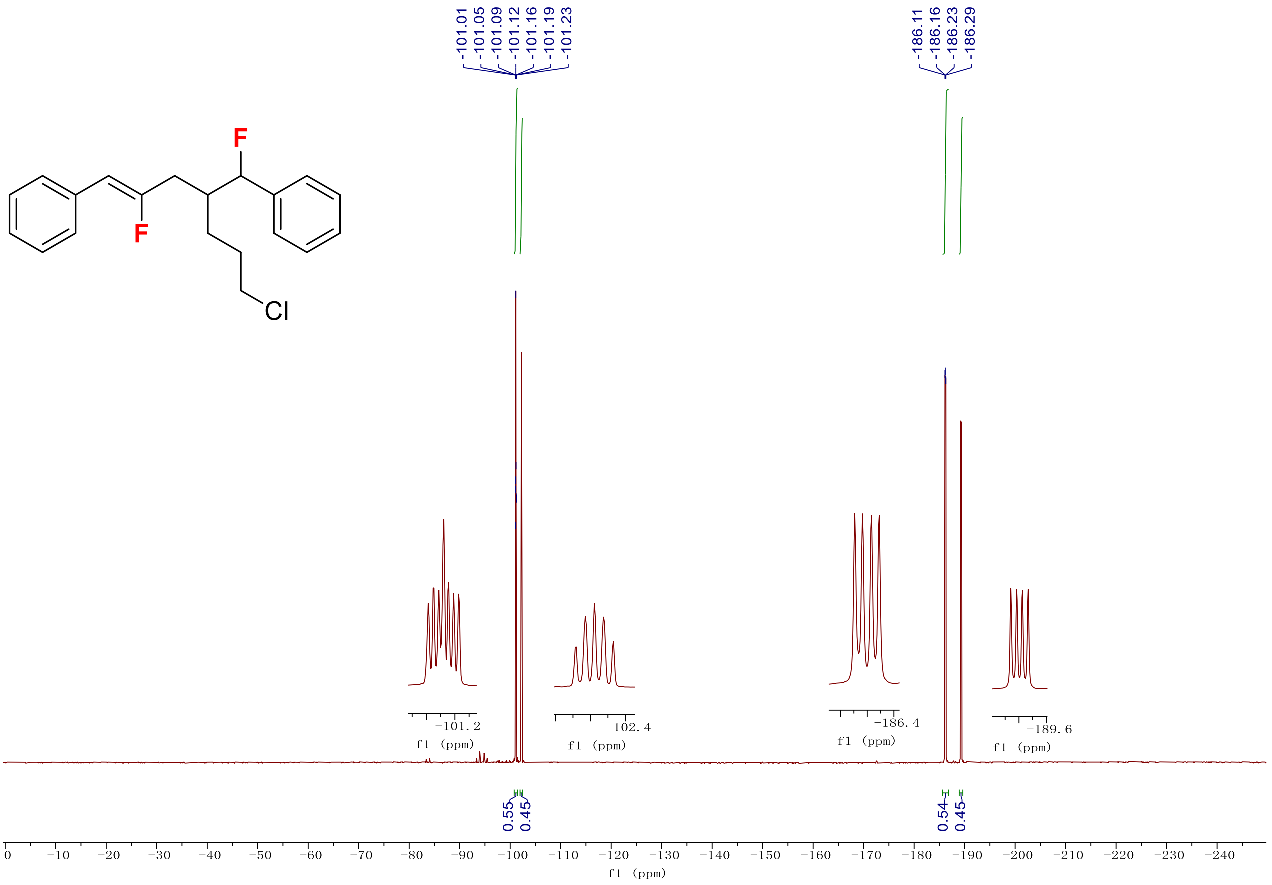

^{1}H NMR (400 MHz, $CDCl_3$) spectrum of 3r

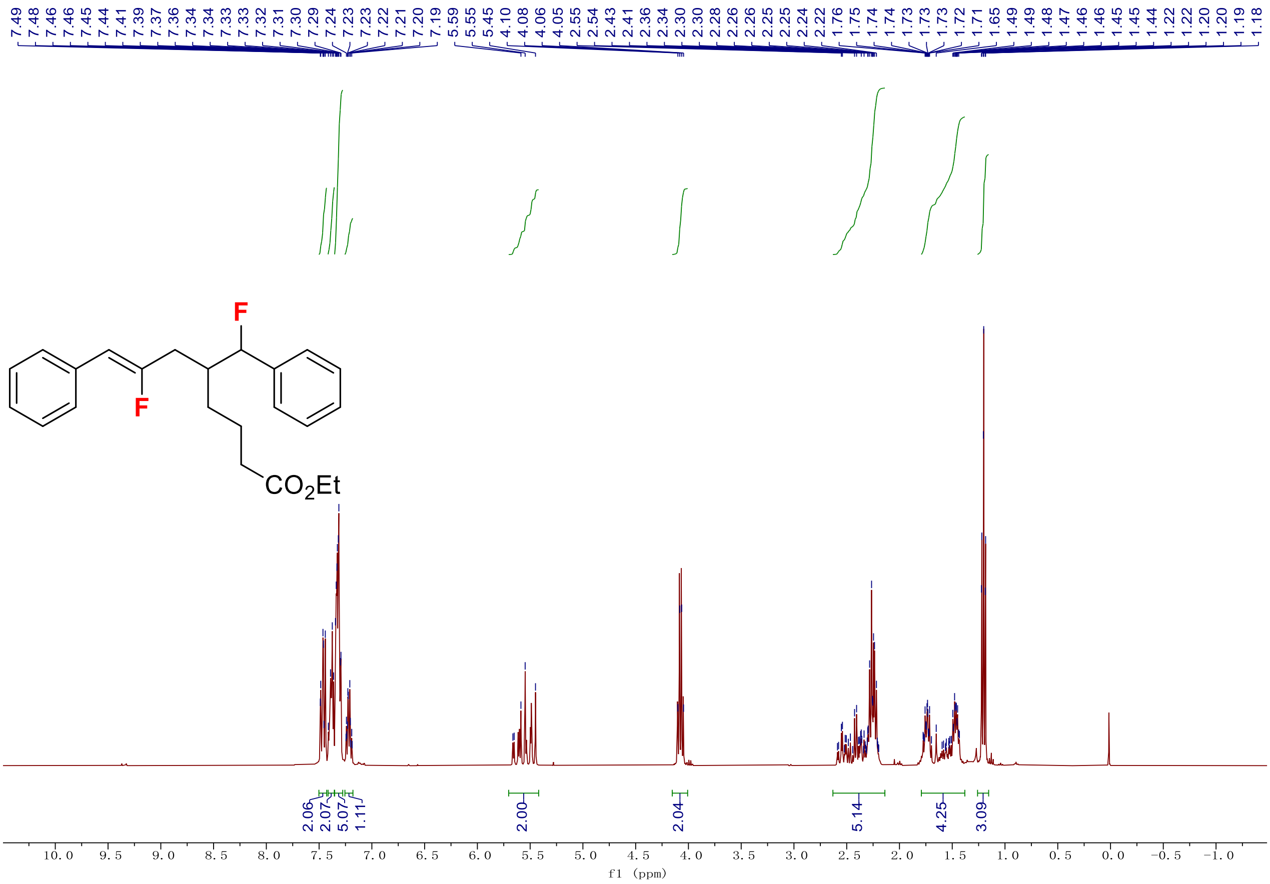

^{13}C NMR (101 MHz, $CDCl_3$) spectrum of 3r

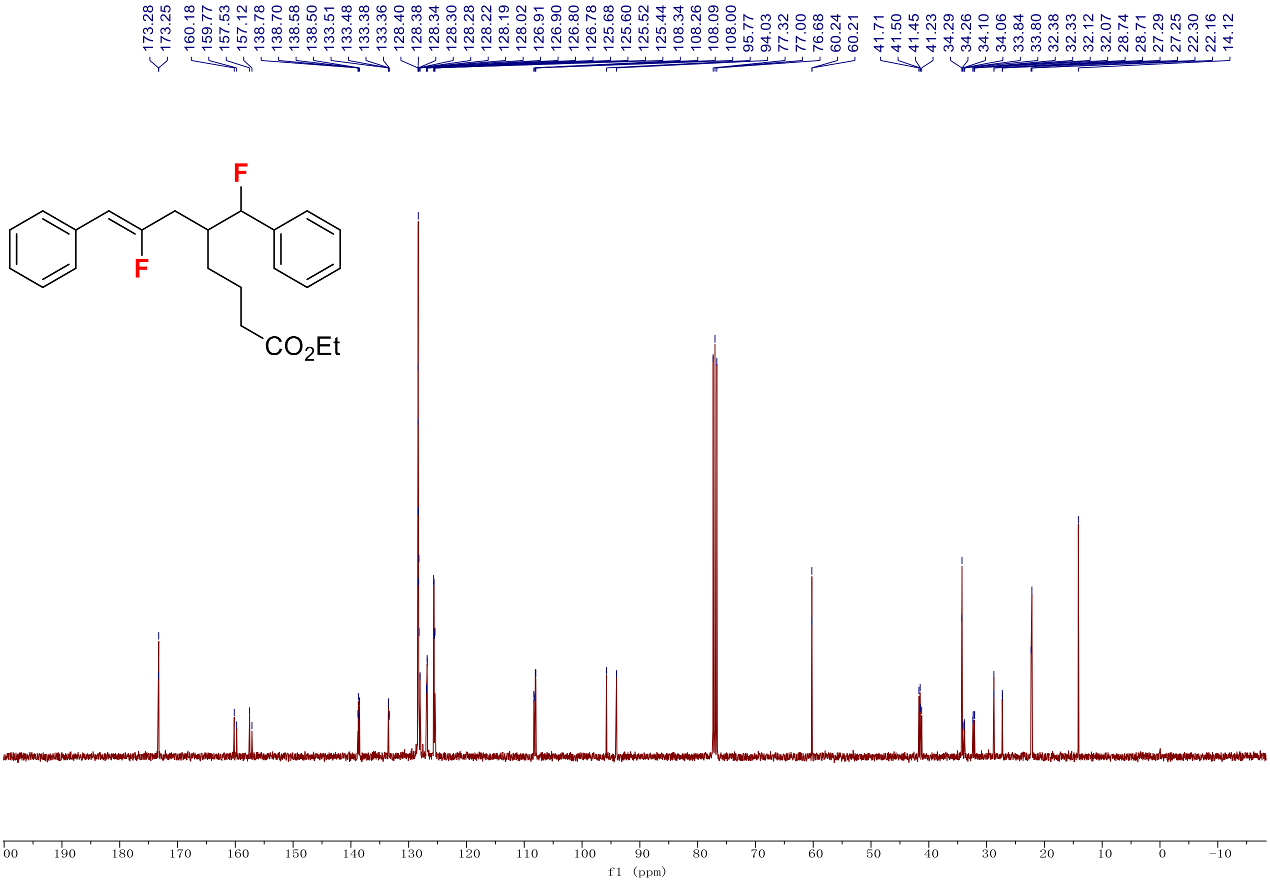

^{19}F NMR (376 MHz, CDCl3) spectrum of 3r

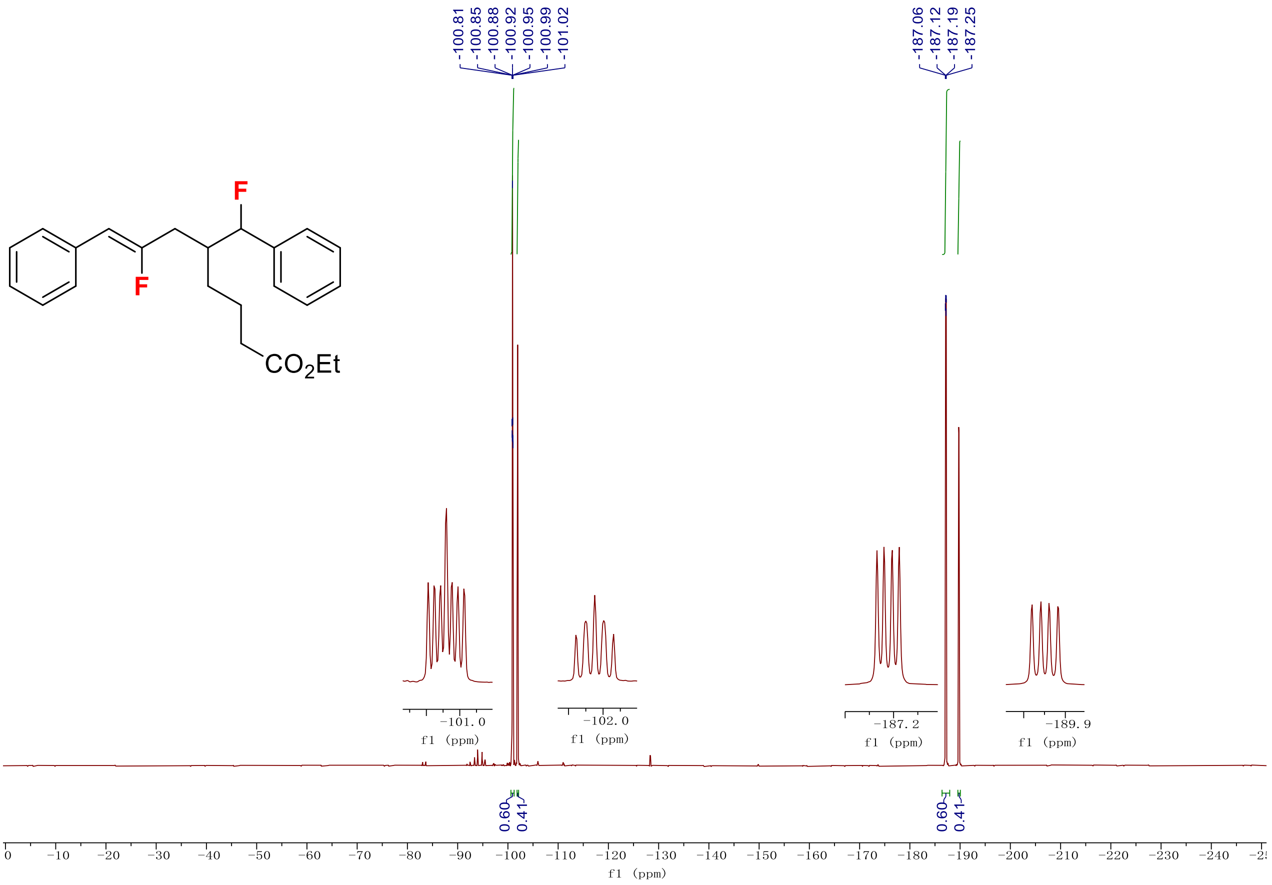

^{1}H NMR (400 MHz, CDCl$_3$) spectrum of 3s

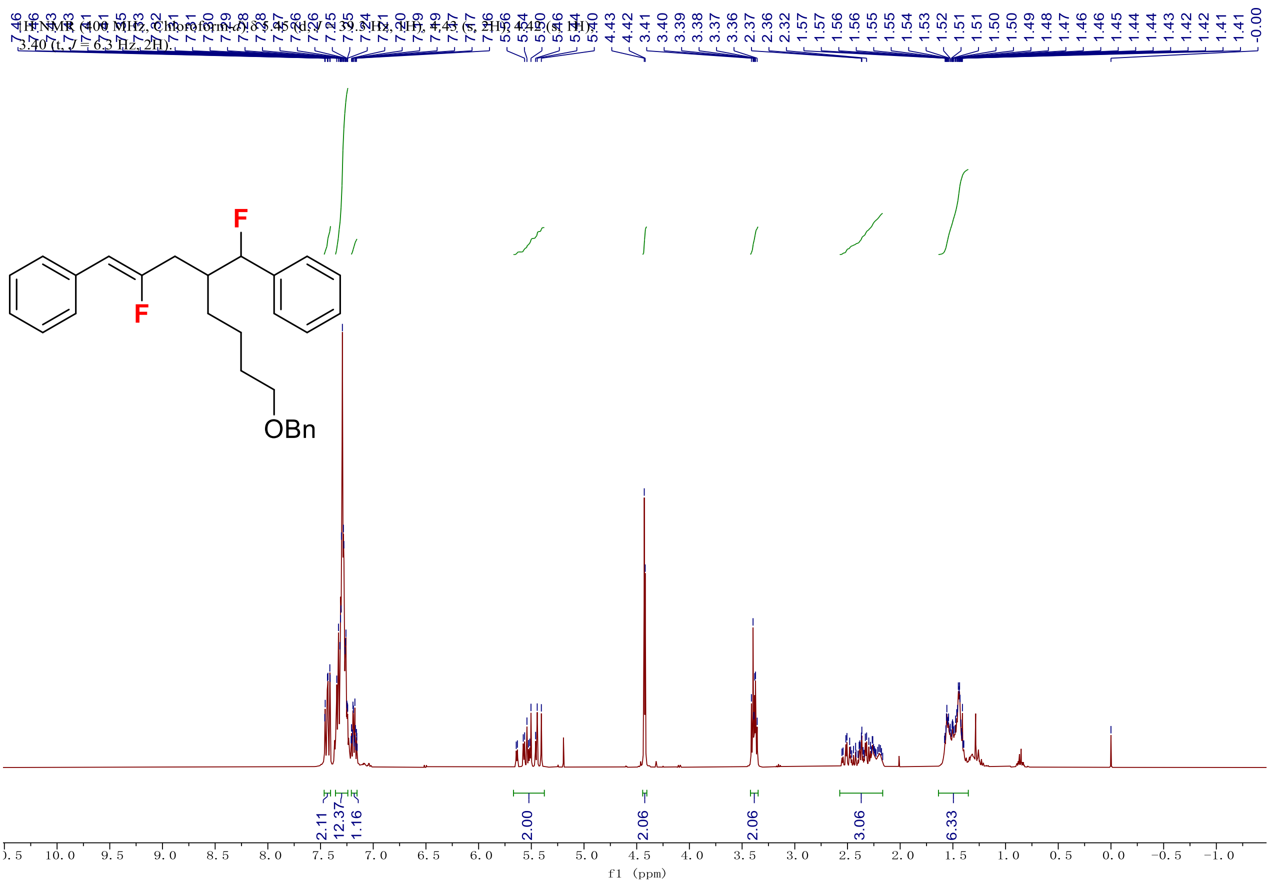

^{13}C NMR (101 MHz, CDCl$_3$) spectrum of 3s

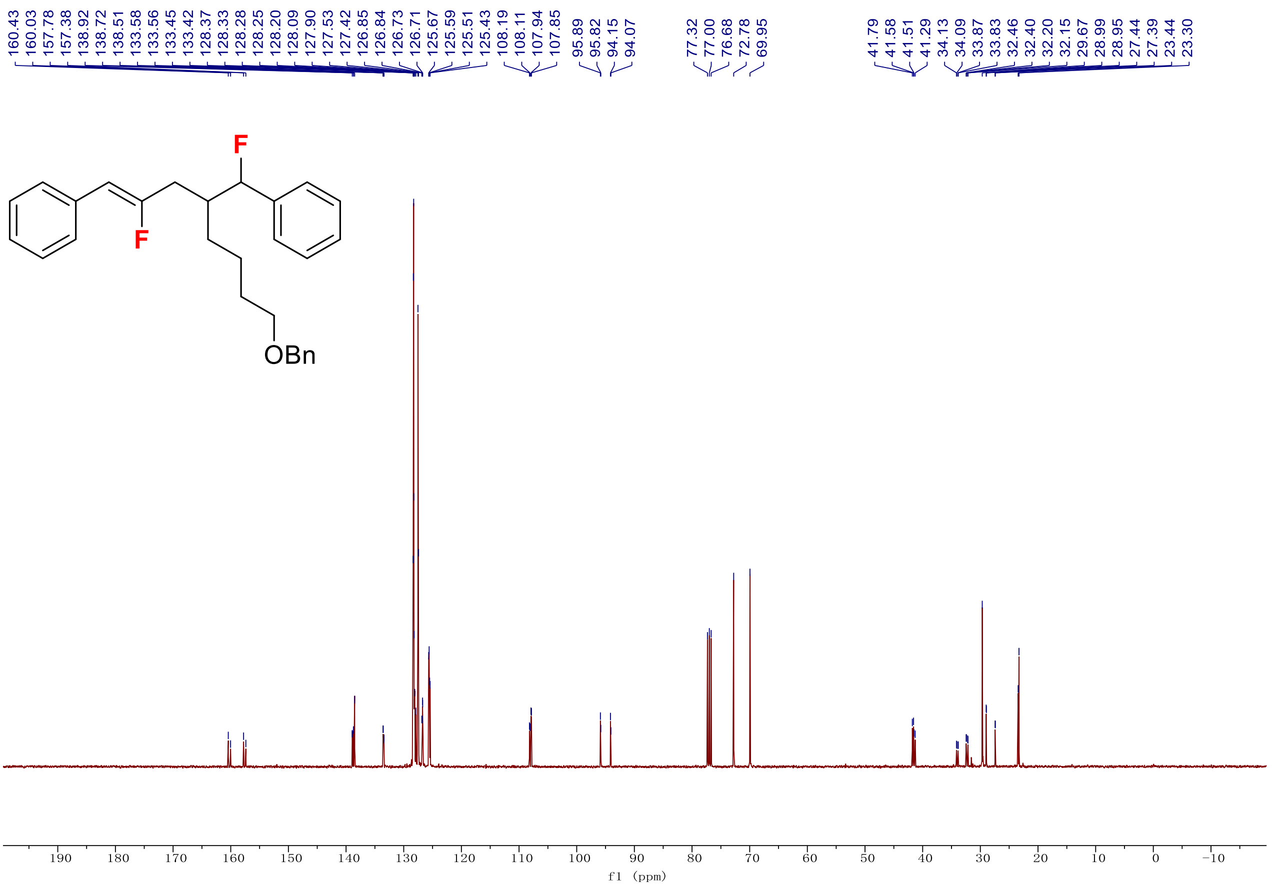

^{19}F NMR (376 MHz, $CDCl_3$) spectrum of 3s

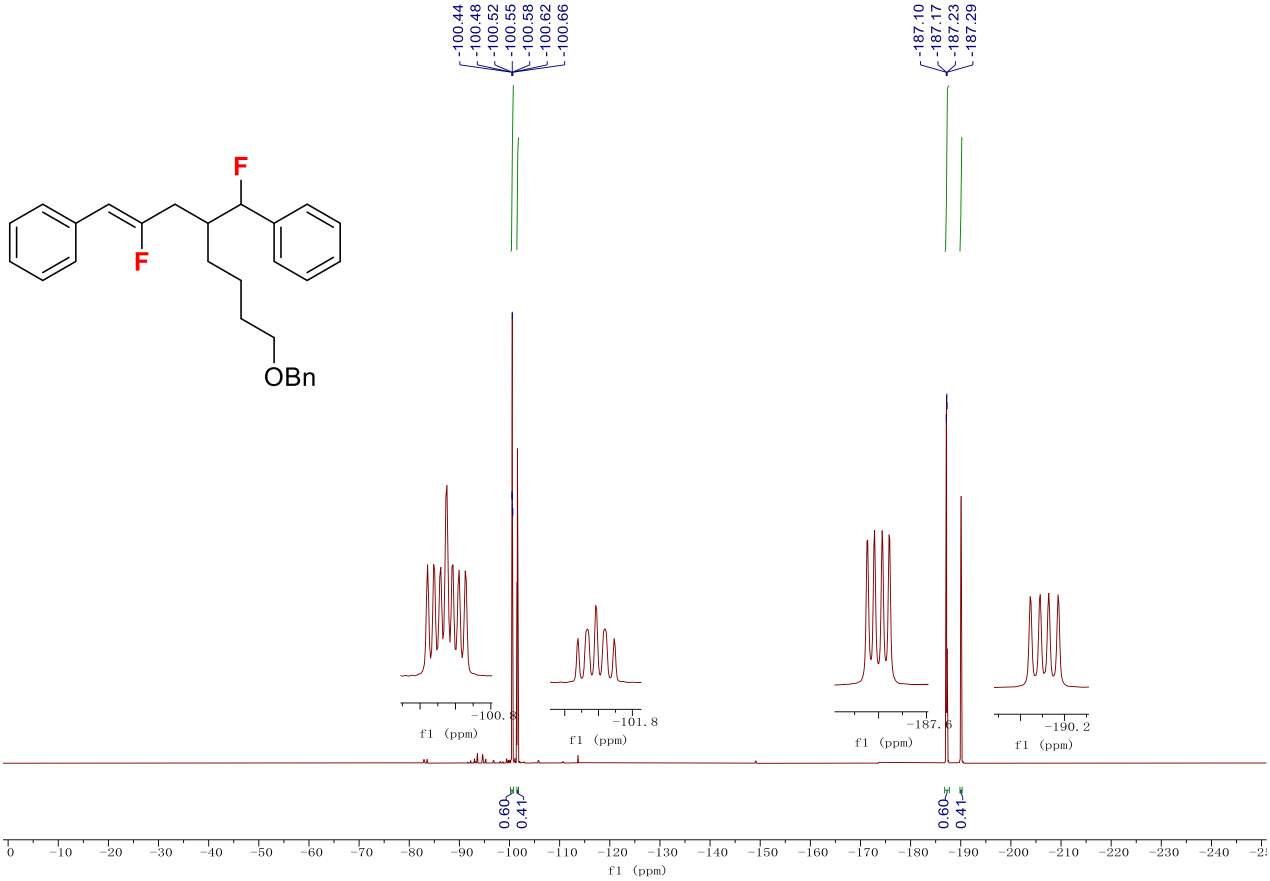

^{1}H NMR (400 MHz, CDCl3) spectrum of 3t

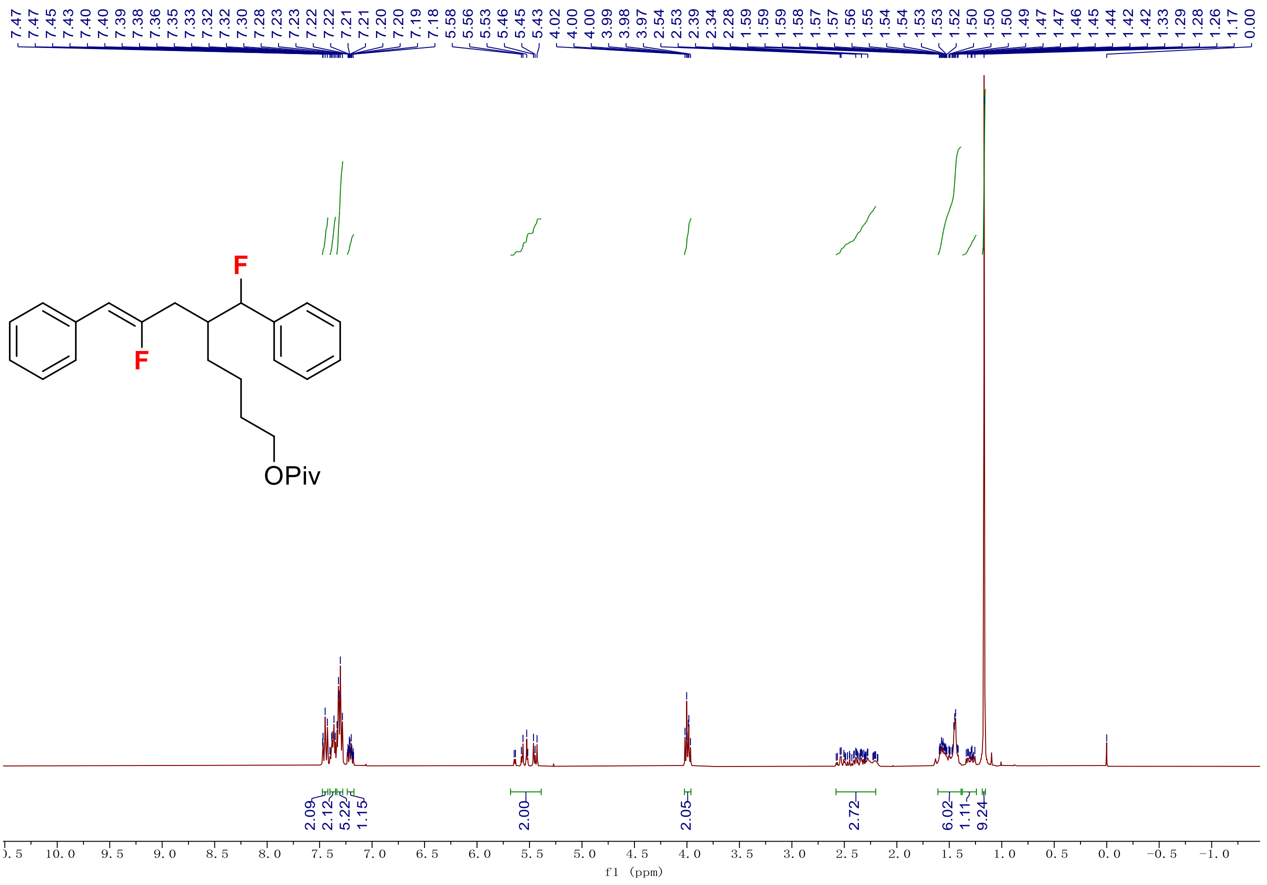

^{13}C NMR (101 MHz, CDCl3) spectrum of 3t

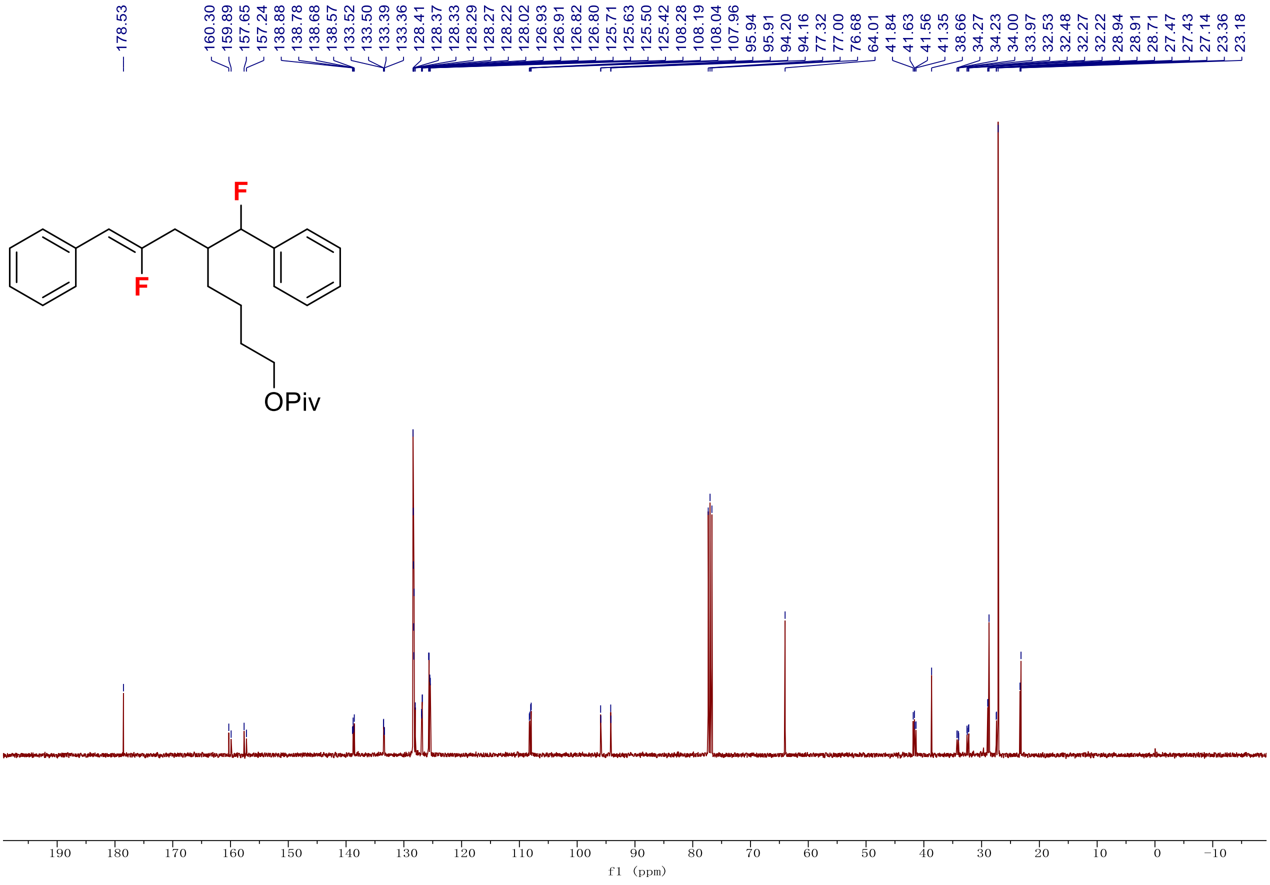

^{19}F NMR (376 MHz, $CDCl_3$) spectrum of 3t

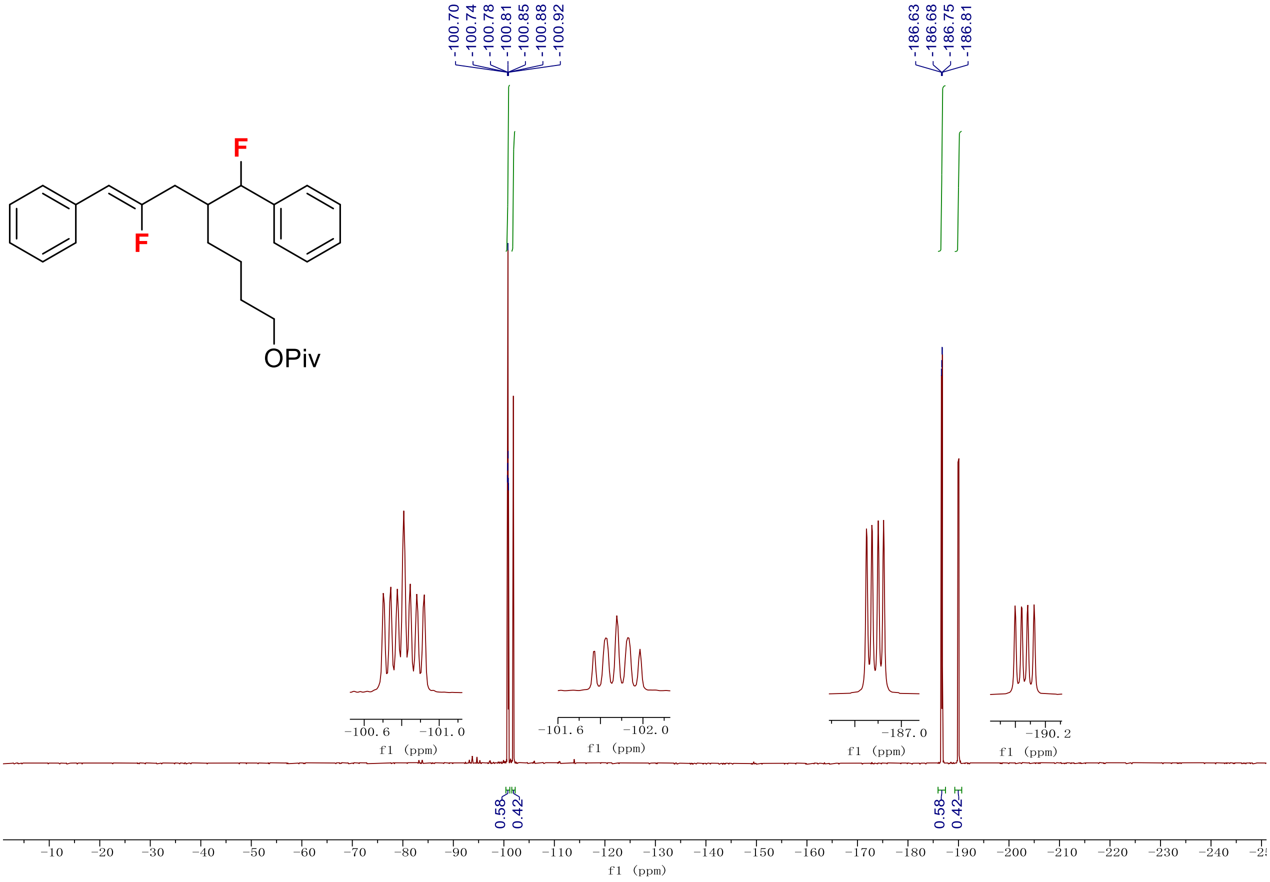

^{1}H NMR (400 MHz, $CDCl_3$) spectrum of 3u

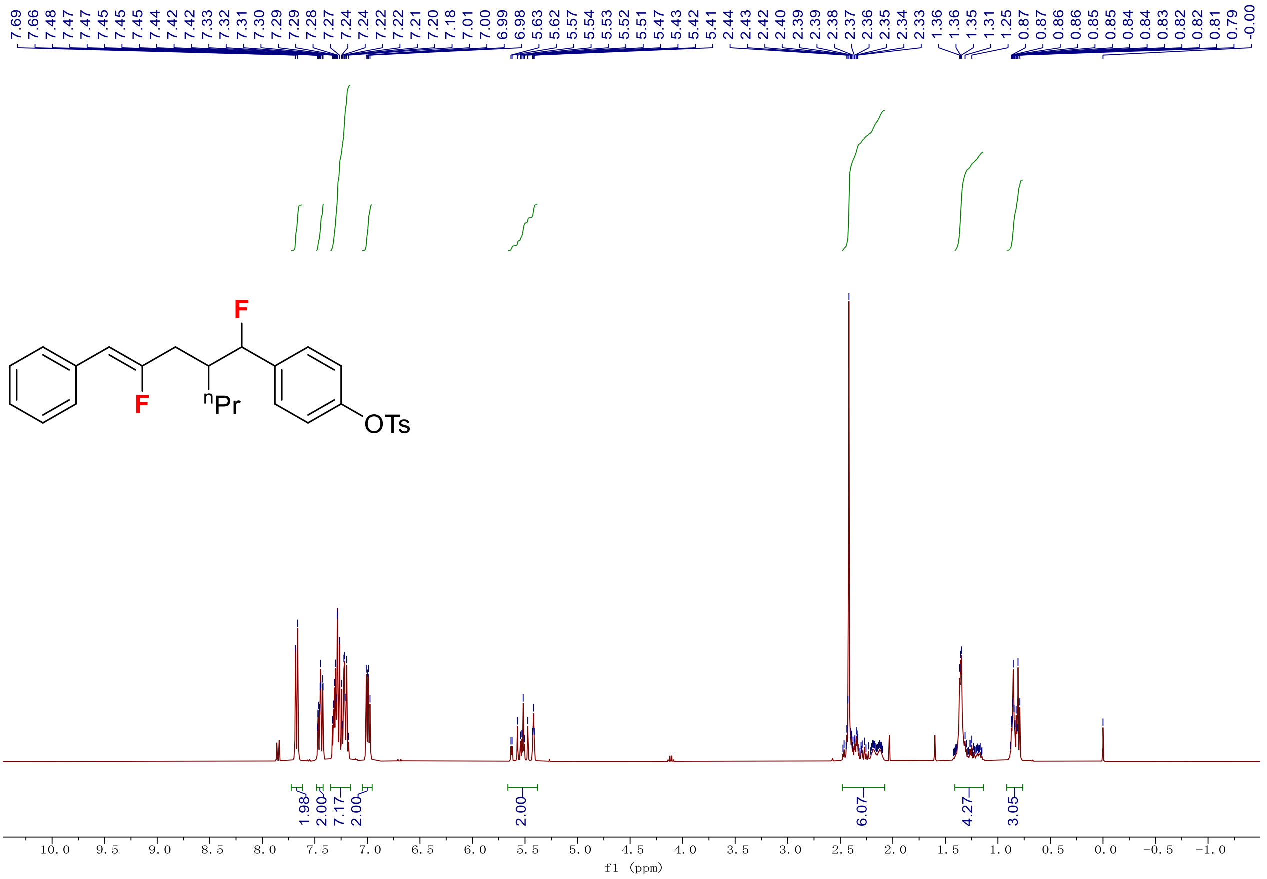

^{13}C NMR (101 MHz, $CDCl_3$) spectrum of 3u

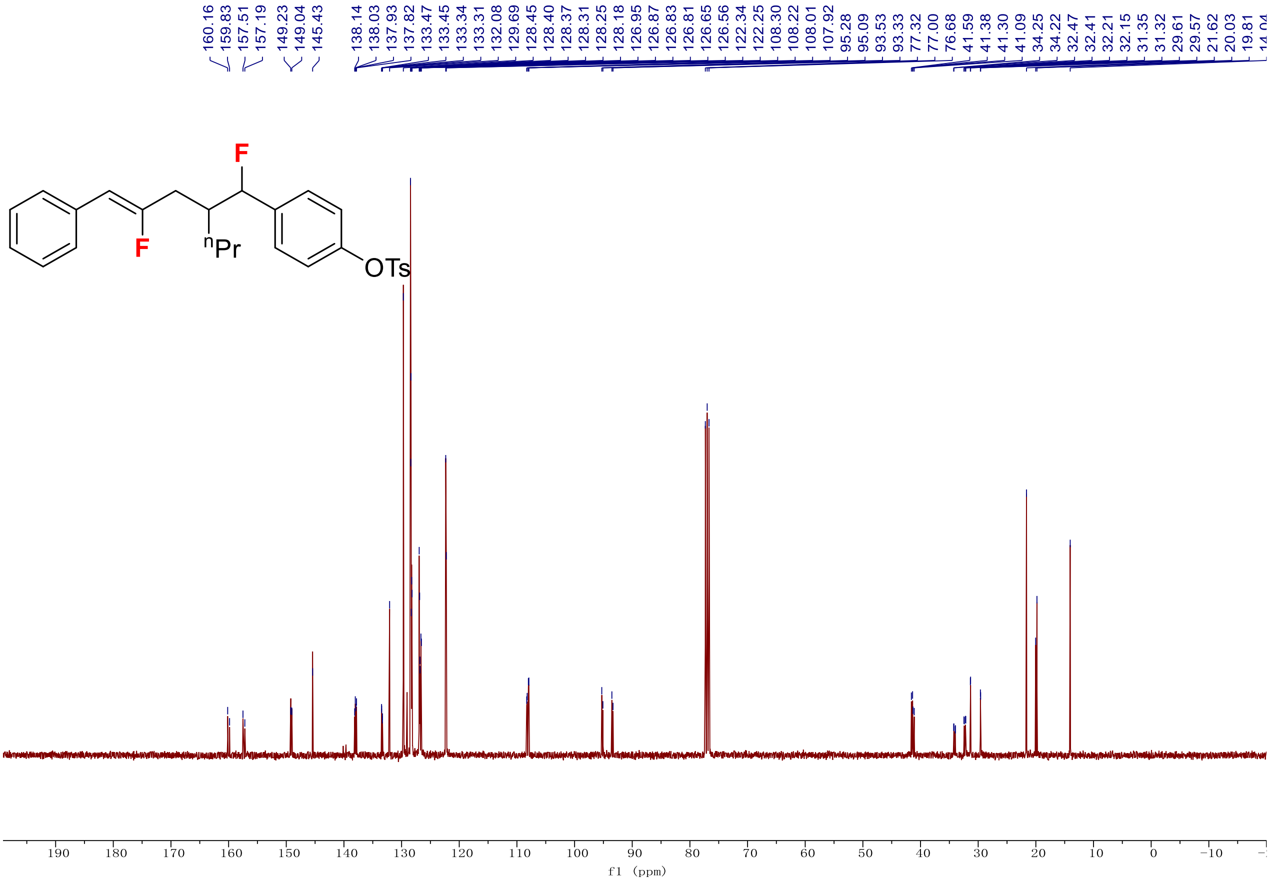

^{19}F NMR (376 MHz, CDCl3) spectrum of 3u

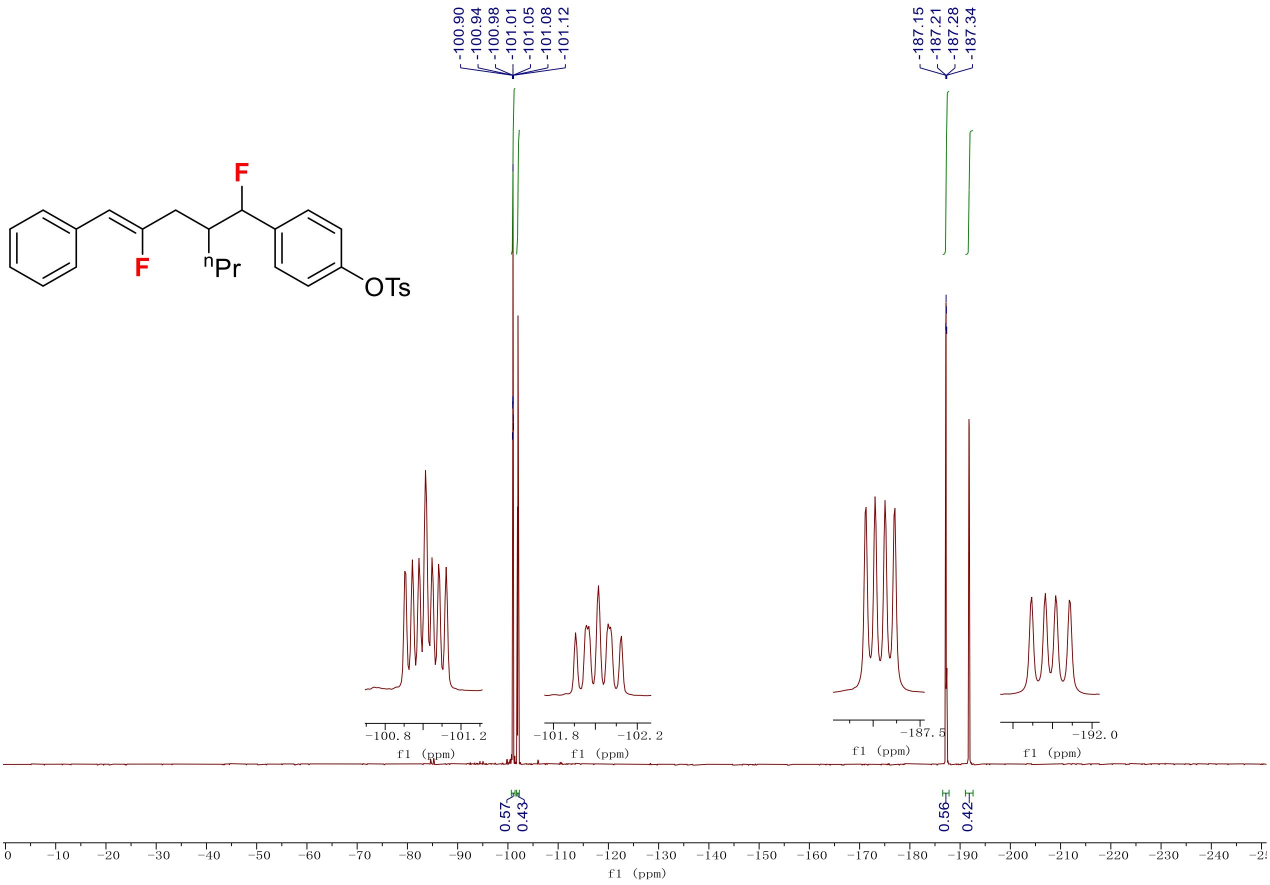

^{1}H NMR (400 MHz, $CDCl_3$) spectrum of 3v

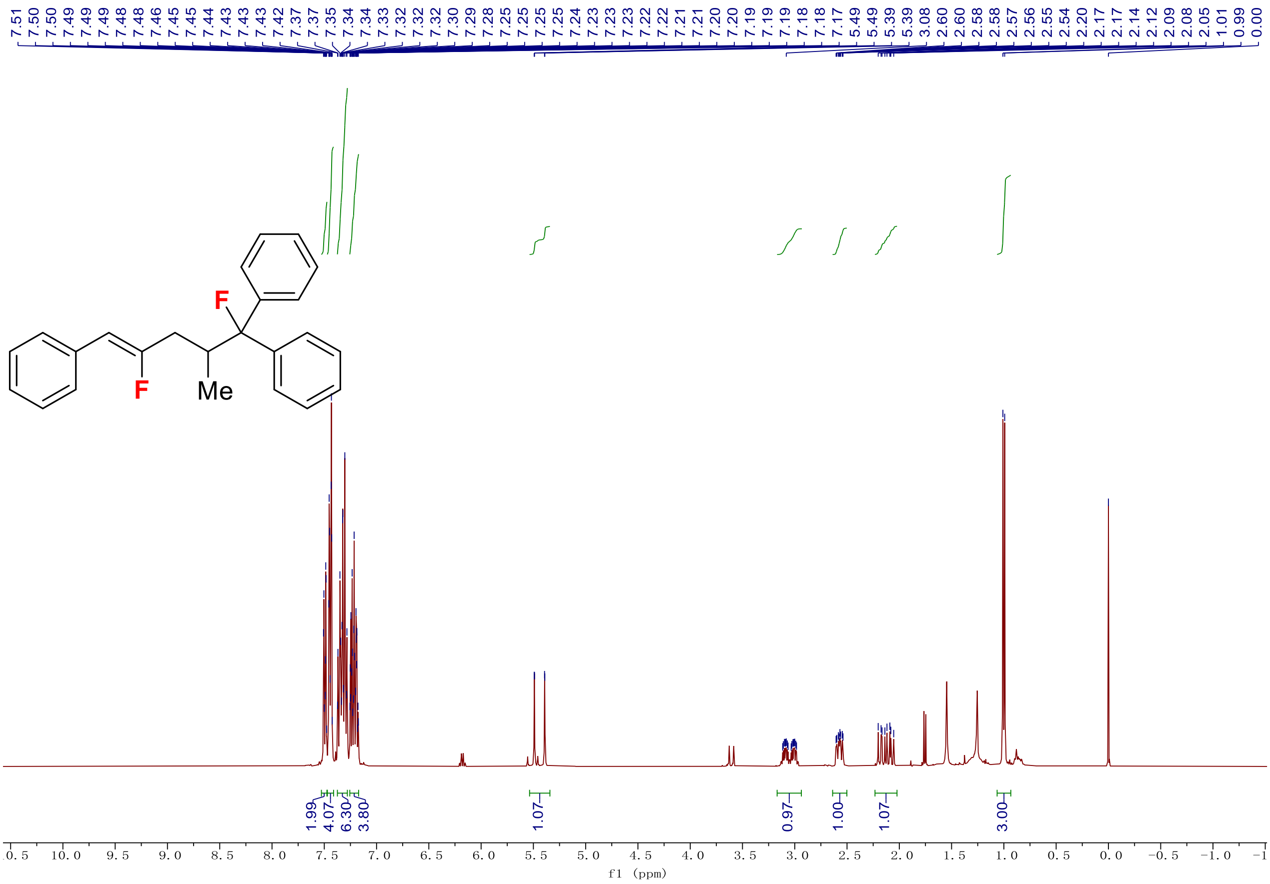

^{13}C NMR (101 MHz, $CDCl_3$) spectrum of 3v

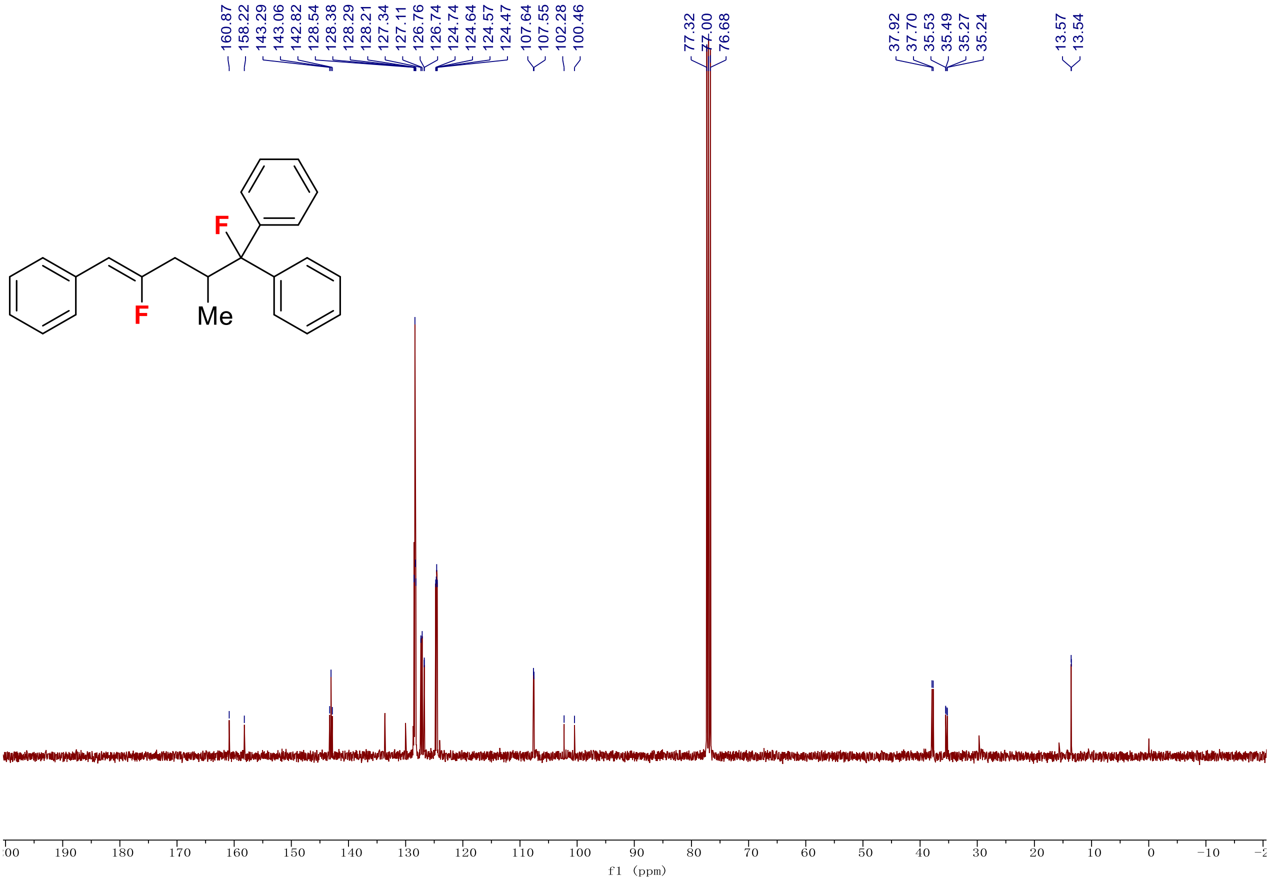

^{19}F NMR (376 MHz, CDCl3) spectrum of 3v

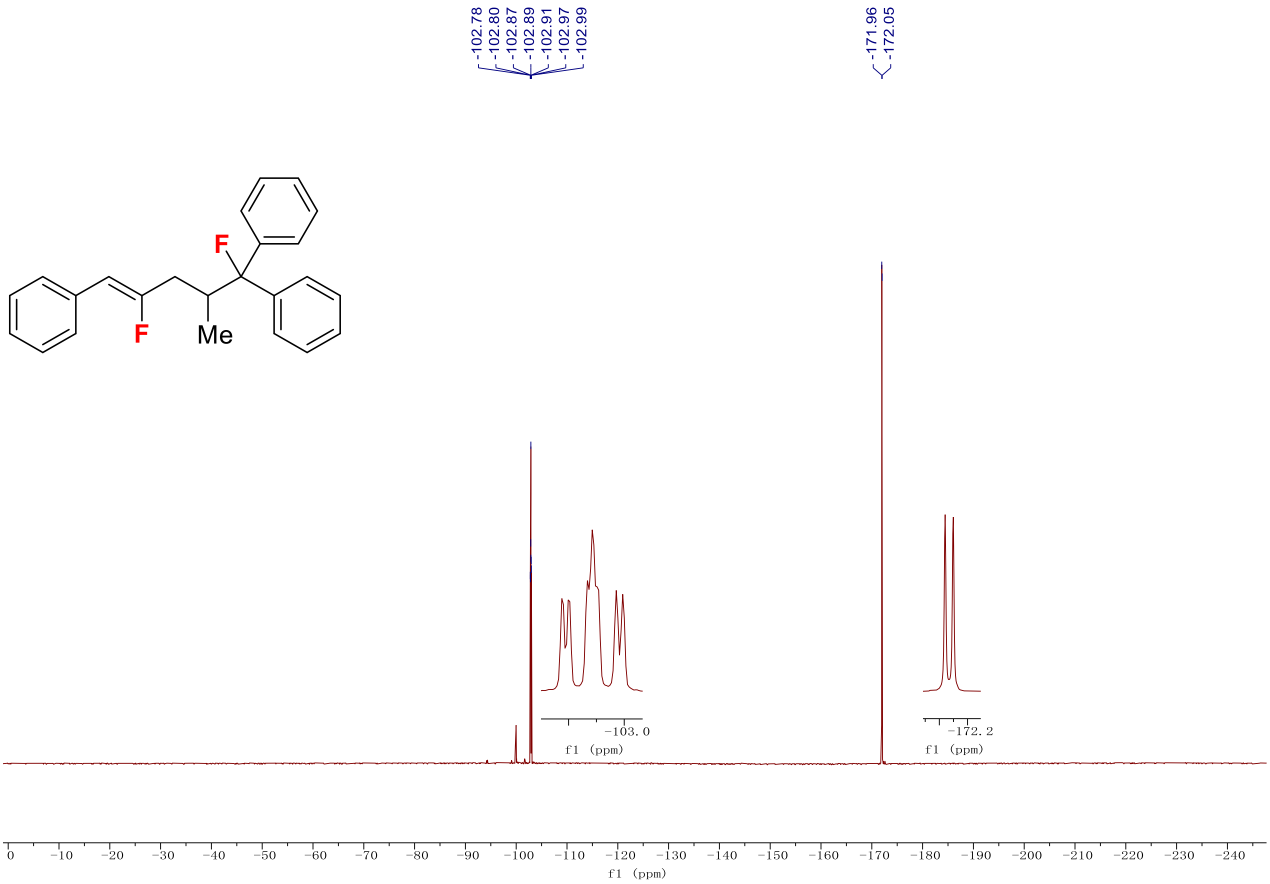

^{1}H NMR (400 MHz, CDCl3) spectrum of 3w

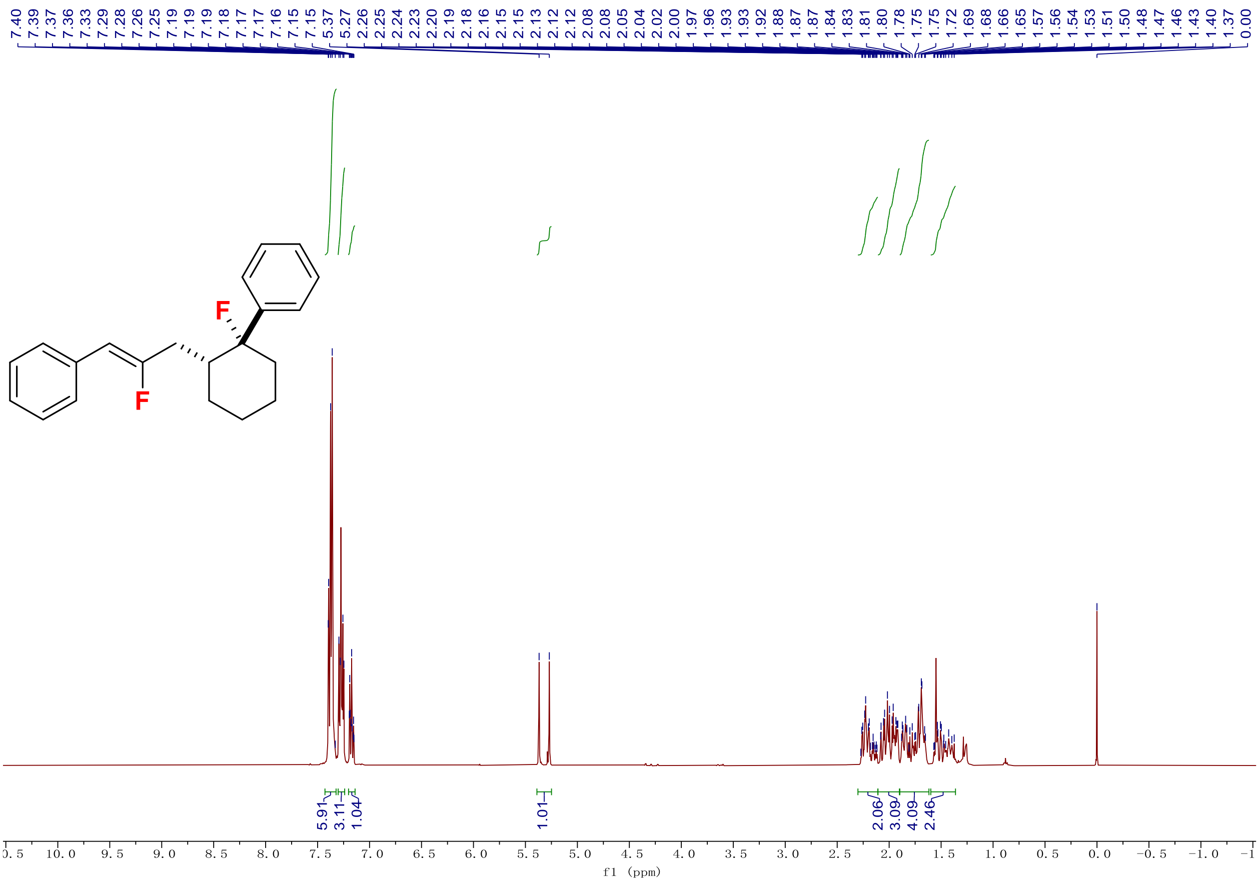

^{13}C NMR (101 MHz, CDCl3) spectrum of 3w

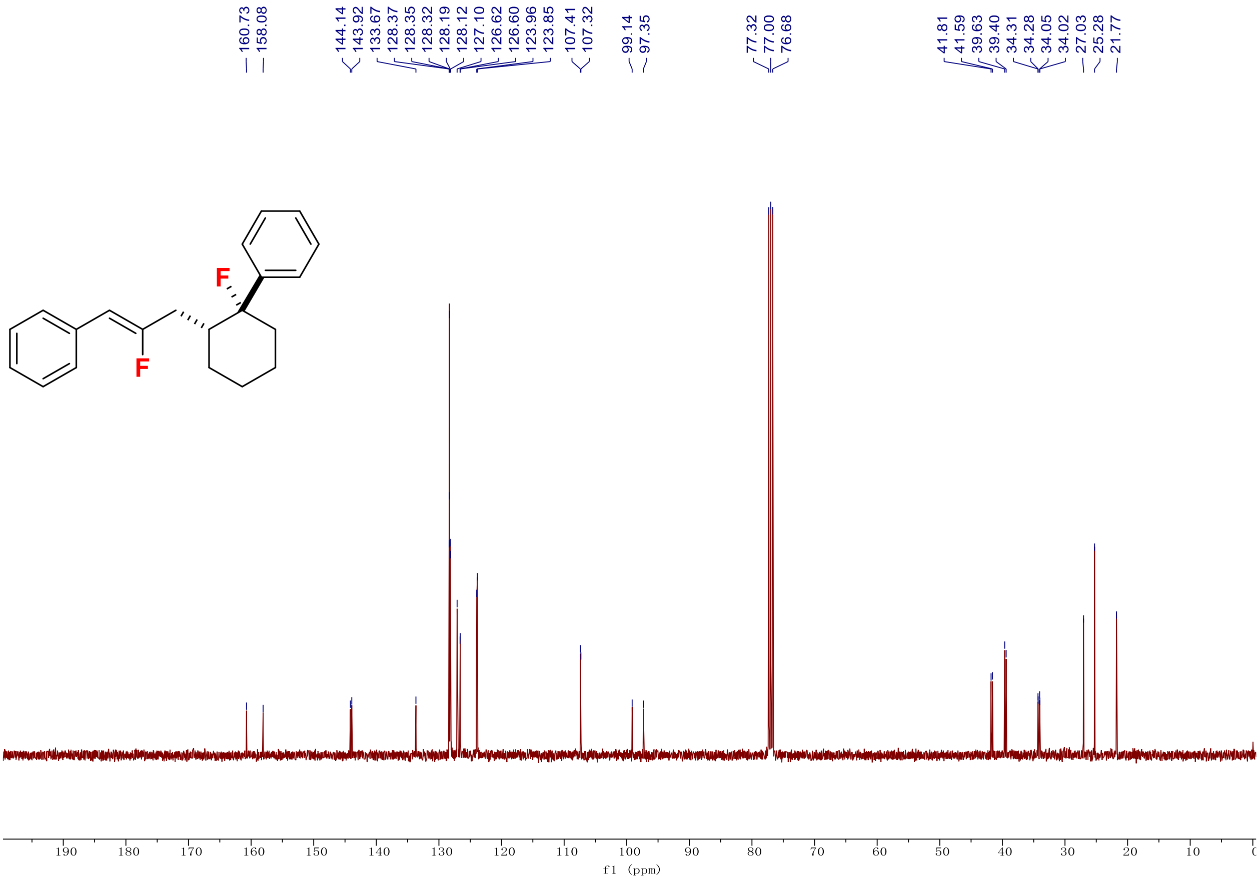

^{19}F NMR (376 MHz, CDCl$_3$) spectrum of 3w

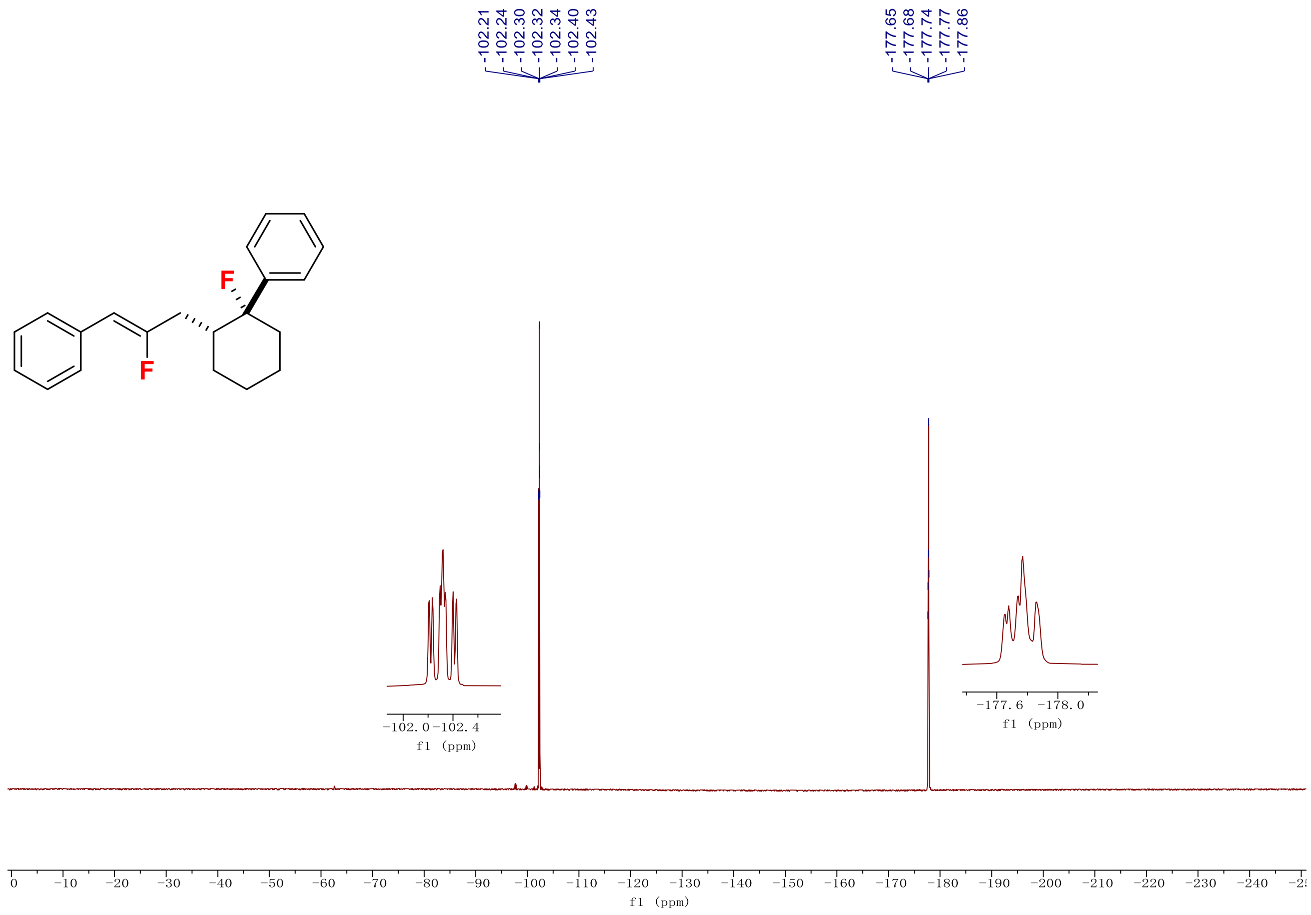

^{1}H NMR (400 MHz, $CDCl_3$) spectrum of 3x

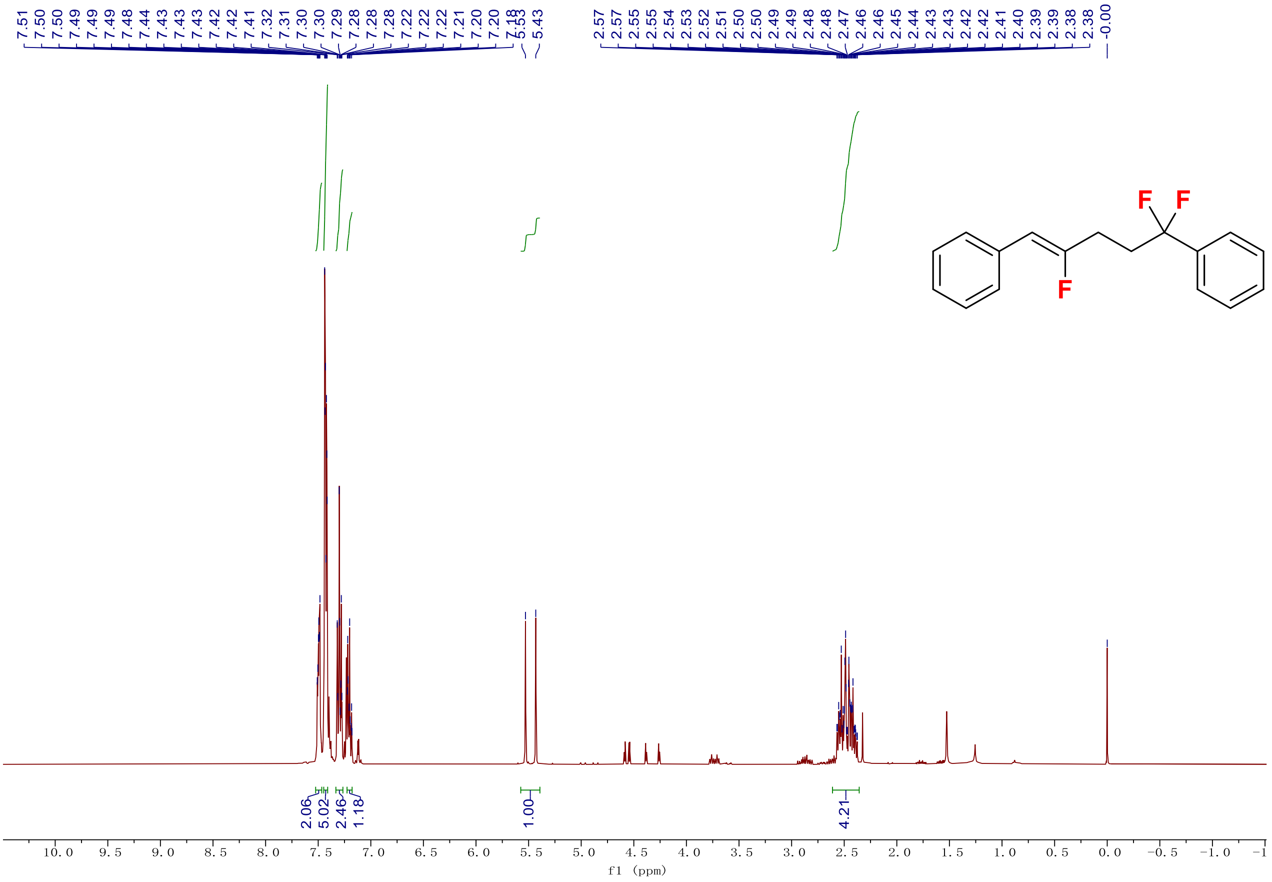

^{13}C NMR (101 MHz, $CDCl_3$) spectrum of 3x

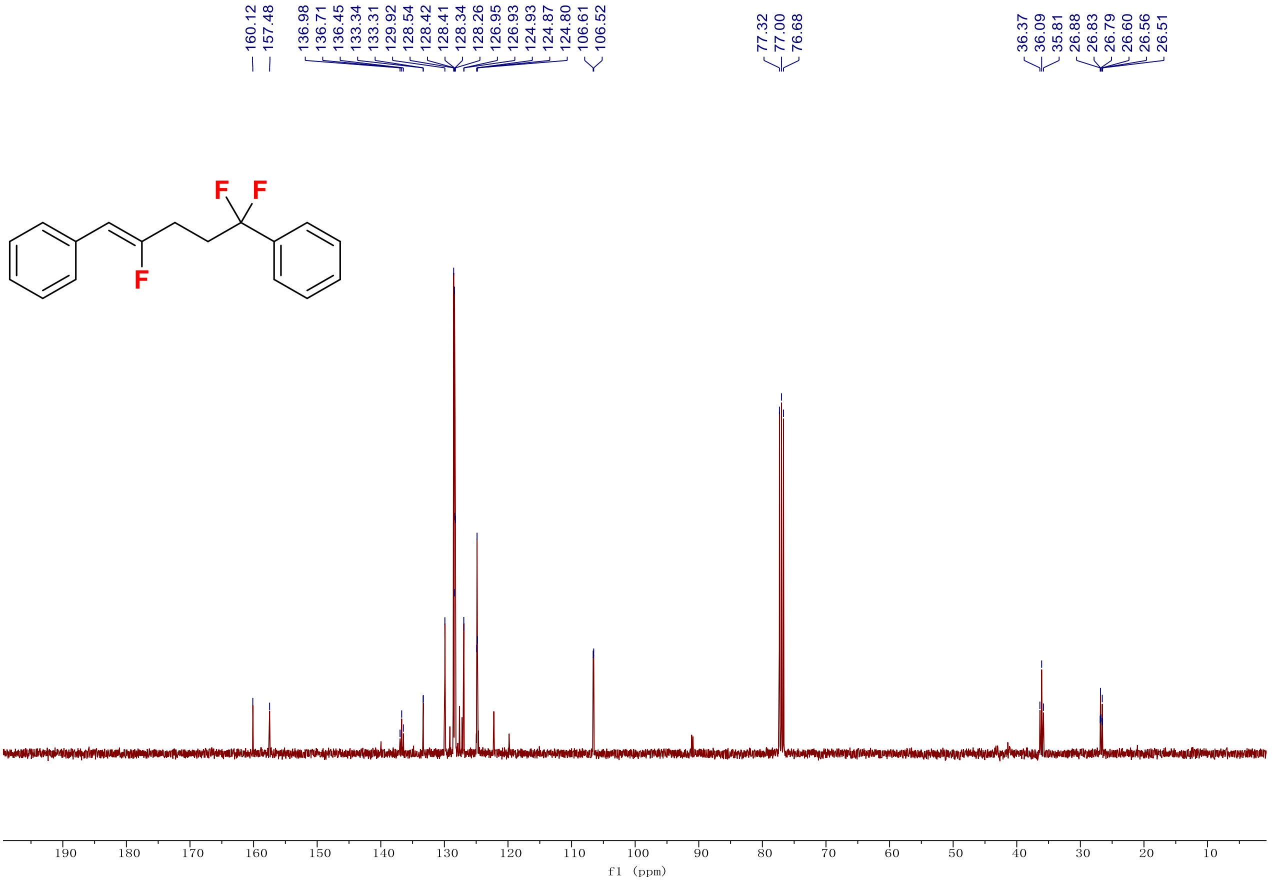

^{19}F NMR (376 MHz, CDCl₃) spectrum of 3x

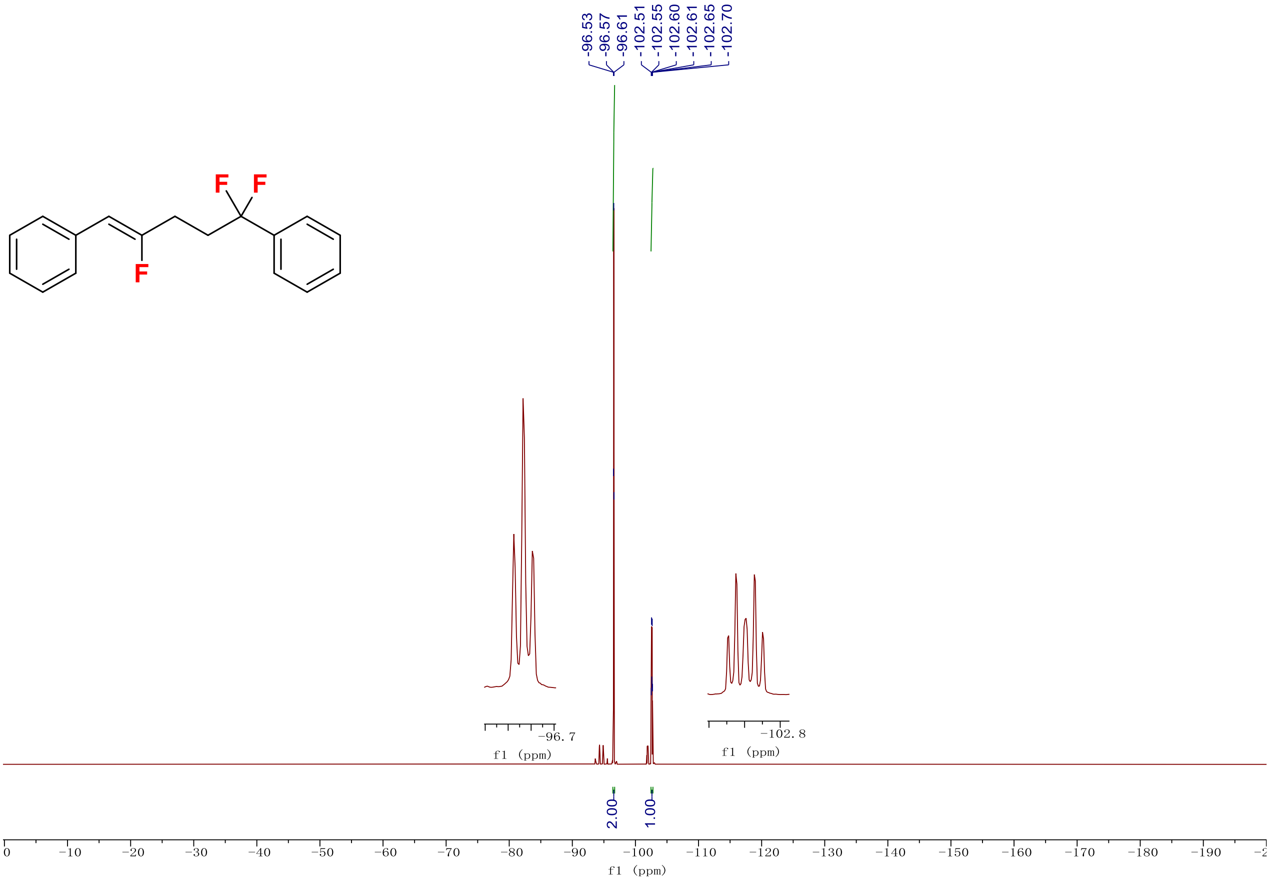

^{1}H NMR (400 MHz, $CDCl_3$) spectrum of 3y

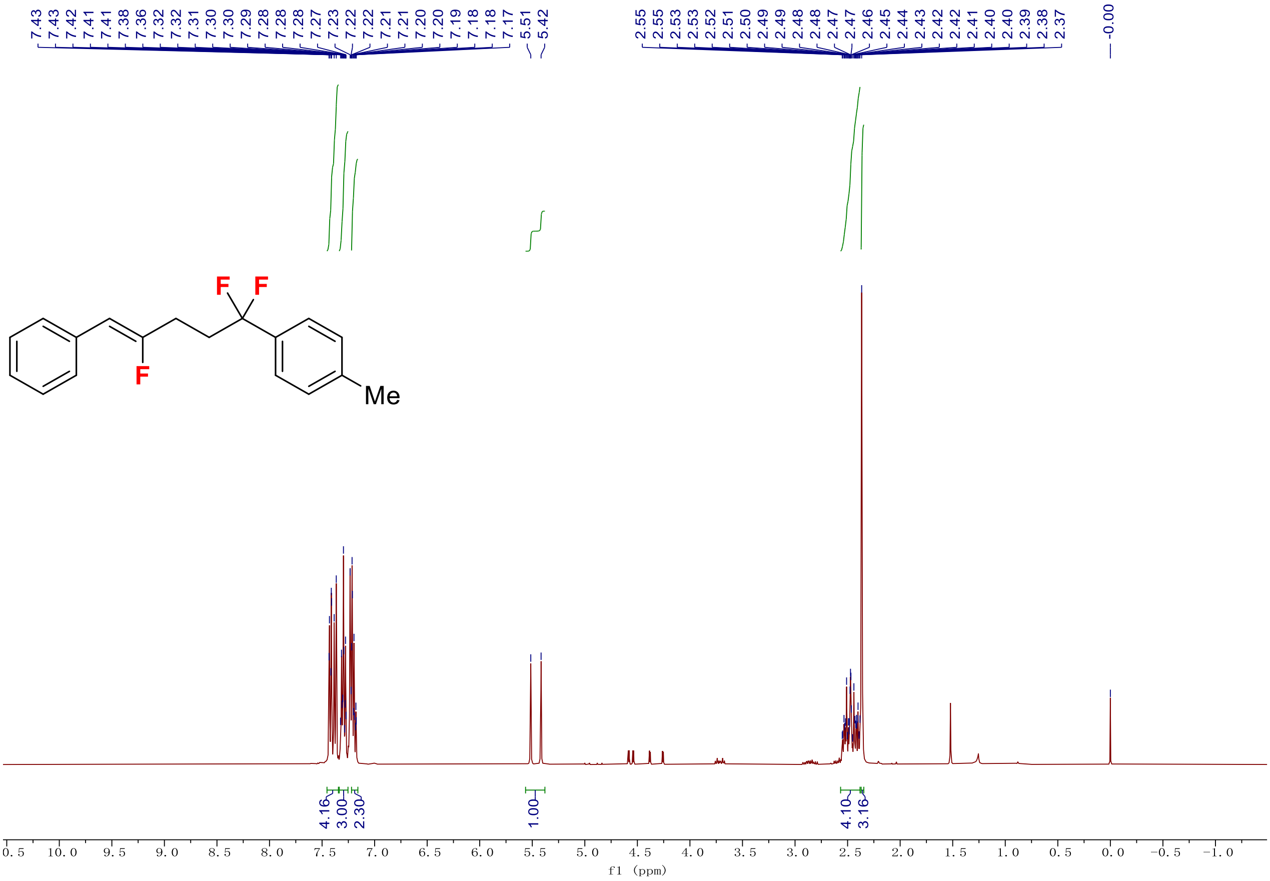

^{13}C NMR (101 MHz, $CDCl_3$) spectrum of 3y

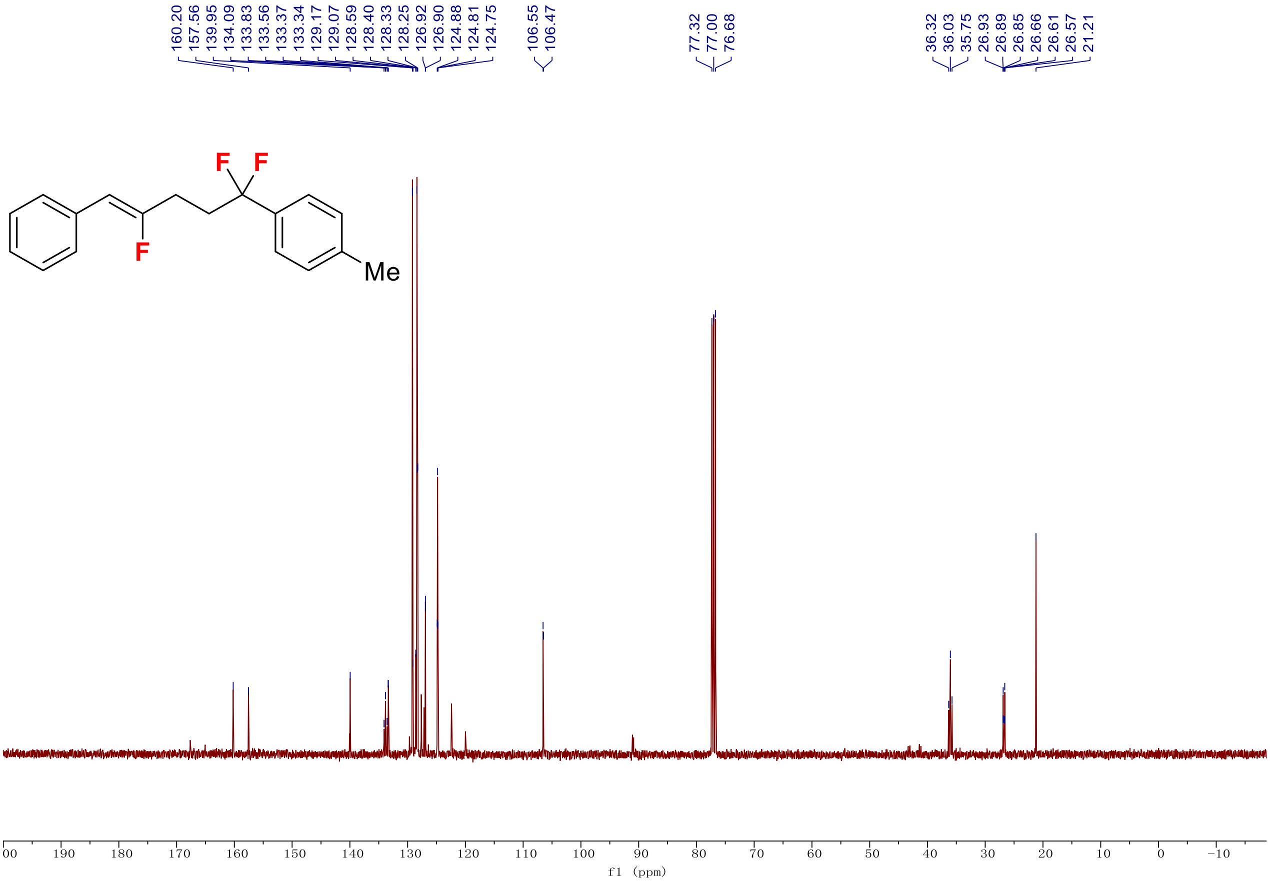

^{19}F NMR (376 MHz, CDCl3) spectrum of 3y

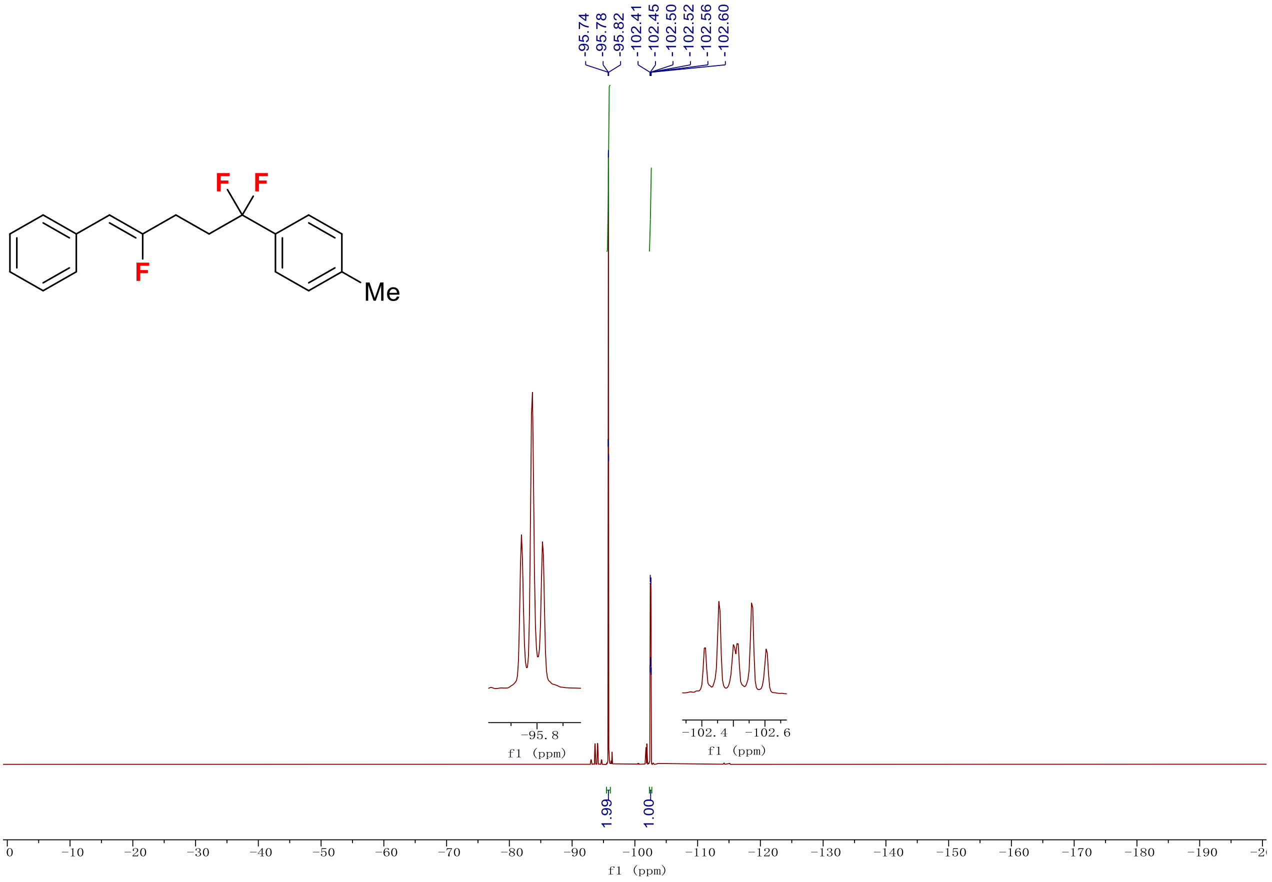

^{1}H NMR (400 MHz, CDCl3) spectrum of 3z

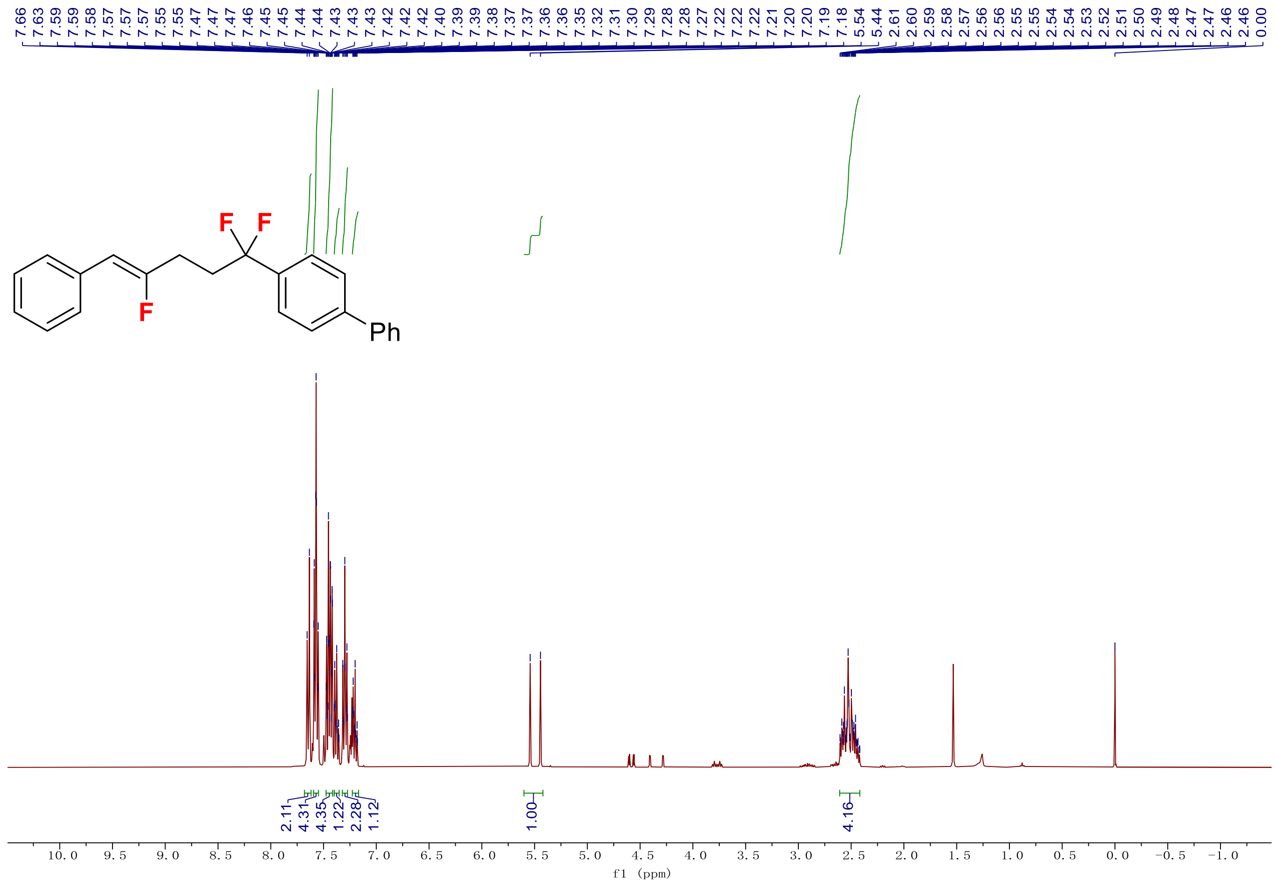

^{13}C NMR (101 MHz, CDCl3) spectrum of 3z

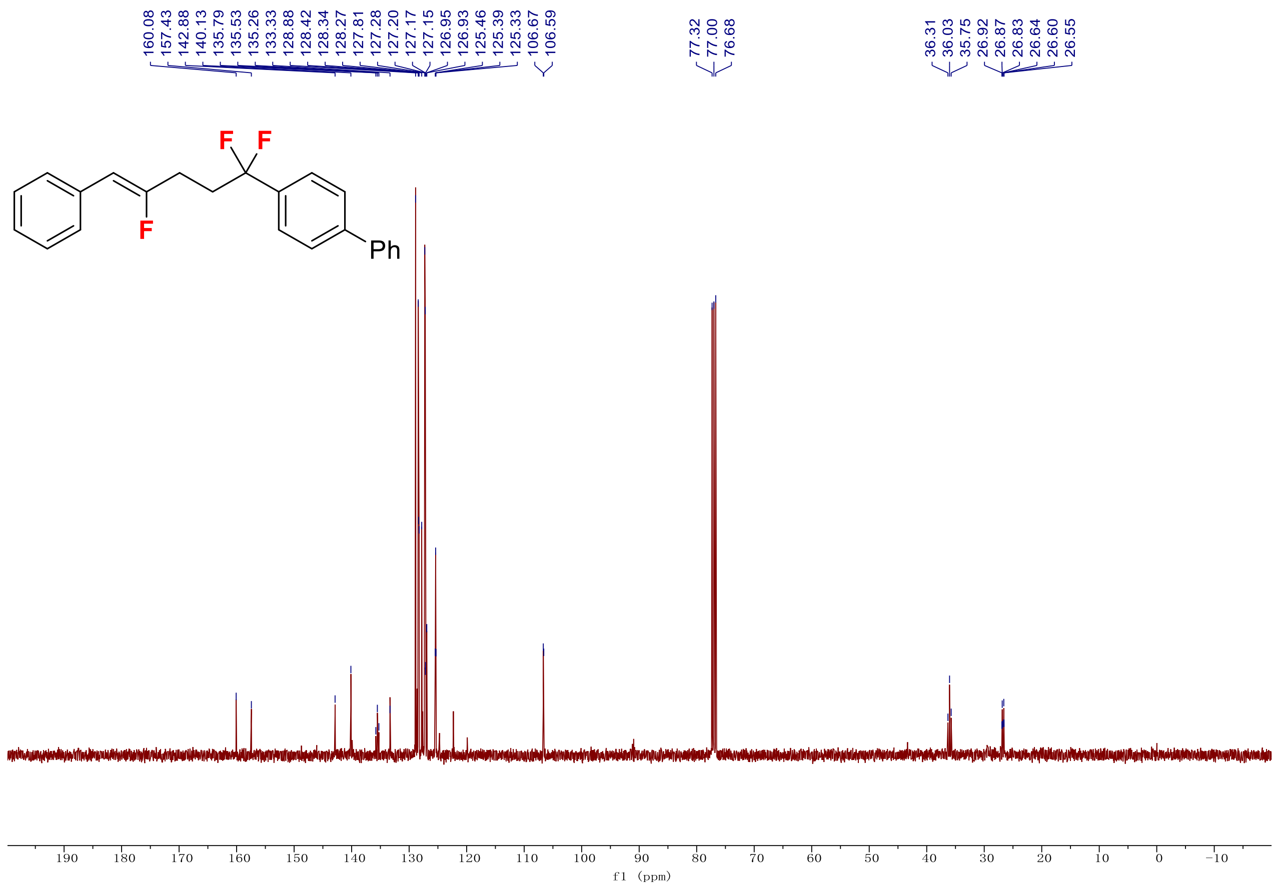

^{19}F NMR (376 MHz, CDCl3) spectrum of 3z

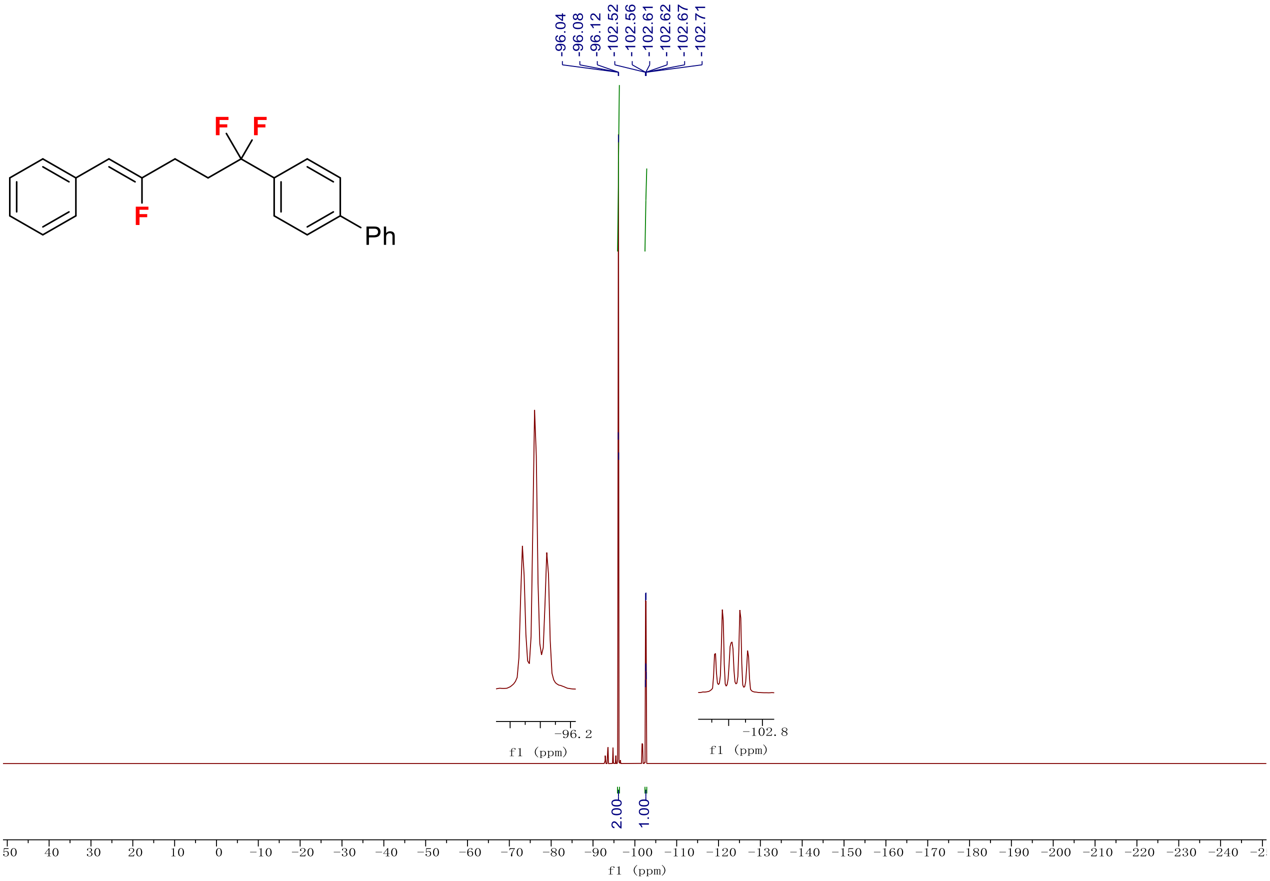

^{1}H NMR (400 MHz, $CDCl_3$) spectrum of 3aa

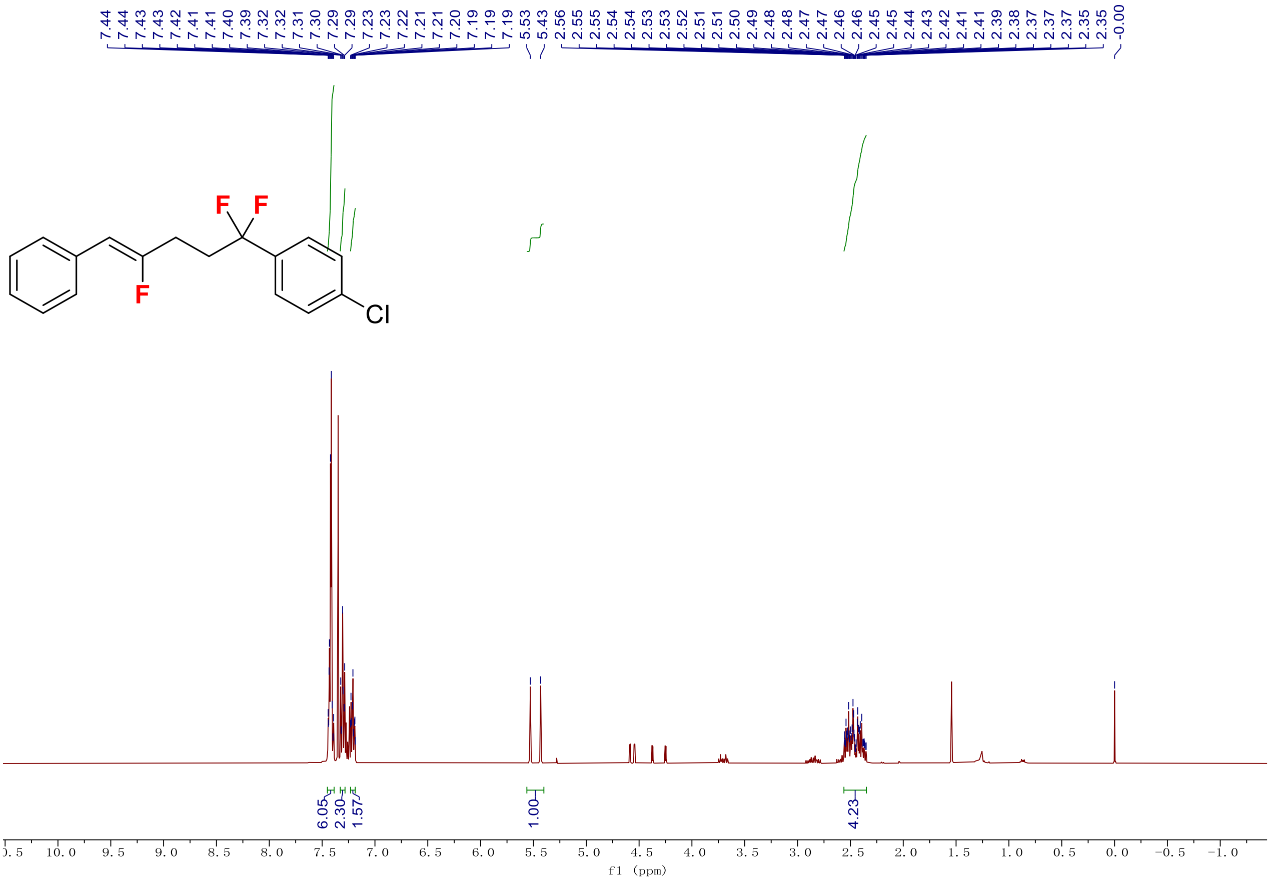

^{13}C NMR (101 MHz, $CDCl_3$) spectrum of 3aa

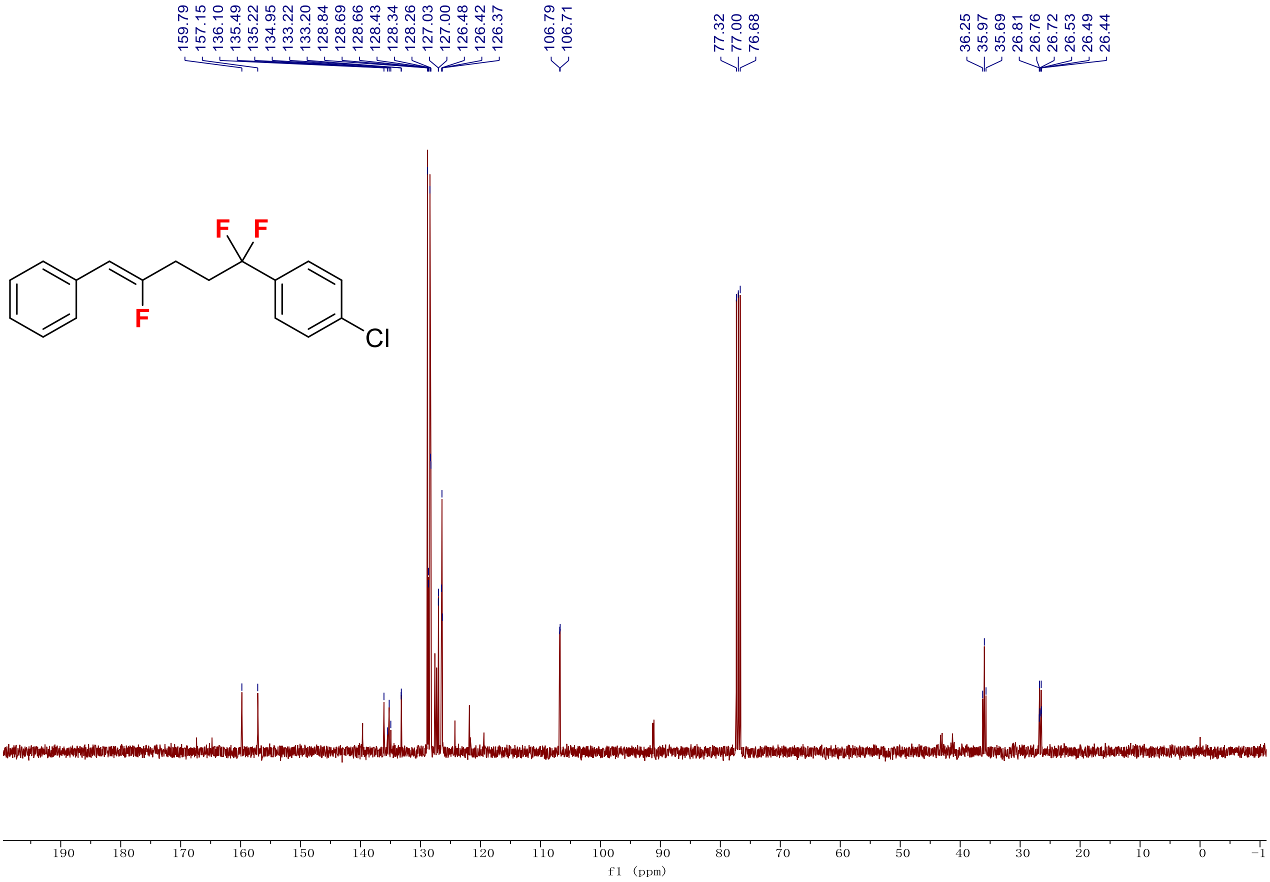

^{19}F NMR (376 MHz, $CDCl_3$) spectrum of 3aa

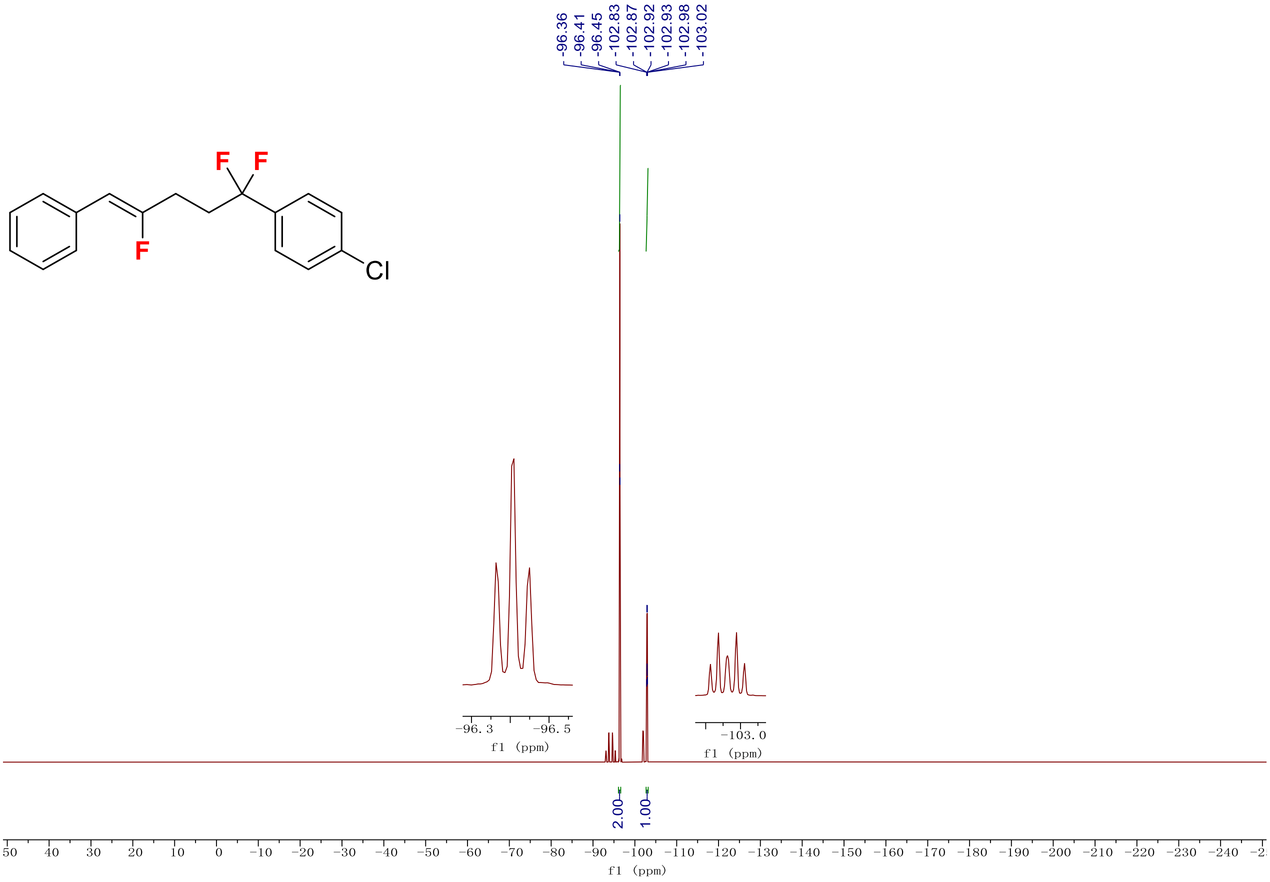

^{1}H NMR (400 MHz, CDCl$_3$) spectrum of 3ab

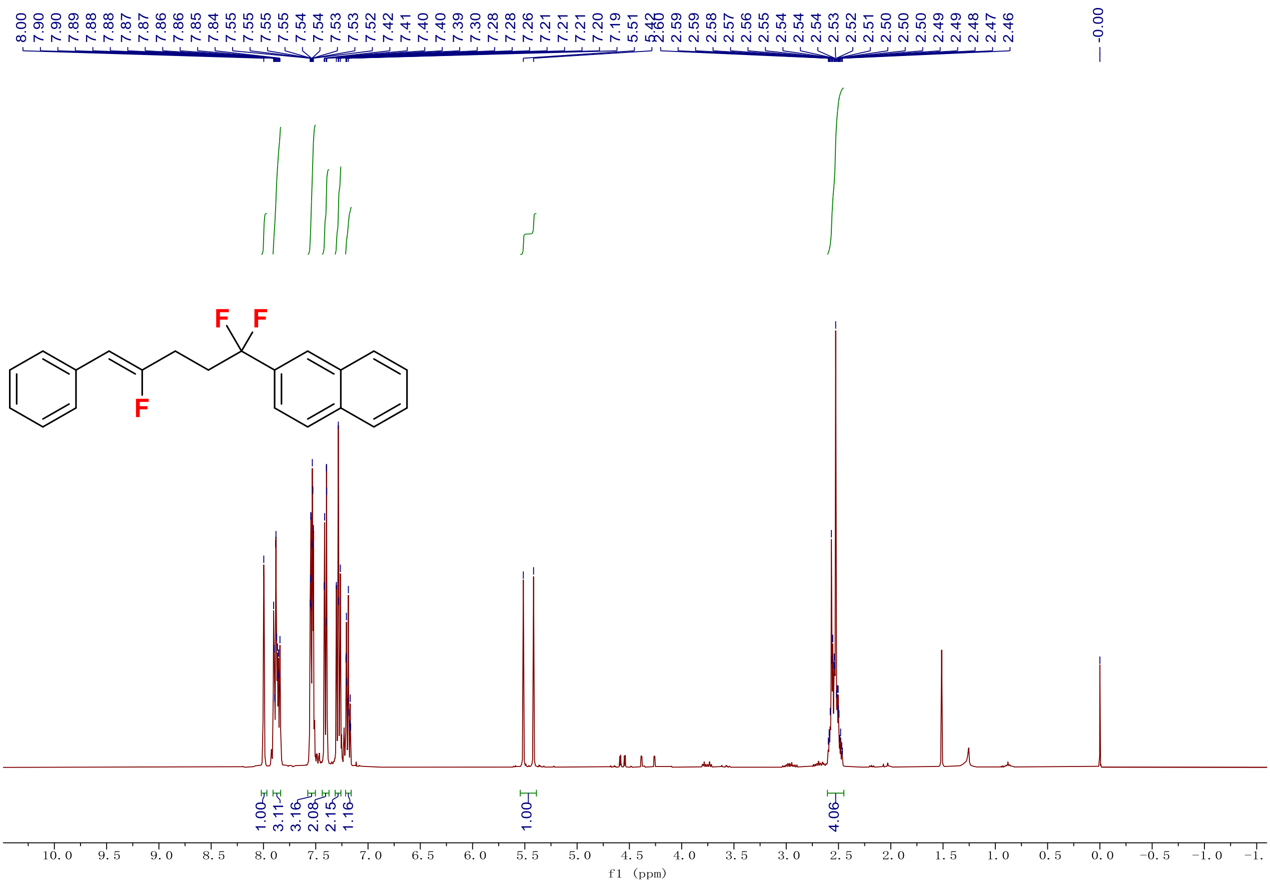

^{13}C NMR (101 MHz, CDCl$_3$) spectrum of 3ab

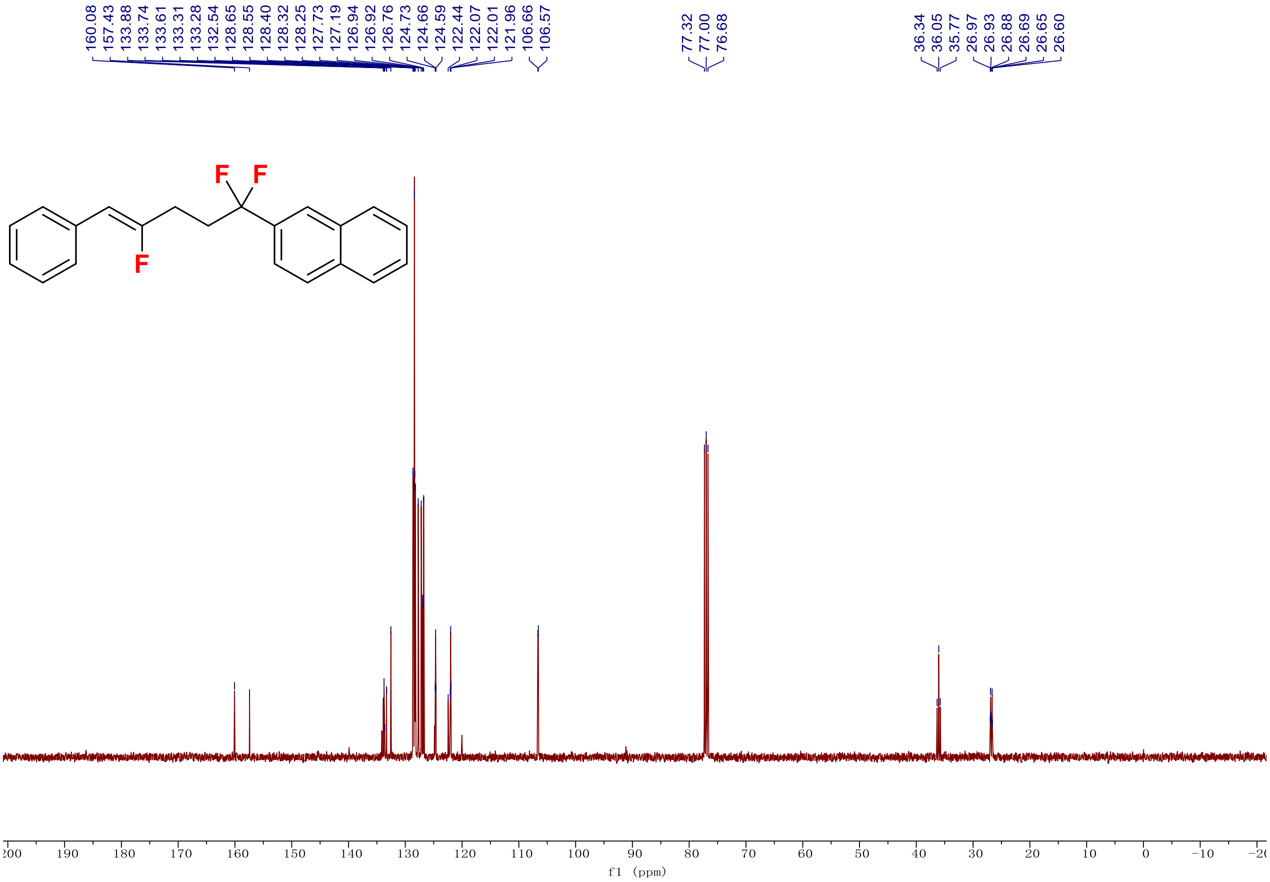

^{19}F NMR (376 MHz, $CDCl_3$) spectrum of 3ab

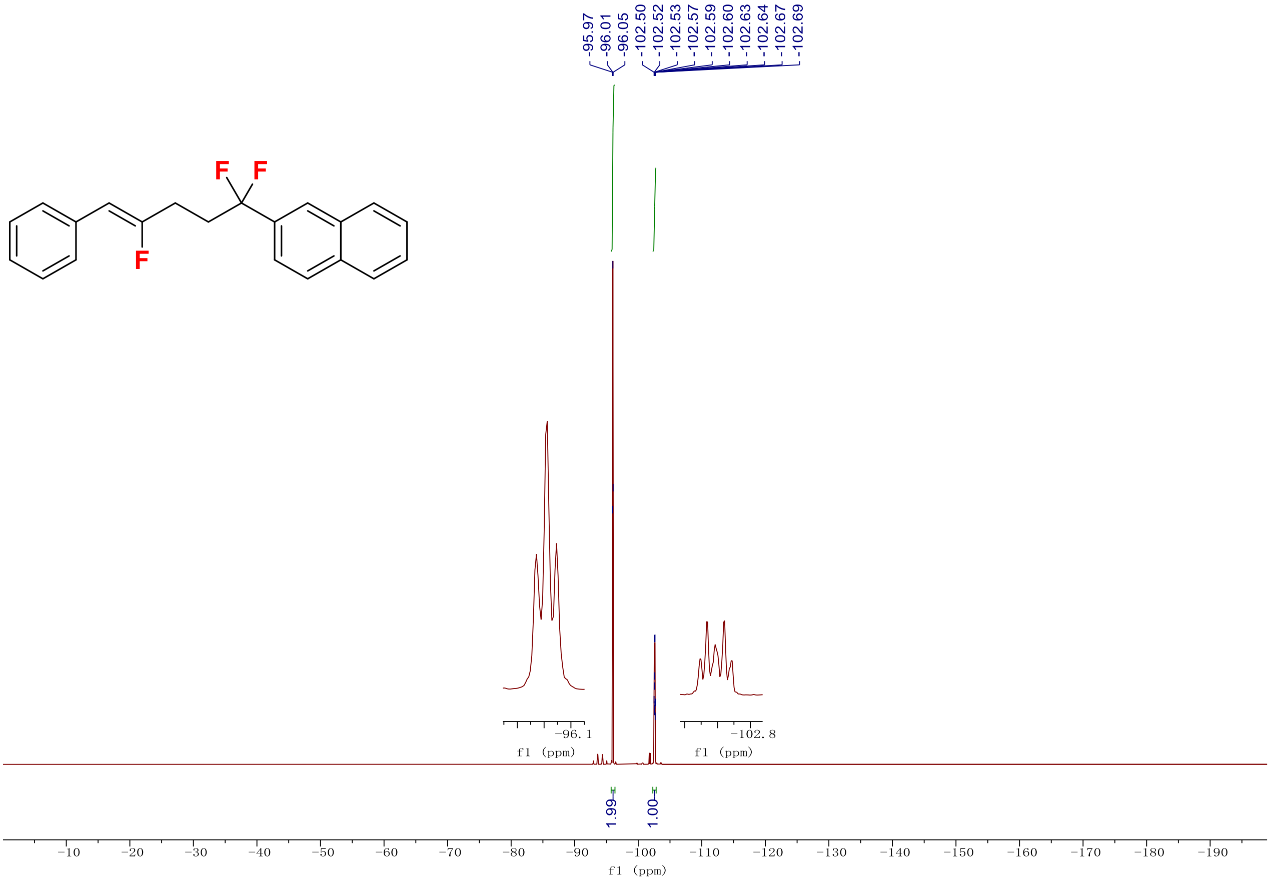

^{1}H NMR (400 MHz, CDCl3) spectrum of 3ac

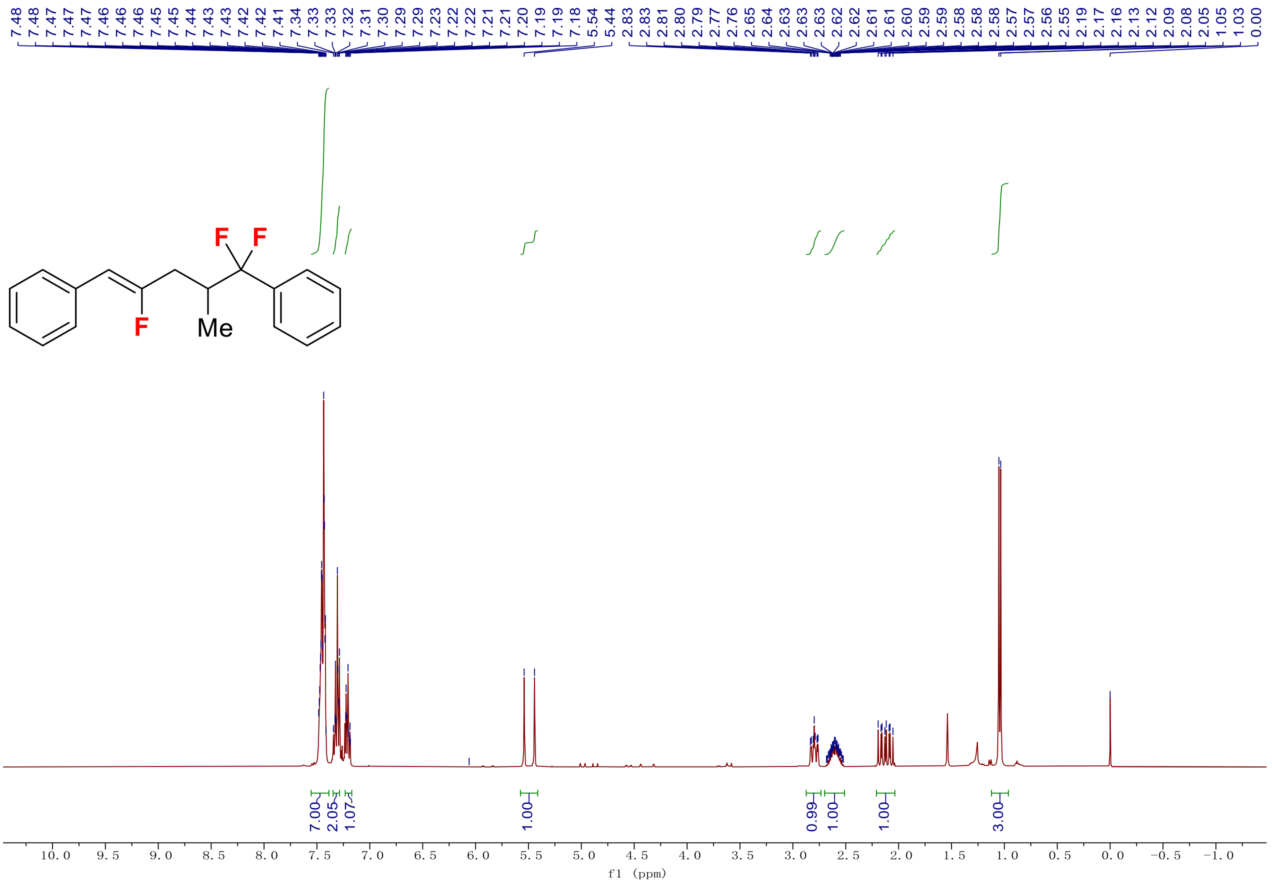

^{13}C NMR (101 MHz, CDCl3) spectrum of 3ac

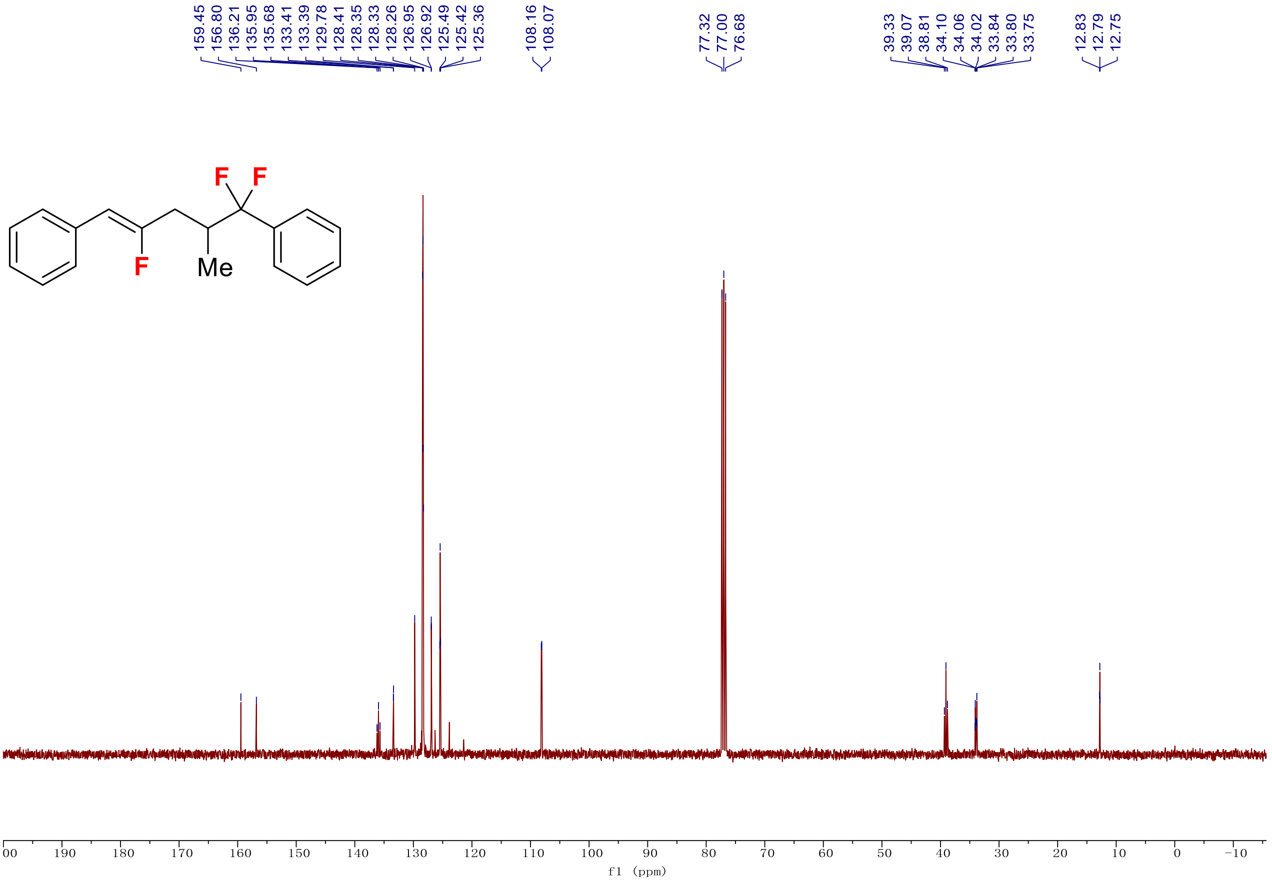

^{19}F NMR (376 MHz, $CDCl_3$) spectrum of 3ac

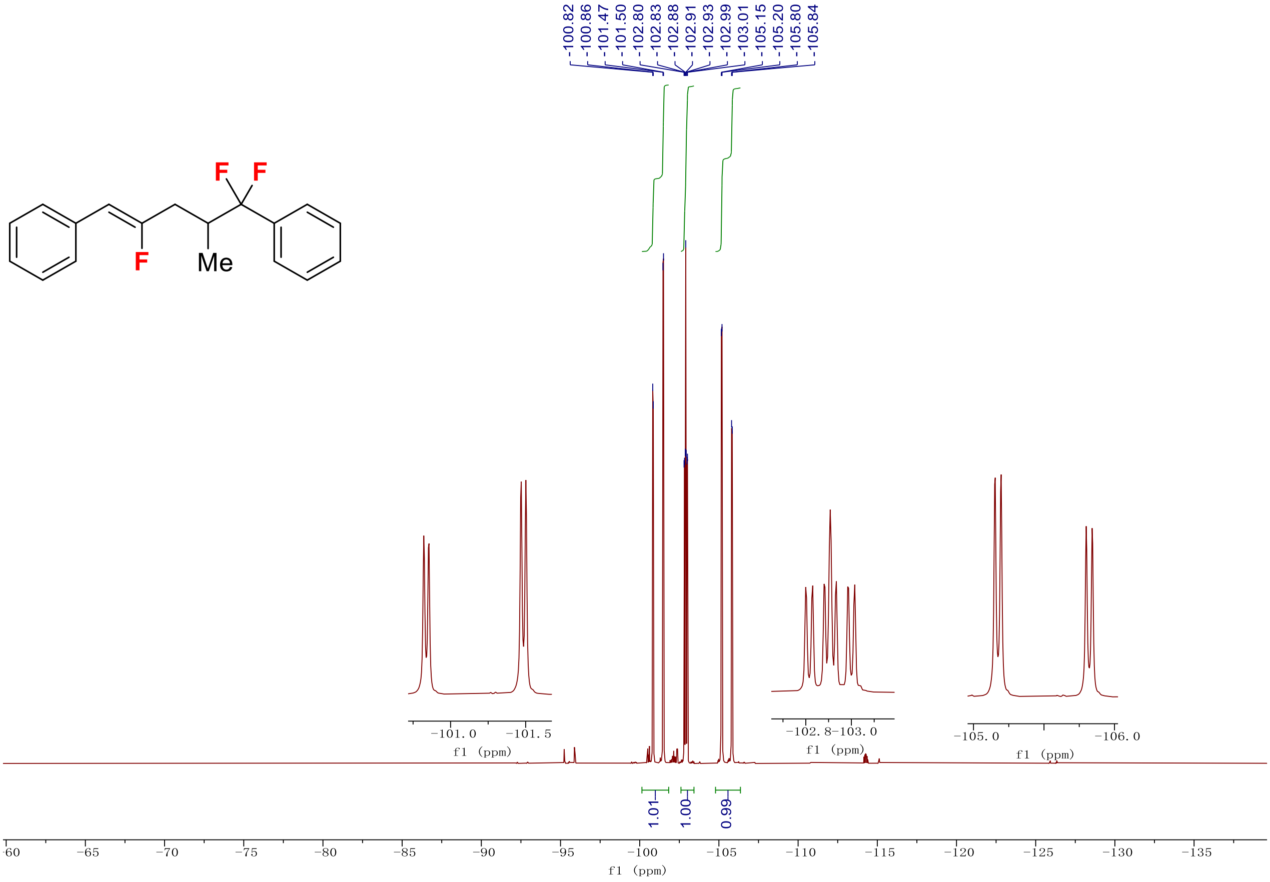

^{1}H NMR (400 MHz, $CDCl_3$) spectrum of 4b

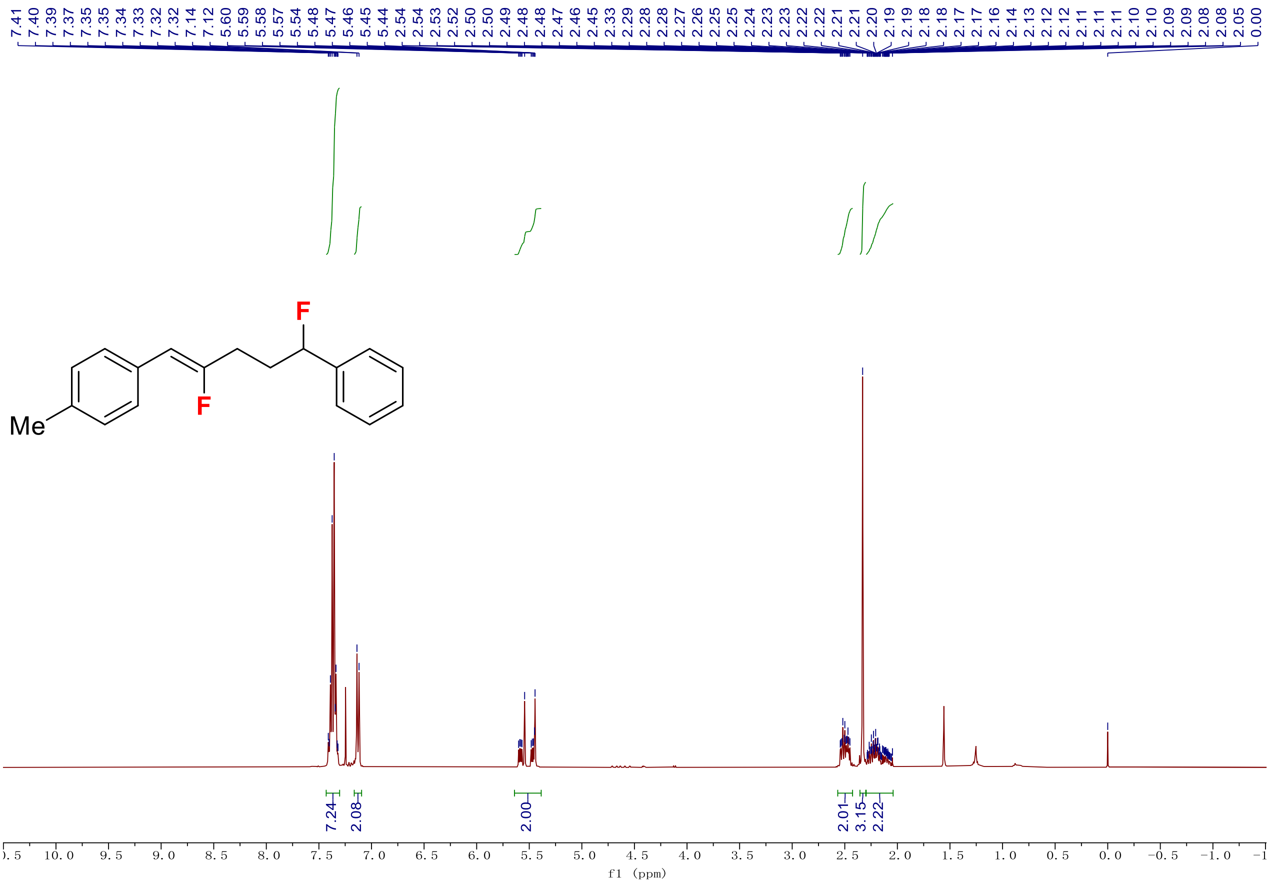

^{13}C NMR (101 MHz, $CDCl_3$) spectrum of 4b

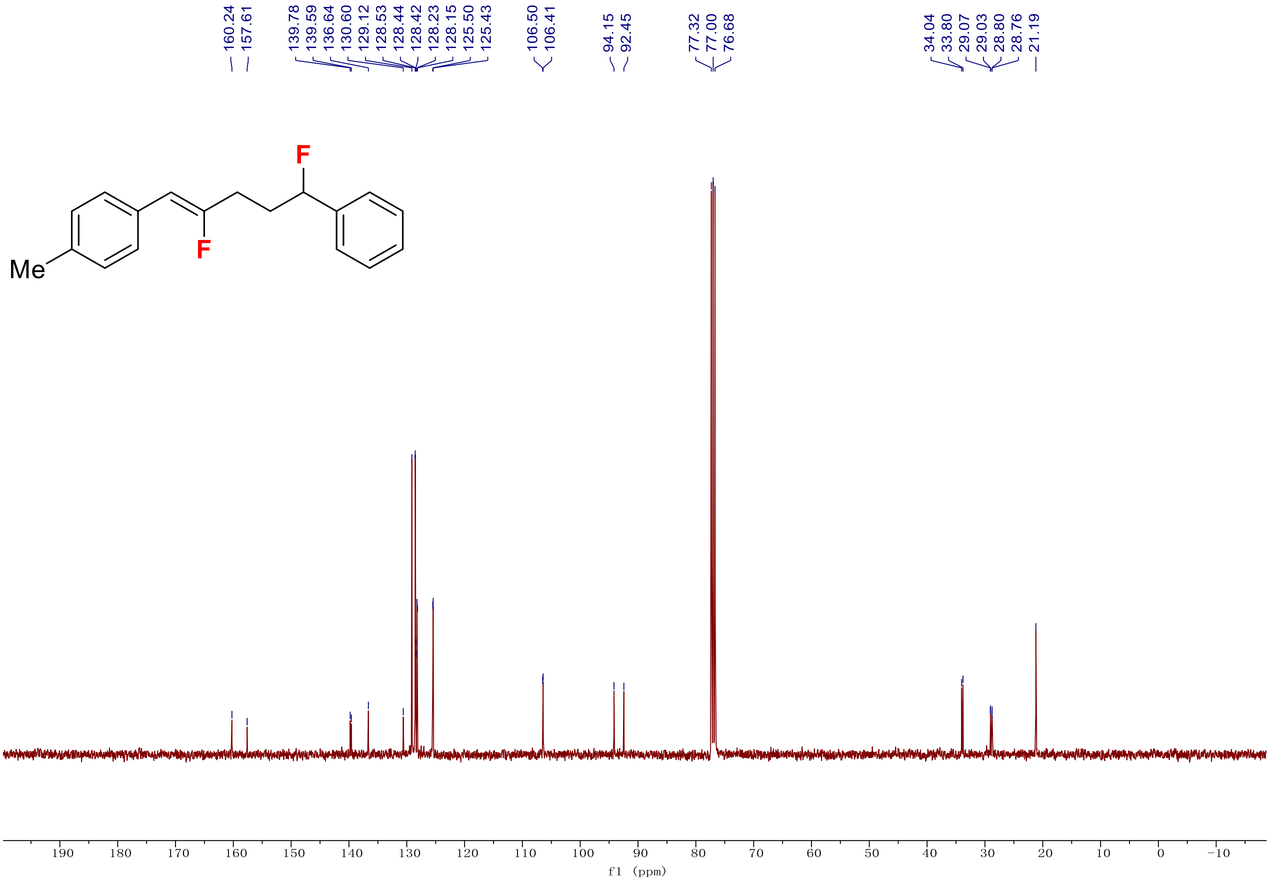

^{19}F NMR (376 MHz, CDCl3) spectrum of 4b

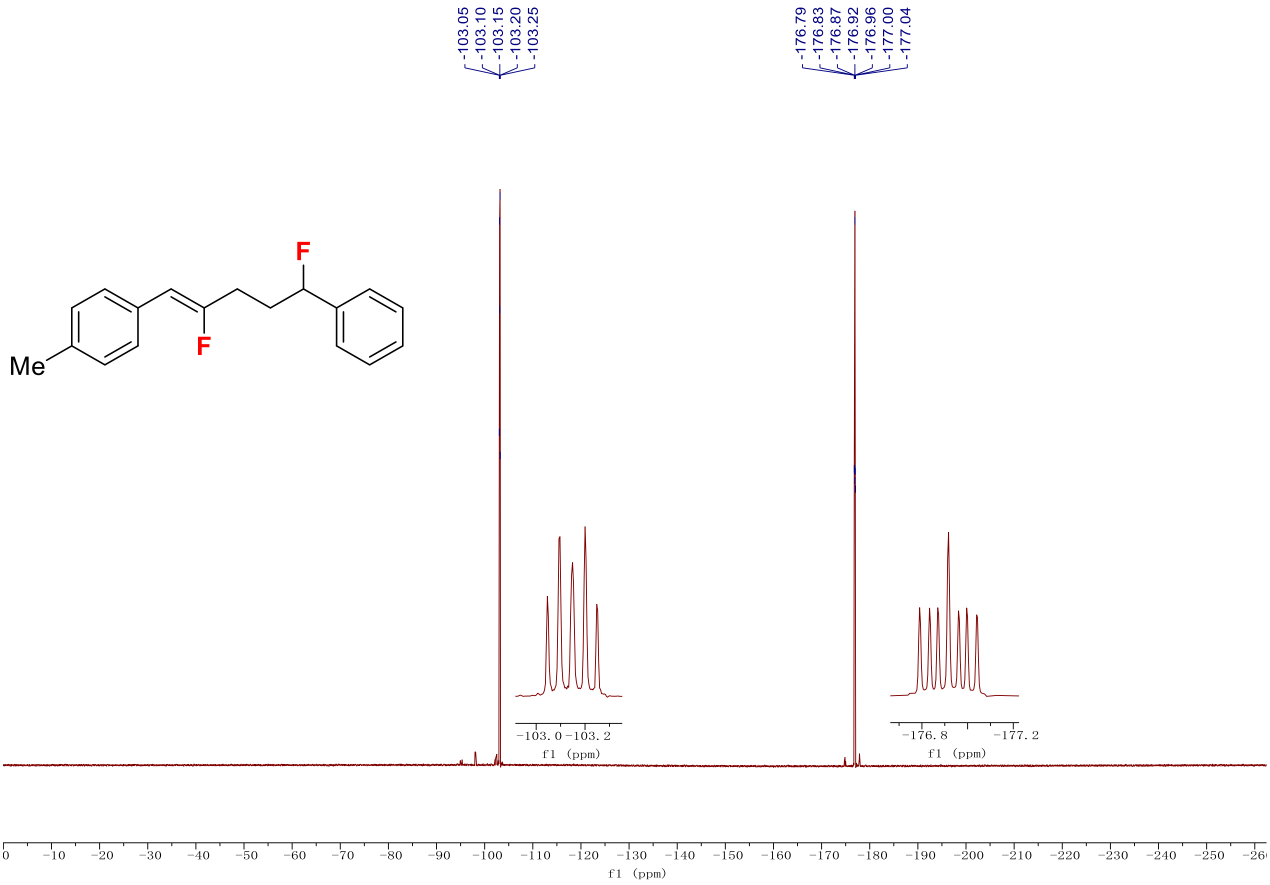

^{1}H NMR (400 MHz, CDCl3) spectrum of 4c

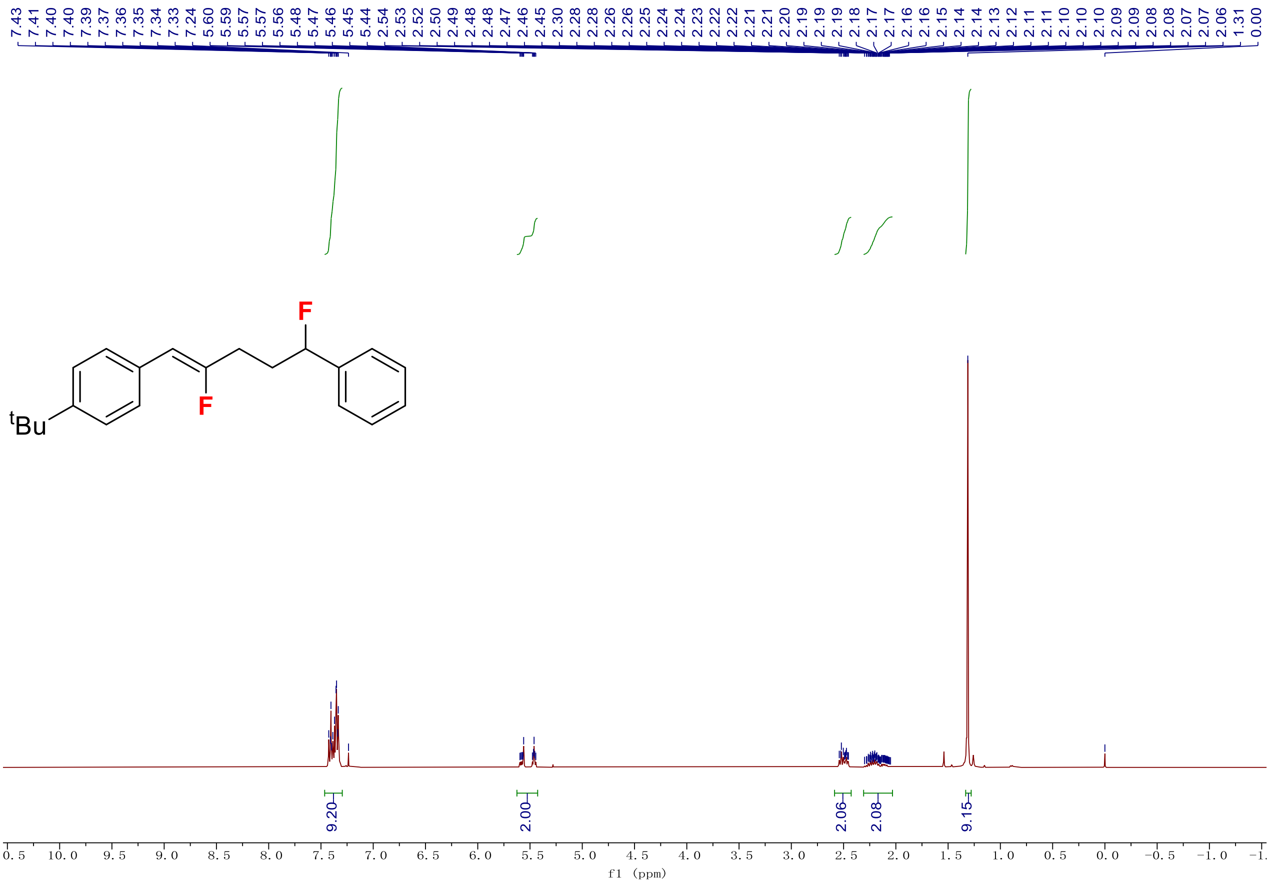

^{13}C NMR (101 MHz, CDCl3) spectrum of 4c

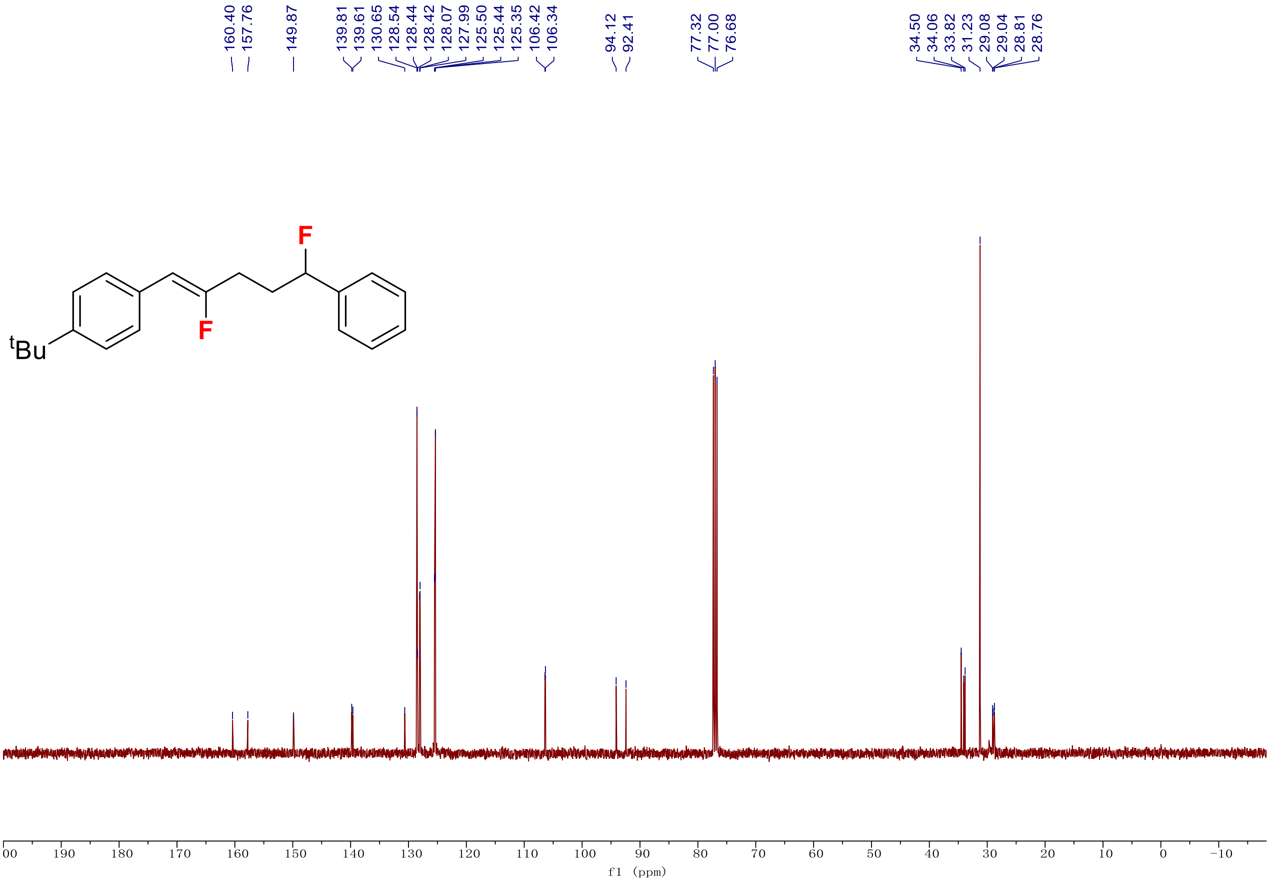

^{19}F NMR (376 MHz, $CDCl_3$) spectrum of 4c

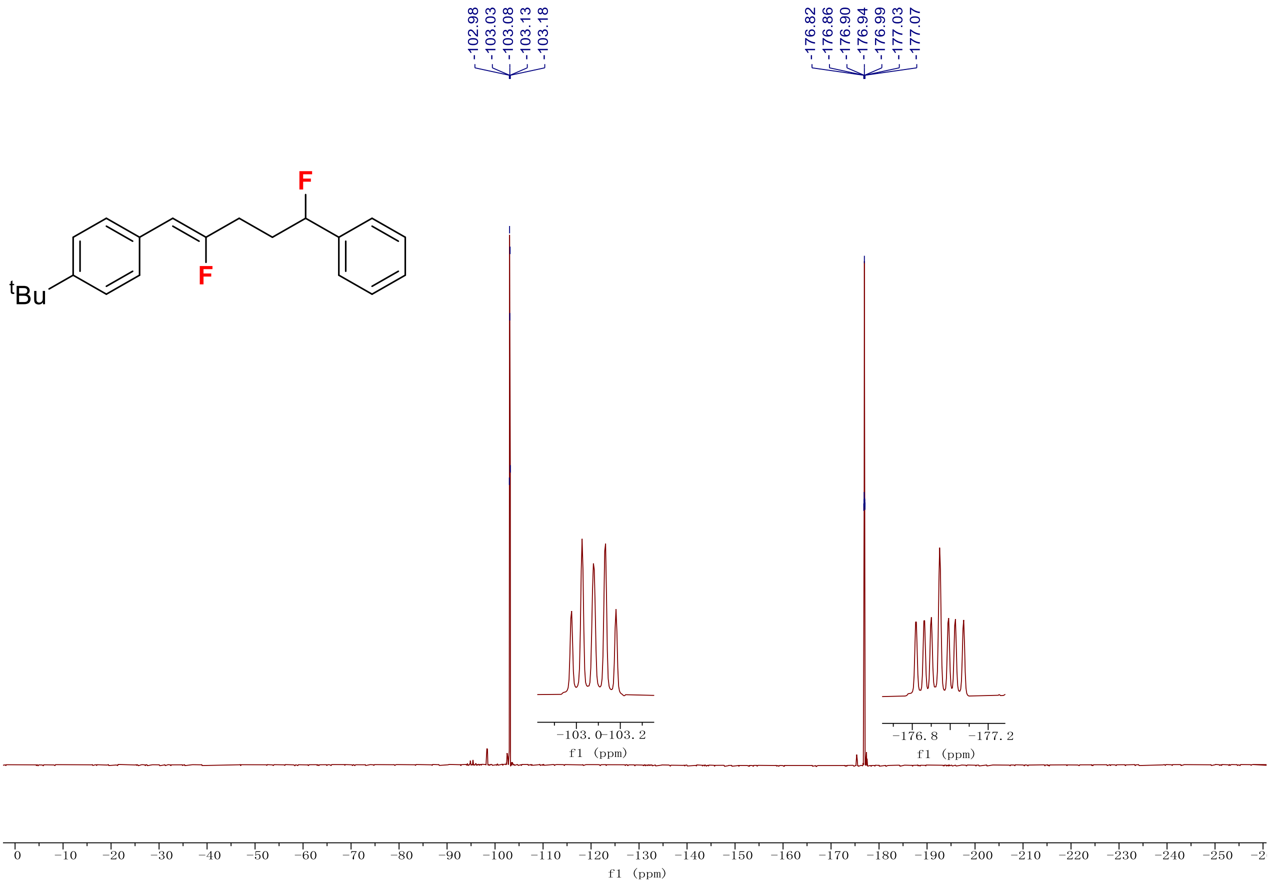

^{1}H NMR (400 MHz, CDCl$_3$) spectrum of 4d

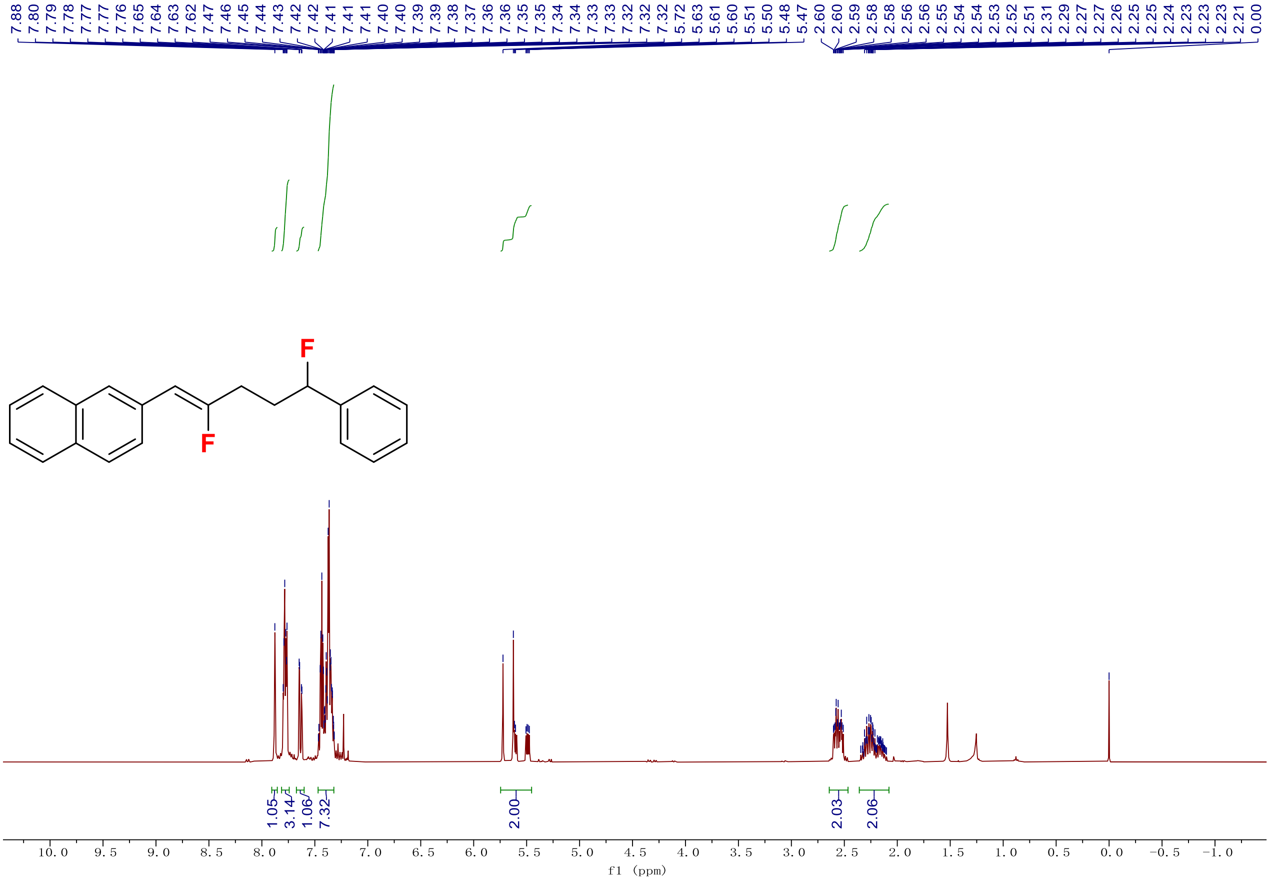

^{13}C NMR (101 MHz, CDCl$_3$) spectrum of 4d

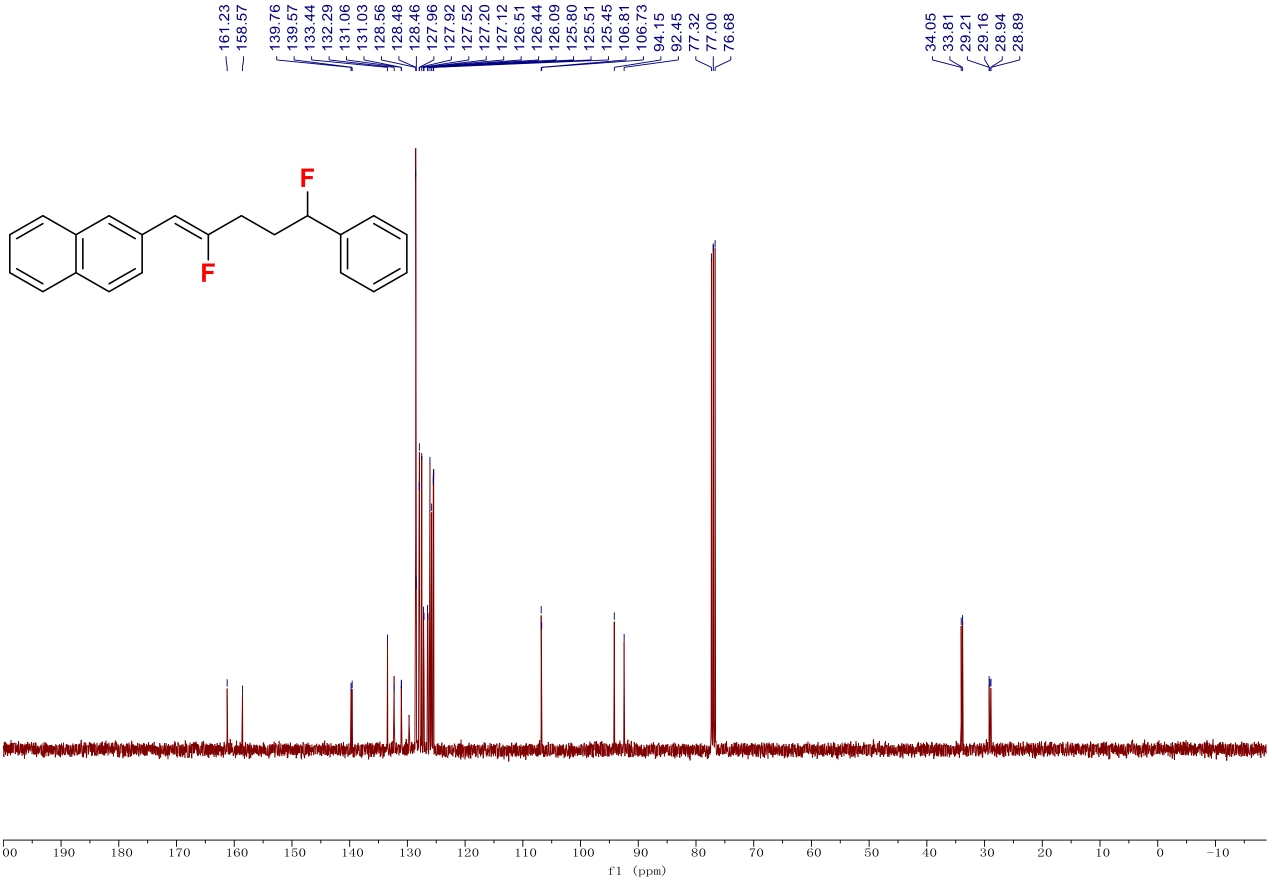

^{19}F NMR (376 MHz, CDCl₃) spectrum of 4d

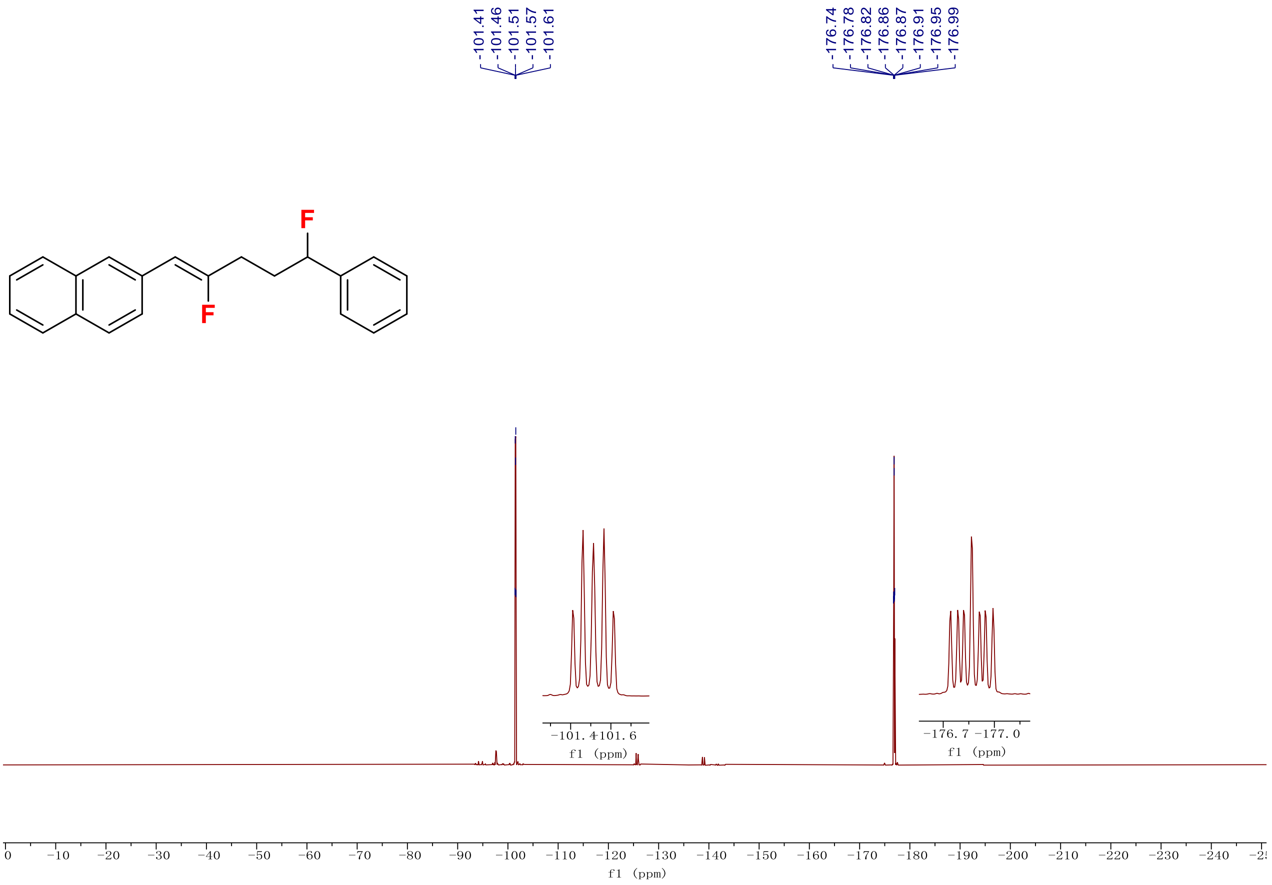

^{1}H NMR (400 MHz, $CDCl_3$) spectrum of 4e

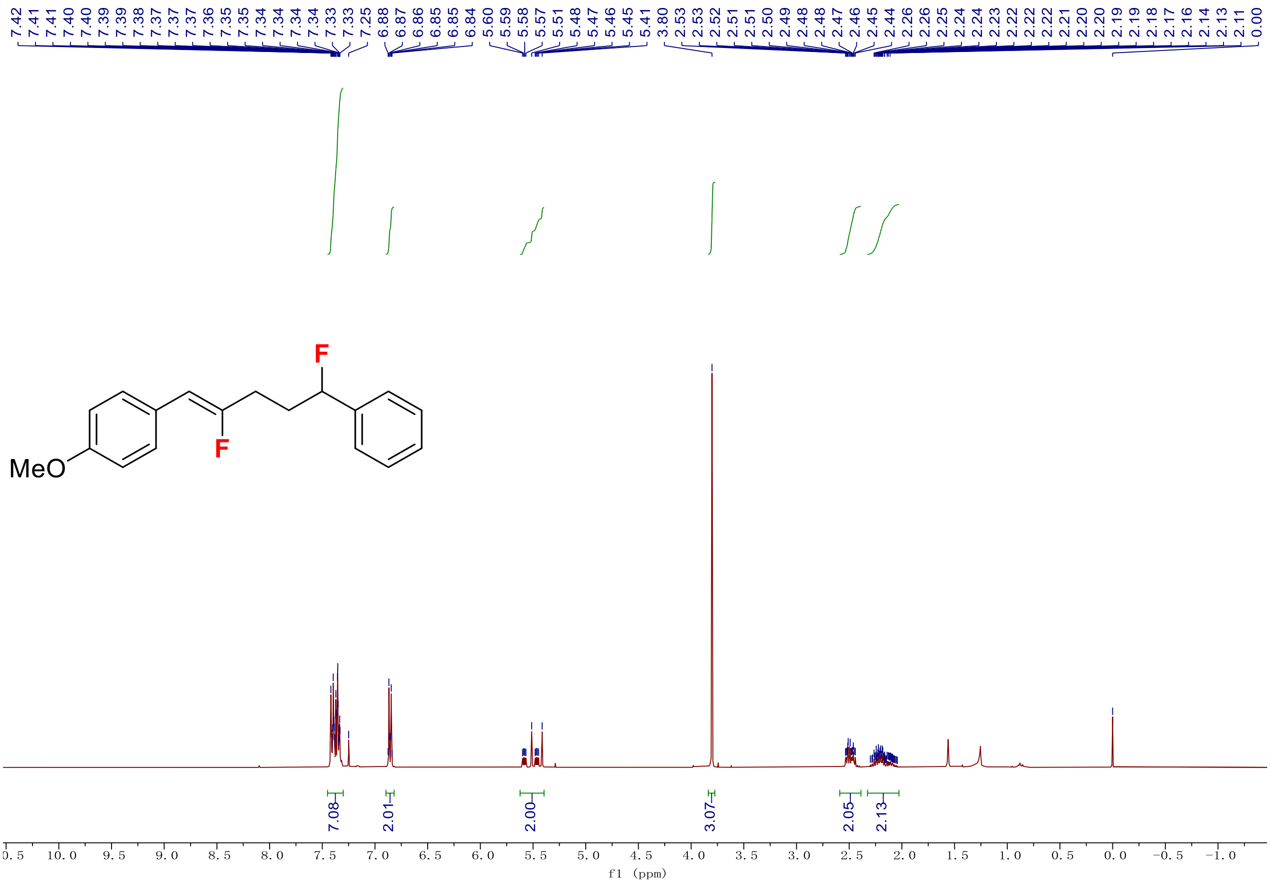

^{13}C NMR (101 MHz, $CDCl_3$) spectrum of 4e

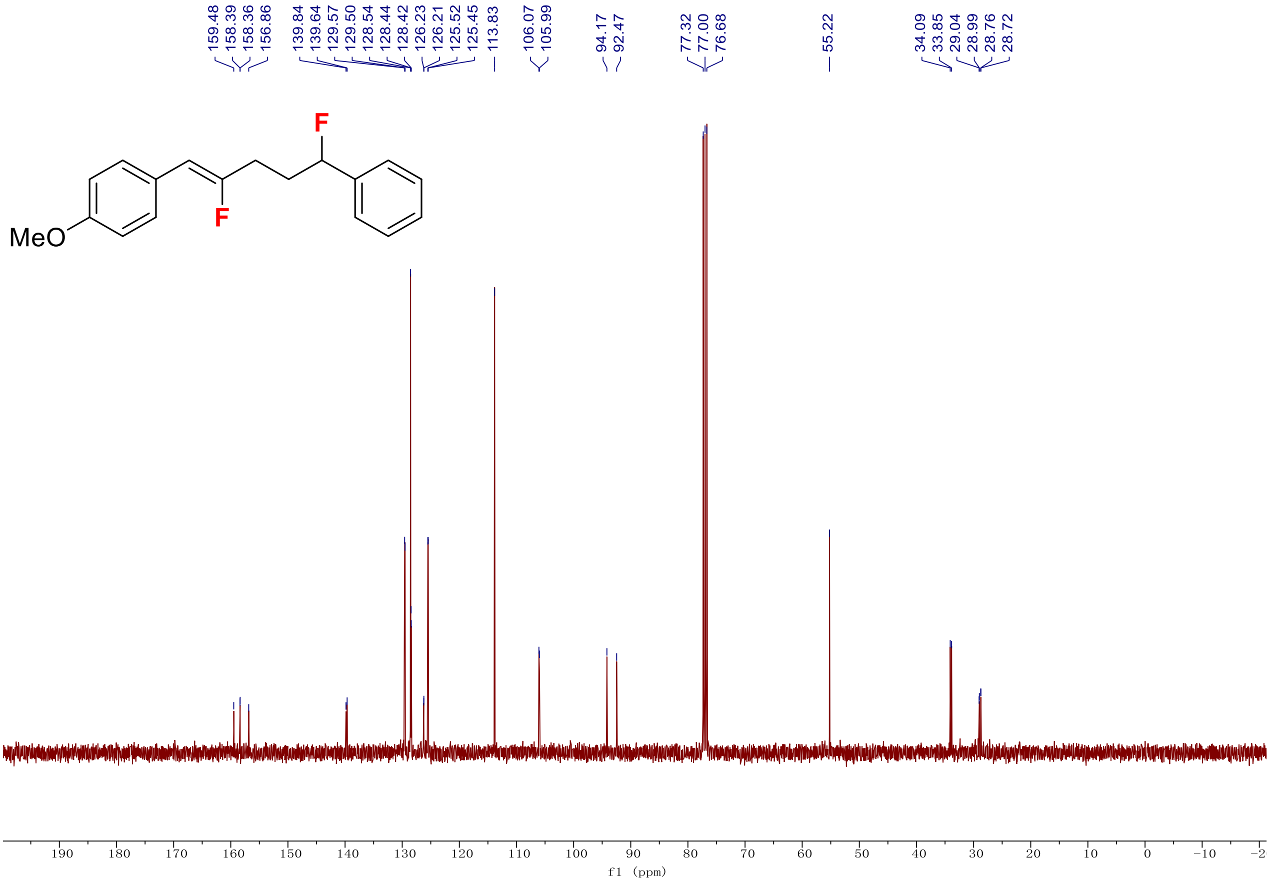

^{19}F NMR (376 MHz, $CDCl_3$) spectrum of 4e

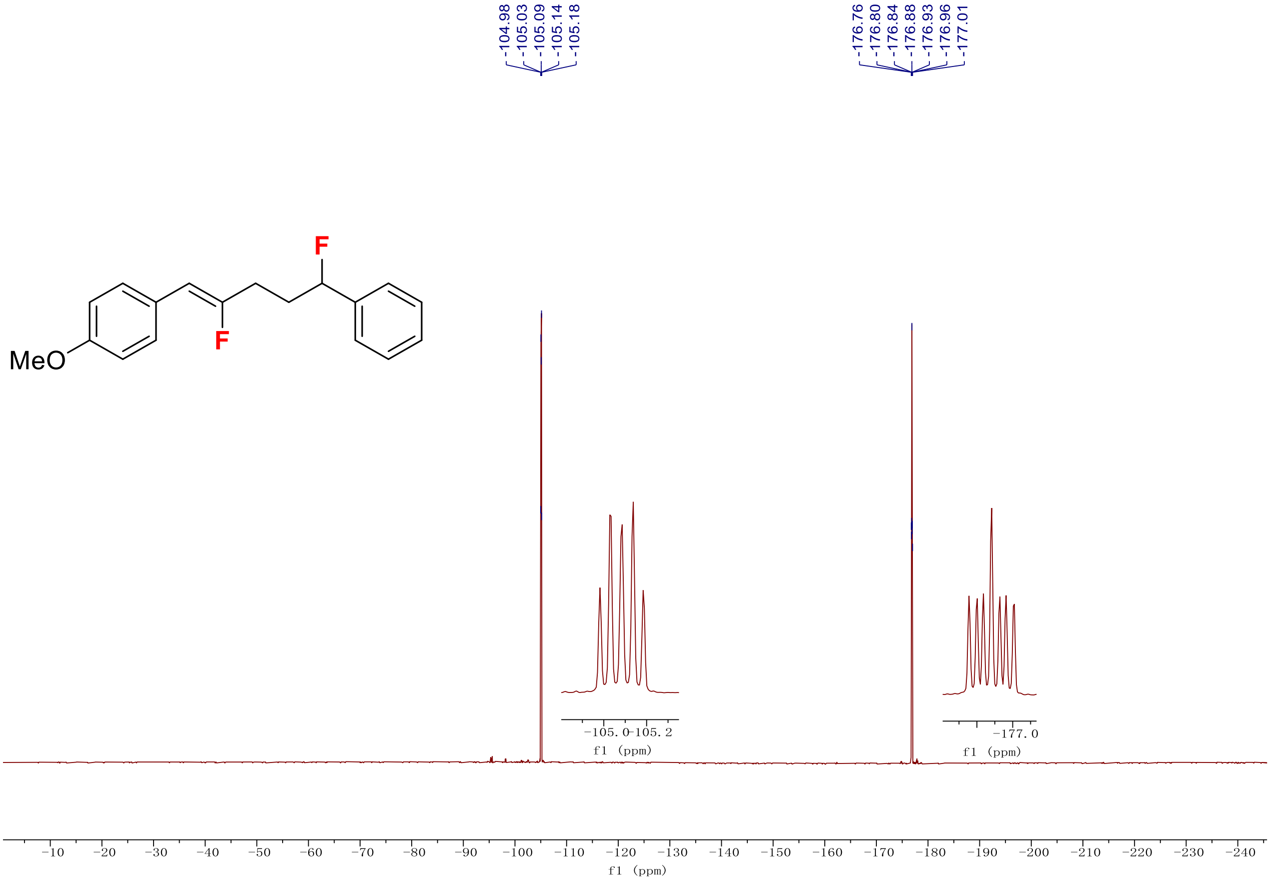

^1H NMR (400 MHz, $CDCl_3$) spectrum of 4f

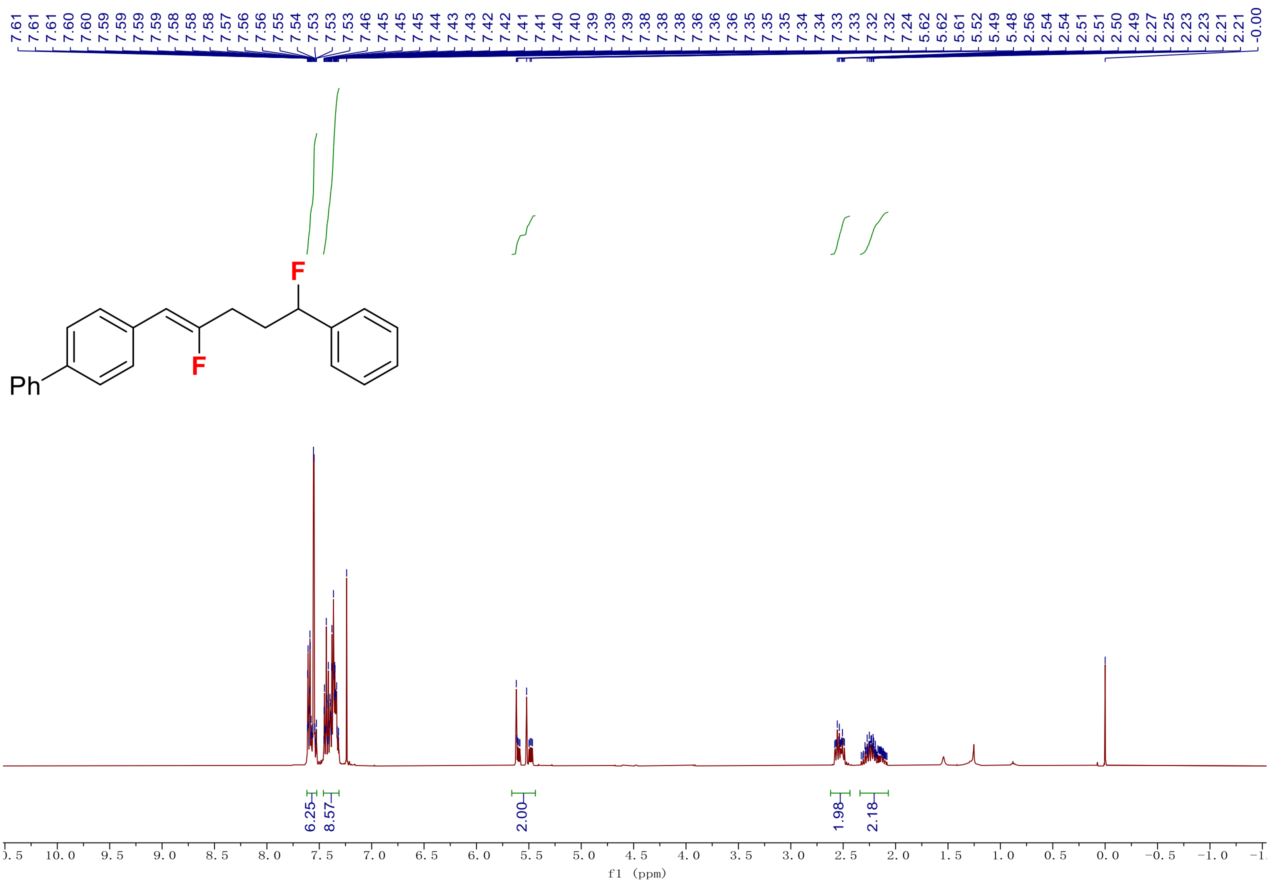

^{13}C NMR (101 MHz, $CDCl_3$) spectrum of 4f

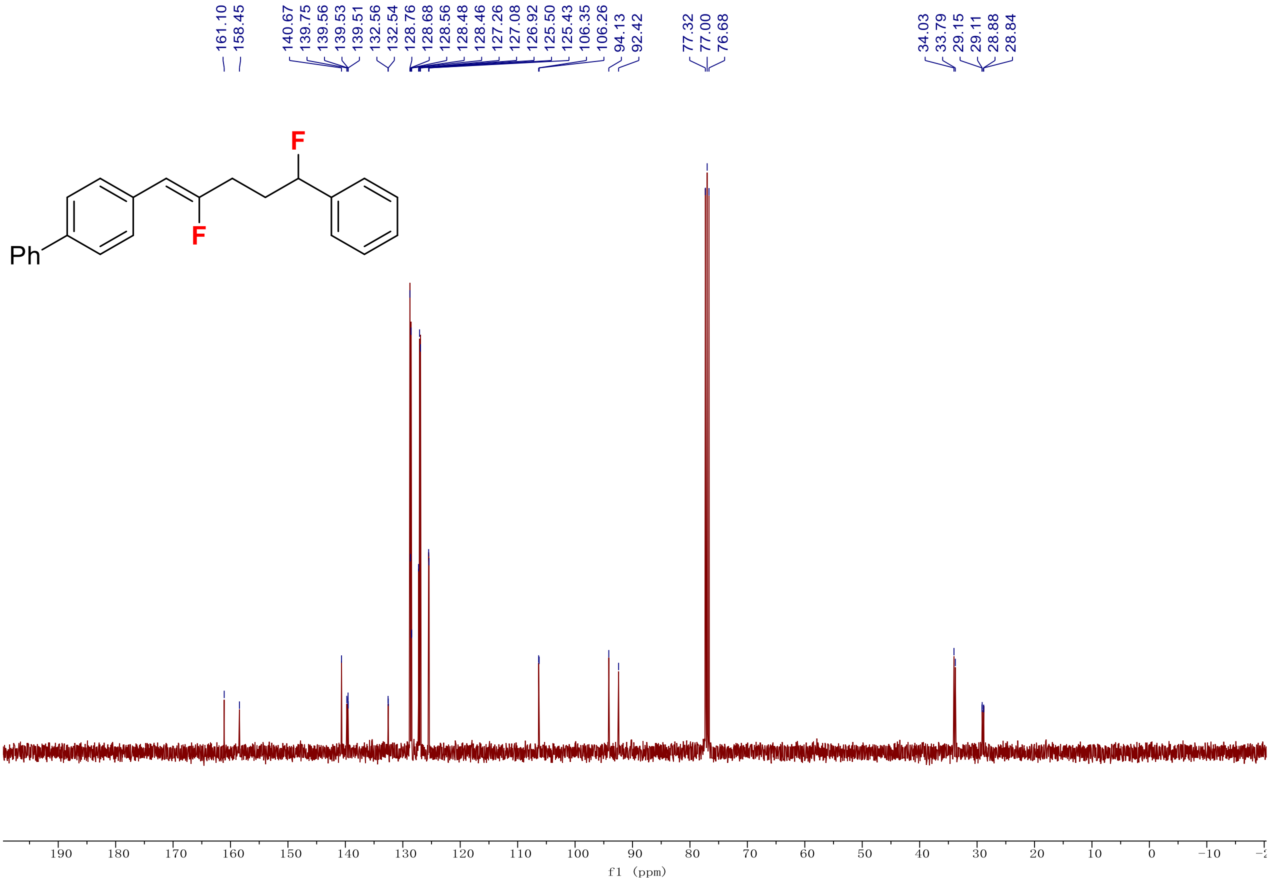

^{19}F NMR (376 MHz, CDCl₃) spectrum of 4f

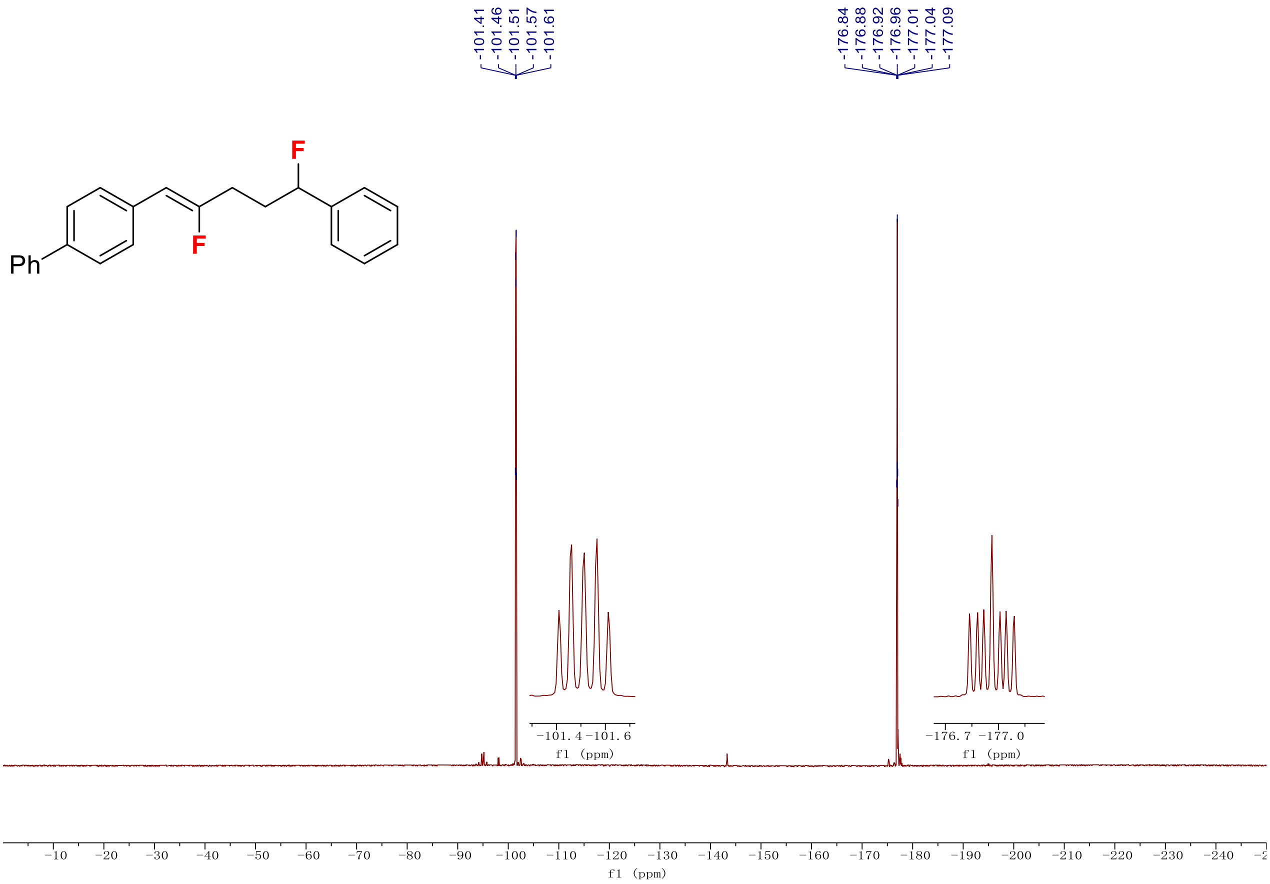

^{1}H NMR (400 MHz, CDCl3) spectrum of 4g

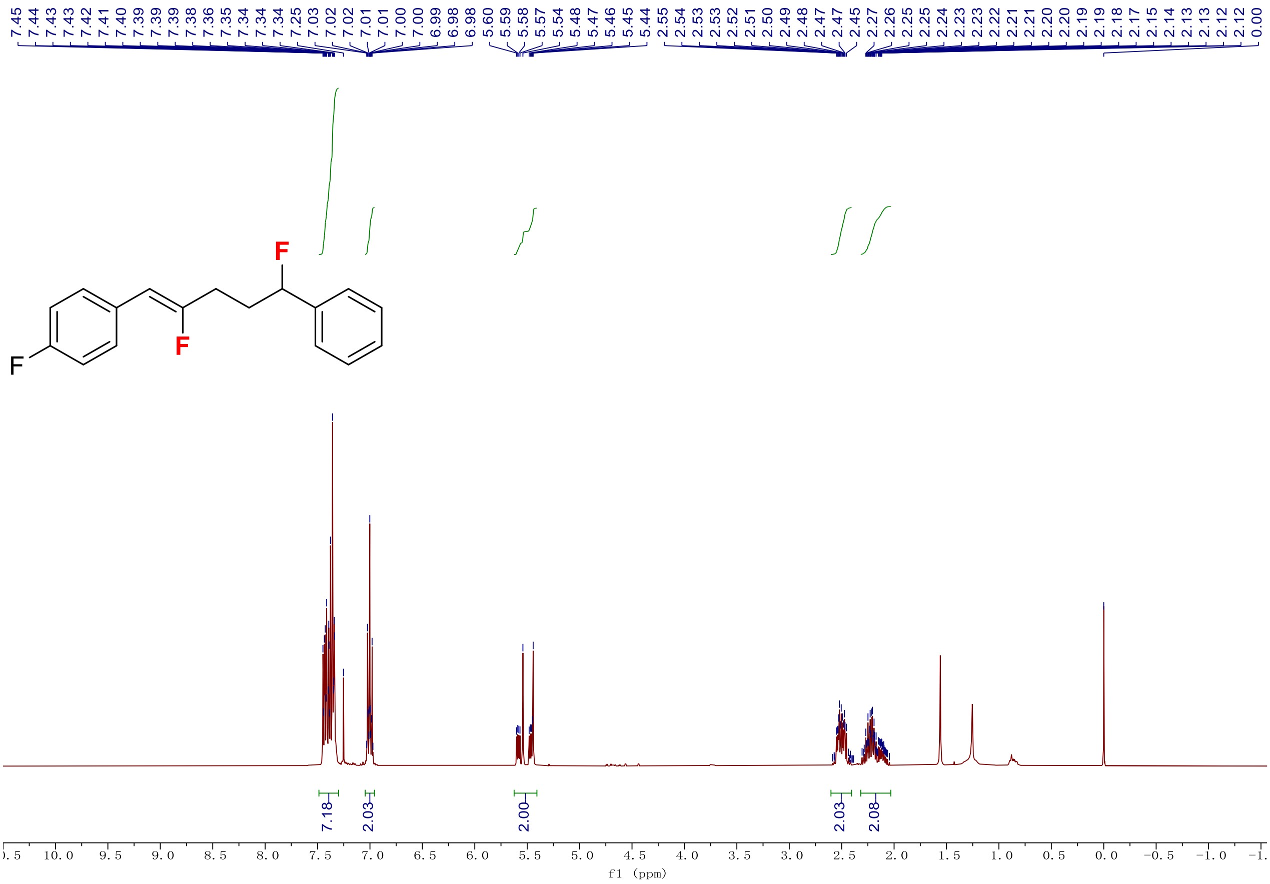

^{13}C NMR (101 MHz, CDCl3) spectrum of 4g

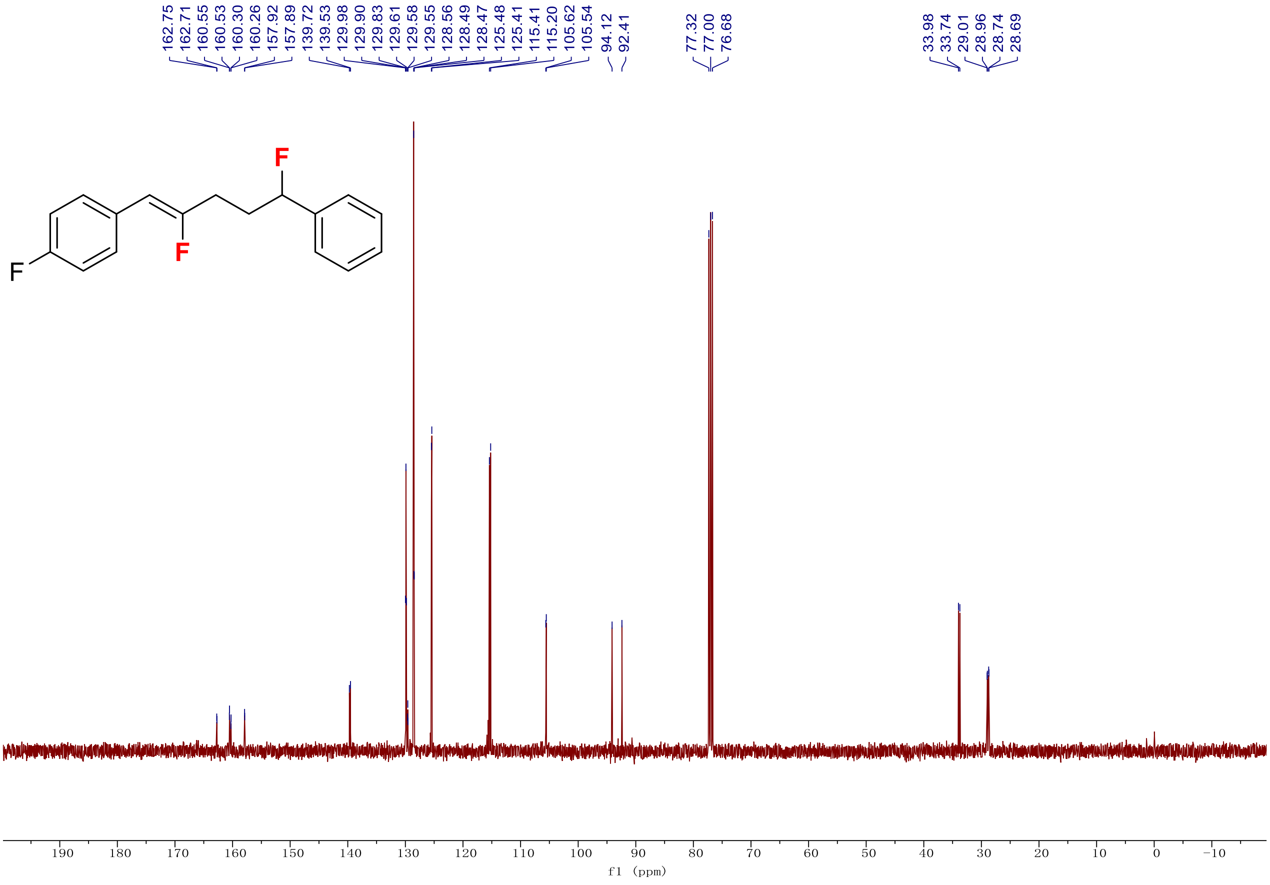

^{19}F NMR (376 MHz, CDCl3) spectrum of 4g

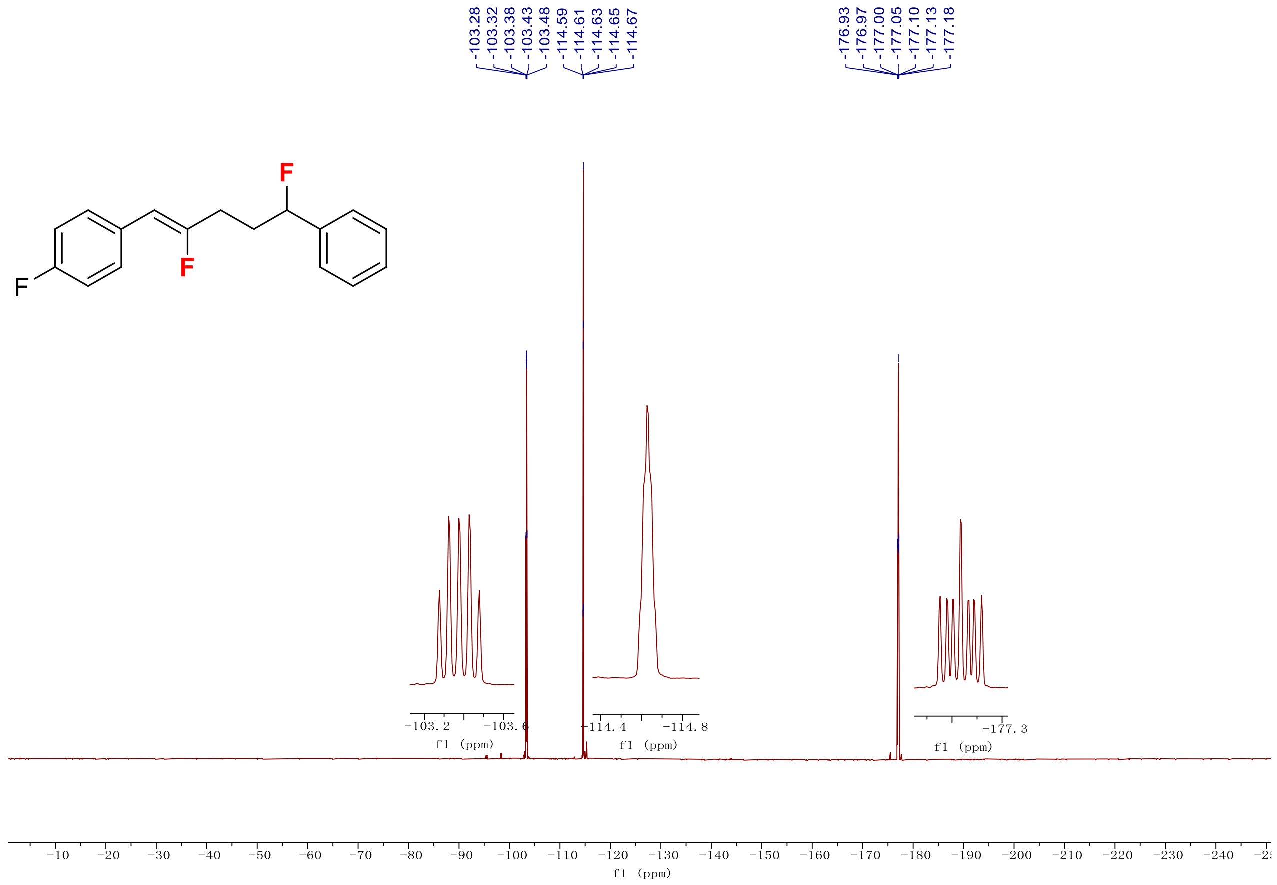

^{1}H NMR (400 MHz, CDCl$_3$) spectrum of 4h

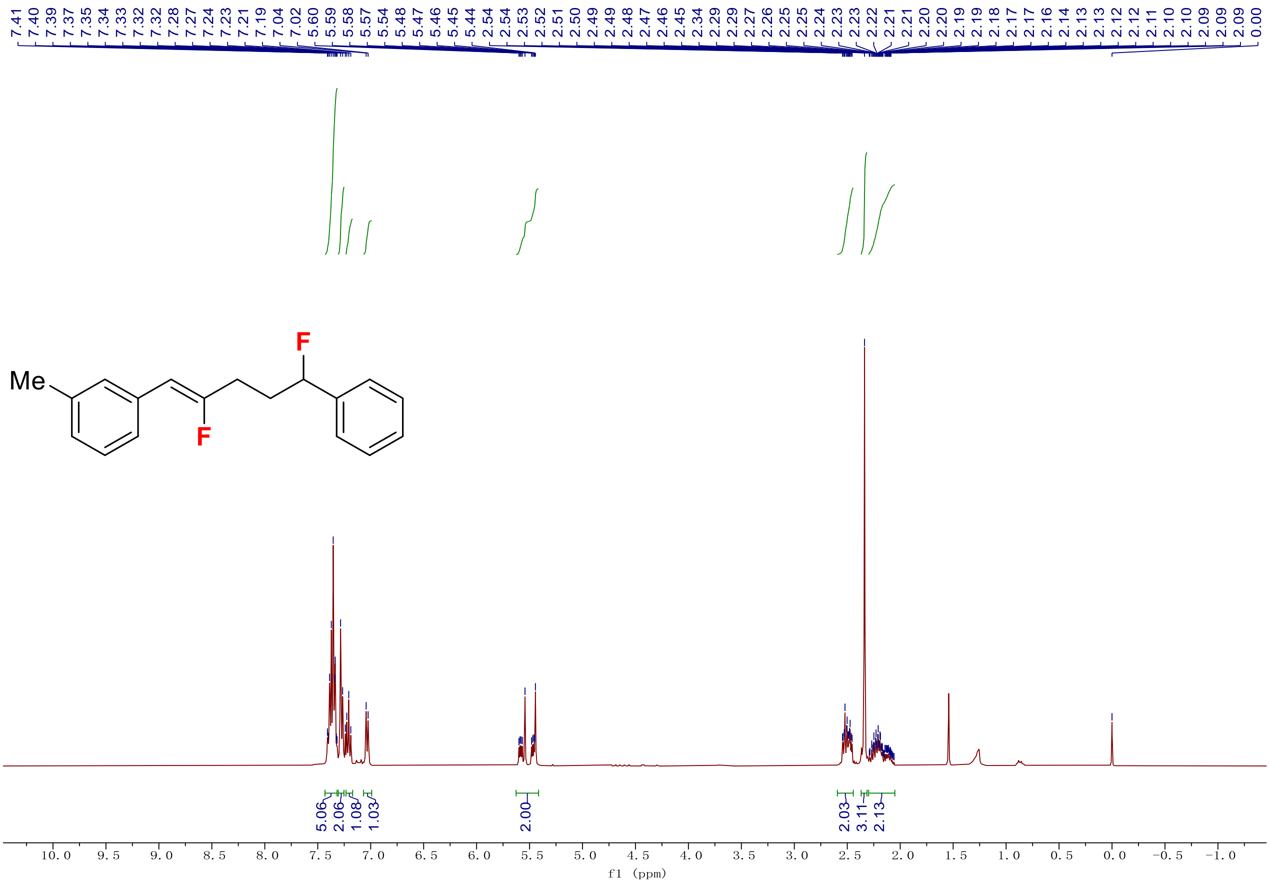

^{13}C NMR (101 MHz, CDCl$_3$) spectrum of 4h

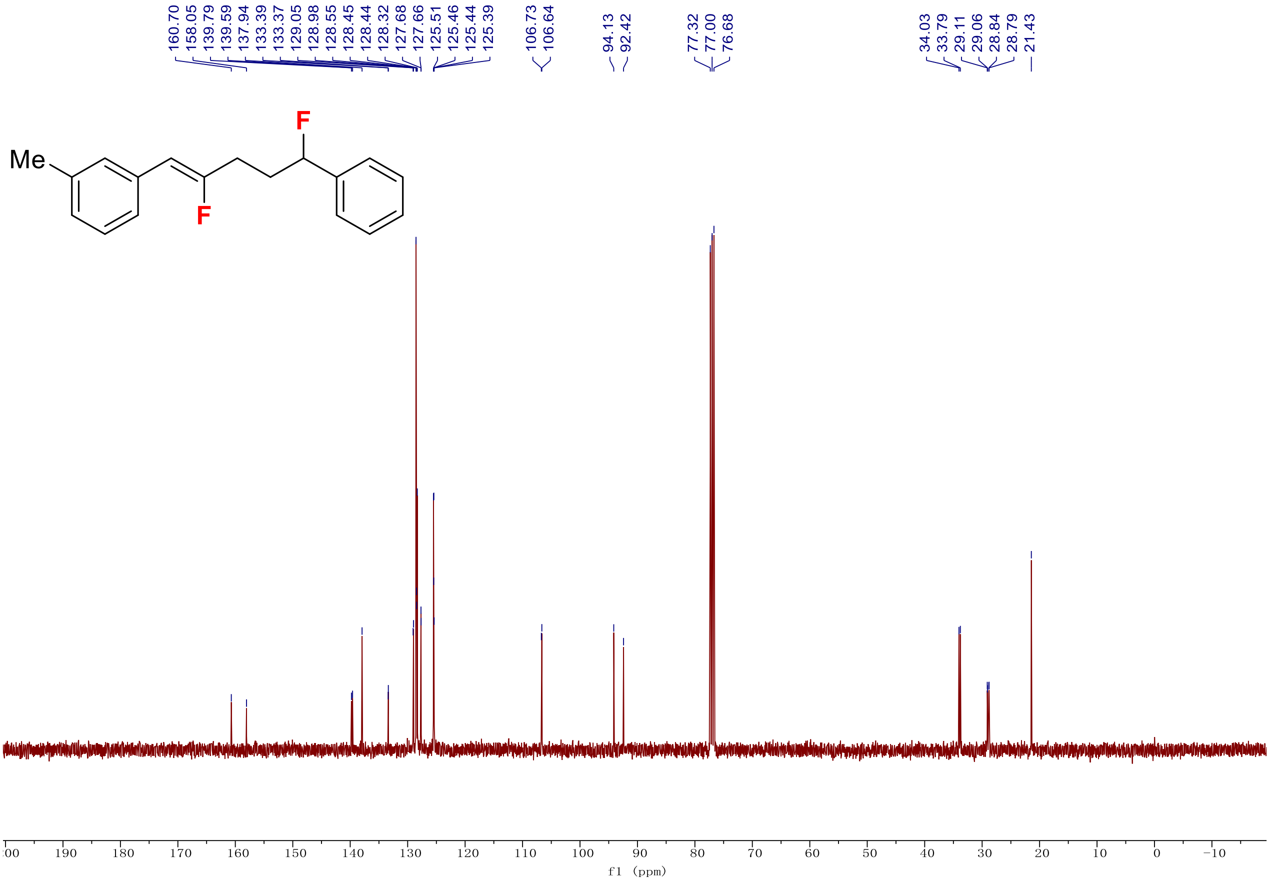

^{19}F NMR (376 MHz, $CDCl_3$) spectrum of 4h

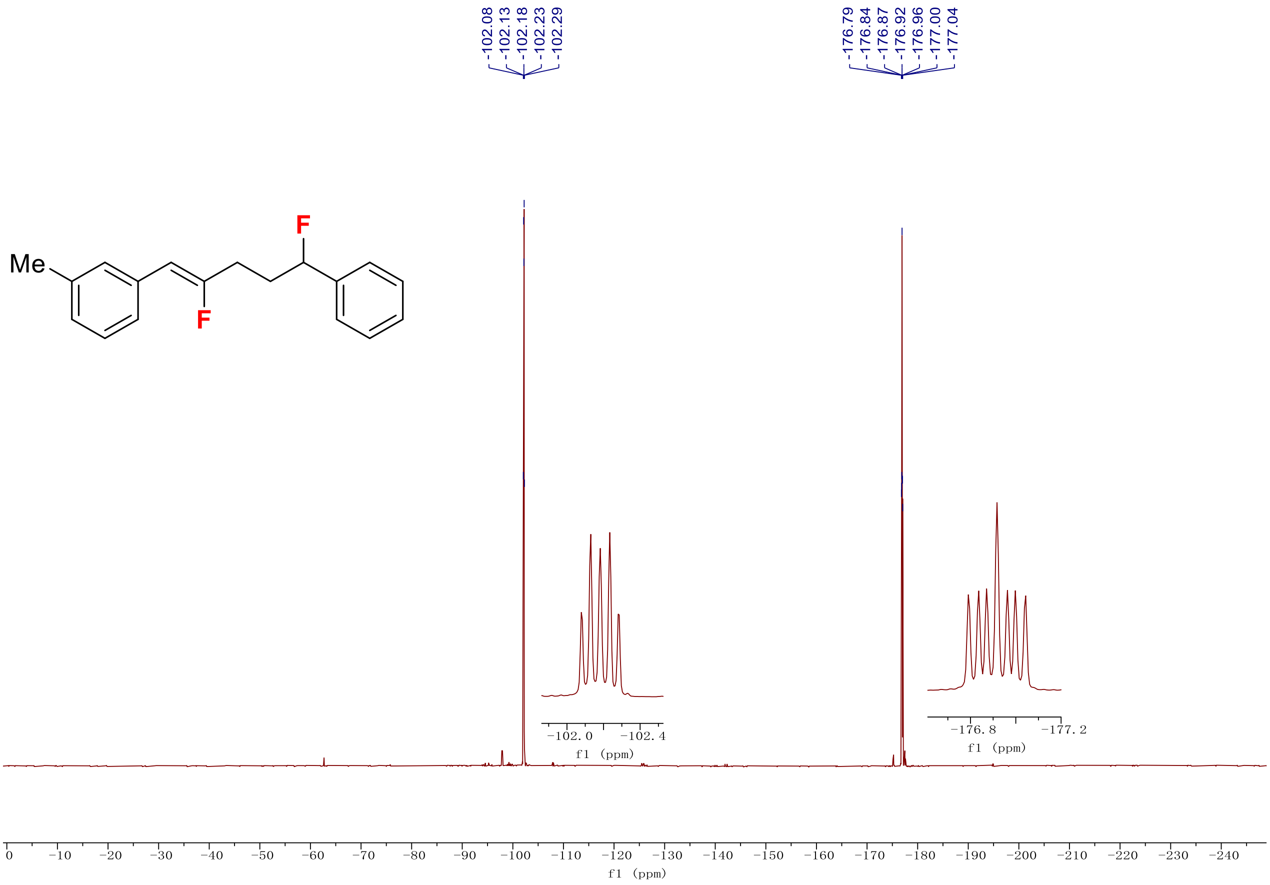

^{1}H NMR (400 MHz, CDCl3) spectrum of 5a

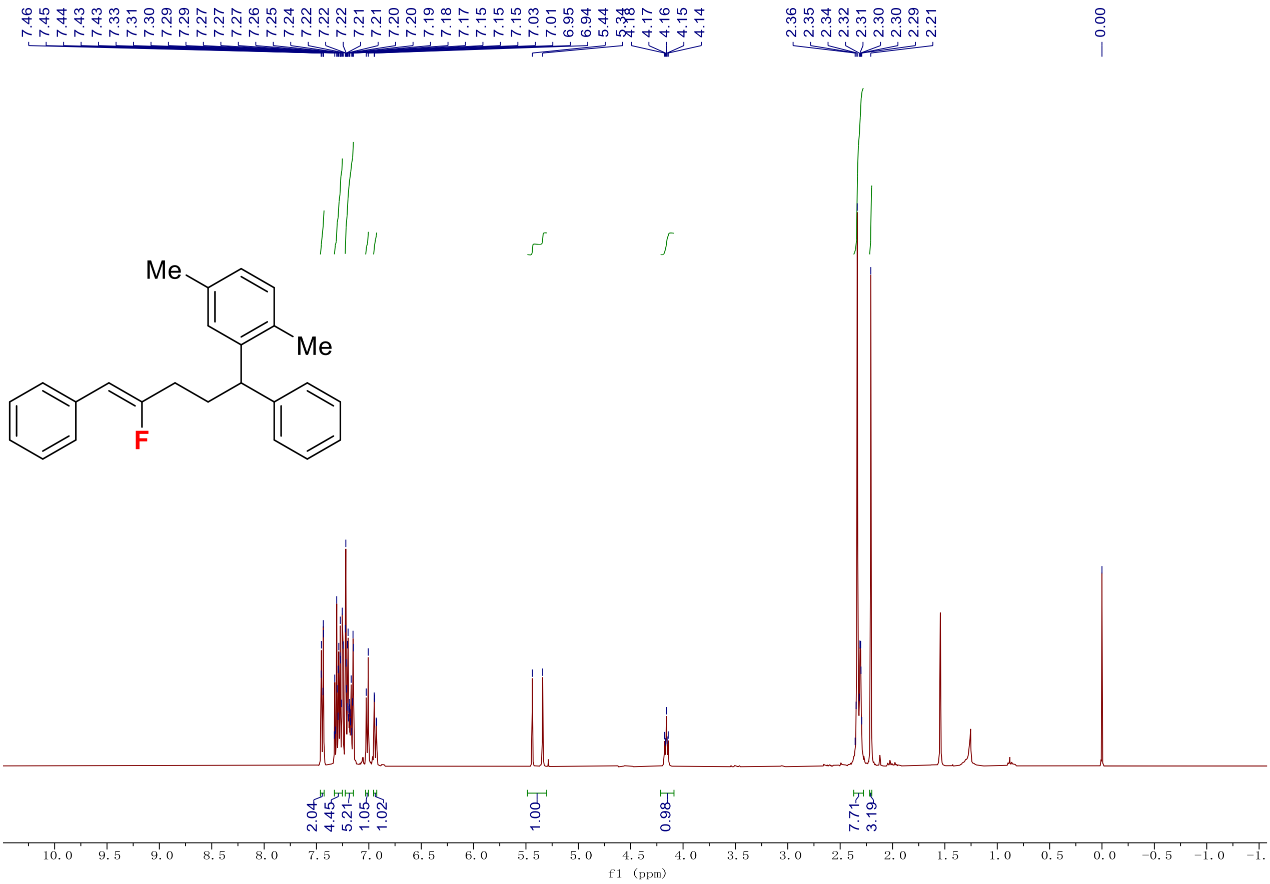

^{13}C NMR (101 MHz, CDCl3) spectrum of 5a

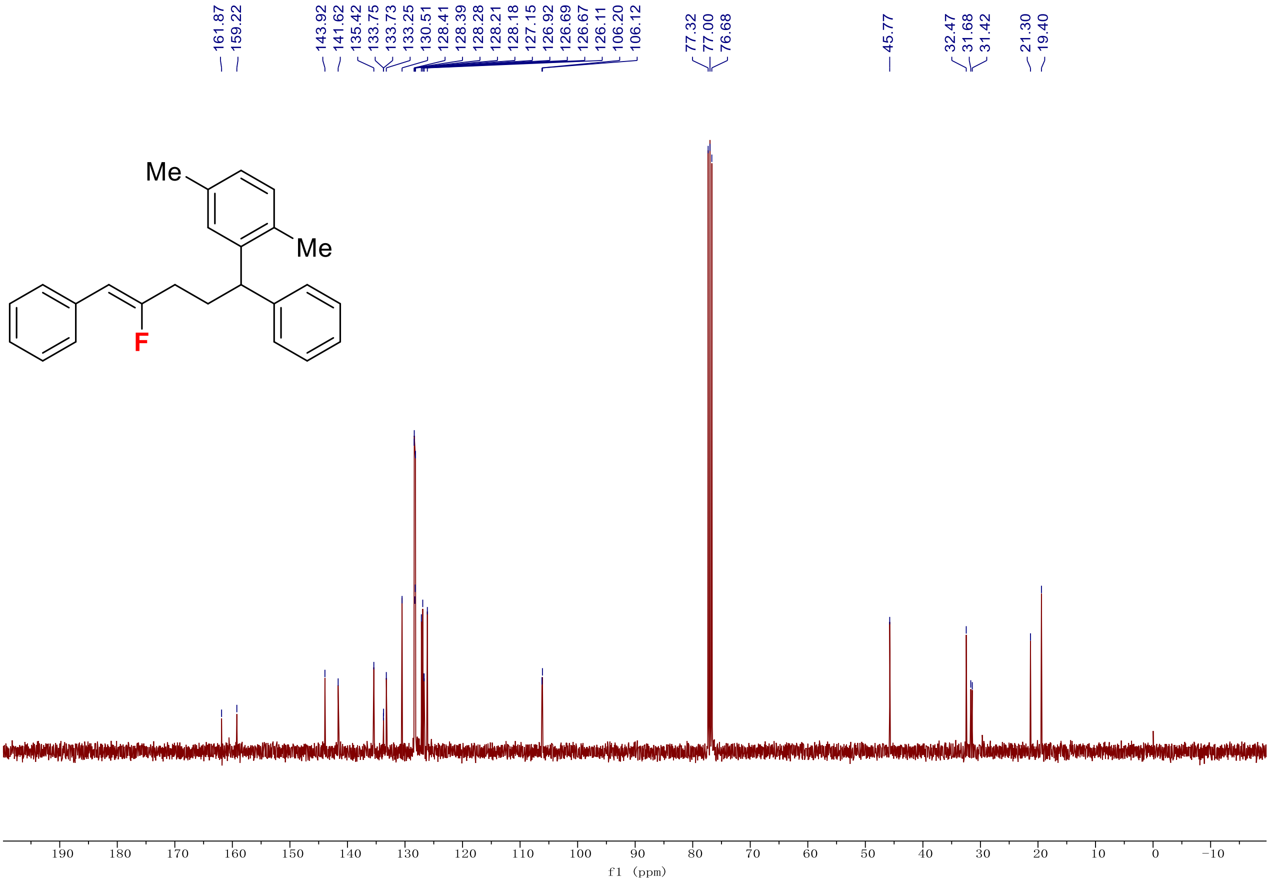

^{19}F NMR (376 MHz, CDCl$_3$) spectrum of 5a

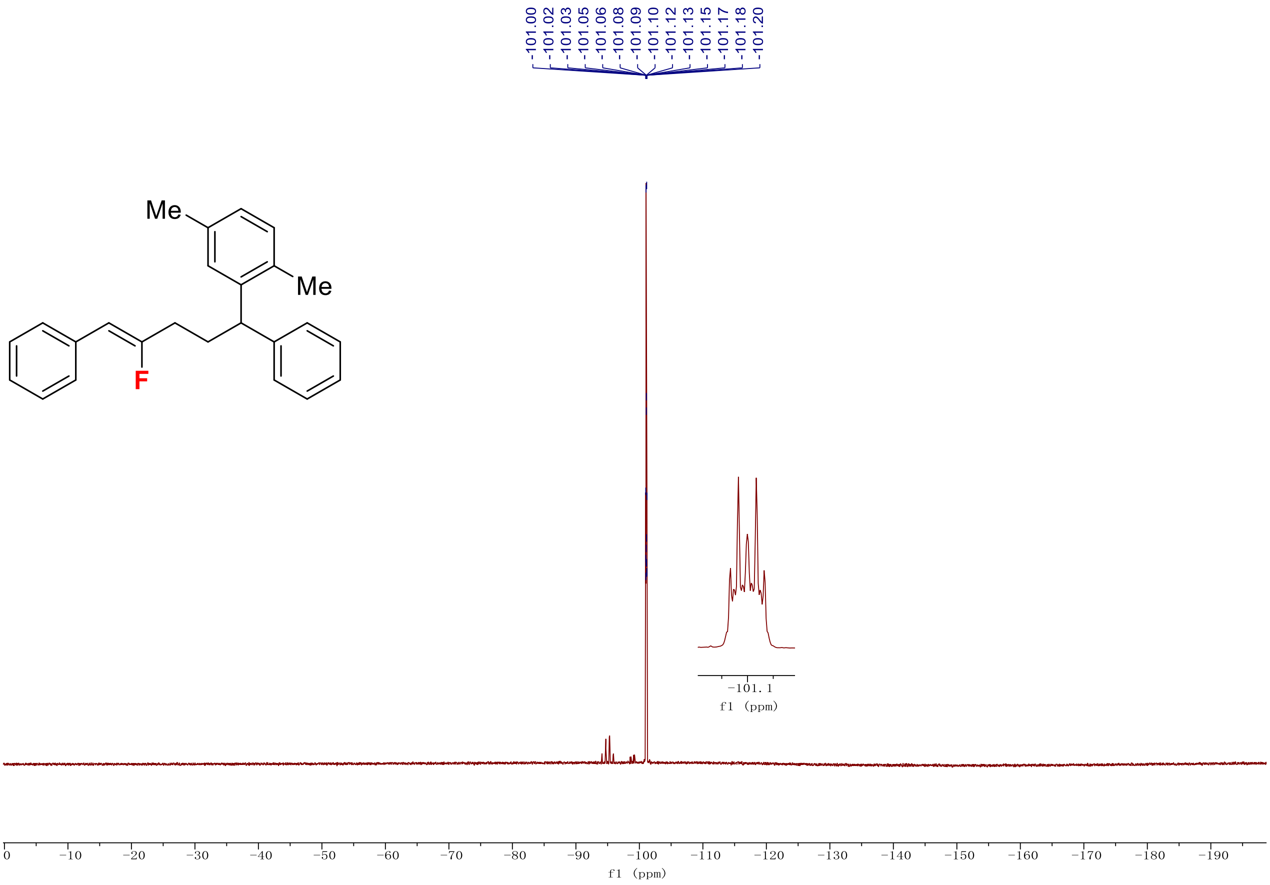

^{1}H NMR (400 MHz, CDCl3) spectrum of 5b

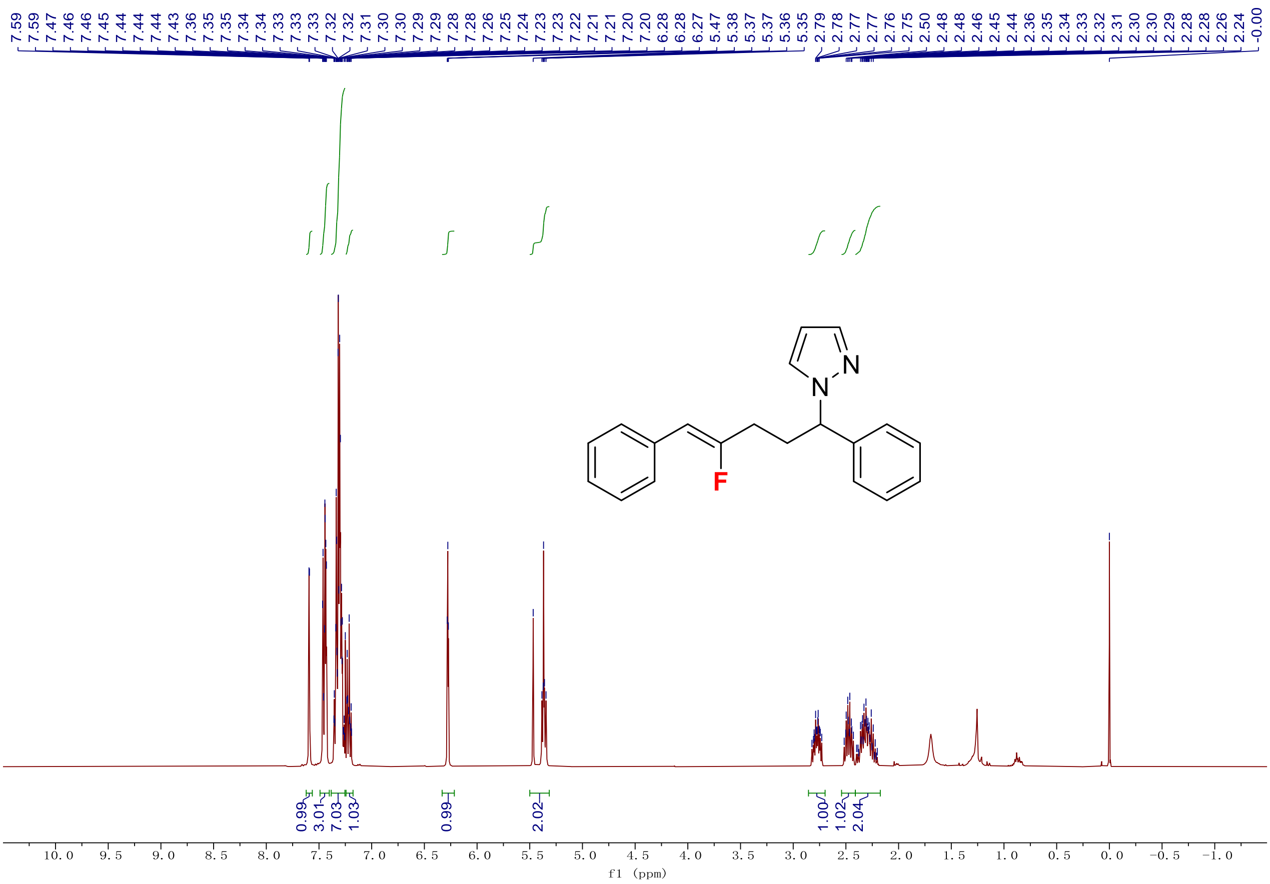

^{13}C NMR (101 MHz, CDCl3) spectrum of 5b

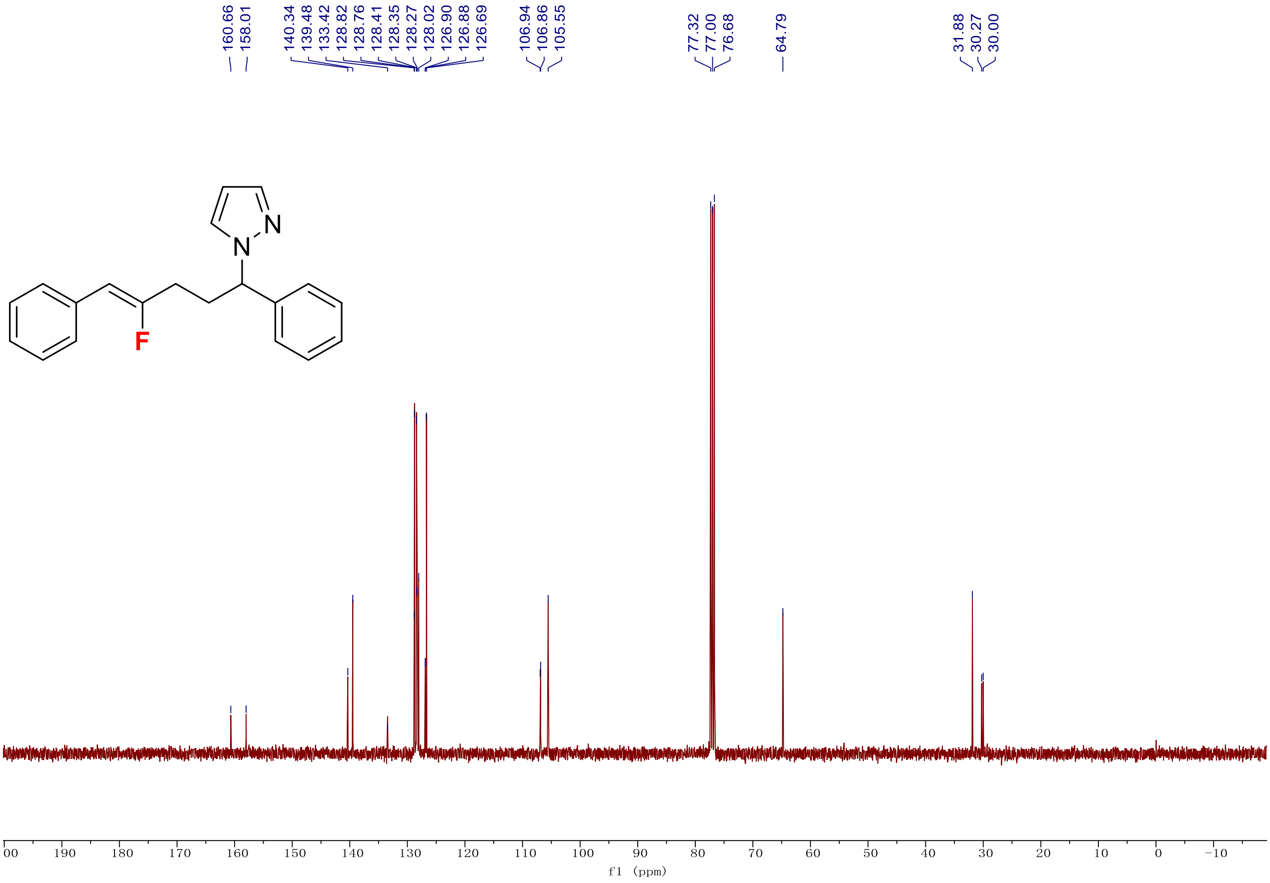

^{19}F NMR (376 MHz, $CDCl_3$) spectrum of 5b

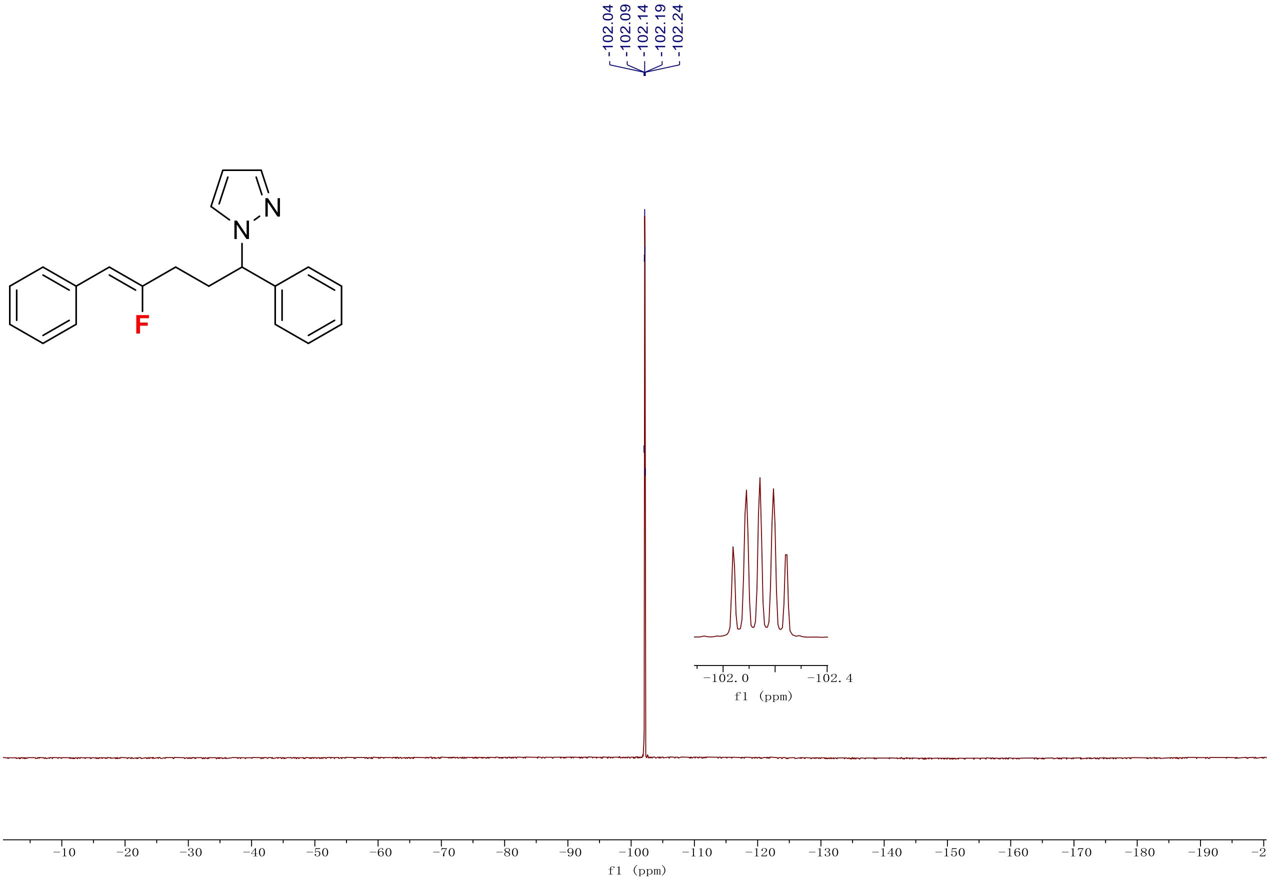

^{1}H NMR (400 MHz, $CDCl_3$) spectrum of 5c

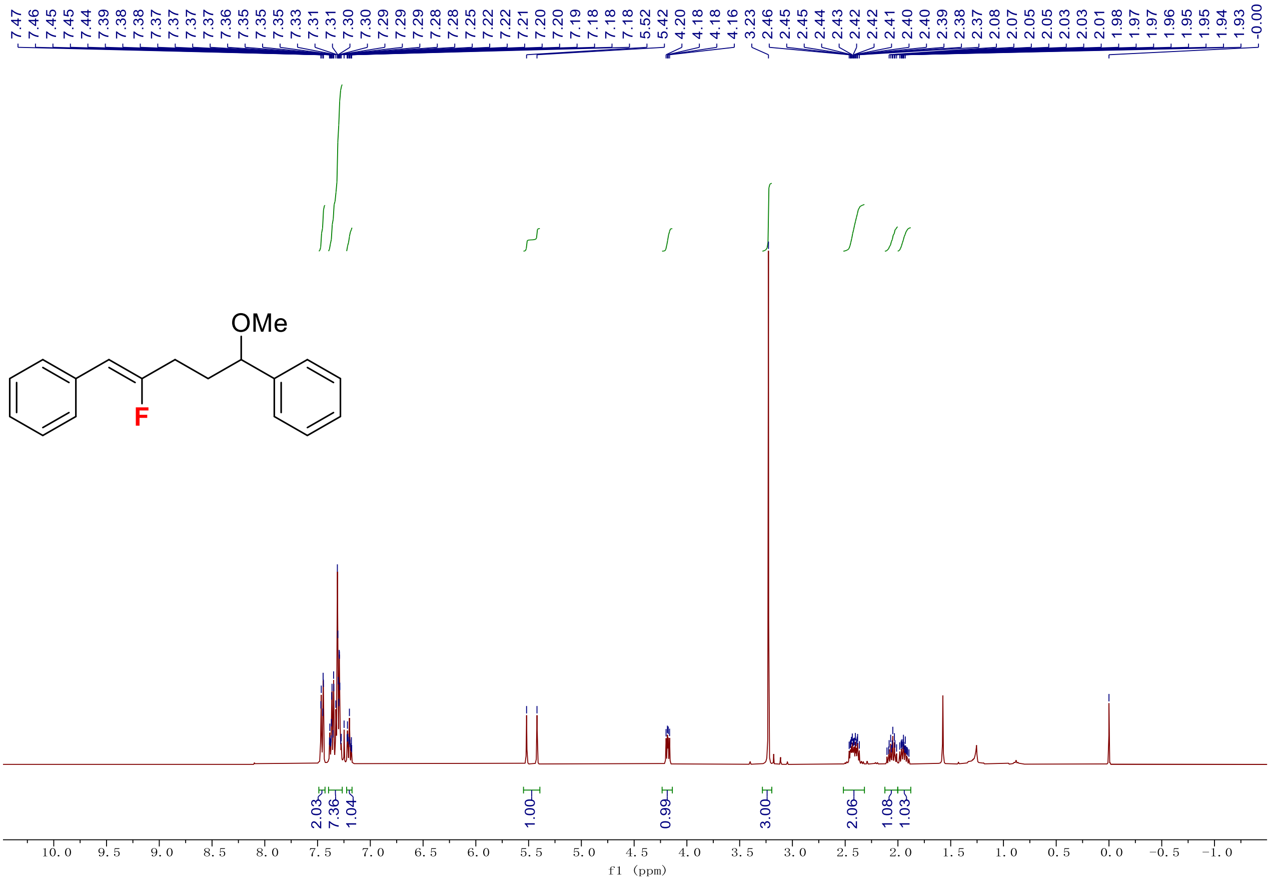

^{13}C NMR (101 MHz, $CDCl_3$) spectrum of 5c

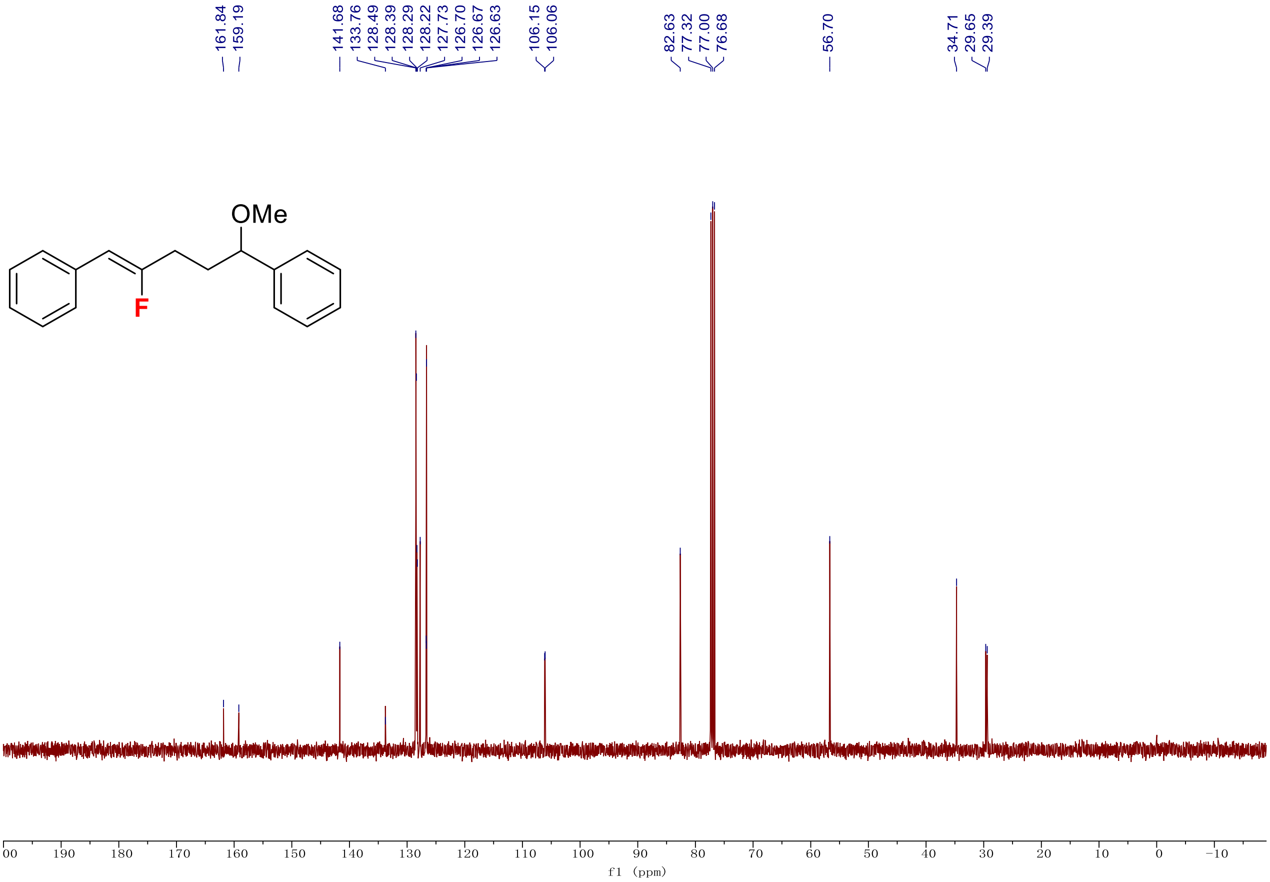

^{19}F NMR (376 MHz, $CDCl_3$) spectrum of 5c

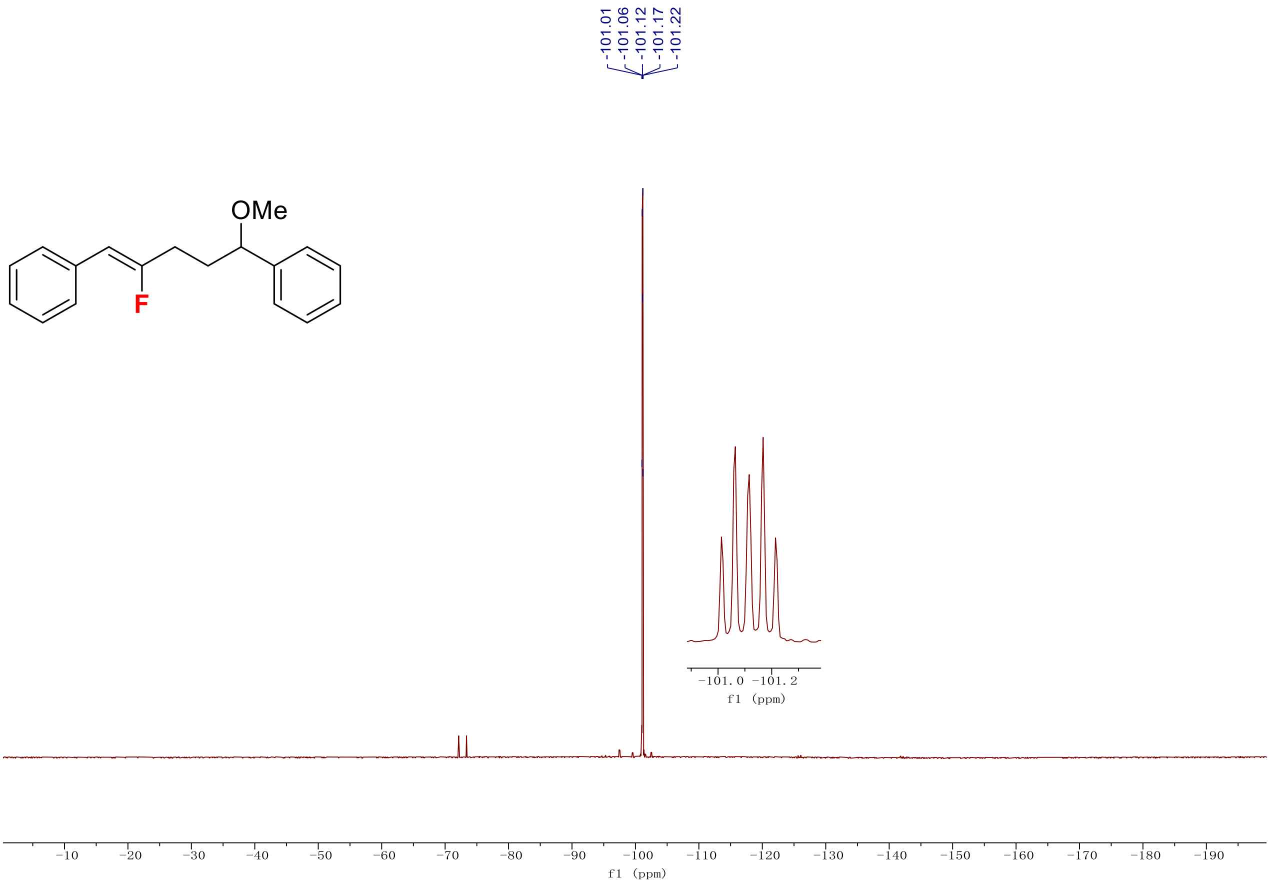

^{1}H NMR (400 MHz, $CDCl_3$) spectrum of 5d

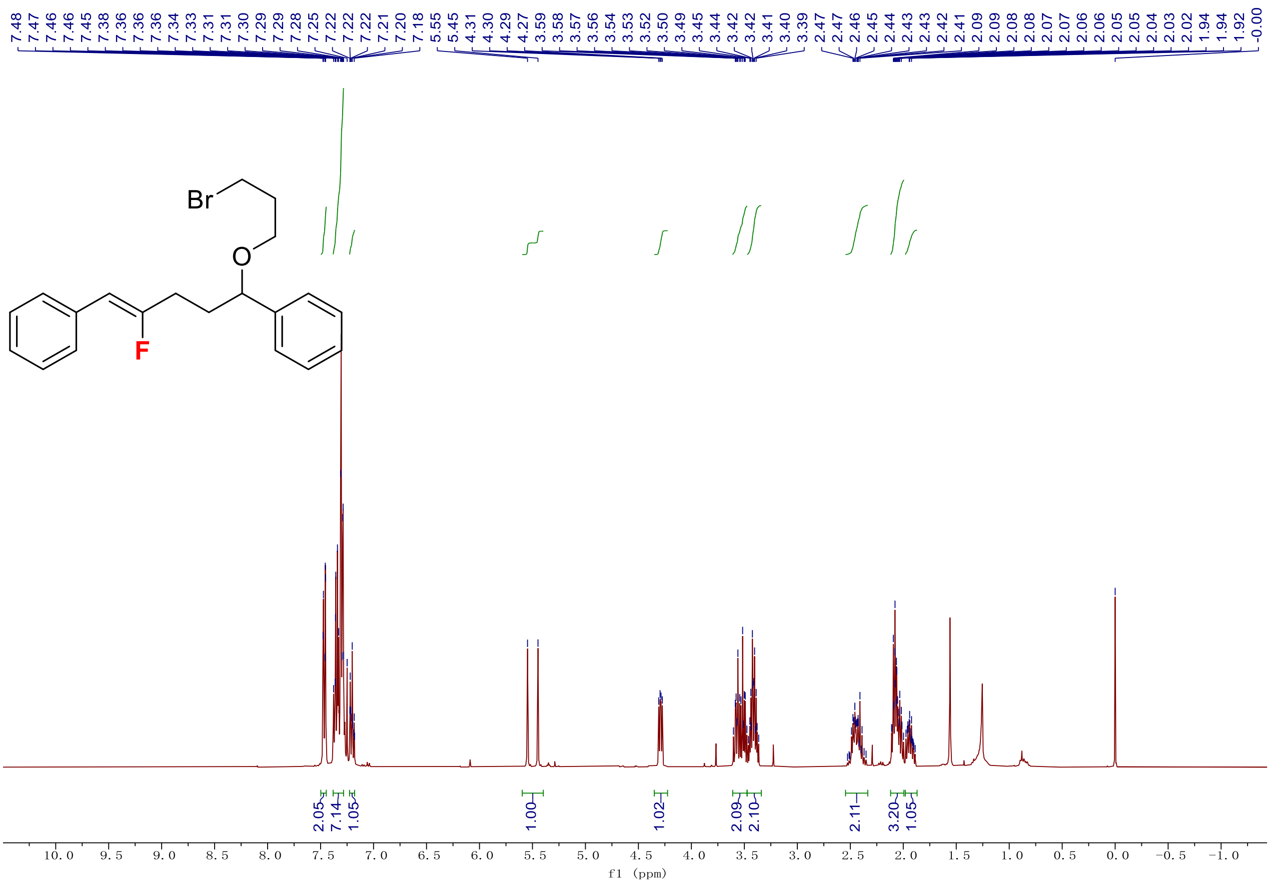

^{13}C NMR (101 MHz, $CDCl_3$) spectrum of 5d

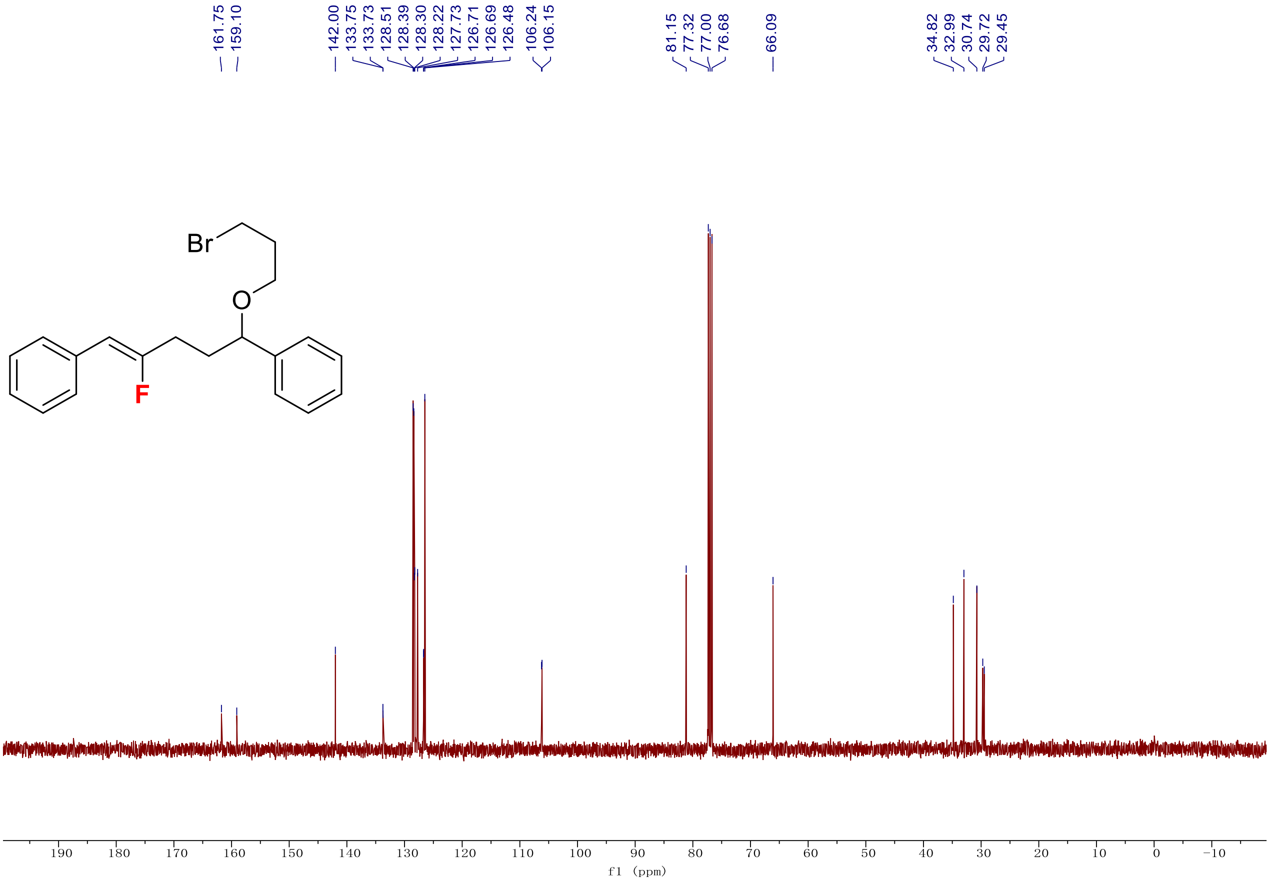

^{19}F NMR (376 MHz, $CDCl_3$) spectrum of 5d

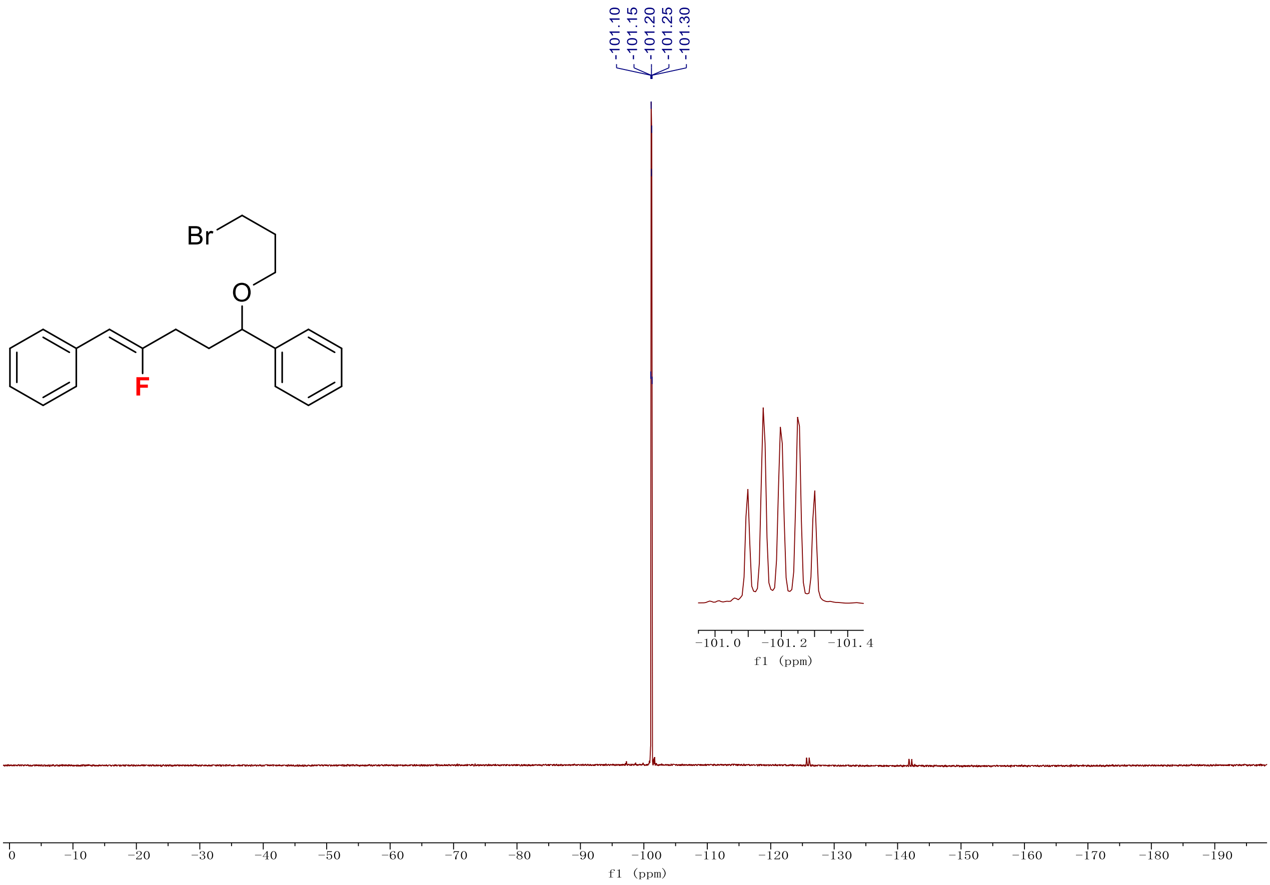

^{1}H NMR (400 MHz, CDCl3) spectrum of 5e

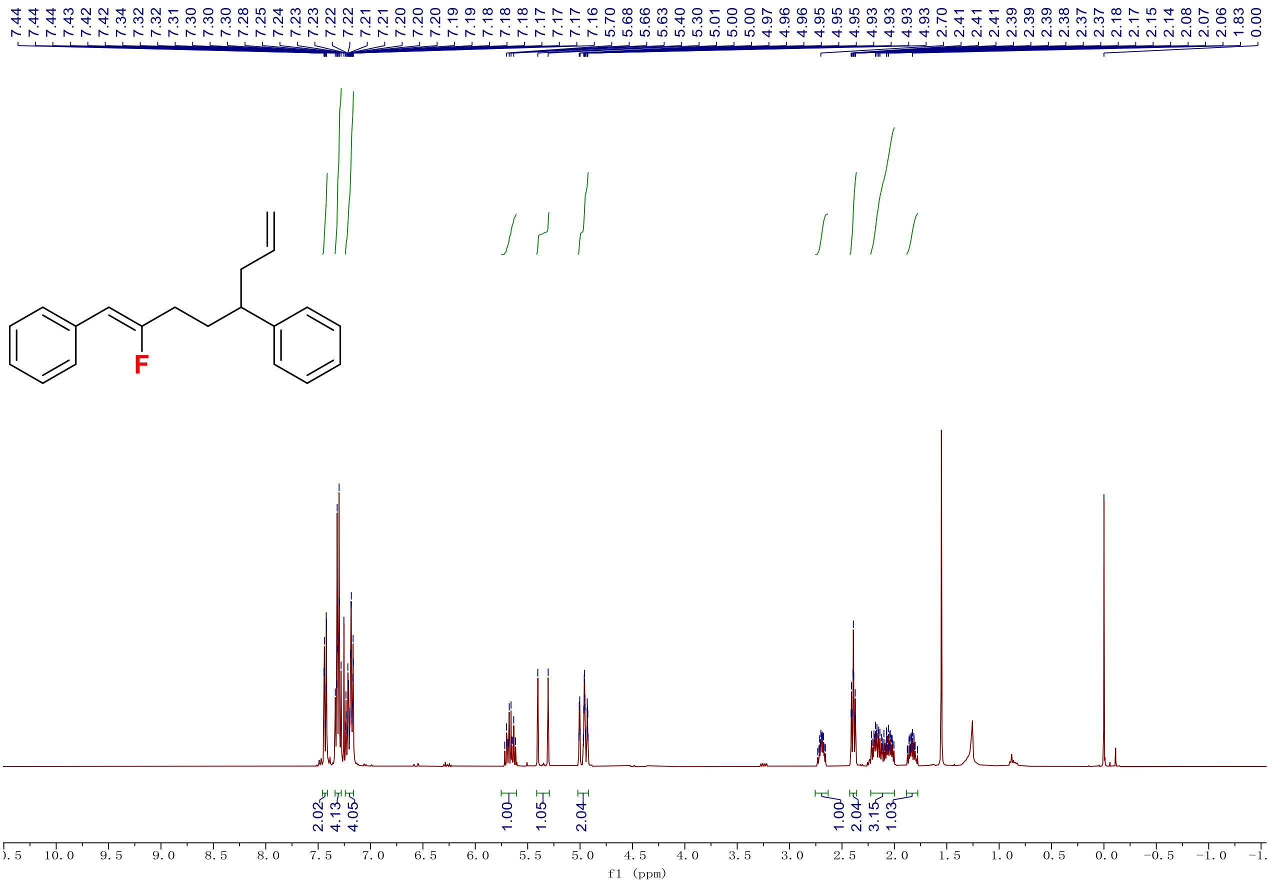

^{13}C NMR (101 MHz, CDCl3) spectrum of 5e

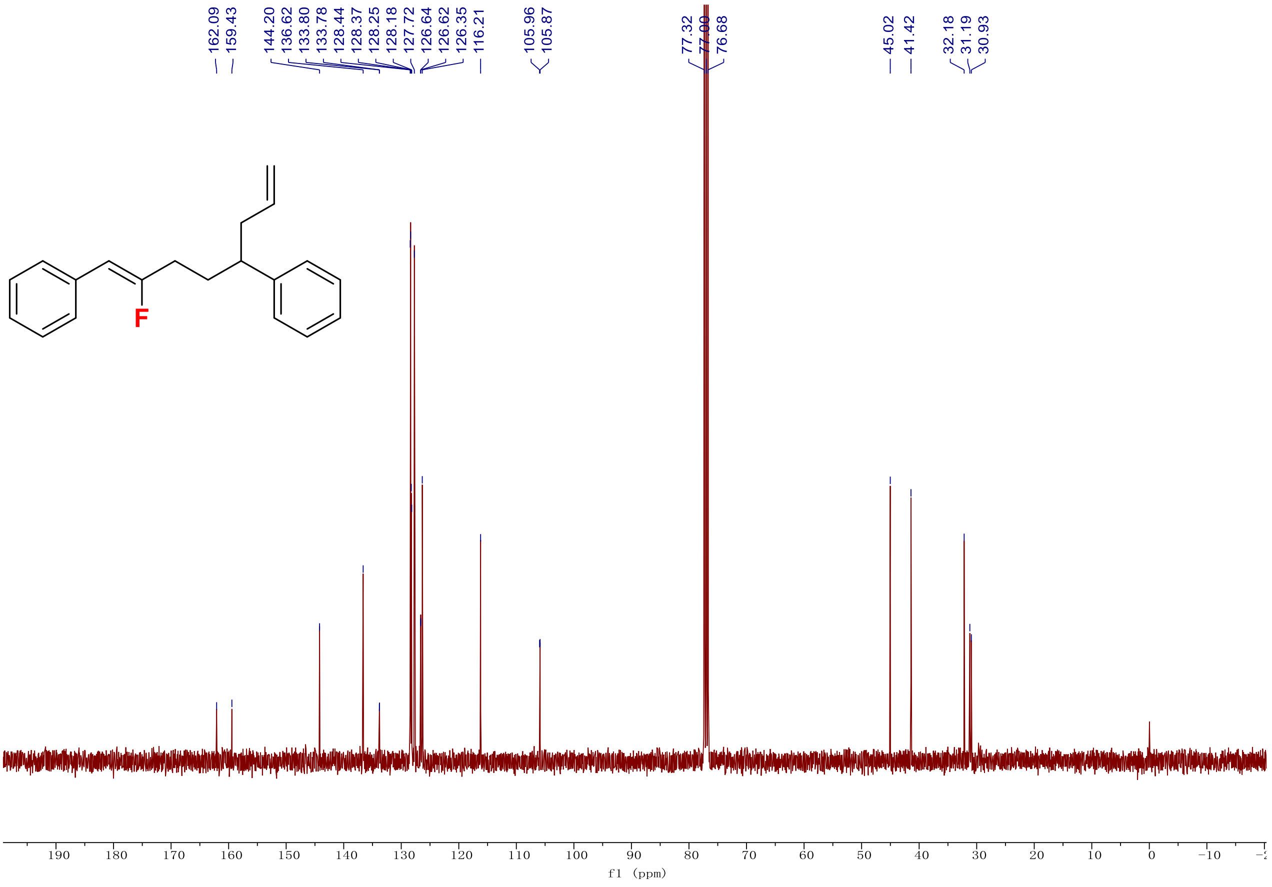

^{19}F NMR (376 MHz, $CDCl_3$) spectrum of 5e

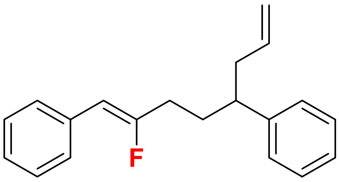

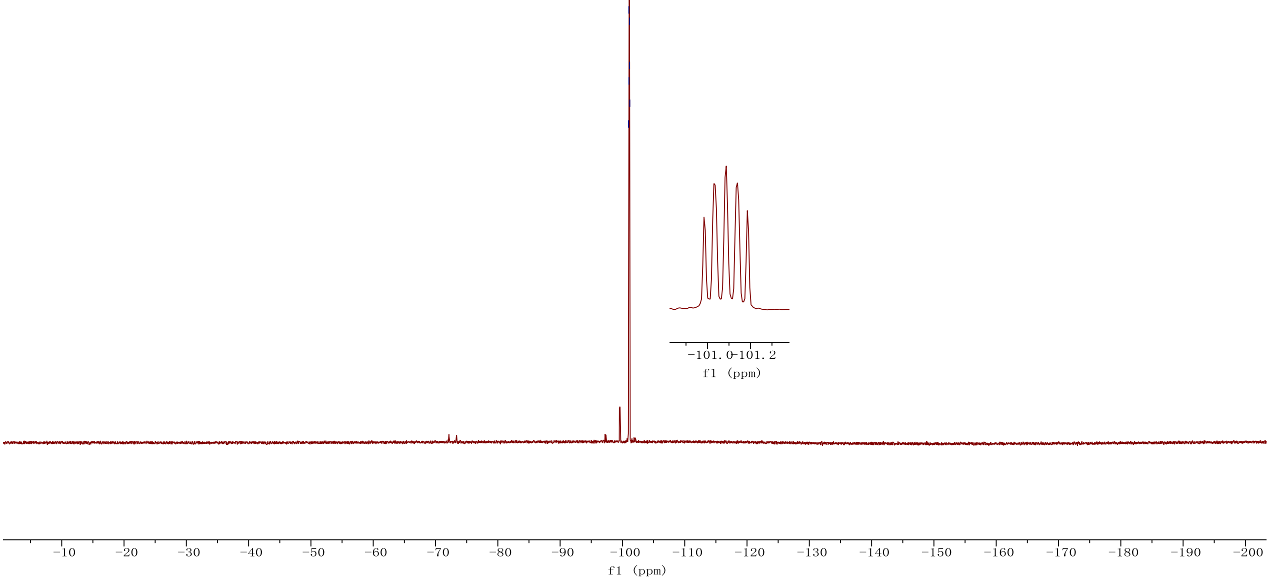

^{1}H NMR (400 MHz, $CDCl_3$) spectrum of 6a

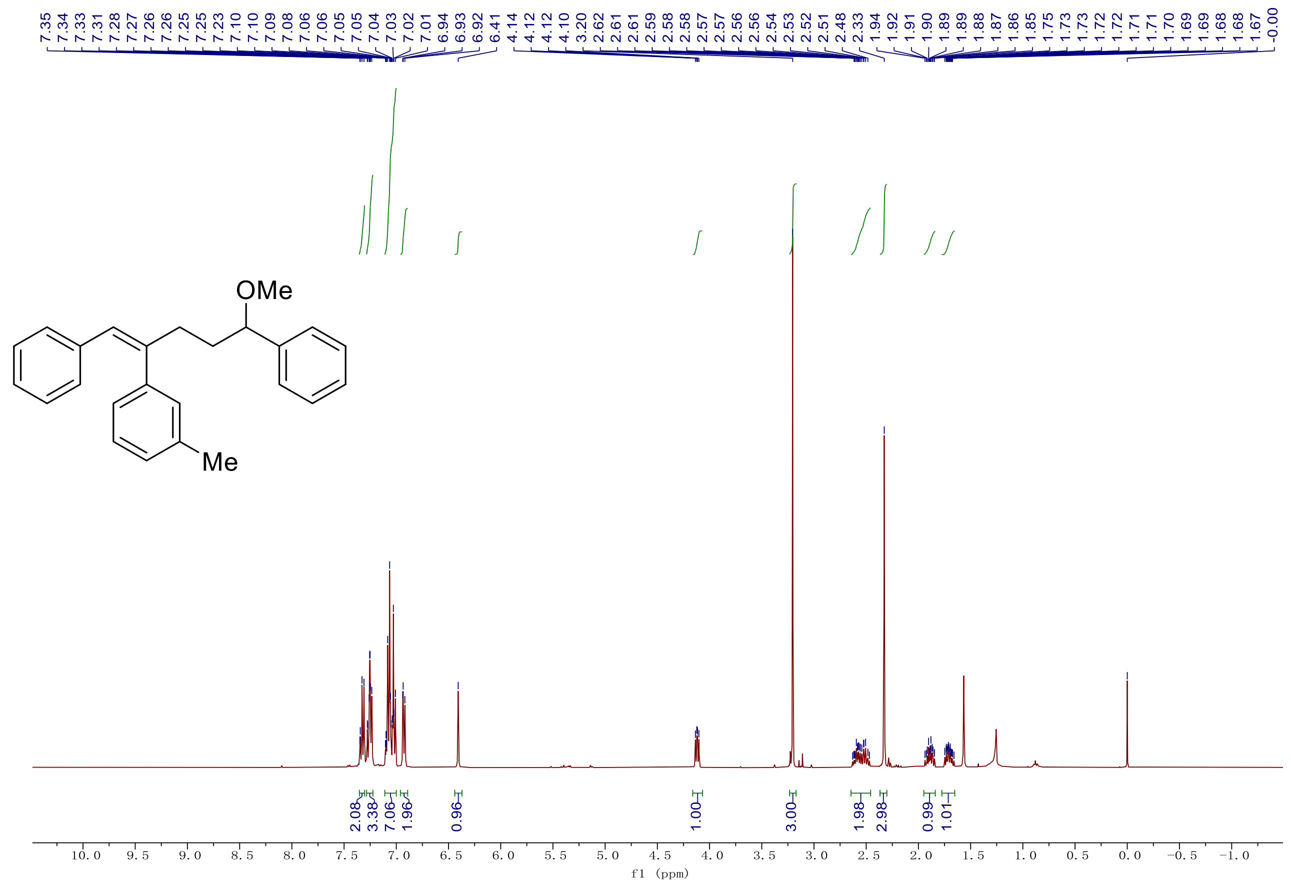

^{13}C NMR (101 MHz, $CDCl_3$) spectrum of 6a

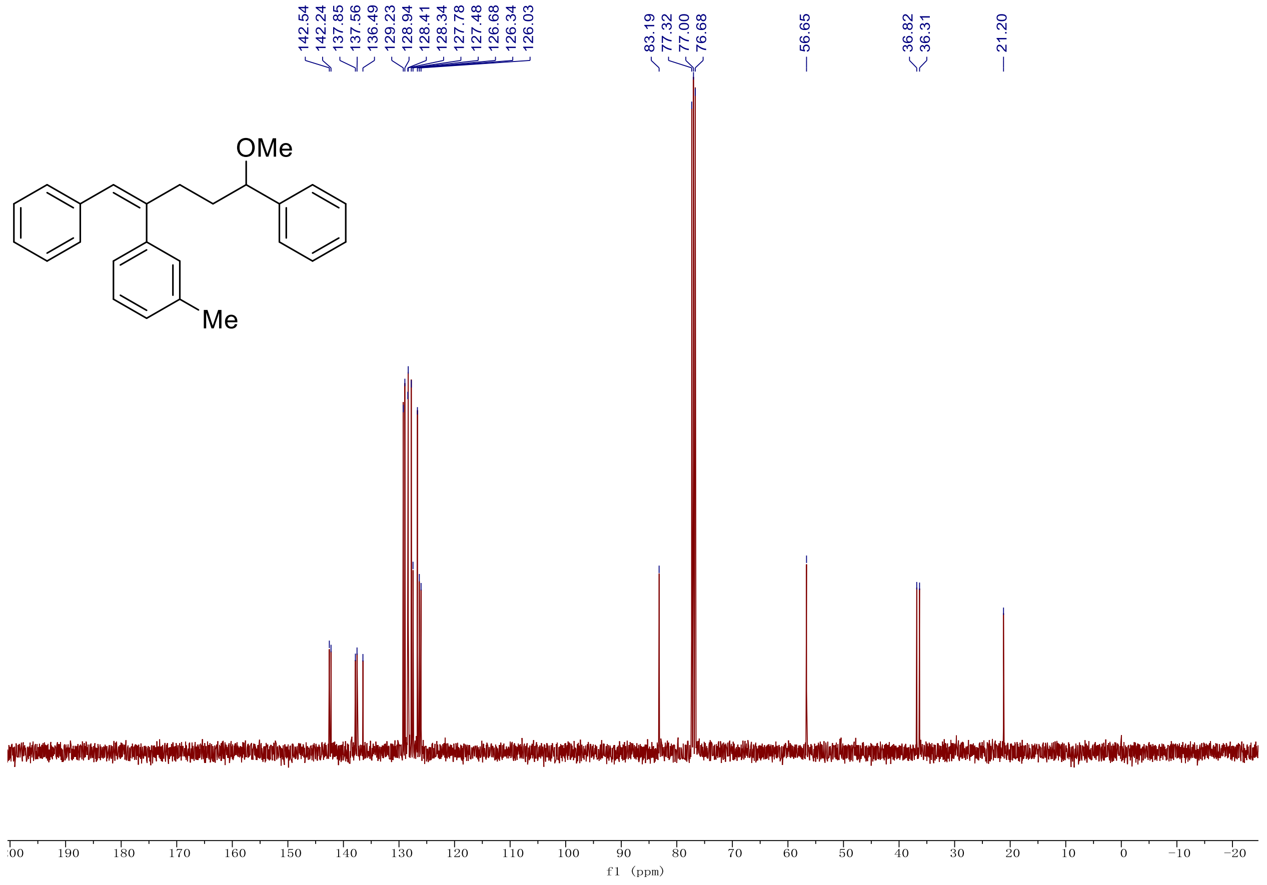

^{1}H NMR (400 MHz, CDCl3) spectrum of 7a

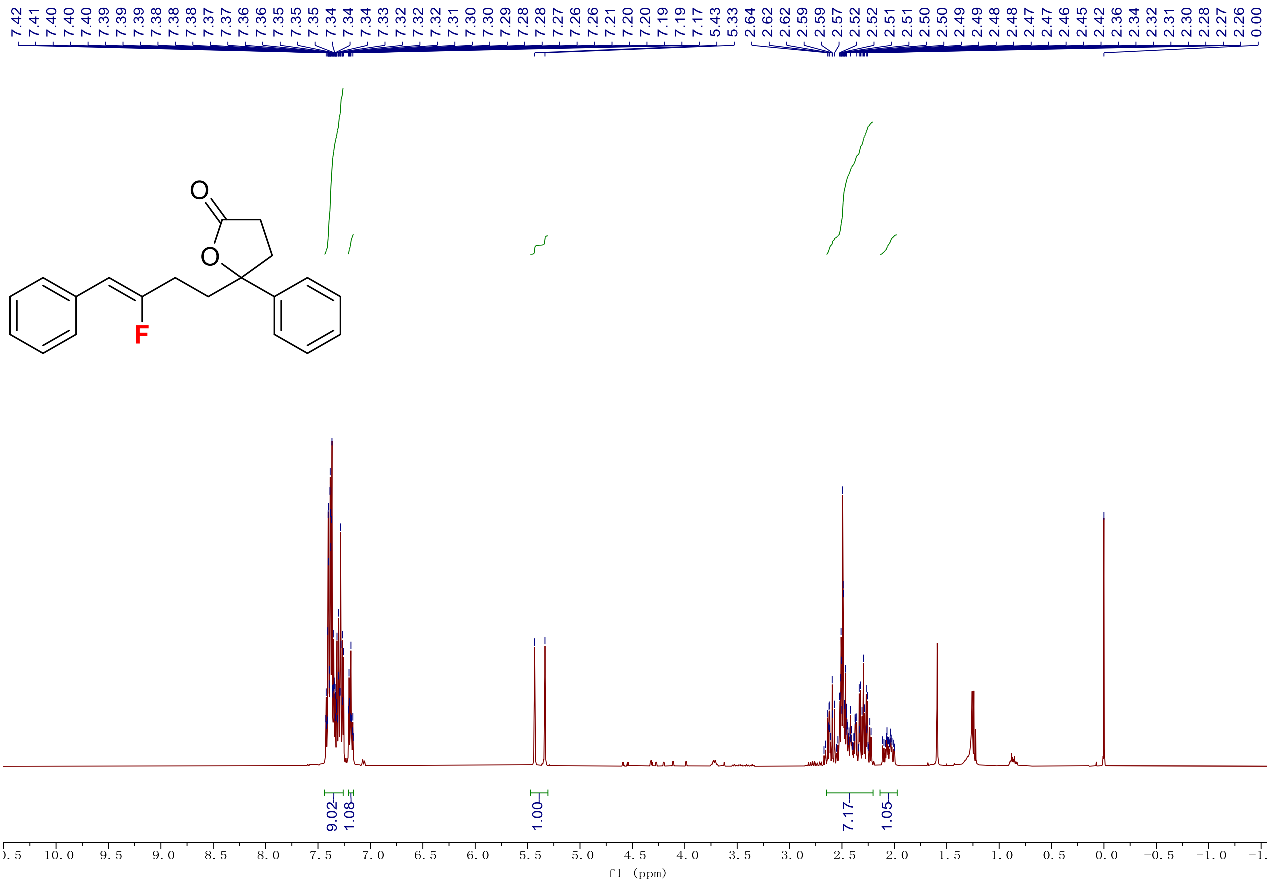

^{13}C NMR (101 MHz, CDCl3) spectrum of 7a

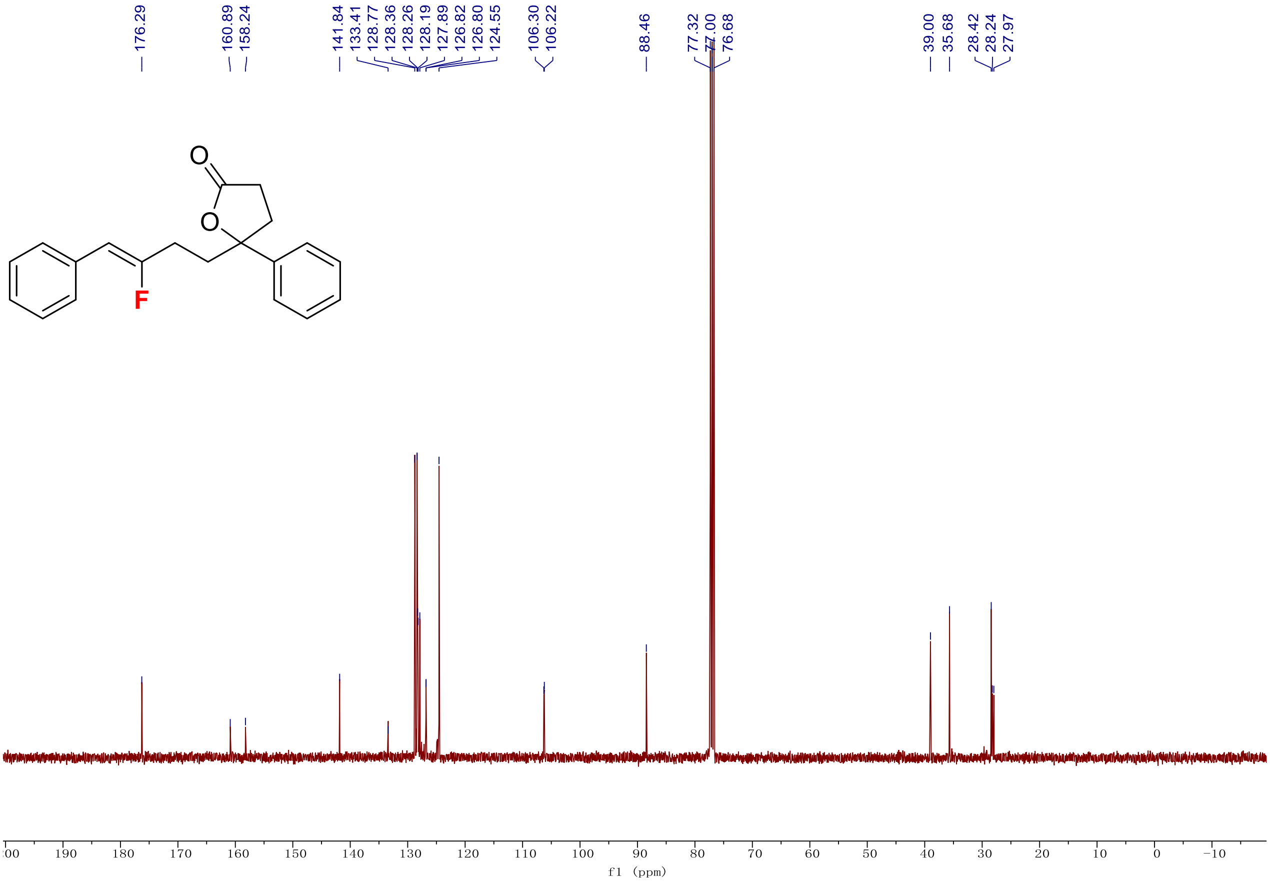

^{19}F NMR (376 MHz, CDCl3) spectrum of 7a

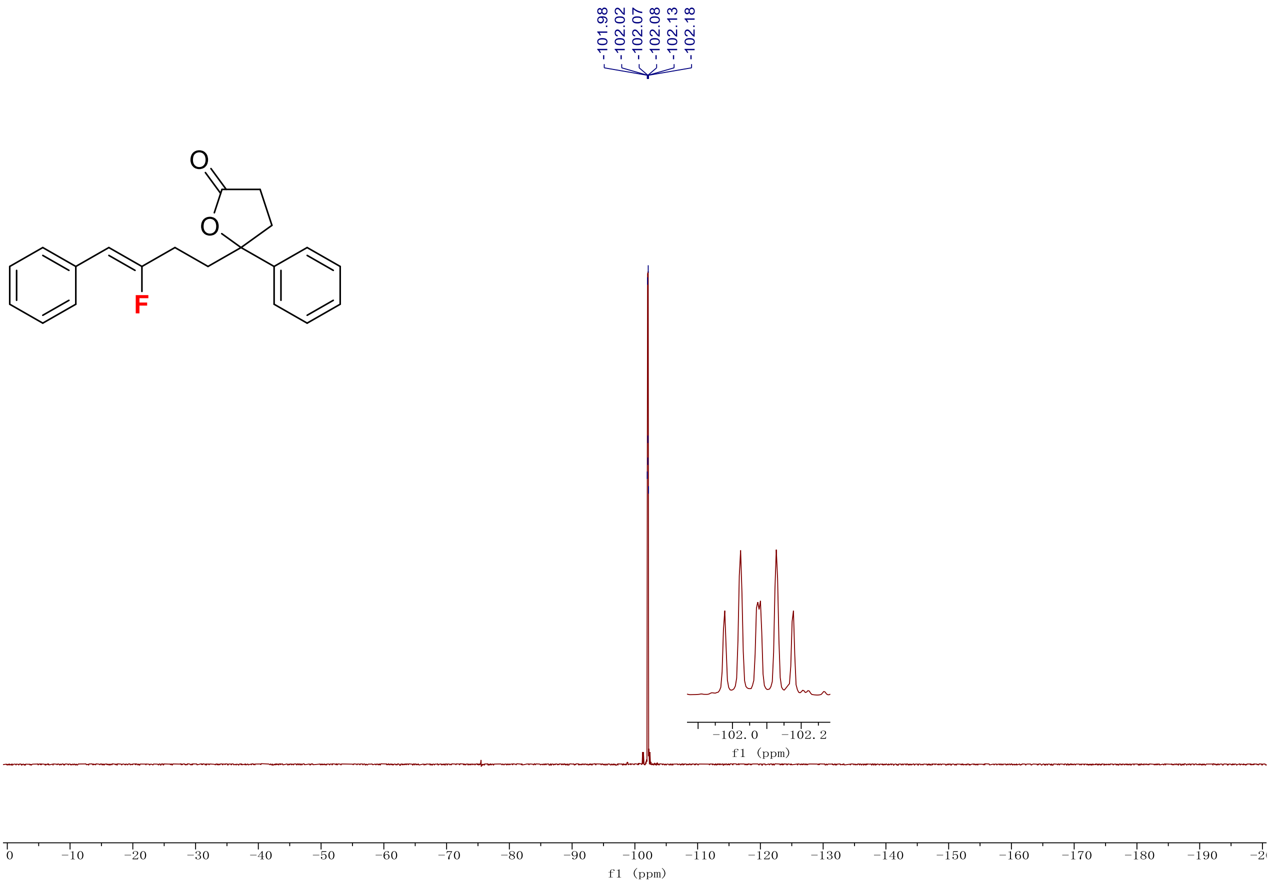

^{1}H NMR (400 MHz, CDCl3) spectrum of 3aj

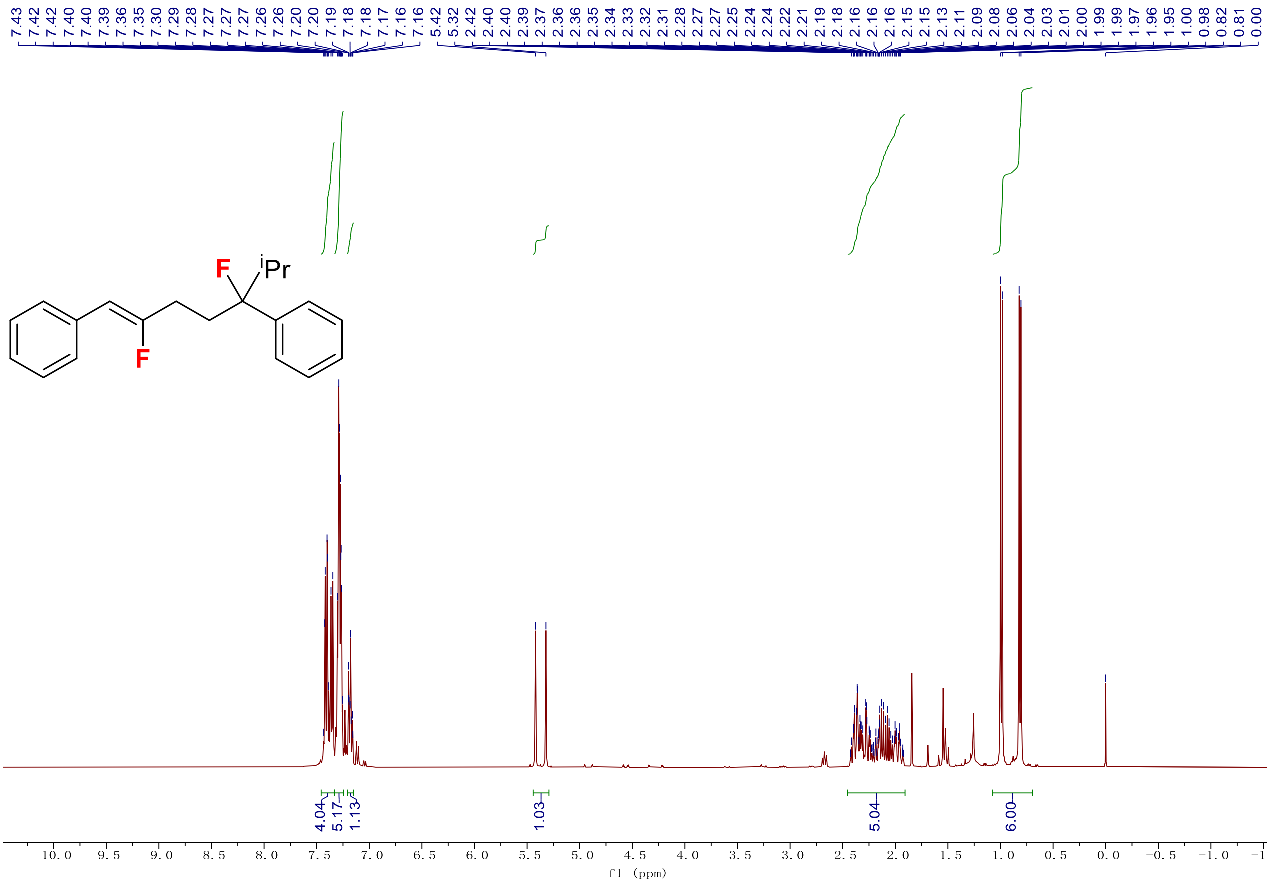

^{13}C NMR (101 MHz, CDCl3) spectrum of 3aj

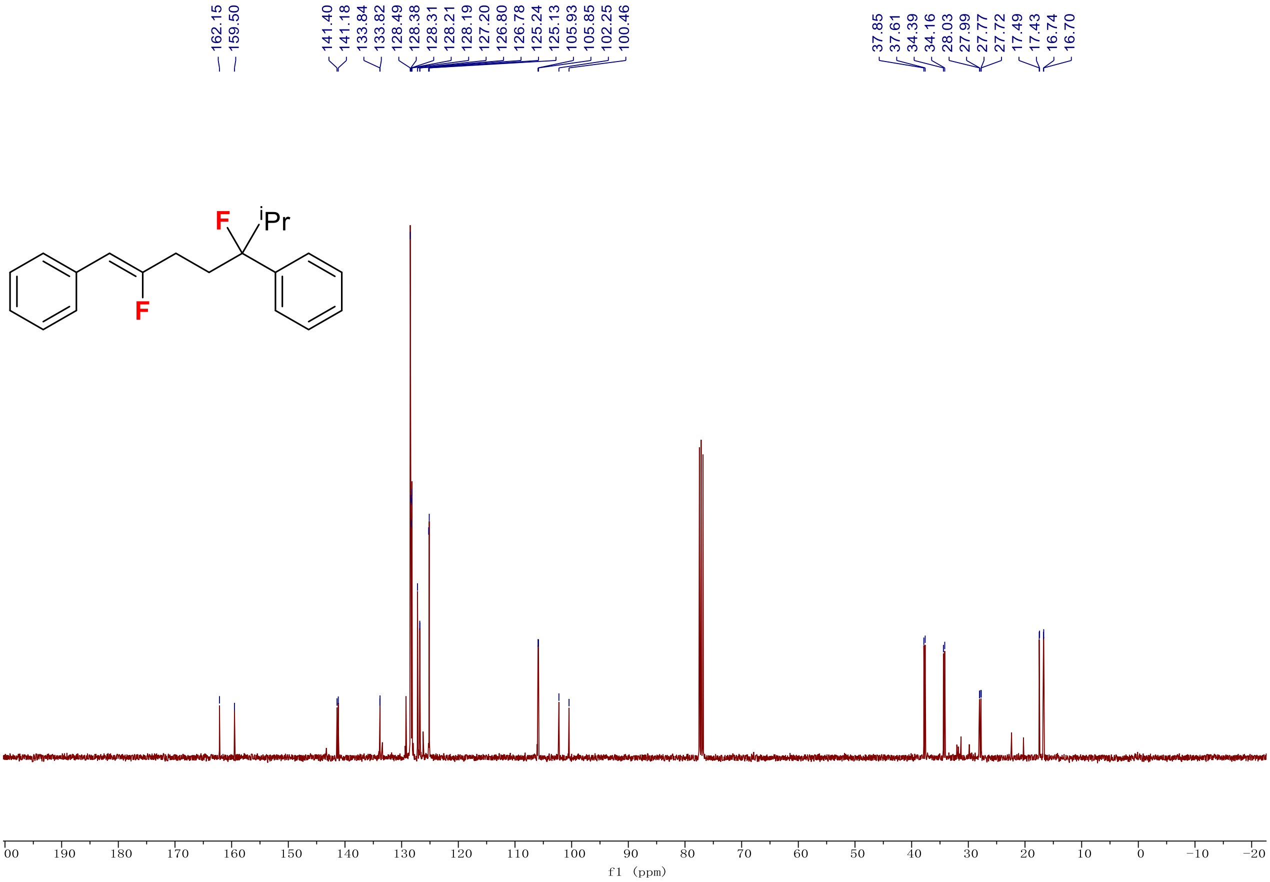

^{19}F NMR (376 MHz, $CDCl_3$) spectrum of 3aj

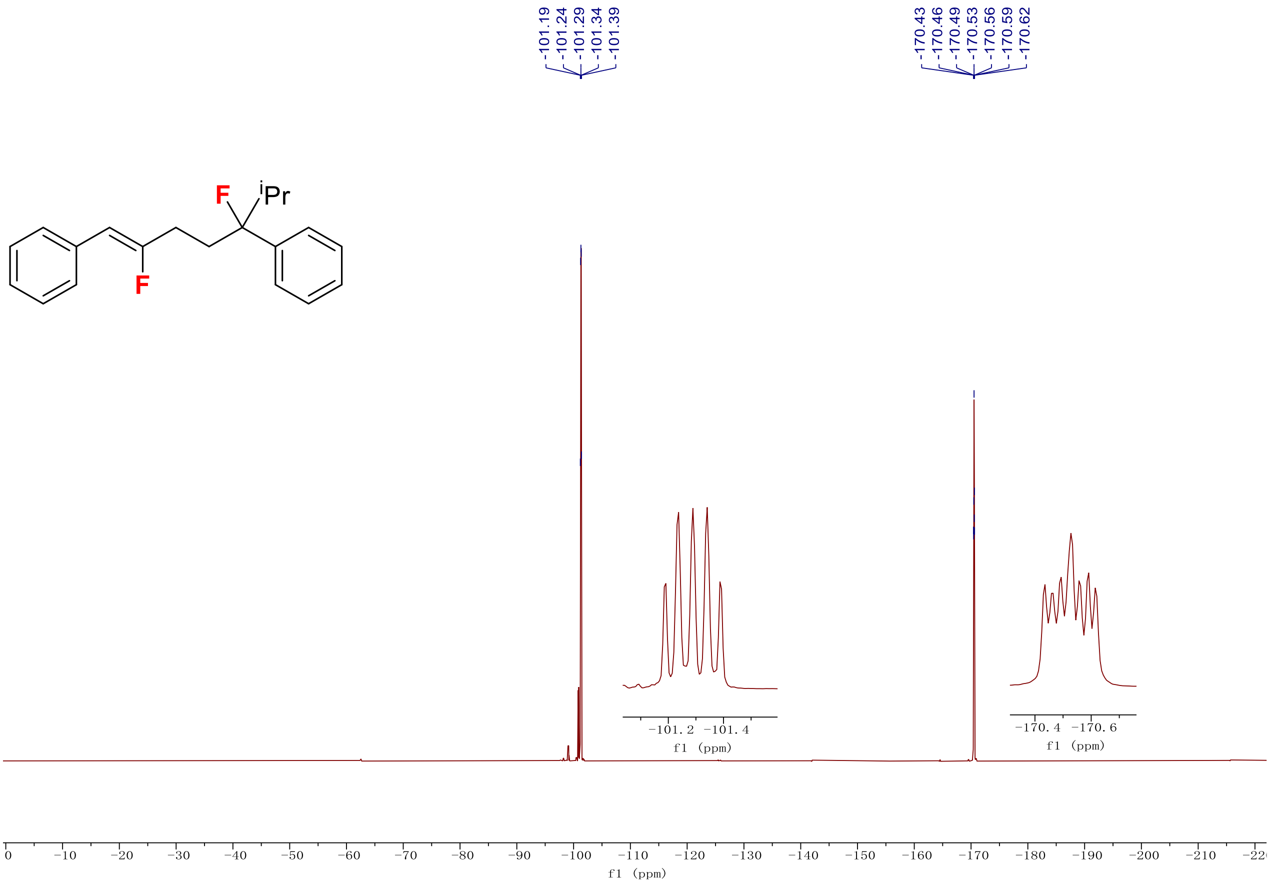

^{1}H NMR (400 MHz, $CDCl_3$) spectrum of 3aj'

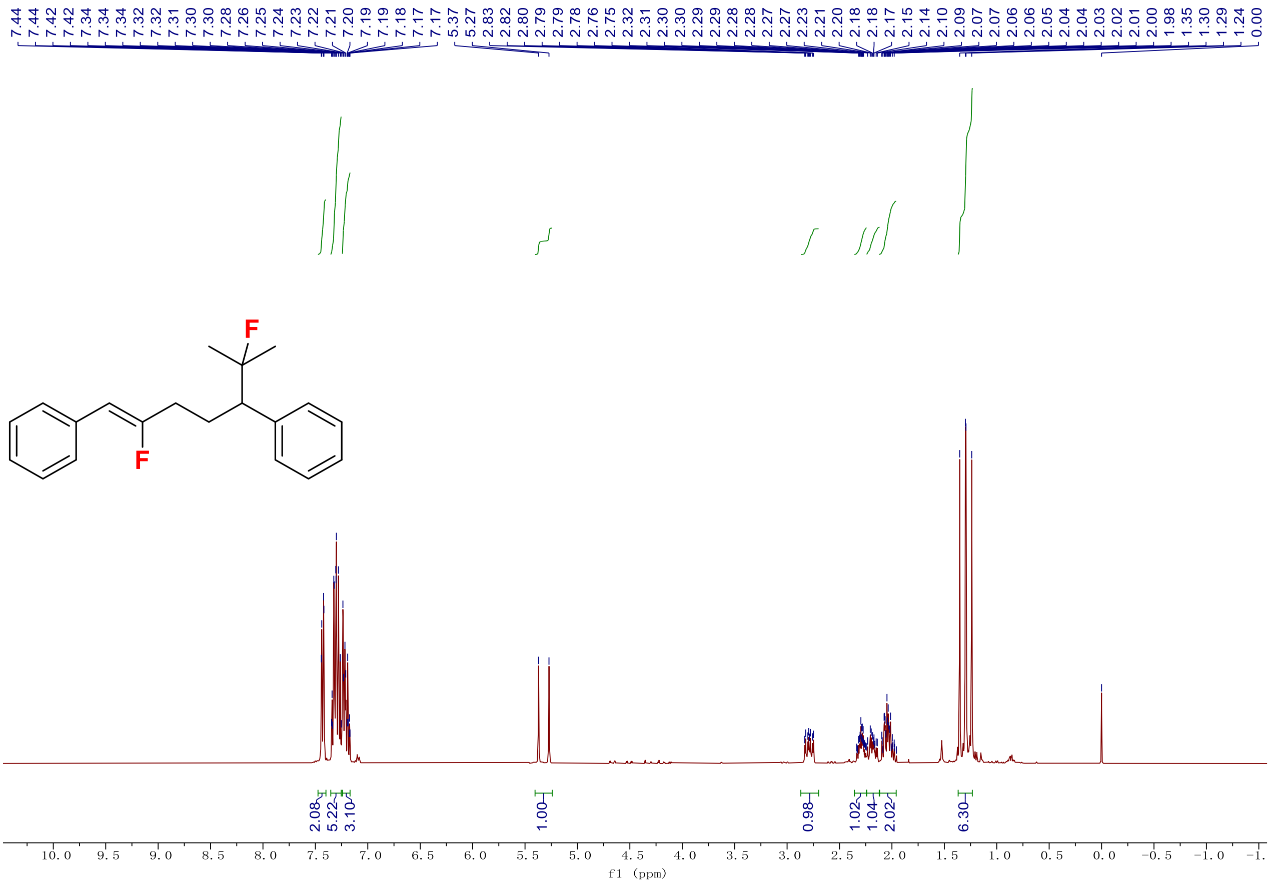

^{13}C NMR (101 MHz, $CDCl_3$) spectrum of 3aj'

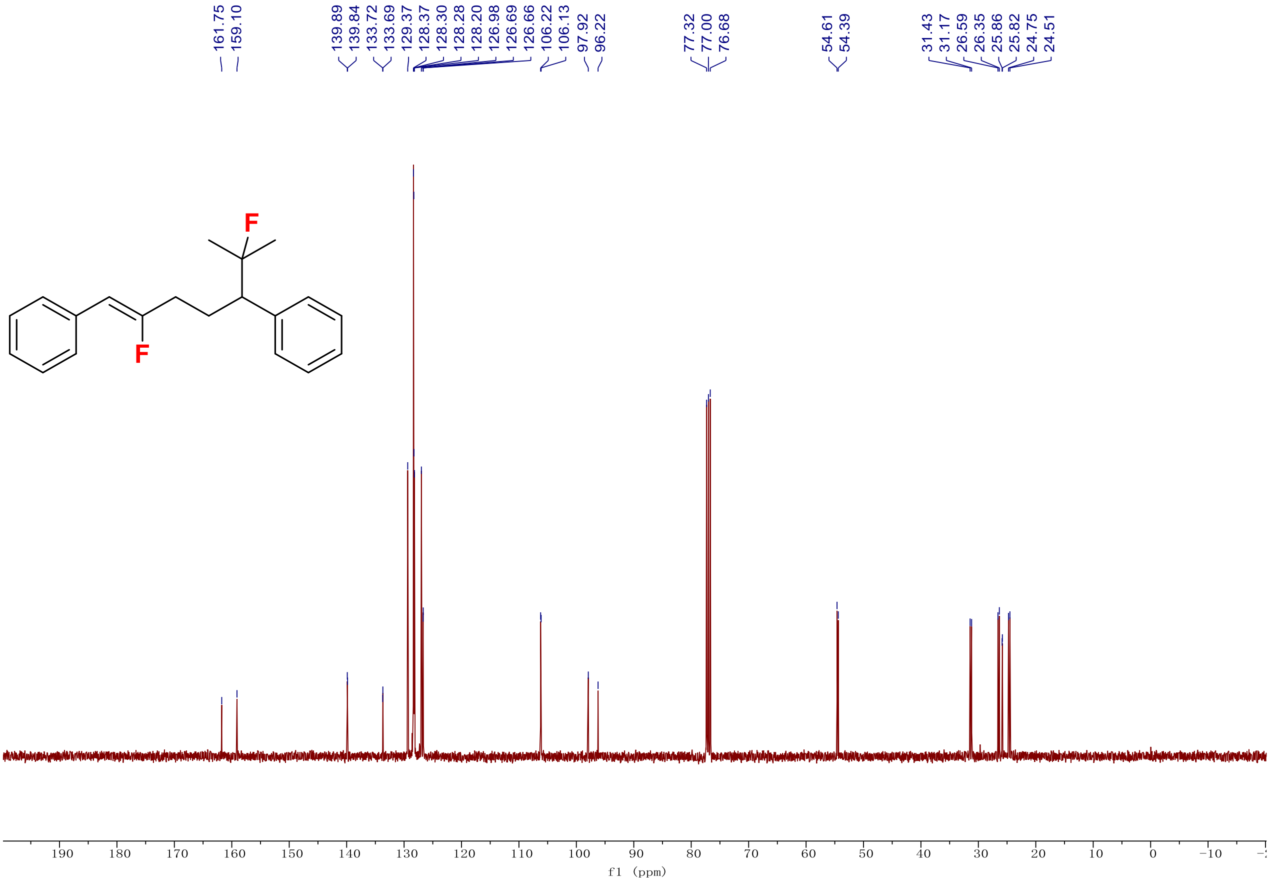

^{19}F NMR (376 MHz, $CDCl_3$) spectrum of 3aj'

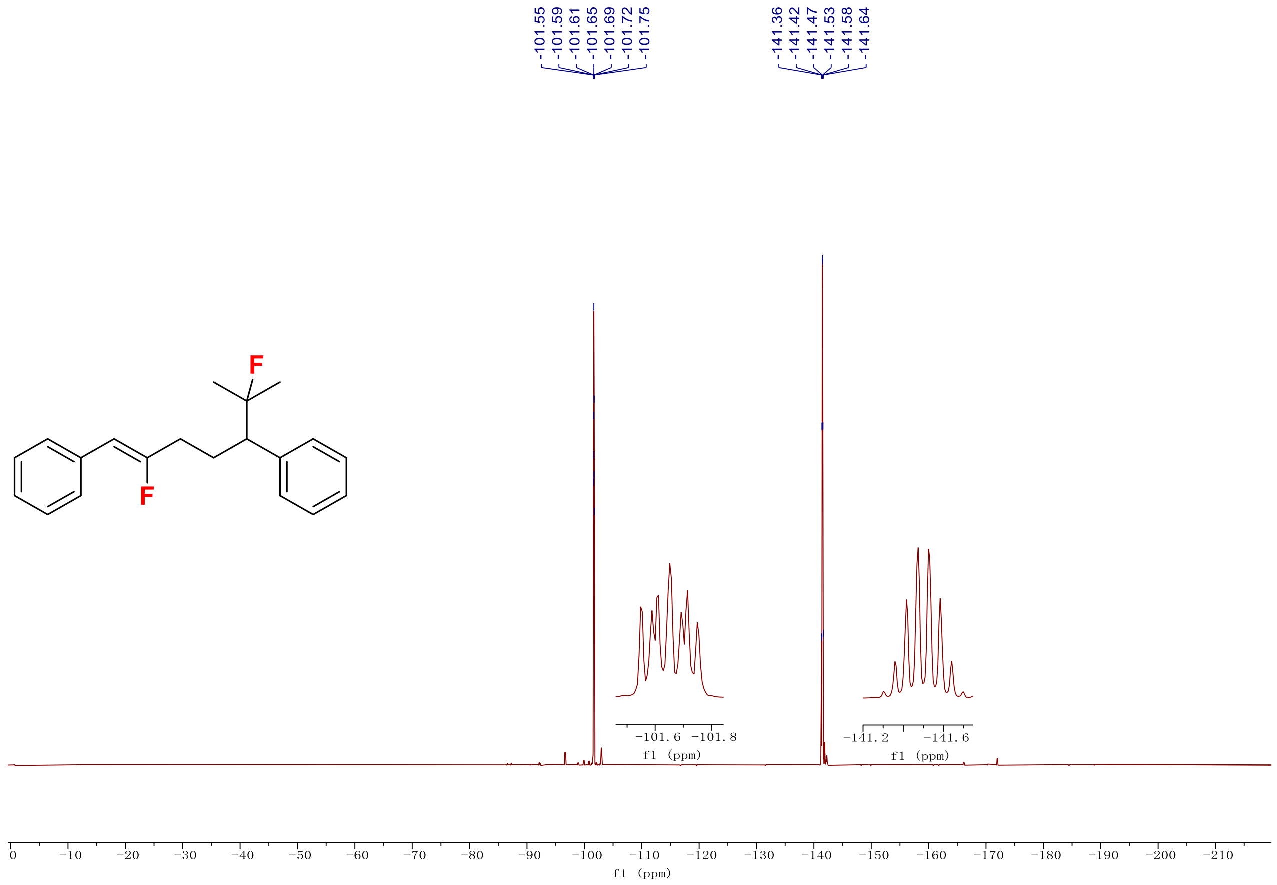

^{1}H NMR (400 MHz, $CDCl_3$) spectrum of $[Rh(CO)_2(BINAP)BF_4]$

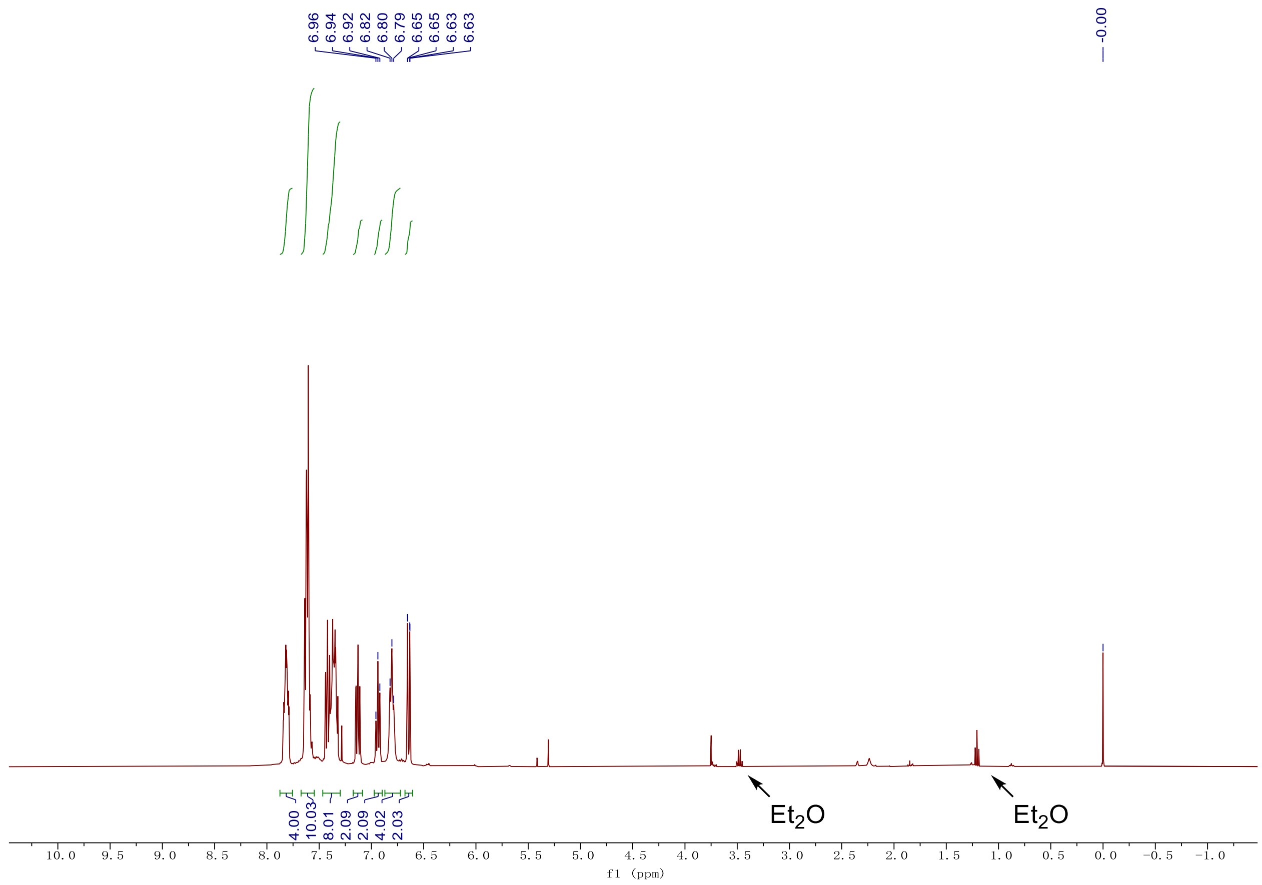

^{13}C NMR (101 MHz, $CDCl_3$) spectrum of $[Rh(CO)_2(BINAP)BF_4]$

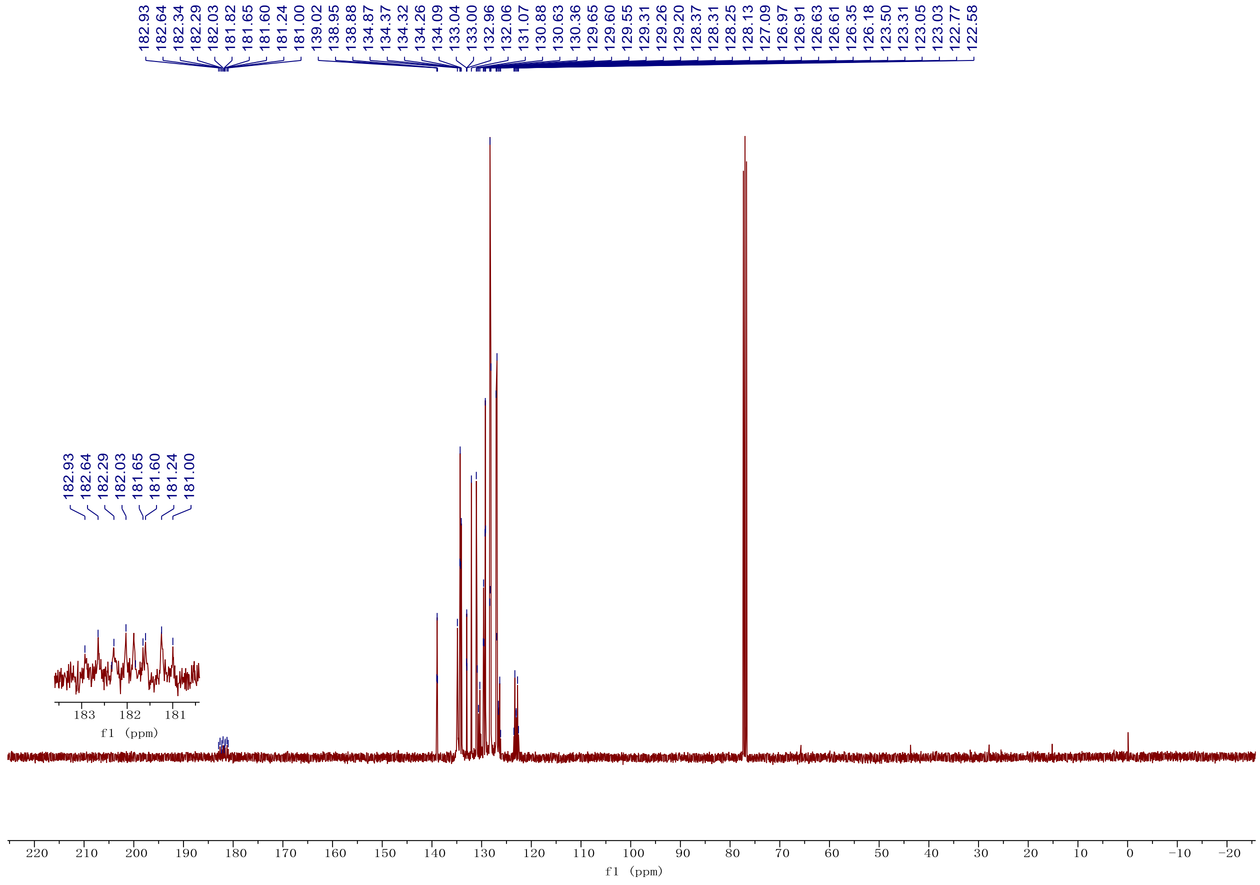

^{31}P NMR (162 MHz, $CDCl_3$) spectrum of $[Rh(CO)_2(BINAP)BF_4]$

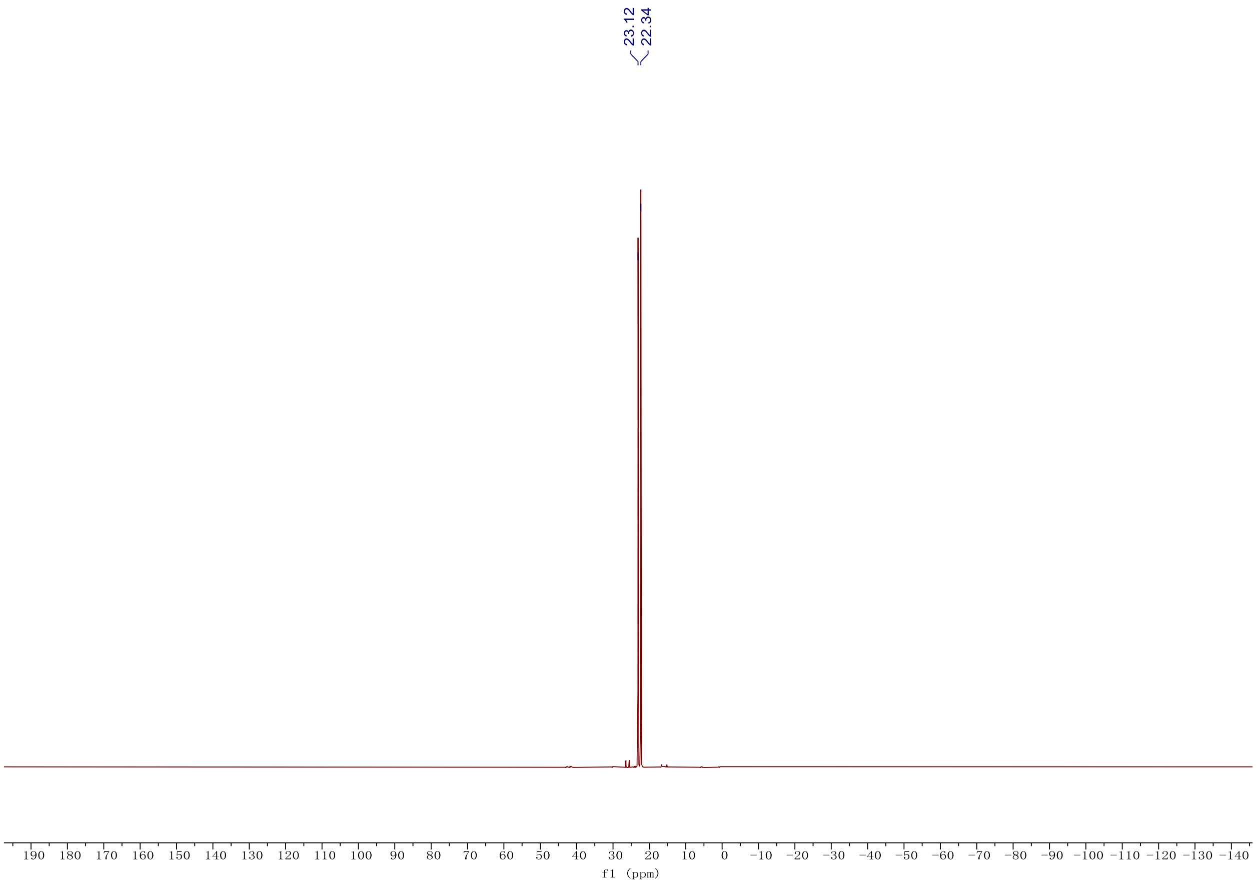

^{19}F NMR (376 MHz, $CDCl_3$) spectrum of $[Rh(CO)_2(BINAP)BF_4]$

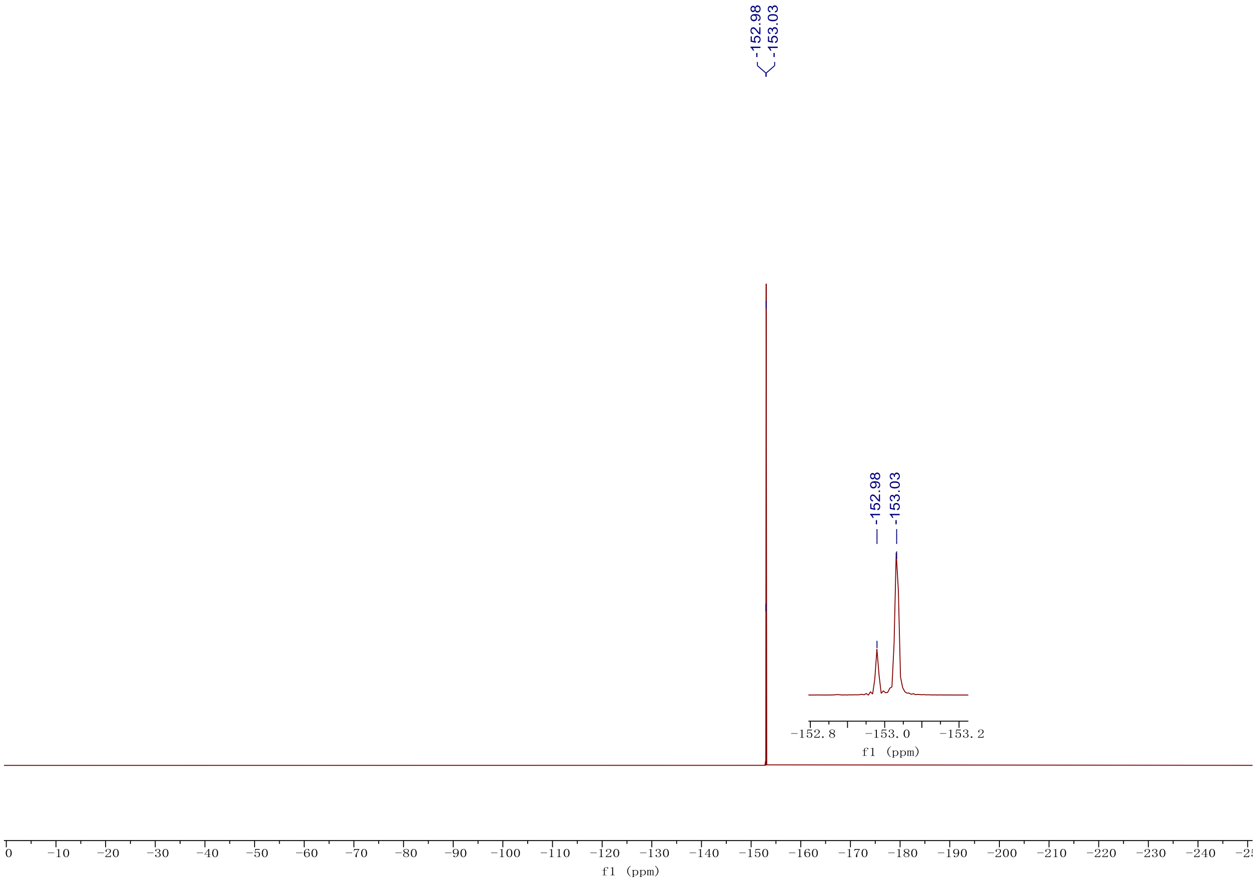

^{1}H NMR (400 MHz, CD_2Cl_2) spectrum of $[Rh(CO)_2(BINAP^{Me})BF_4]$

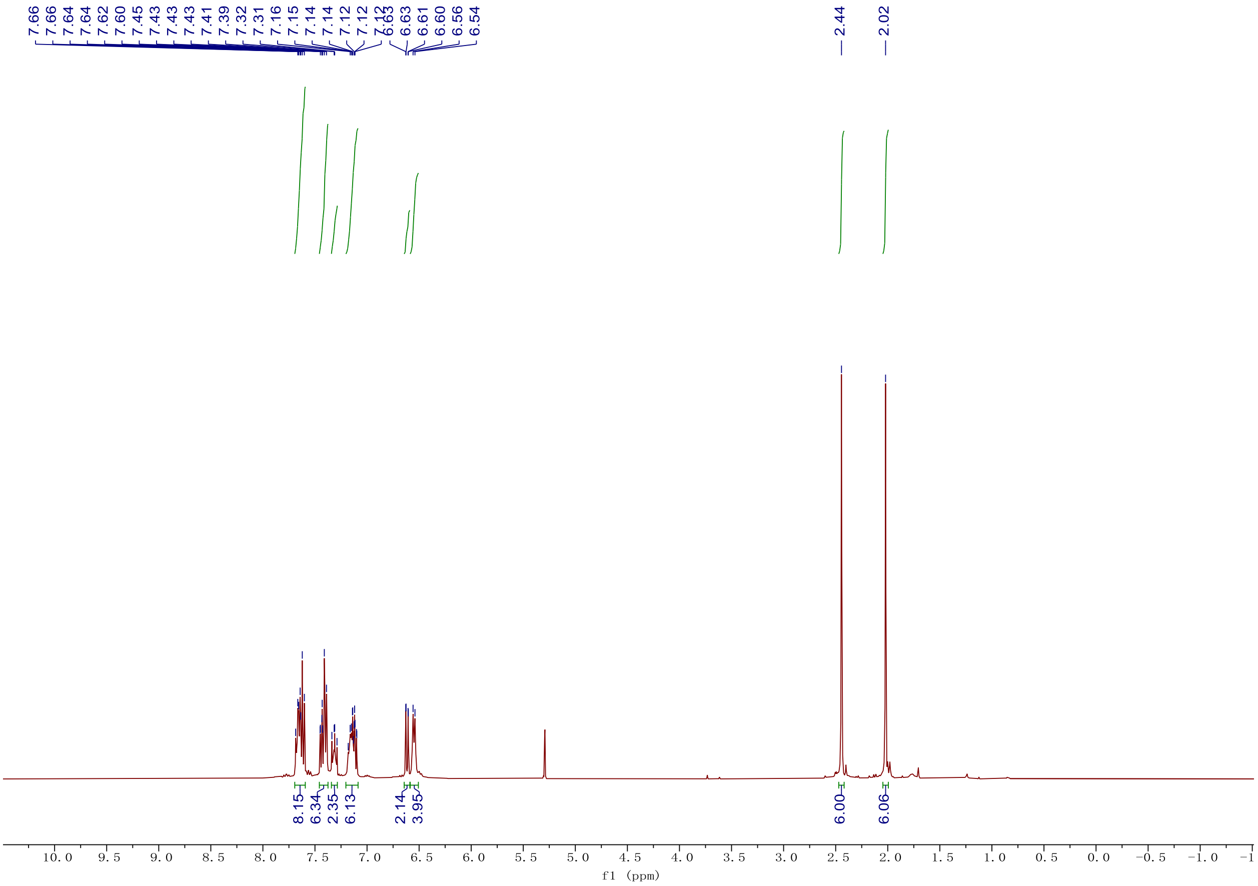

^{13}C NMR (101 MHz, CD_2Cl_2) spectrum of $[Rh(CO)_2(BINAP^{Me})BF_4]$

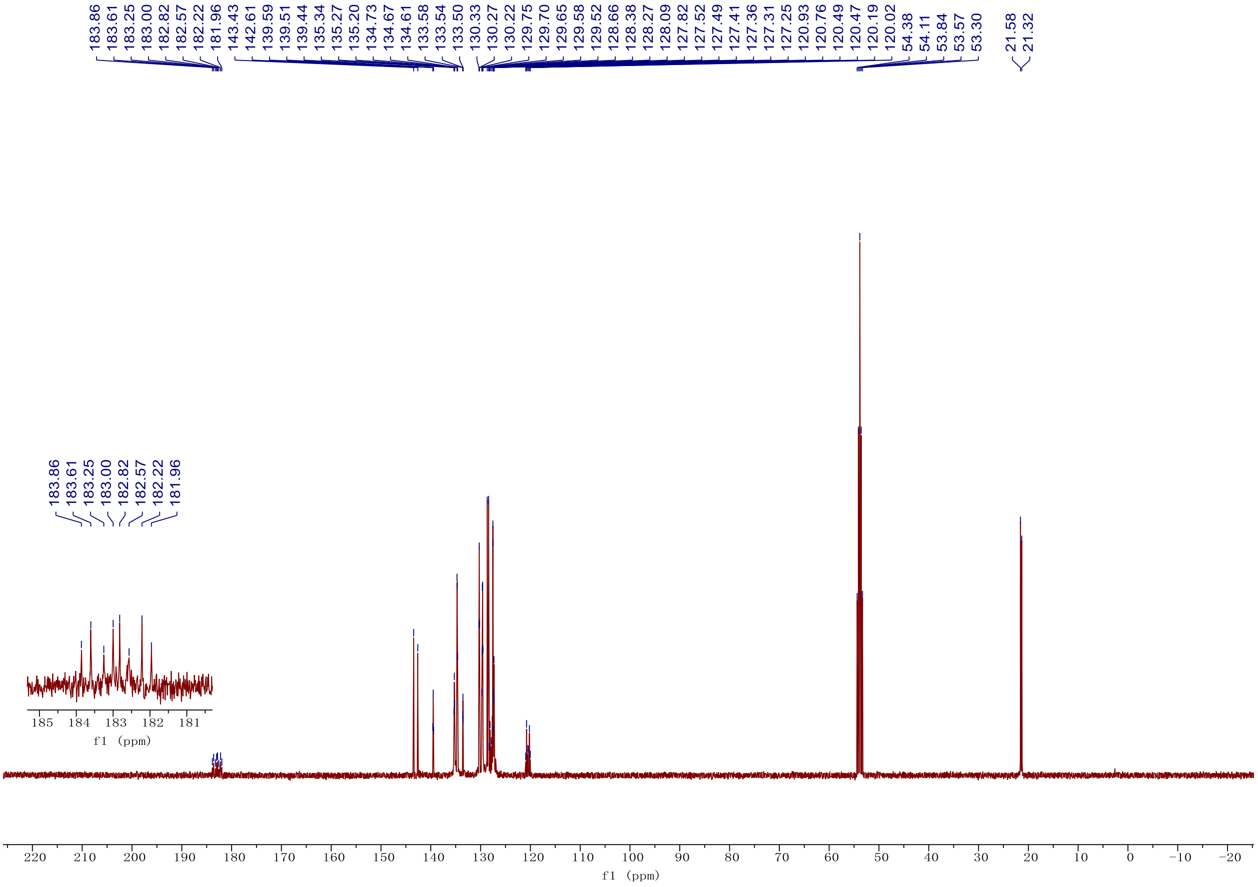

^{31}P NMR (162 MHz, CD_2Cl_2) spectrum of [Rh(CO)$_2$(BINAP^{Me})BF$_4$]

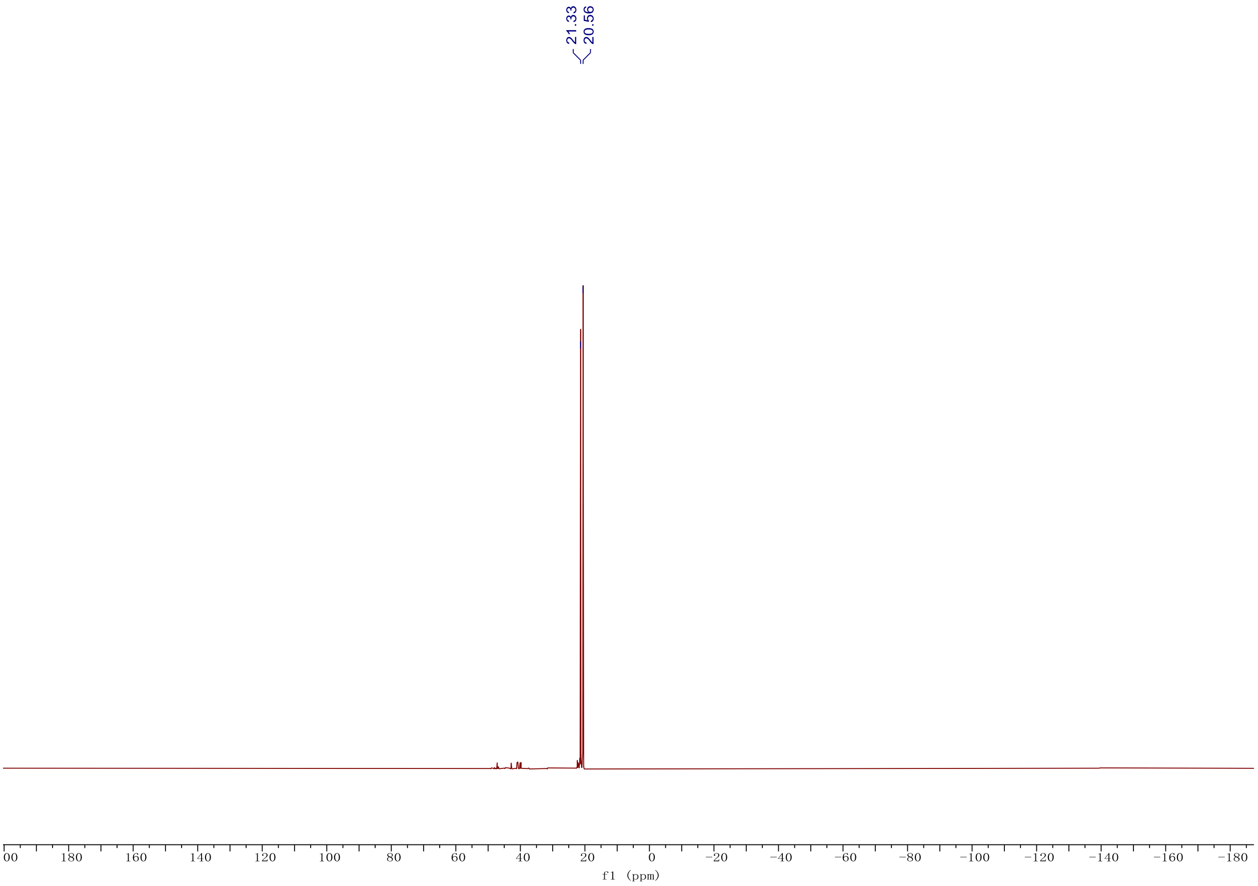

^{19}F NMR (376 MHz, CD_2Cl_2) spectrum of [Rh(CO)$_2$(BINAP^{Me})BF$_4$]

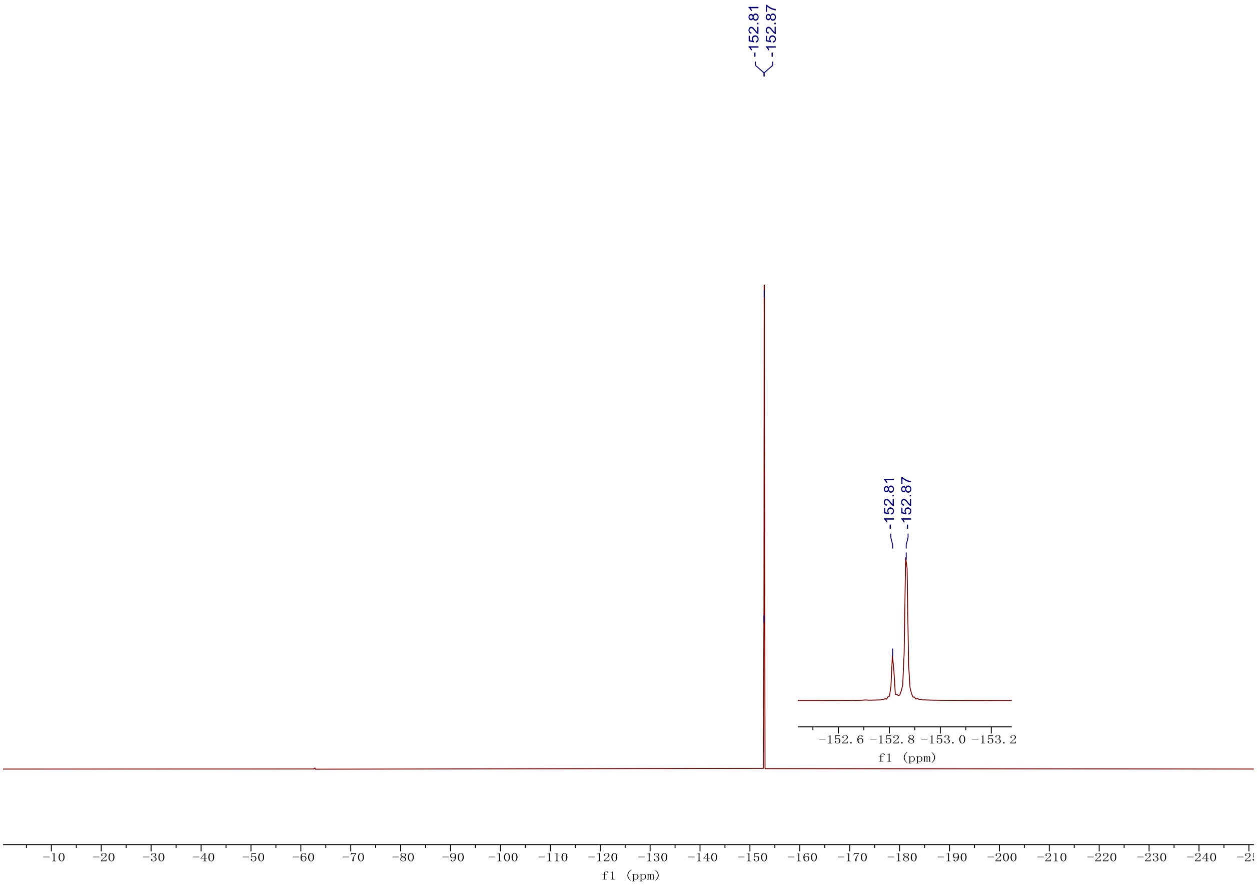

^{1}H NMR (400 MHz, $CDCl_3$) spectrum of [Rh(CO)$_2$(BINAP^{OMe})BF_4]

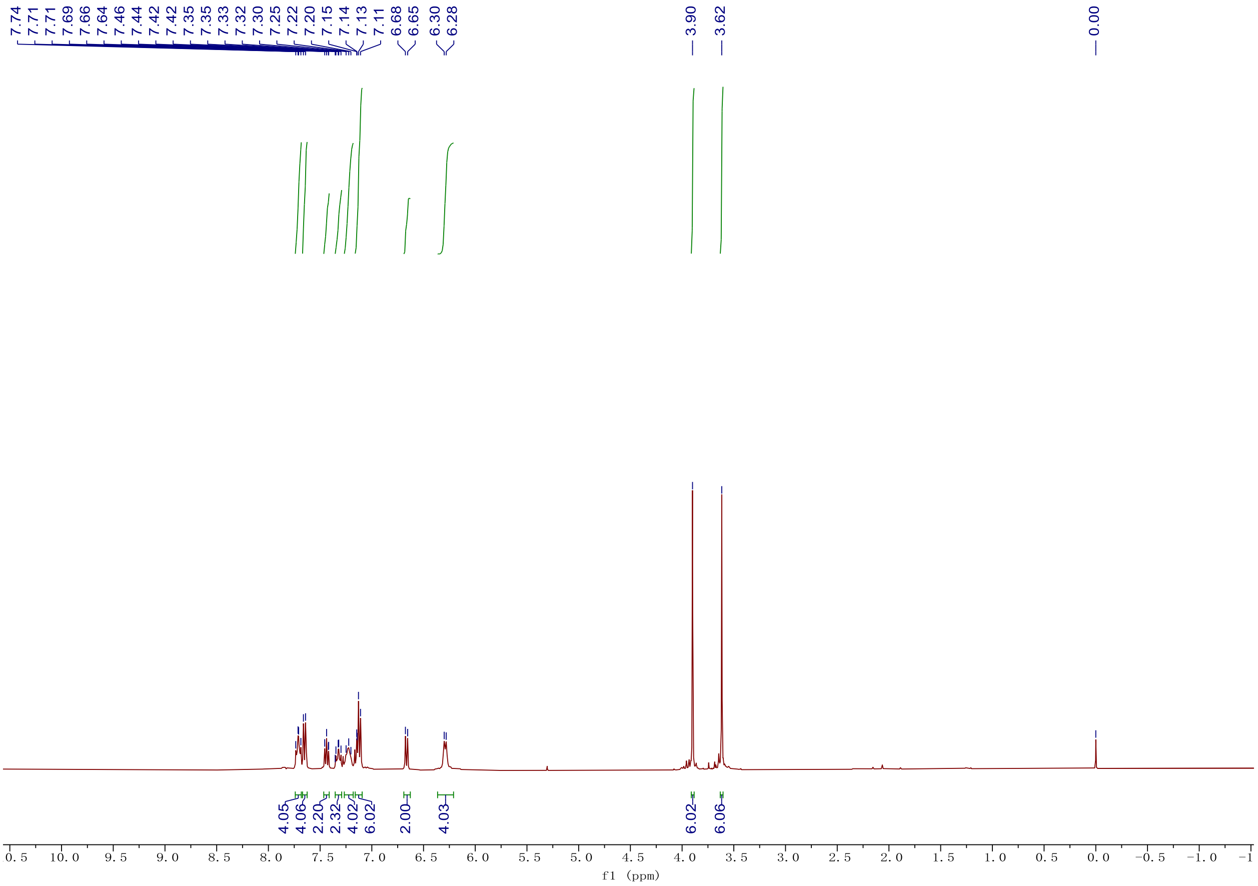

^{13}C NMR (101 MHz, $CDCl_3$) spectrum of [Rh(CO)$_2$(BINAP^{OMe})BF_4]

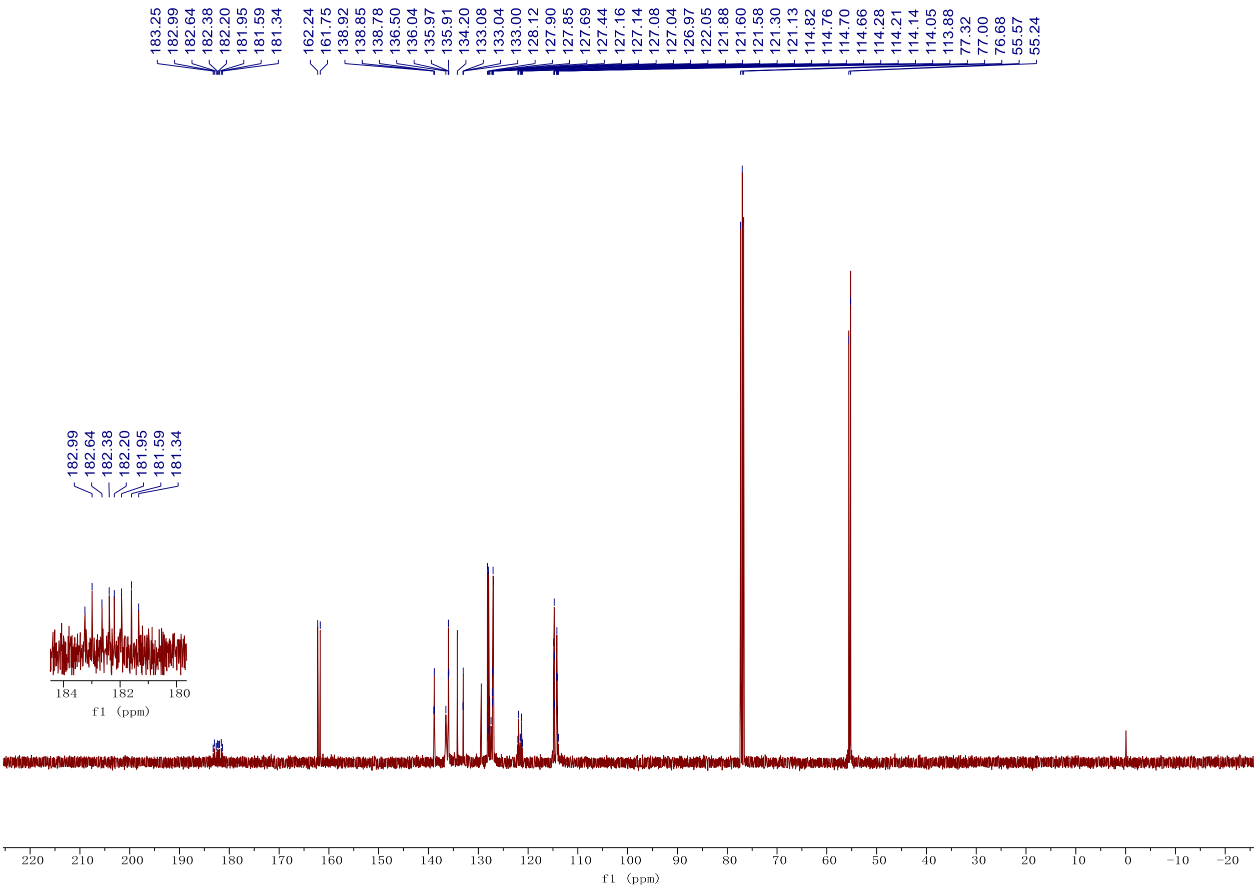

^{31}P NMR (162 MHz, $CDCl_3$) spectrum of $[Rh(CO)_2(BINAP^{OMe})BF_4]$

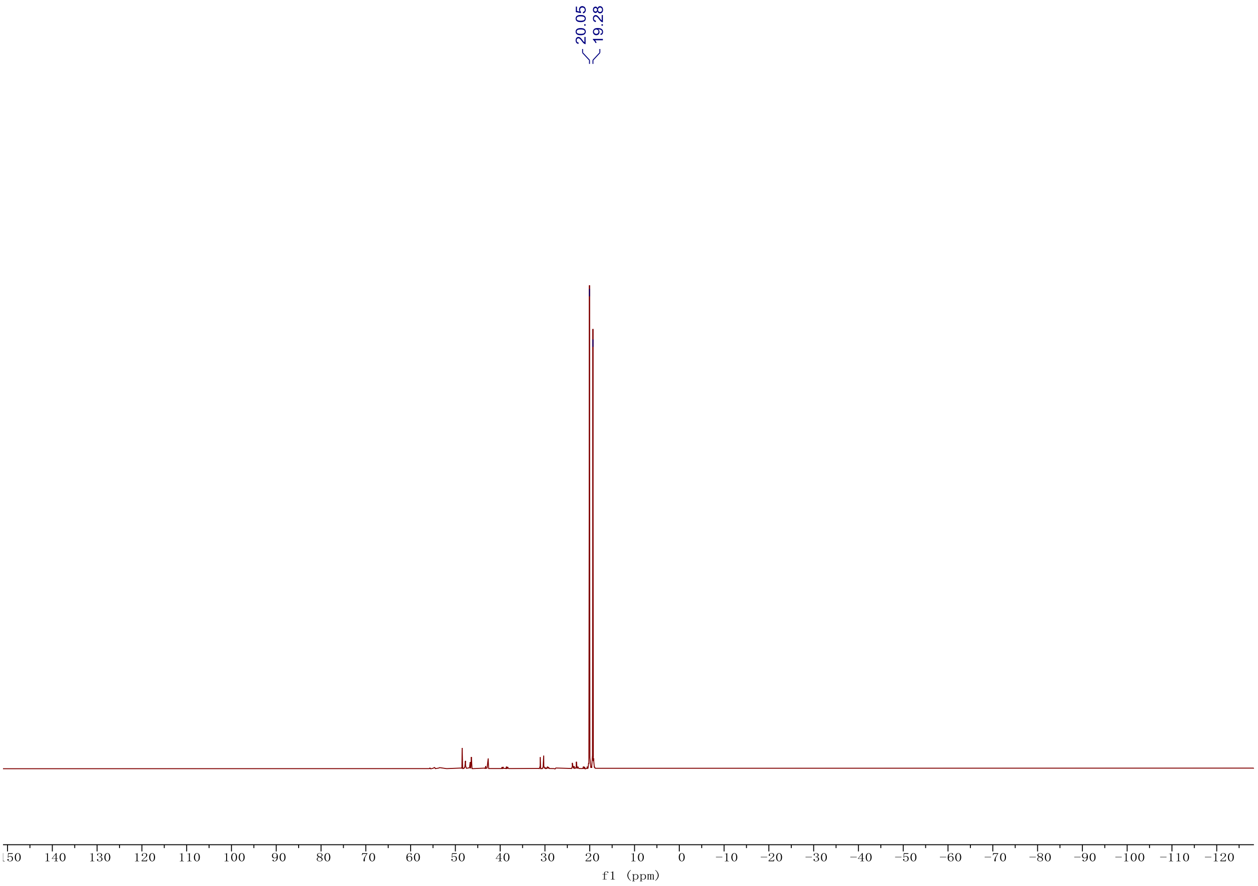

^{19}F NMR (376 MHz, $CDCl_3$) spectrum of $[Rh(CO)_2(BINAP^{OMe})BF_4]$

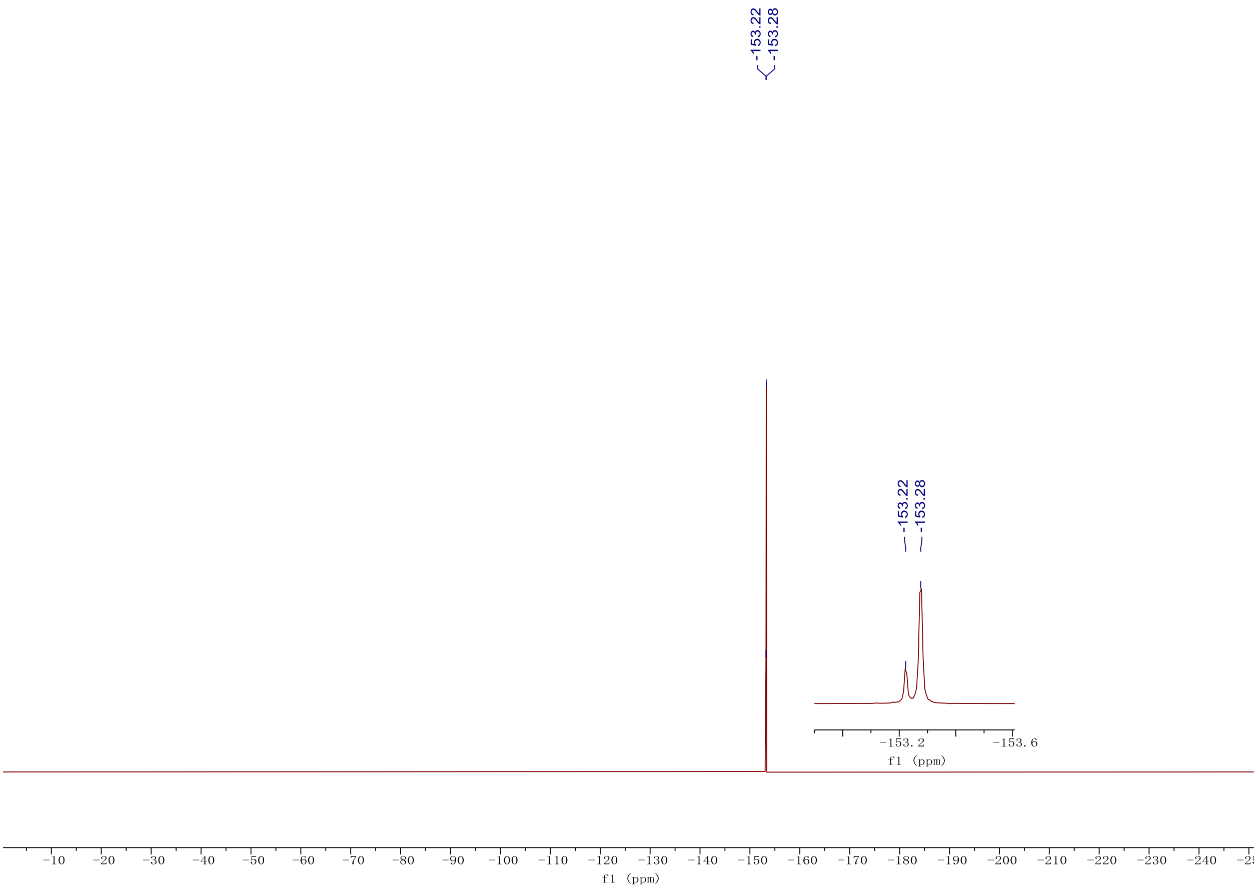

^{1}H NMR (400 MHz, $CDCl_3$) spectrum of $[Rh(CO)_2(BINAP^{NMe2})BF_4]$

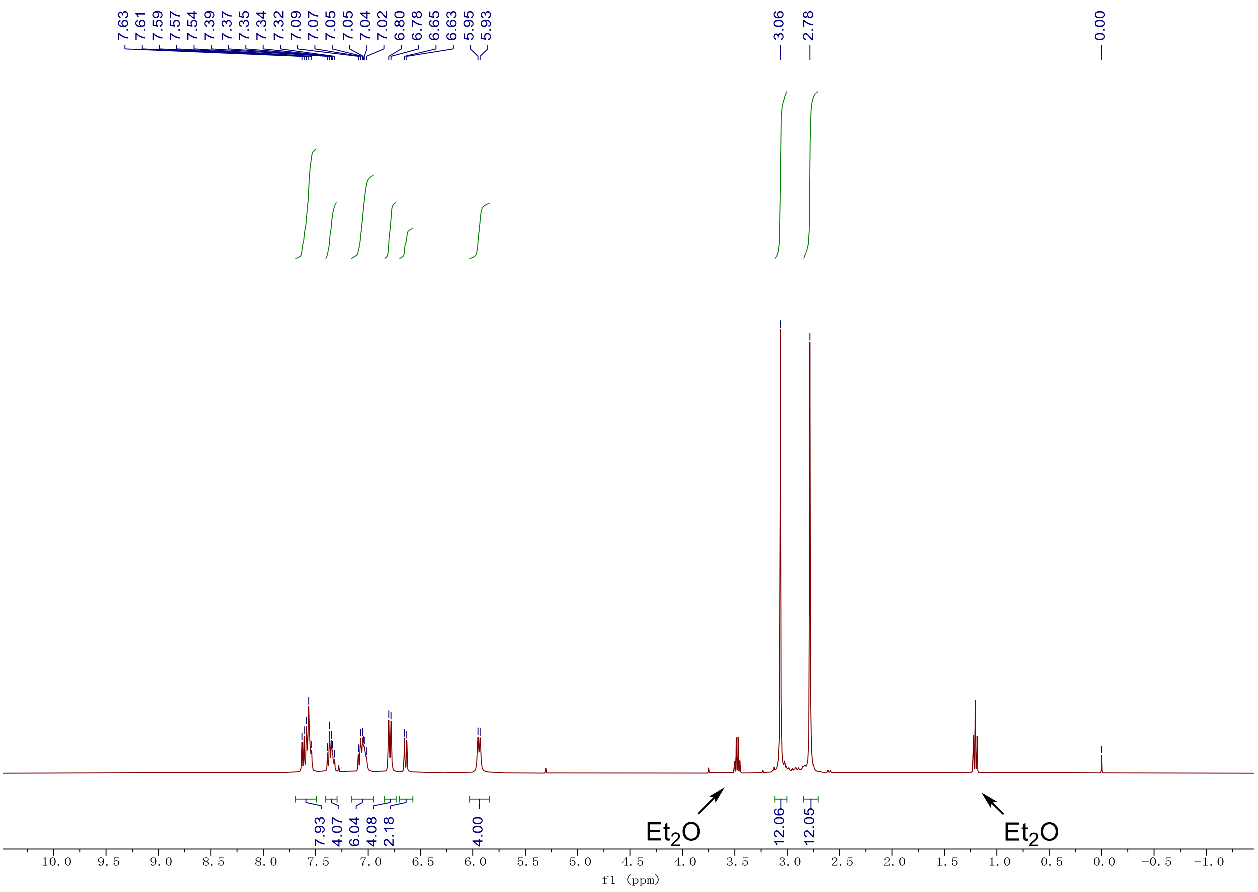

^{13}C NMR (101 MHz, $CDCl_3$) spectrum of $[Rh(CO)_2(BINAP^{NMe2})BF_4]$

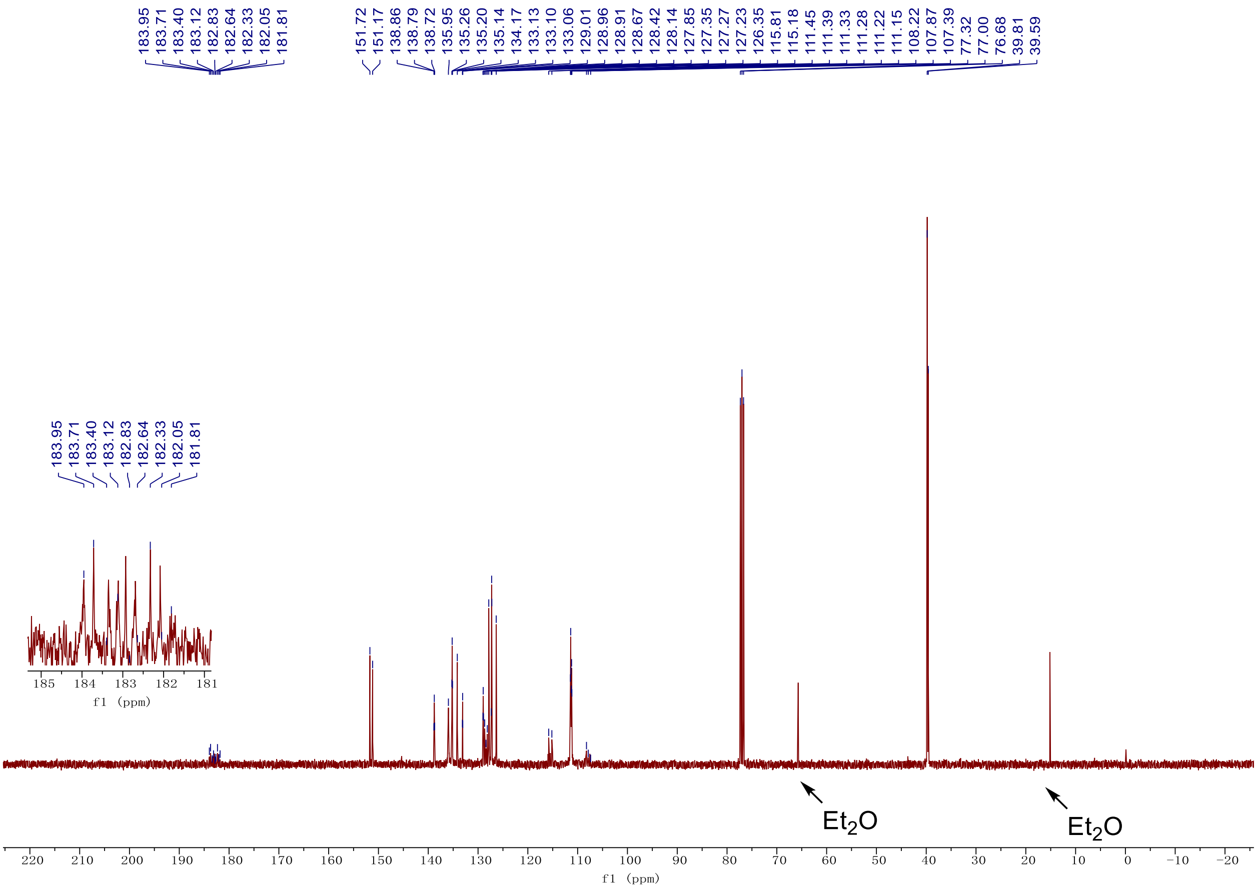

^{31}P NMR (162 MHz, $CDCl_3$) spectrum of [Rh(CO)$_2$(BINAP^{NMe2})BF_4]

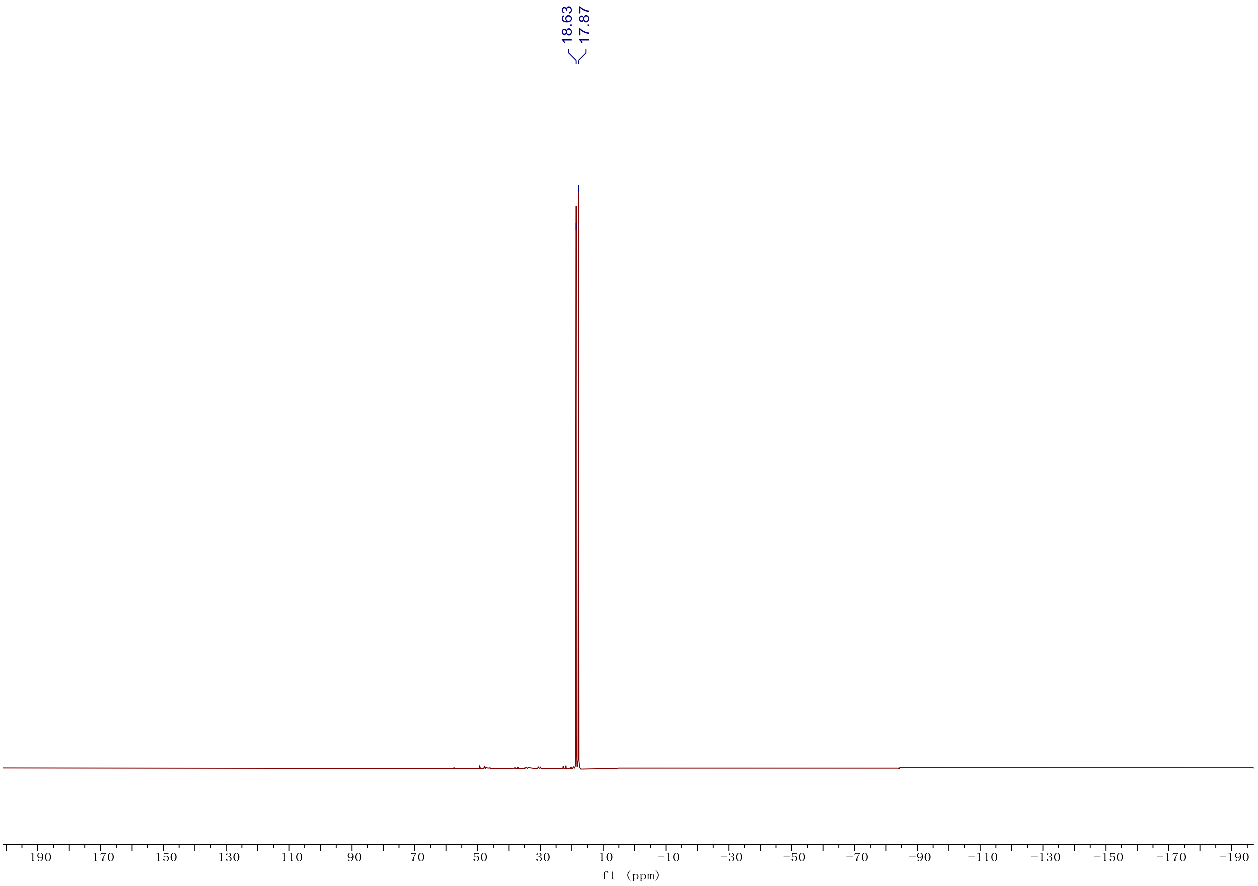

^{19}F NMR (376 MHz, $CDCl_3$) spectrum of [Rh(CO)$_2$(BINAP^{NMe2})BF_4]

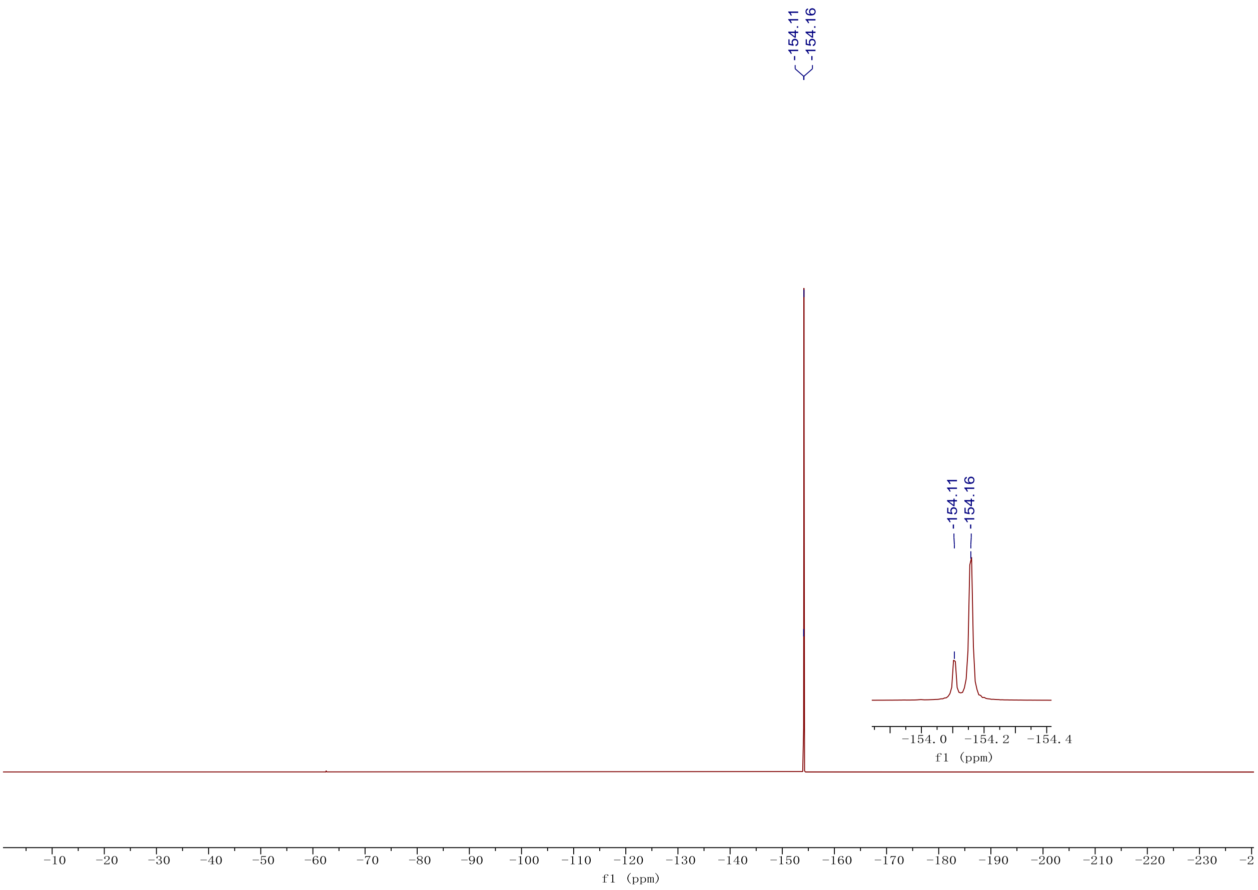

13. References

1. Wang, F. et al. synthesis of *gem*-difluorinated cyclopropanes and cyclopropenes: trifluoromethyltrimethylsilane as a difluorocarbene source. *Angew. Chem. Int. Ed.* **50**, 7153−7157 (2011).
2. Ni, J., Nishonov, B., Pardaev, A. & Zhang, A. Palladium-catalyzed ring-opening coupling of *gem*-difluorocyclopropanes for the construction of 2-fluoroallylic sulfones. *J. Org. Chem.* **84**, 13646−13654 (2019).
3. Xu, J. et al. Pd-catalyzed regioselective activation of *gem*-difluorinated cyclopropanes: a highly efficient approach to 2- fluorinated allylic scaffolds. *Angew. Chem. Int. Ed.* **54**, 8231−8235 (2015).
4. Jiang, Z.-T, Huang, J., Zeng, Y., Hu, F. & Xia, Y. Rhodium catalyzed regioselective C−H allylation of simple arenes via C−C bond activation of *gem*-difluorinated cyclopropanes. *Angew. Chem. Int. Ed.* **60**, 10626−10631 (2021).
5. Zhang, S. et al. Cobalt(II)-catalyzed stereoselective olefin isomerization: facile access to acyclic trisubstituted alkenes. *J. Am. Chem. Soc.* **142**, 8910−8917 (2020).
6. Wang, X., Wang, Z., Asanuma, Y. & Nishihara, Y. Synthesis of 2-substituted propenes by bidentate phosphine-assisted methylenation of acyl fluorides and acyl chlorides with $AlMe_3$. *Org. Lett.* **21**, 3640−3643 (2019).
7. Wahl, J. M., Conner, M. L. & Brown, M. K. Allenoates in enantioselective [2+2] cycloadditions: from a mechanistic curiosity to a stereospecific transformation. *J. Am. Chem. Soc.* **140**, 15943–15949 (2018).
8. Kolb, S. & Werz, D. B. Site-selective hydrogenation/deuteration of benzylic olefins enabled by electroreduction using water. *Chem. Eur. J.* **29**, e202300849 (2023).
9. Walker, J. C. L. & Oestreich, M. Regioselective transfer hydrodeuteration of alkenes with a hydrogen deuteride surrogate using $B(C_6F_5)_3$ catalysis. *Org. Lett.* **20**, 6411–6414 (2018).
10. Deng, Y., Wei, X.-J, Wang, X., Sun, Y. & Noël, T. Iron-catalyzed cross-coupling of alkynyl and styrenyl chlorides with alkyl Grignard reagents in batch and flow. *Chem. Eur. J.* **25**, 14532−14535 (2019).
11. Zhang, Z., Jia, J., Hu, F. & Xia, Y. Aldehyde olefination with arylboroxines enabled by binary rhodium catalysis. *Org. Lett.* **25**, 3228−3233 (2023).

12. Kikuchi, J., Ye, H. & Terada, M. Chiral phosphoric acid catalyzed enantioselective [4+2] cycloaddition reaction of α-fluorostyrenes with imines. *Org. Lett.* **22**, 8957−8961 (2020).
13. Garg, A., Gerwien, N. J., Fasting, C., Charlton, A. & Hopkinson, M. N. Formal insertion of alkenes into C(sp^3)−F bonds mediated by fluorine-hydrogen bonding. *Angew. Chem. Int. Ed.* **62**, e202302860 (2023).
14. Gauthier, R. et al. Gold N-Heterocyclic carbene catalysts for the hydrofluorination of alkynes using hydrofluoric acid: reaction scope, mechanistic studies and the tracking of elusive intermediates. *Chem. Eur. J.* **28**, e202103886 (2022).
15. Ngo Ndimba, A., Vincent, É. & Brioche, J. Radical amido- and azido-fluorination of α-fluorostyrene derivatives: an innovative approach towards β-aryl-β,β-difluoroamino motifs. *Eur. J. Org. Chem.* **2022**, e202201165 (2022).
16. Luo, H. & Loh, T. Synthesis of aryl allylic fluorides by direct electrophilic fluorination of alkenes. *Tetrahedron. Lett.* **50**, 1554−1556 (2009).
17. Tanaka, S., Watanabe, K., Tanaka, Y. & Hattori, T. $EtAlCl_2$/2,6-disubstituted pyridine-mediated carboxylation of alkenes with carbon dioxide. *Org. Lett.* **18**, 2576−2579 (2016).
18. Kanemoto, S., Shimizu, M. & Yoshioka, H. Chemoselective synthesis of homoallylic fluorides from cyclopropylmethanols by ring opening. *Tetrahedron Lett.* **28**, 663−666 (1987).
19. Kanemoto, S., Shimizu, M. & Yoshioka, H. Ring-opening fluorination and ring-expansion fluorination of cyclopropanemethanols with amine/metal fluoride/poly(hydrogen fluoride)–pyridine complex. *Bull. Chem. Soc. Jpn.* **62**, 2024−2031 (1989).
20. Peng, P., Lu, Q., Peng, L., Liu, C., Wang, G. & Lei, A. Dioxygen-induced oxidative activation of a P–H bond: radical oxyphosphorylation of alkenes and alkynes toward β-oxy phosphonates. *Chem. Commun.* **52**, 12338−12341 (2016).
21. Yamada, M., Goto, M. & Yamano, M. Direct conversion of sec-phosphine oxides to sec-phosphine-boranes using BH_3. *Tetrahedron Lett.* **67**, 152837 (2021).
22. Xiao, D., Zhang, Z. & Zhang, X. Synthesis of a novel chiral binaphthyl phospholane and its application in the highly enantioselective hydrogenation of enamides. *Org. Lett.* **1**, 1679−1681 (1999).

23. Goto, M. et al. Process research on the asymmetric hydrogenation of a benzophenone for developing the manufacturing process of the squalene synthase inhibitor TAK-475. *Org. Process Res. Dev.* **15**, 1178−1184 (2011).
24. Bürgi, J. J. et al.Unprecedented selectivity via electronic substrate recognition in the 1,4-addition to cyclic olefins using a chiral disulfoxide rhodium catalyst. *Angew. Chem. Int. Ed.* **48**, 2768−2771 (2009).
25. Lv, L. & Li, C.-J. Palladium-catalyzed defluorinative alkylation of *gem*-difluorocyclopropanes: switching regioselectivity via simple hydrazones. *Angew. Chem. Int. Ed.* **60**, 13098−13104 (2021).
26. Champagne, P. A., Benhassine, Y., Desroches, J. & Paquin, J. Friedel–crafts reaction of benzyl fluorides: selective activation of C–F bonds as enabled by hydrogen bonding. *Angew. Chem. Int. Ed.* **53**, 13835–13839 (2014).
27. Dai, W., Xiao, J., Jin, G., Wu, J. & Cao, S. Palladium- and nickel-Ccatalyzed kumada cross-coupling reactions of *gem*-difluoroalkenes and monofluoroalkenes with Grignard reagents *J. Org. Chem*. **79**, 10537−10546 (2014).
28. Cresswell, A. J., Davies, S. G., Roberts, P. M. & Tomson, J. E. Beyond the Balz−Schiemann reaction: the utility of tetrafluoroborates and boron trifluoride as nucleophilic fluoride sources. *Chem. Rev.* **115**, 566−611 (2015).
29. Frisch, M. J. et al. Gaussian 16 Rev. C.01, Wallingford, CT, 2016.
30. Zhao, Y. & Truhlar, D. G. Density functionals with broad applicability in chemistry. *Acc. Chem. Res*. **41**, 157−167 (2008).
31. Marenich, A. V., Cramer, C. J. & Truhlar, D. G. Universal solvation model based on solute electron density and on a continuum model of the solvent defined by the bulk dielectric constant and atomic surface tensions. *J. Phys. Chem. B.* **113**, 6378−6396 (2009).
32. Zeng, Y. et al. Site-divergent alkenyl C–H fluoroallylation of olefins enabled by tunable rhodium catalysis. *ACS Catal.* **12**, 8857−8867 (2022).
33. Lu, G., Fang, C., Xu, T., Dong, G. & Liu, P. Computational study of Rh-catalyzed carboacylation of olefins: ligand-promoted rhodacycle isomerization enables regioselective C–C bond functionalization of benzocyclobutenones. *J. Am. Chem. Soc.* **137**, 8274−8283 (2015).
34. Xia, Y., Lu, G., Liu, P. & Dong, G. Catalytic activation of carbon–carbon bonds in

cyclopentanones. *Nature* **539**, 546−550 (2016).
