# Supplementary material for: Observation of unusual outer-sphere mechanism using simple alkenes as nucleophiles in allylation chemistry
Source: Nat Commun. 2024 May 21;15:4317. doi: 10.1038/s41467-024-48541-5 (PMC11109239; doi:10.1038/s41467-024-48541-5)
Supplement: Supplementary file 3 — Description of Additional Supplementary Files [file 41467_2024_48541_MOESM3_ESM.pdf]

Description of Additional Supplementary Files

Supplementary Data 1: Cartesian coordinates of the calculated structures.
